# Supplementary figures and images for: Spatial and temporal epidemiology of SARS-CoV-2 virus lineages in Teesside, UK, in 2020: effects of socio-economic deprivation, weather, and lockdown on lineage dynamics (part 2 of 2)
Source: Peer Community J. Author manuscript; Available in PMC 2024 Sep 23. (PMC7616629; doi:10.24072/pcjournal.461)

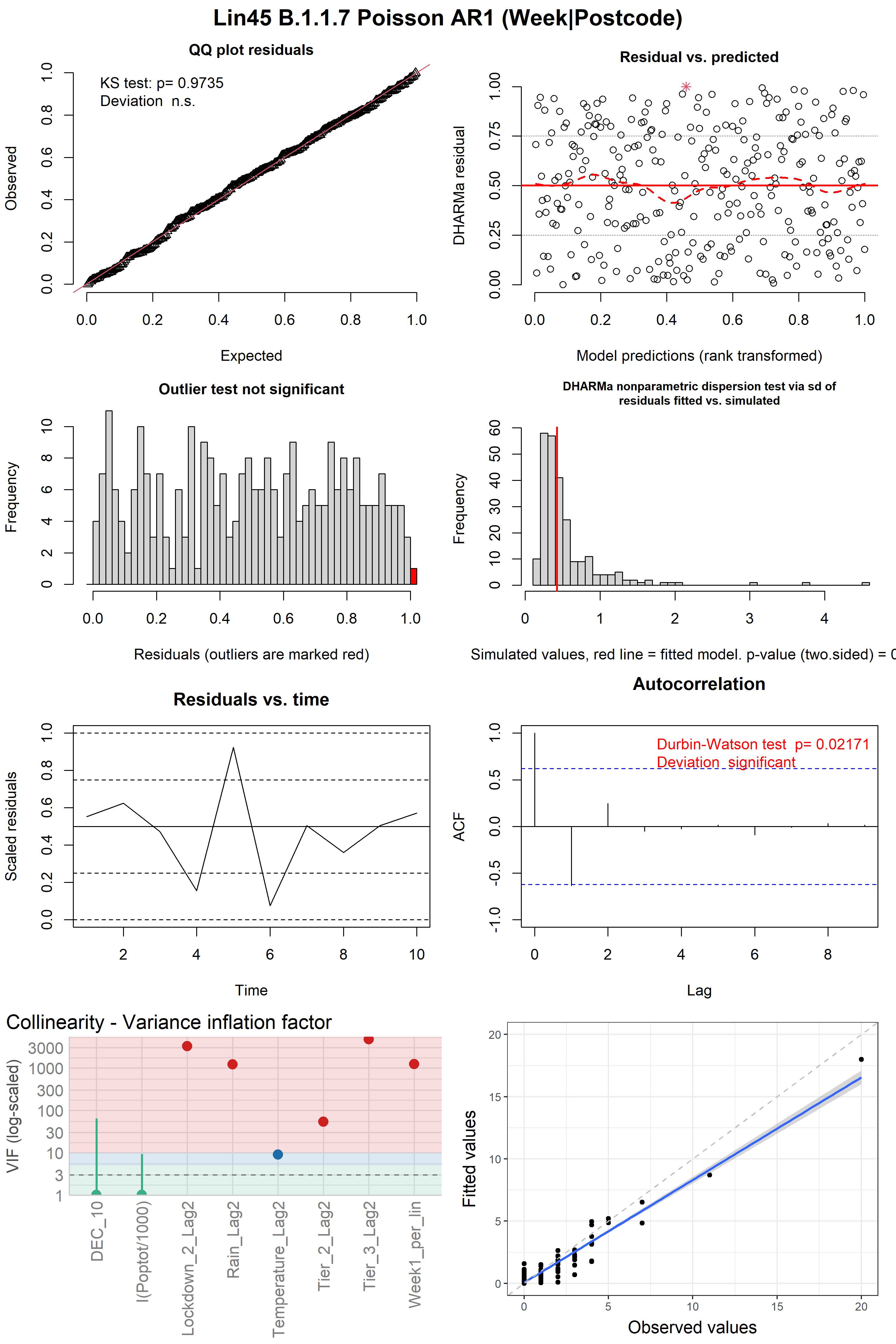

Supplement: Supplementary file: main dataset and code (compressed) [file EMS198536-supplement-Supplementary_file__main_dataset_and_code__compressed_.zip › Covid-19-Teesside-main/Figures/GLMM/Lin45/Lin45-B117_Po_AR1-Week-Postcode_Fit.png]

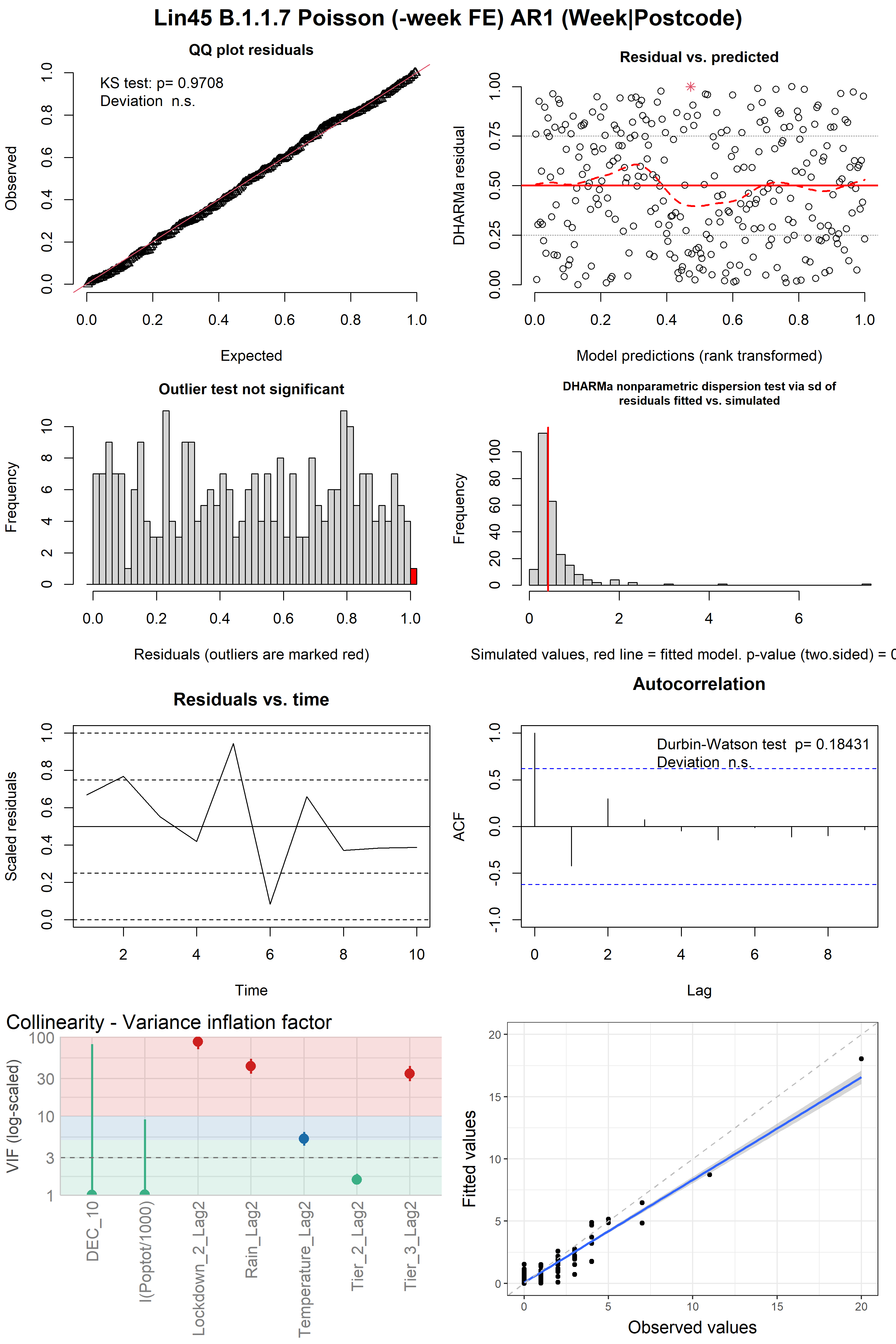

Supplement: Supplementary file: main dataset and code (compressed) [file EMS198536-supplement-Supplementary_file__main_dataset_and_code__compressed_.zip › Covid-19-Teesside-main/Figures/GLMM/Lin45/Lin45-B117_Po_AR1-Week-Postcode_No-week-FE_Fit.png]

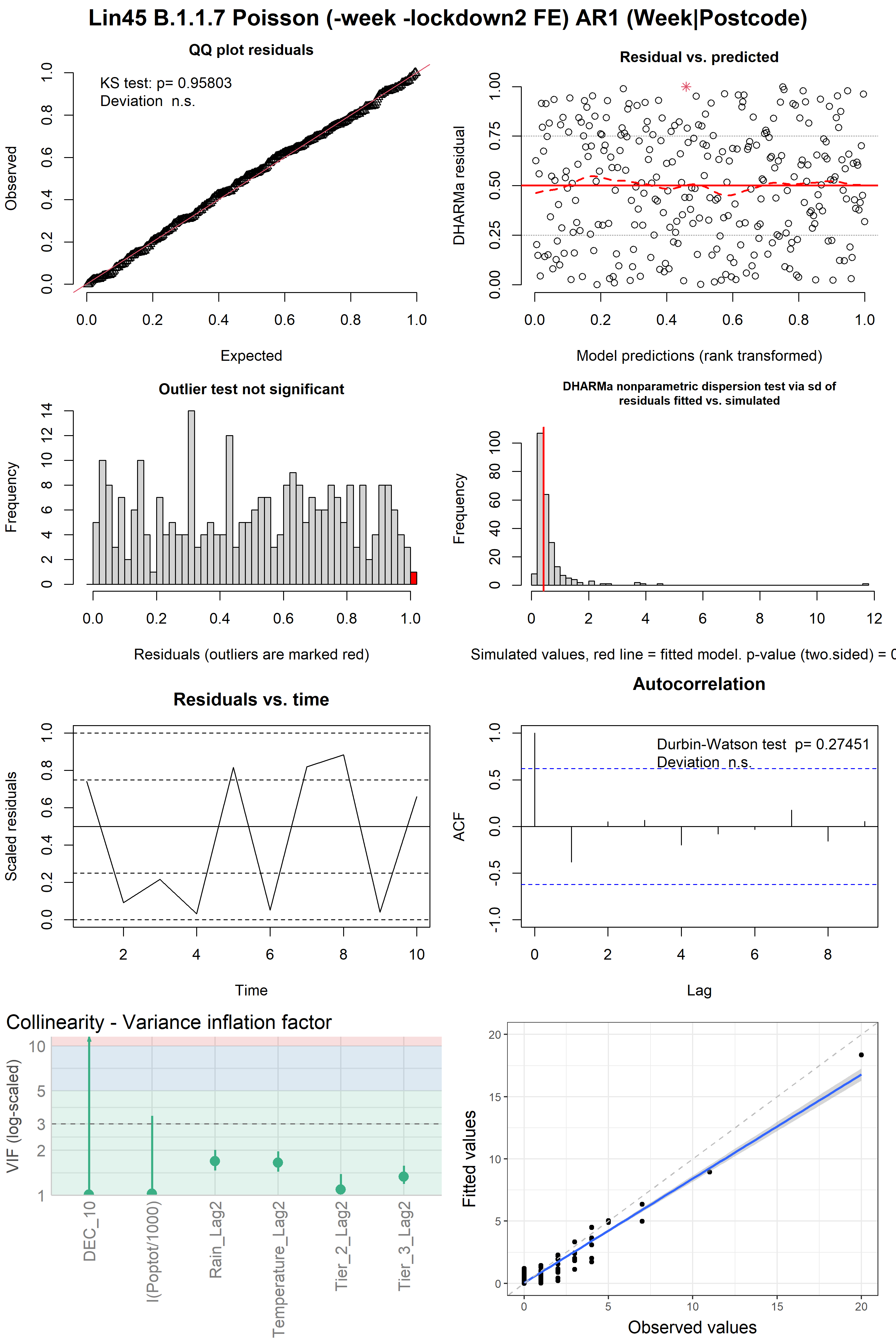

Supplement: Supplementary file: main dataset and code (compressed) [file EMS198536-supplement-Supplementary_file__main_dataset_and_code__compressed_.zip › Covid-19-Teesside-main/Figures/GLMM/Lin45/Lin45-B117_Po_AR1-Week-Postcode_No-week-no-lockdown2-FE_Fit.png]

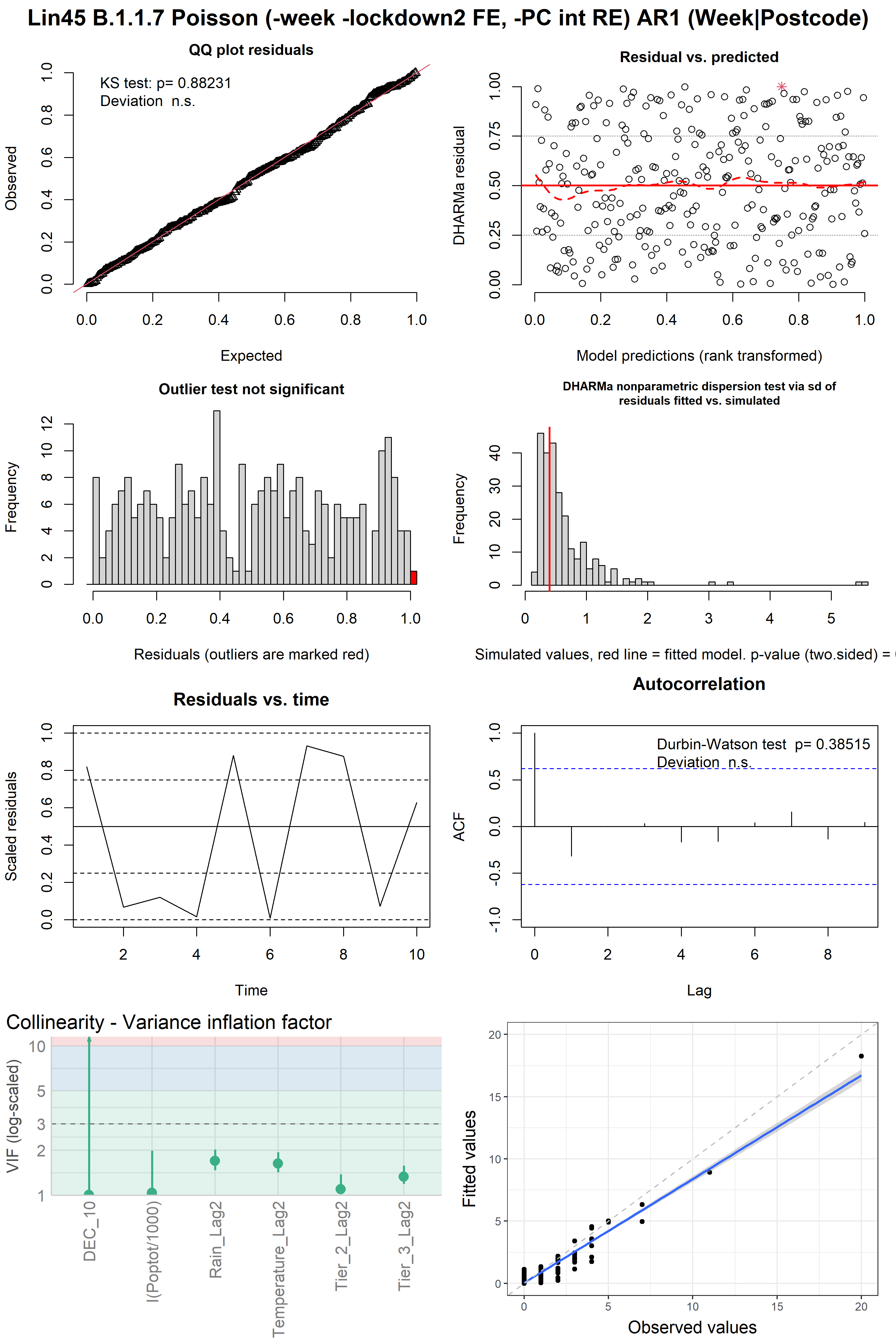

Supplement: Supplementary file: main dataset and code (compressed) [file EMS198536-supplement-Supplementary_file__main_dataset_and_code__compressed_.zip › Covid-19-Teesside-main/Figures/GLMM/Lin45/Lin45-B117_Po_AR1-Week-Postcode_No-week-no-lockdown2-FE_No-PC-int-RE_Fit.png]

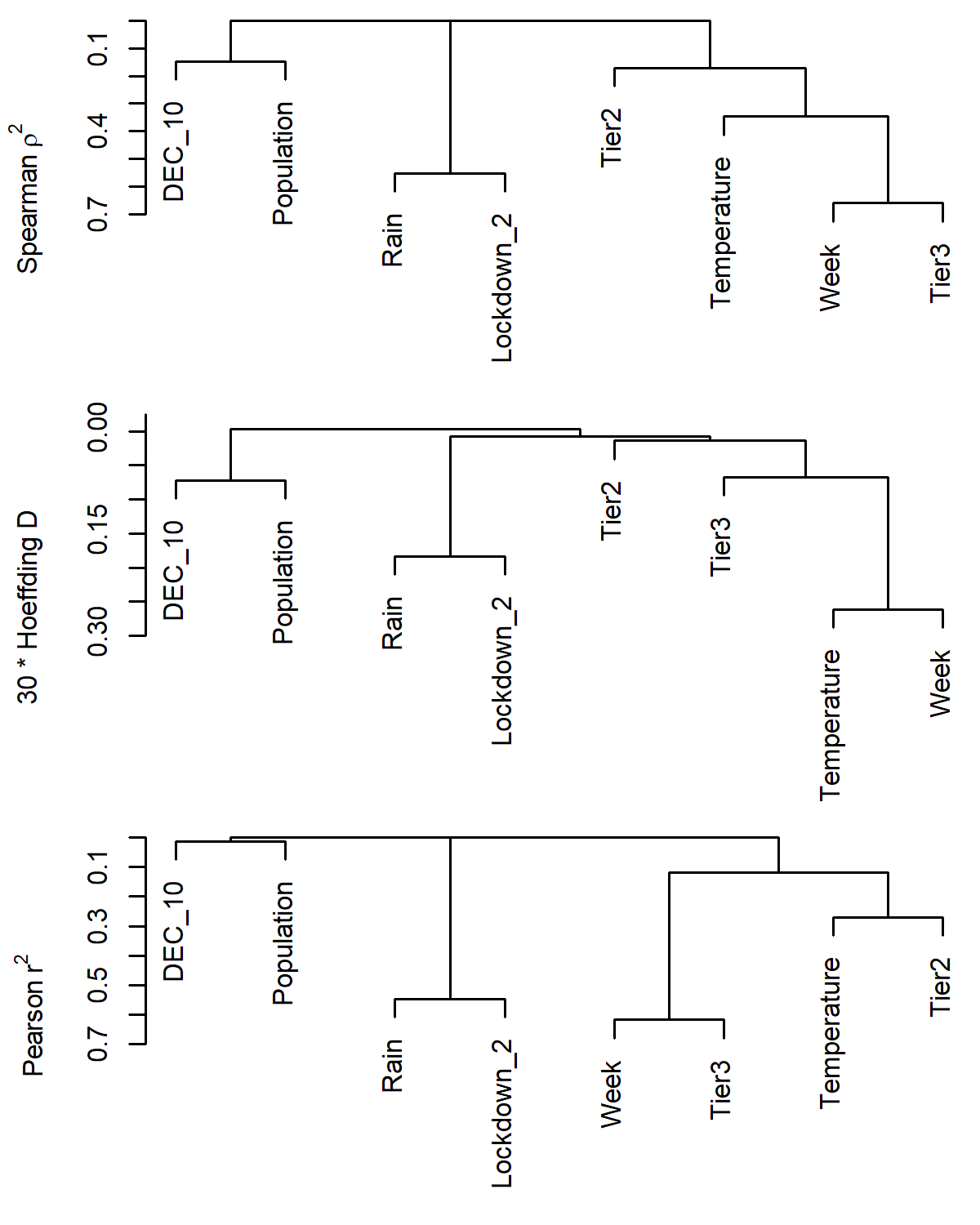

Supplement: Supplementary file: main dataset and code (compressed) [file EMS198536-supplement-Supplementary_file__main_dataset_and_code__compressed_.zip › Covid-19-Teesside-main/Figures/GLMM/Lin45/Lin45-B117_Variable-Clustering.png]

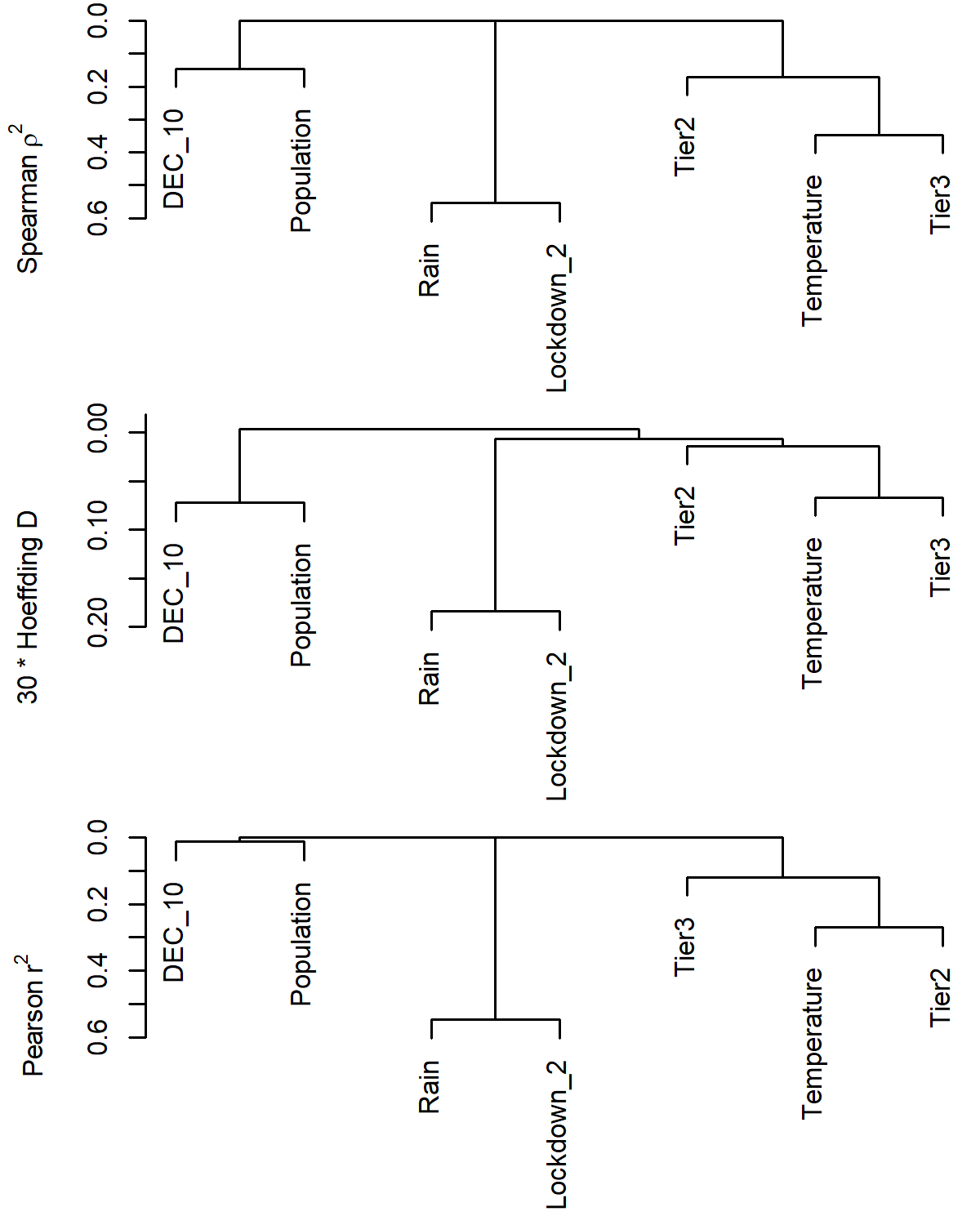

Supplement: Supplementary file: main dataset and code (compressed) [file EMS198536-supplement-Supplementary_file__main_dataset_and_code__compressed_.zip › Covid-19-Teesside-main/Figures/GLMM/Lin45/Lin45-B117_Variable-Clustering_Without-Week.png]

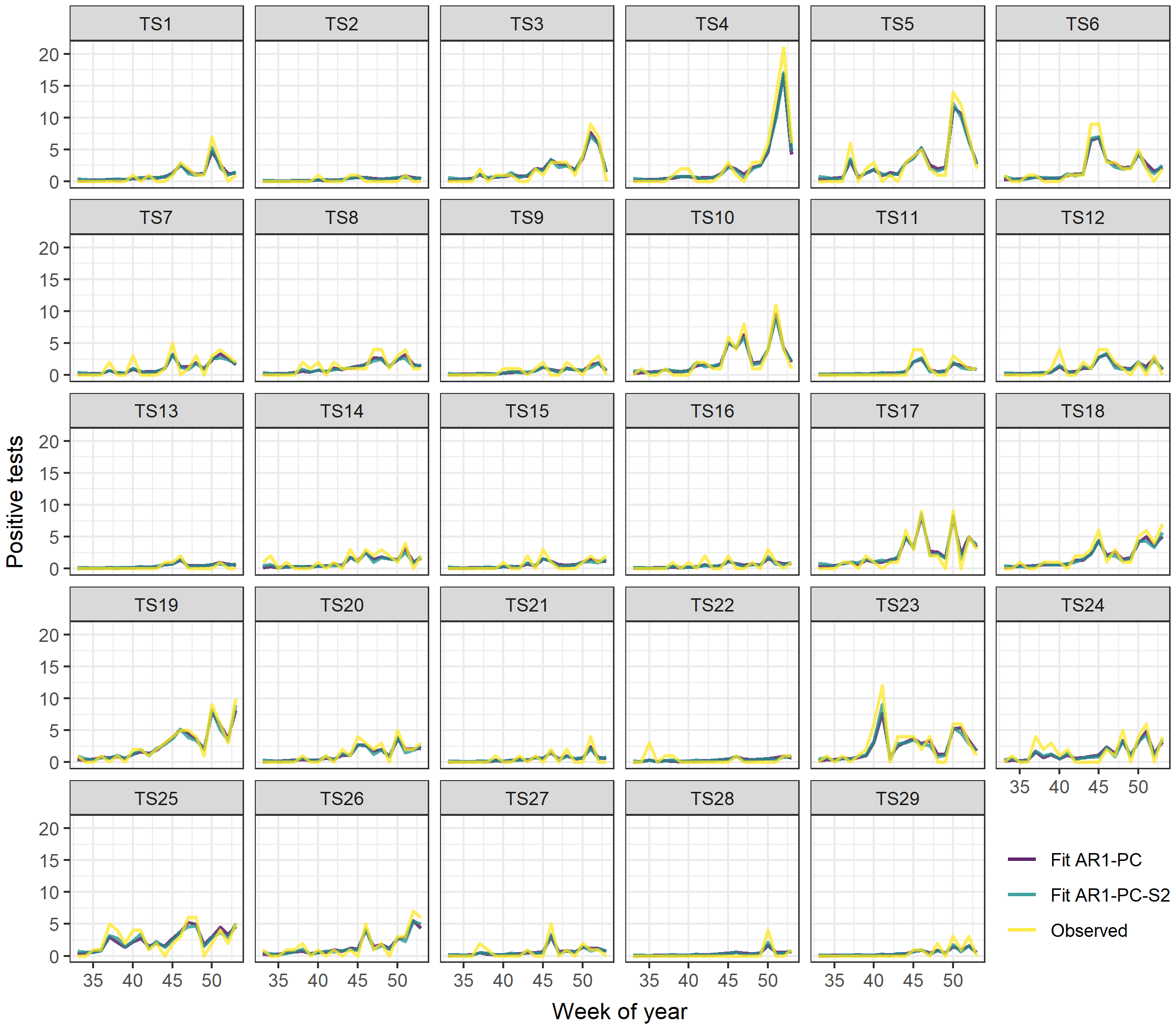

Supplement: Supplementary file: main dataset and code (compressed) [file EMS198536-supplement-Supplementary_file__main_dataset_and_code__compressed_.zip › Covid-19-Teesside-main/Figures/GLMM/Lin51/Lin51-B1177_GLMM_Obs-vs-Fit_AR1PC-AR1PCS2.png]

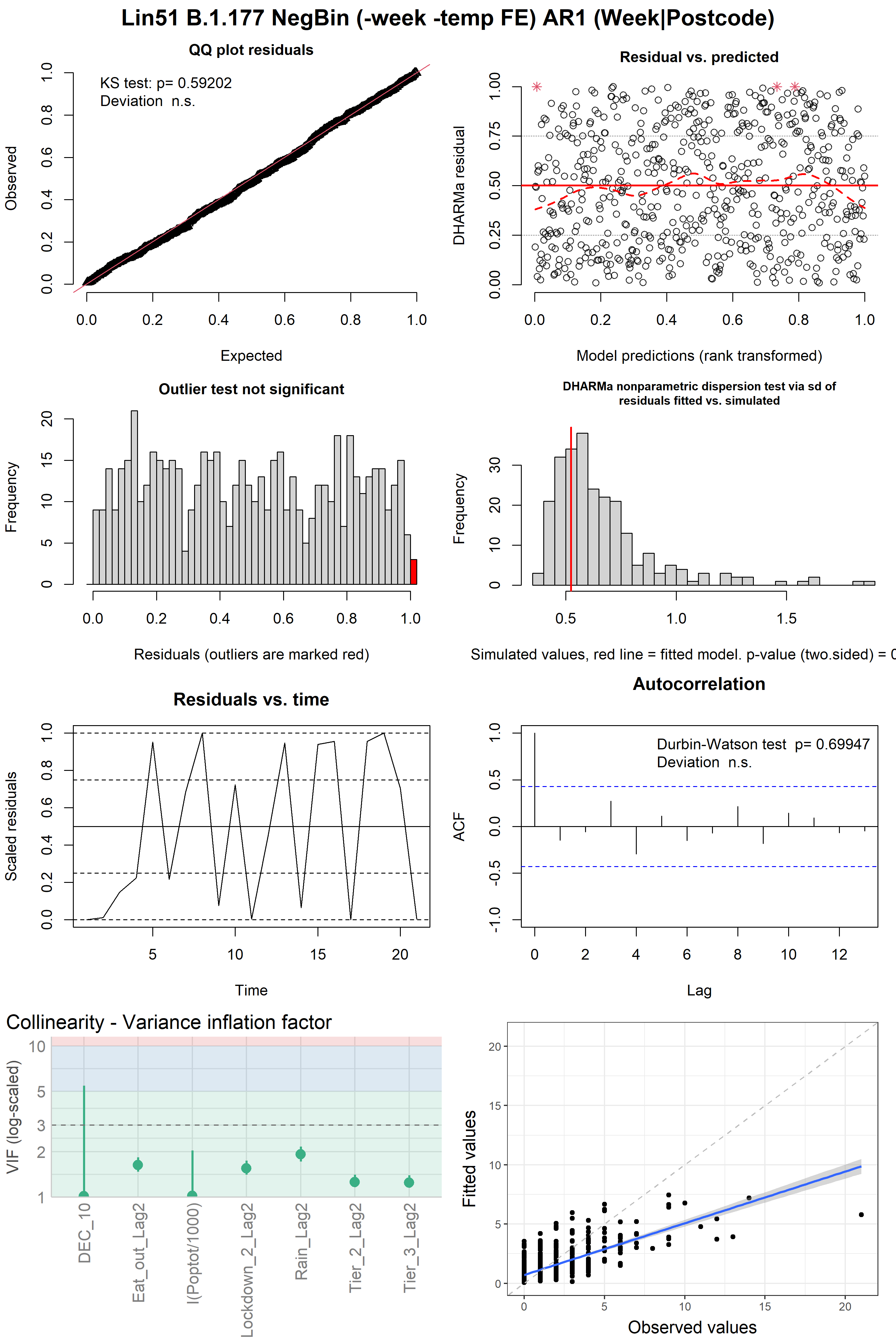

Supplement: Supplementary file: main dataset and code (compressed) [file EMS198536-supplement-Supplementary_file__main_dataset_and_code__compressed_.zip › Covid-19-Teesside-main/Figures/GLMM/Lin51/Lin51-B1177_NB_AR1-Week-Postcode_No-week-no-temp-FE_Fit.png]

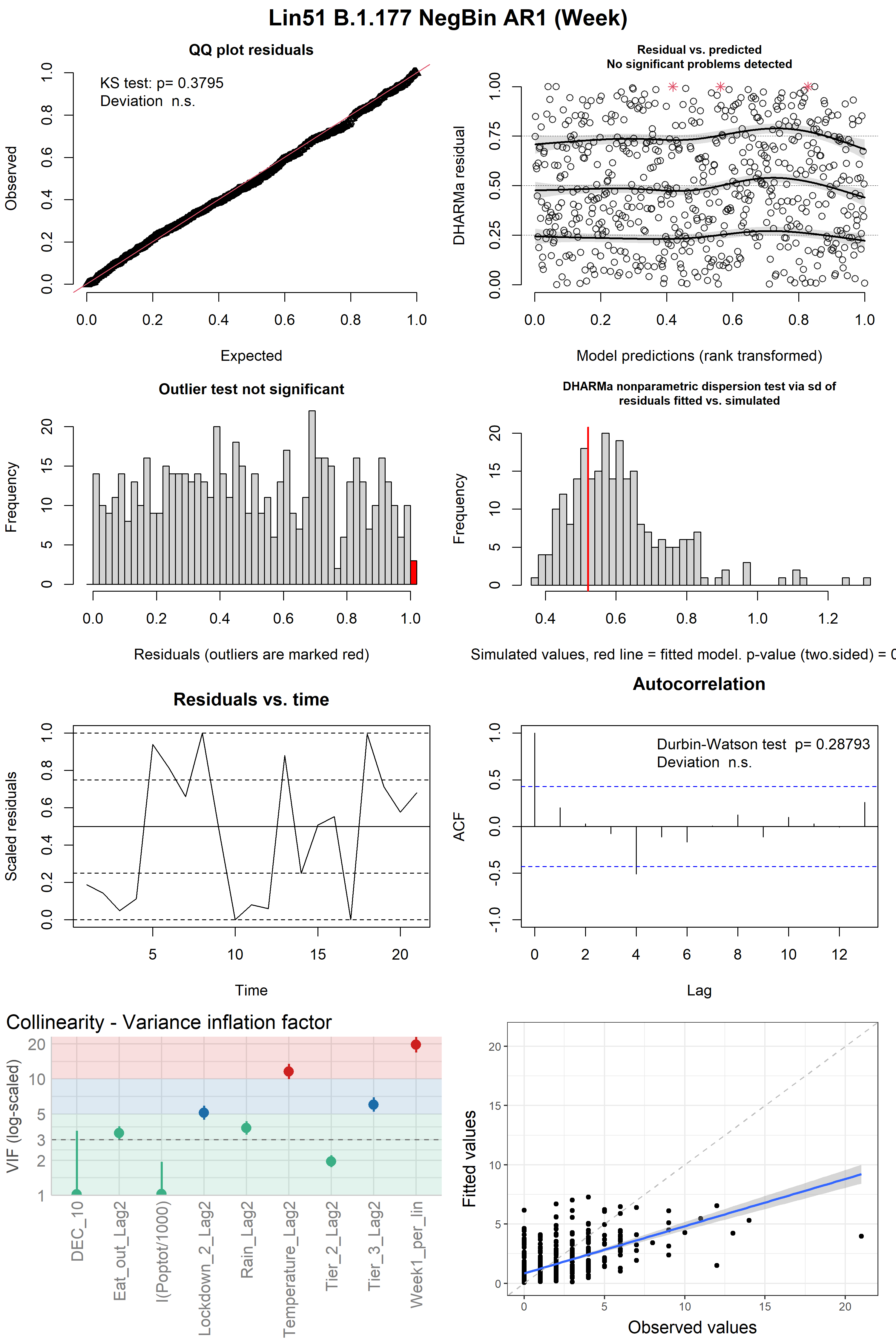

Supplement: Supplementary file: main dataset and code (compressed) [file EMS198536-supplement-Supplementary_file__main_dataset_and_code__compressed_.zip › Covid-19-Teesside-main/Figures/GLMM/Lin51/Lin51-B1177_NB_AR1-Week_Fit.png]

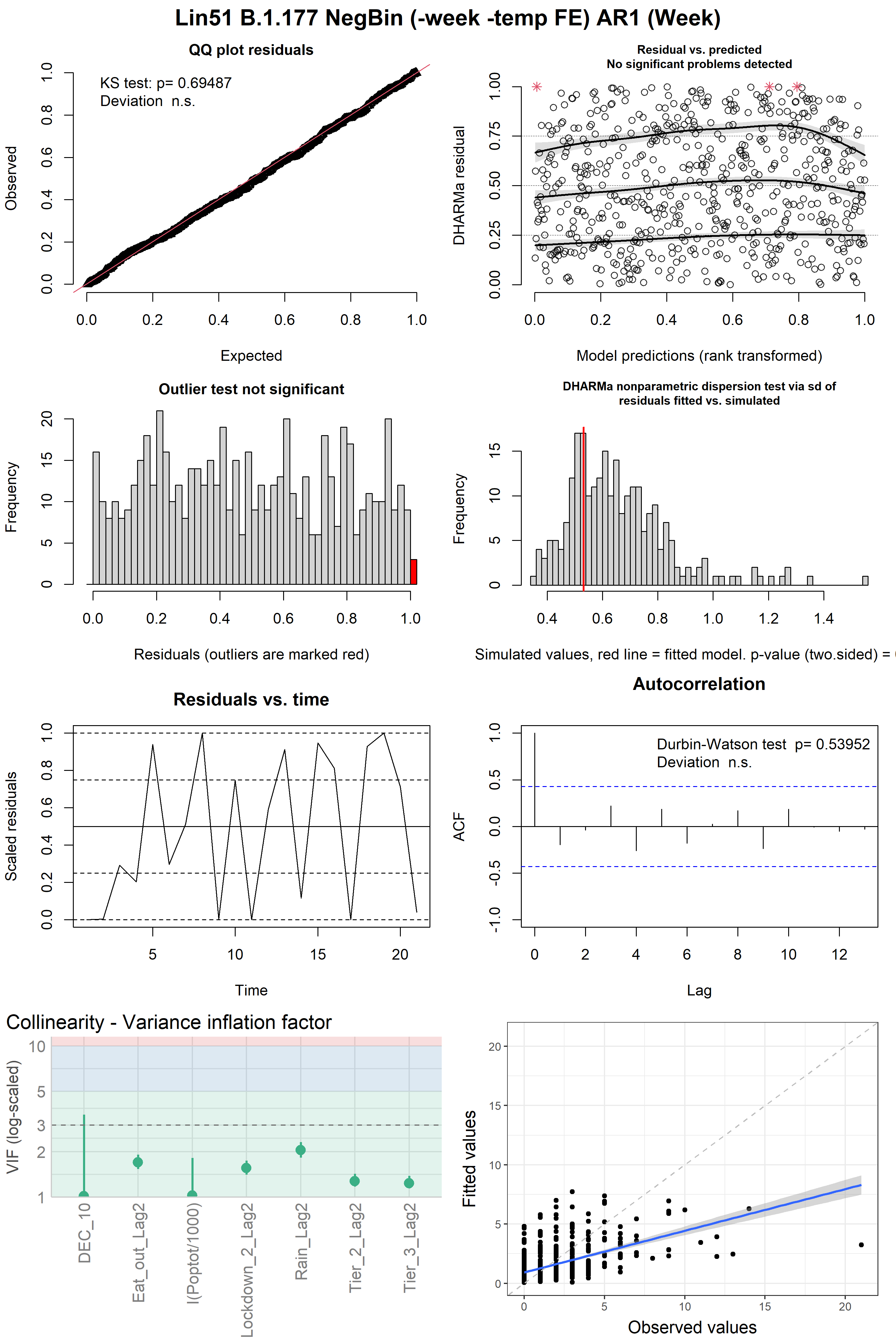

Supplement: Supplementary file: main dataset and code (compressed) [file EMS198536-supplement-Supplementary_file__main_dataset_and_code__compressed_.zip › Covid-19-Teesside-main/Figures/GLMM/Lin51/Lin51-B1177_NB_AR1-Week_No-week-no-temp-FE_Fit.png]

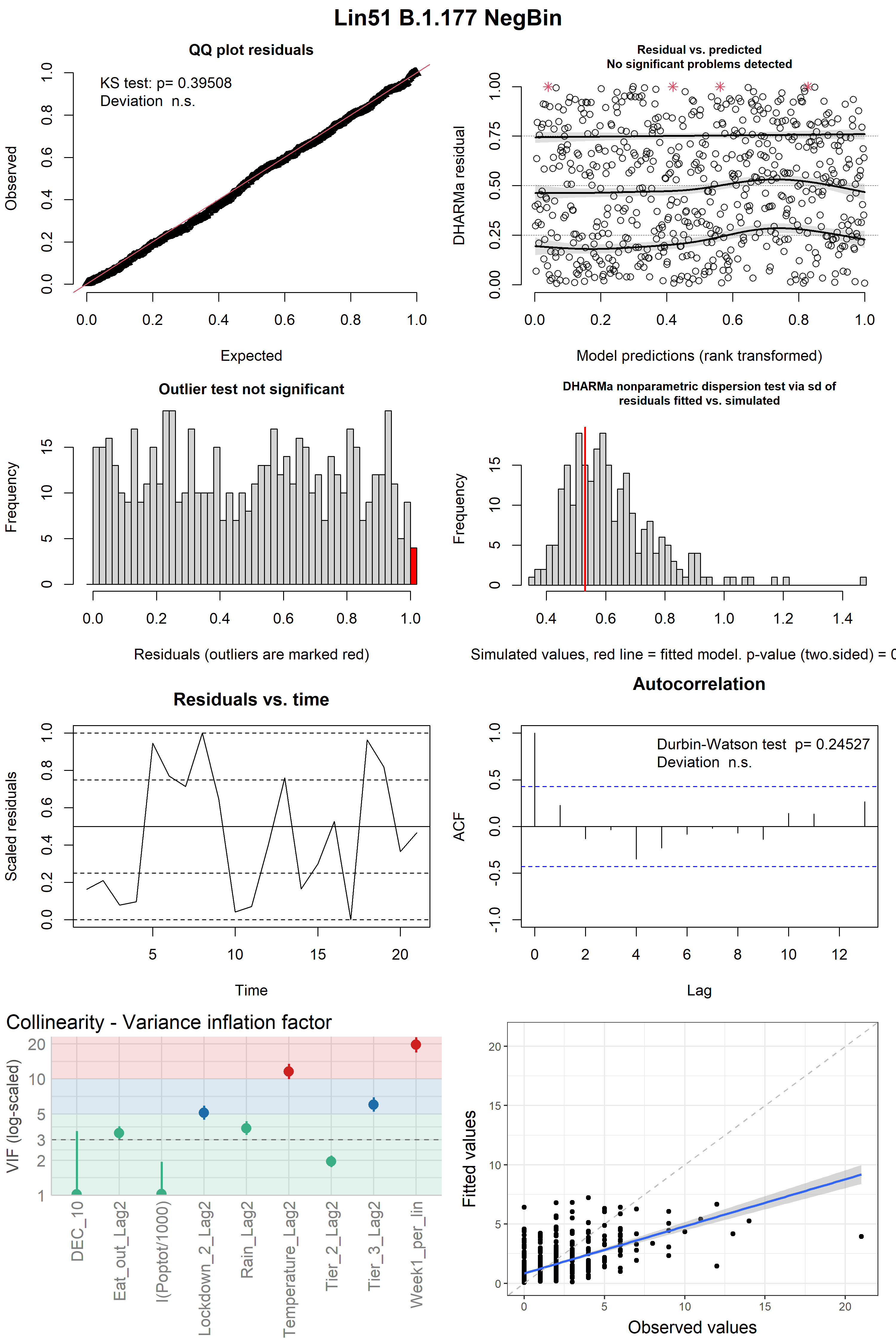

Supplement: Supplementary file: main dataset and code (compressed) [file EMS198536-supplement-Supplementary_file__main_dataset_and_code__compressed_.zip › Covid-19-Teesside-main/Figures/GLMM/Lin51/Lin51-B1177_NB_Full_Fit.png]

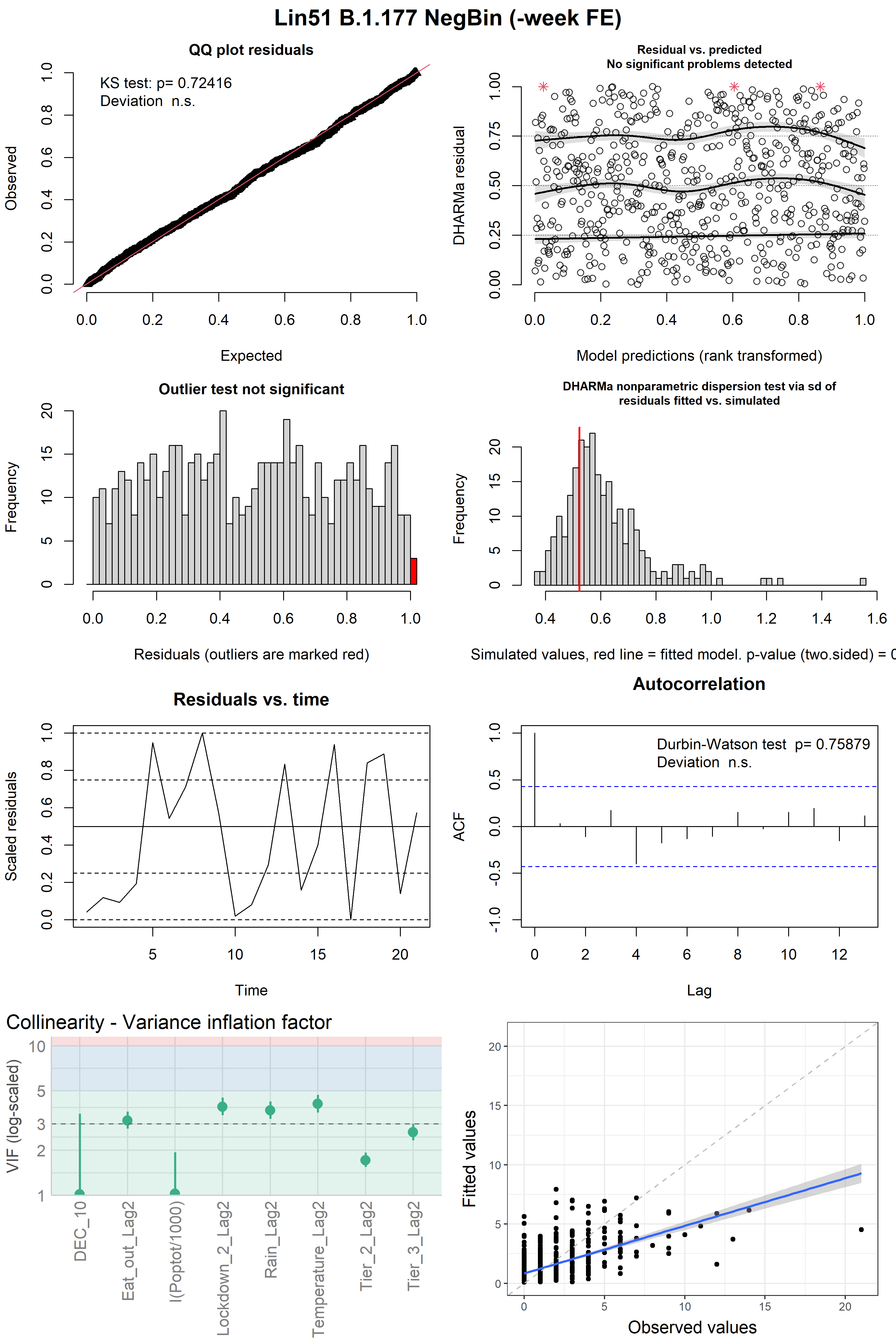

Supplement: Supplementary file: main dataset and code (compressed) [file EMS198536-supplement-Supplementary_file__main_dataset_and_code__compressed_.zip › Covid-19-Teesside-main/Figures/GLMM/Lin51/Lin51-B1177_NB_No-week-FE_Fit.png]

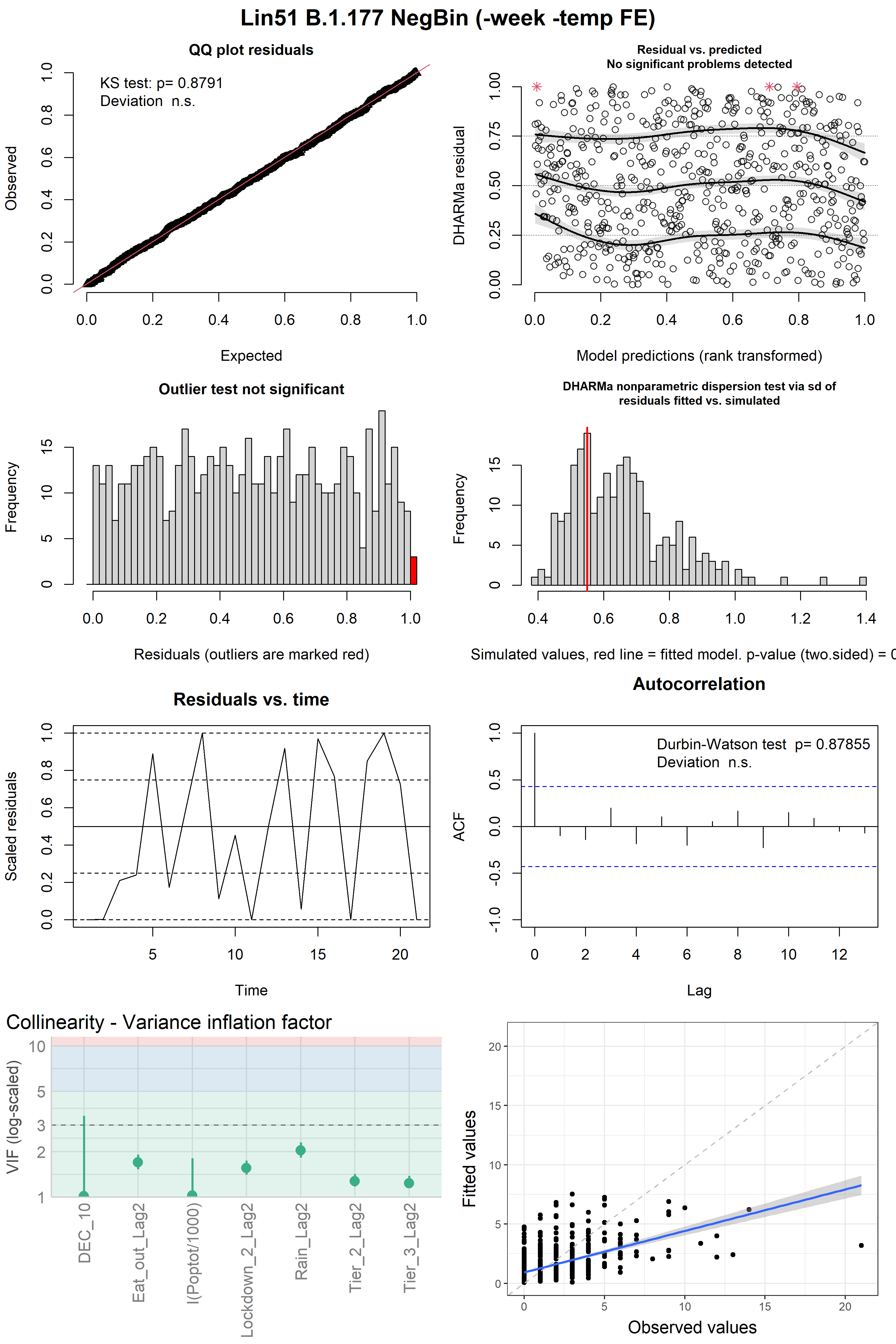

Supplement: Supplementary file: main dataset and code (compressed) [file EMS198536-supplement-Supplementary_file__main_dataset_and_code__compressed_.zip › Covid-19-Teesside-main/Figures/GLMM/Lin51/Lin51-B1177_NB_No-week-no-temp-FE_Fit.png]

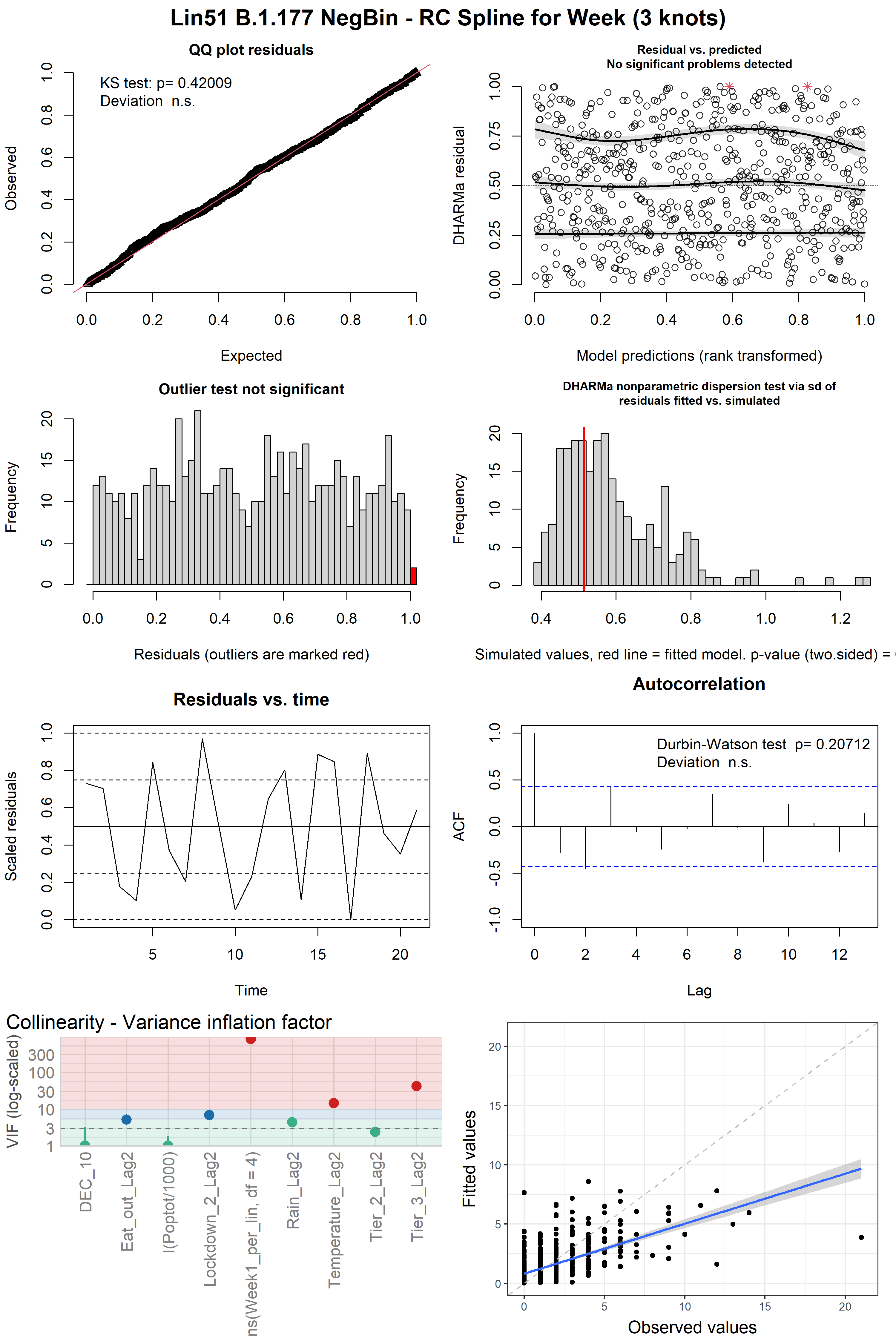

Supplement: Supplementary file: main dataset and code (compressed) [file EMS198536-supplement-Supplementary_file__main_dataset_and_code__compressed_.zip › Covid-19-Teesside-main/Figures/GLMM/Lin51/Lin51-B1177_NB_RCS-Week-3knots_Fit.png]

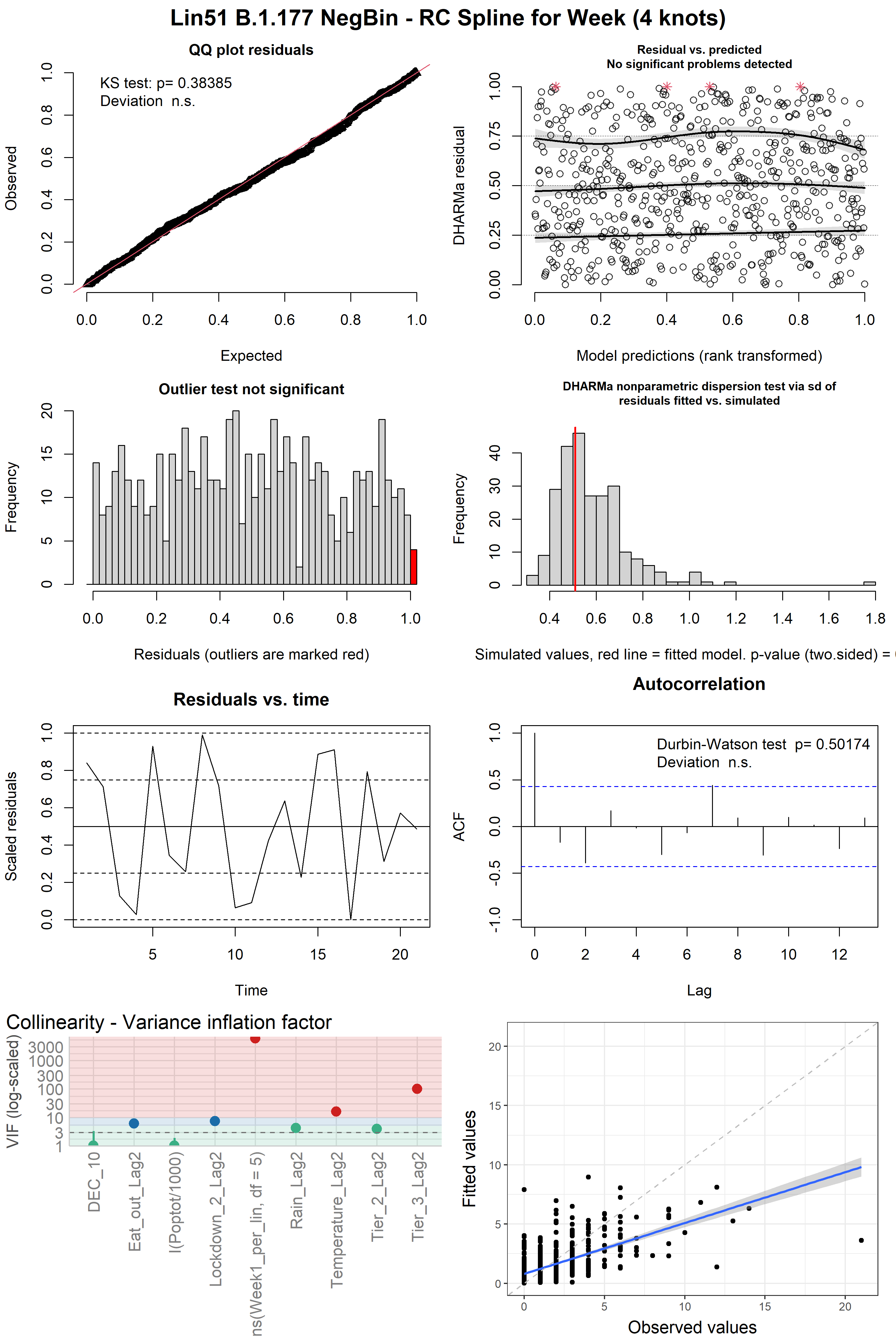

Supplement: Supplementary file: main dataset and code (compressed) [file EMS198536-supplement-Supplementary_file__main_dataset_and_code__compressed_.zip › Covid-19-Teesside-main/Figures/GLMM/Lin51/Lin51-B1177_NB_RCS-Week-4knots_Fit.png]

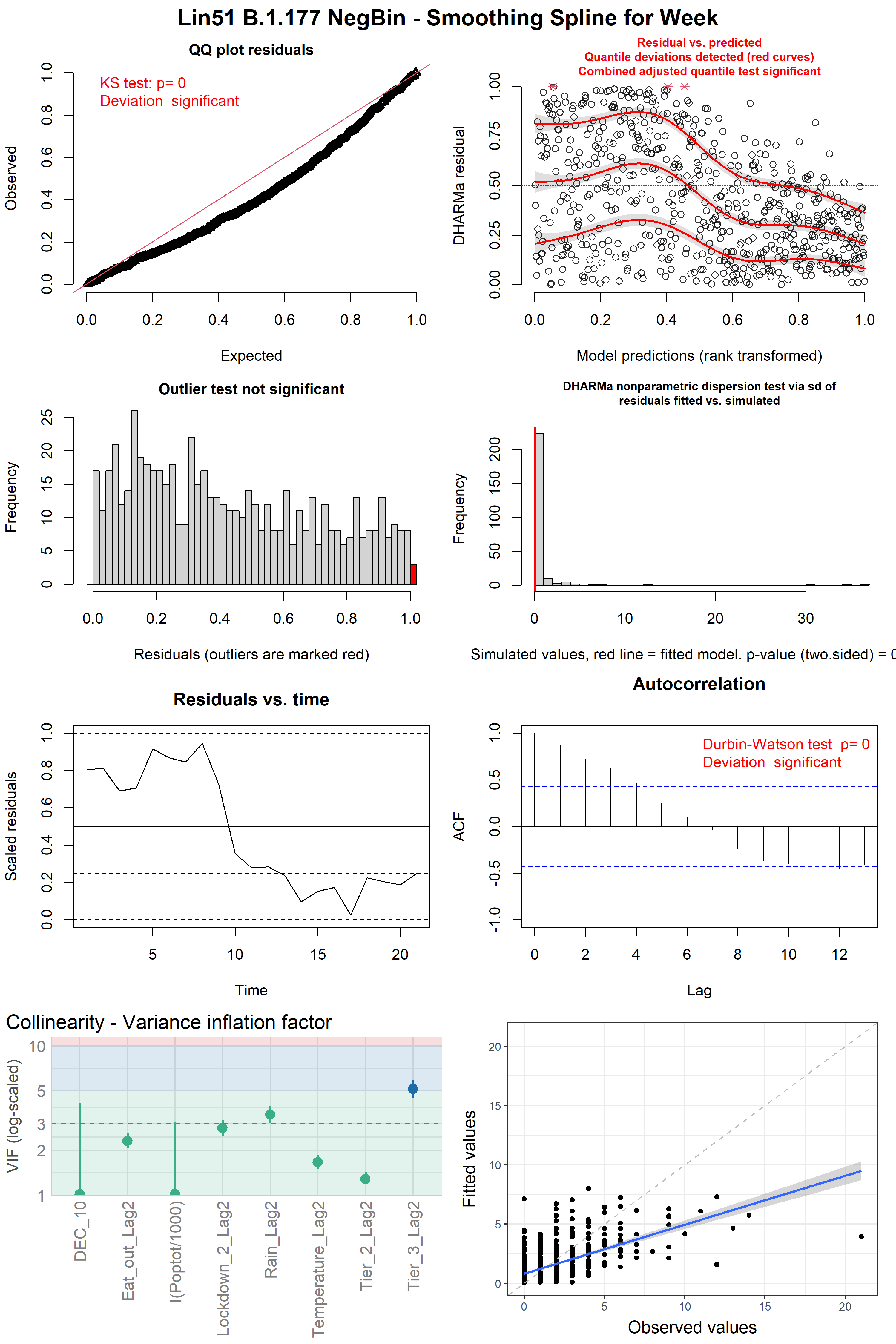

Supplement: Supplementary file: main dataset and code (compressed) [file EMS198536-supplement-Supplementary_file__main_dataset_and_code__compressed_.zip › Covid-19-Teesside-main/Figures/GLMM/Lin51/Lin51-B1177_NB_SmoothSpline-Week-TPS_Fit.png]

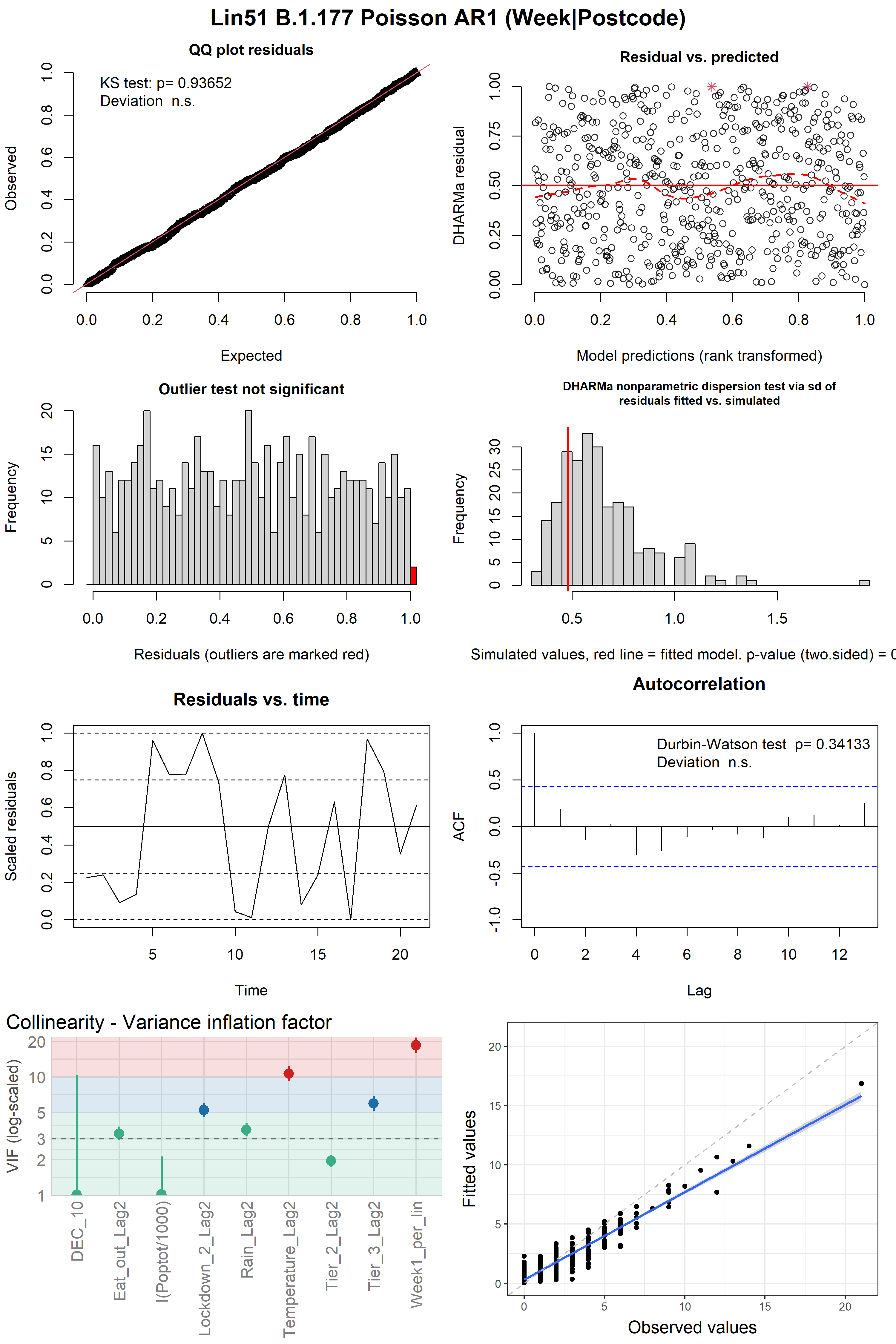

Supplement: Supplementary file: main dataset and code (compressed) [file EMS198536-supplement-Supplementary_file__main_dataset_and_code__compressed_.zip › Covid-19-Teesside-main/Figures/GLMM/Lin51/Lin51-B1177_Po_AR1-Week-Postcode_Fit.png]

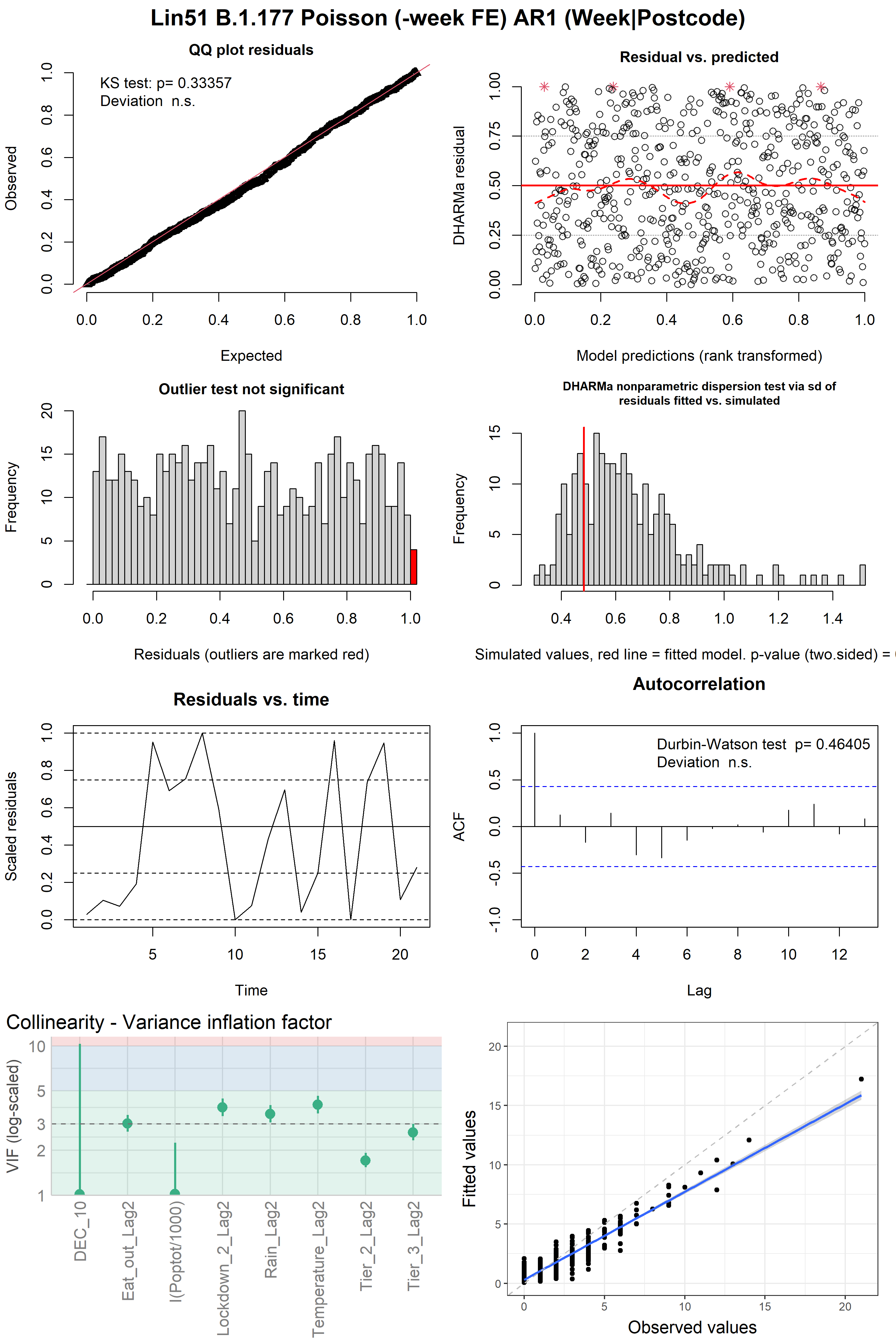

Supplement: Supplementary file: main dataset and code (compressed) [file EMS198536-supplement-Supplementary_file__main_dataset_and_code__compressed_.zip › Covid-19-Teesside-main/Figures/GLMM/Lin51/Lin51-B1177_Po_AR1-Week-Postcode_No-week-FE_Fit.png]

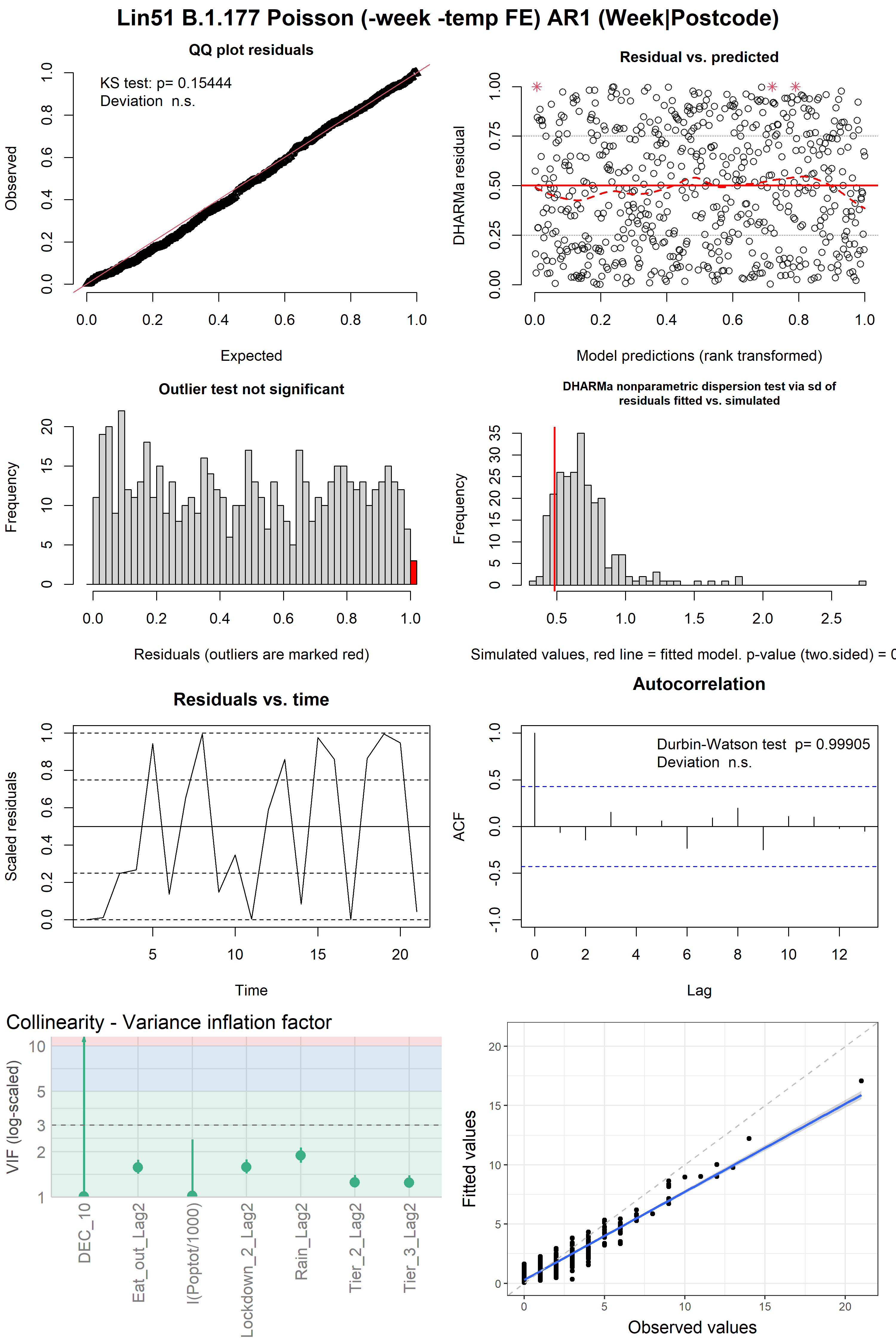

Supplement: Supplementary file: main dataset and code (compressed) [file EMS198536-supplement-Supplementary_file__main_dataset_and_code__compressed_.zip › Covid-19-Teesside-main/Figures/GLMM/Lin51/Lin51-B1177_Po_AR1-Week-Postcode_No-week-no-temp-FE_Fit.png]

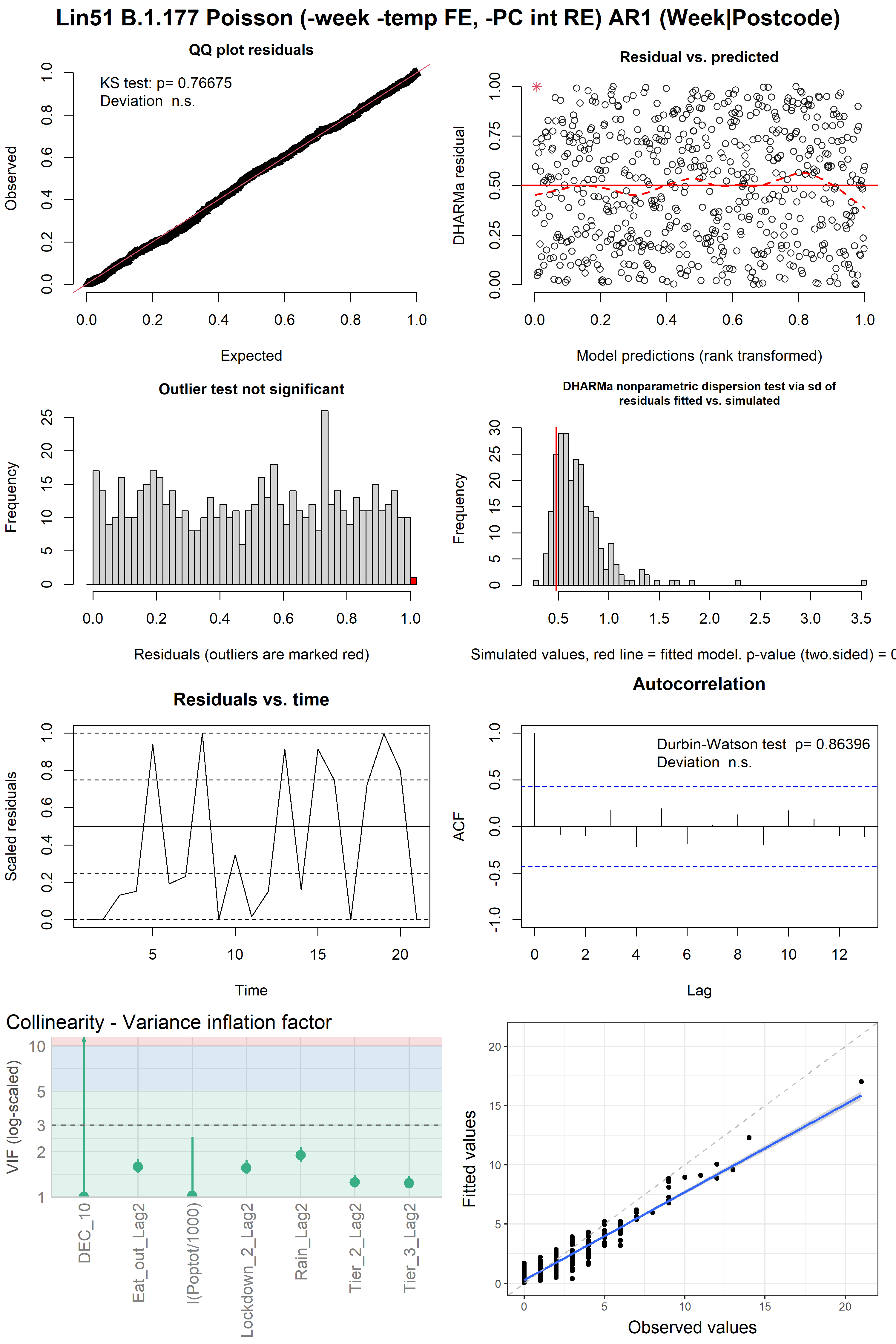

Supplement: Supplementary file: main dataset and code (compressed) [file EMS198536-supplement-Supplementary_file__main_dataset_and_code__compressed_.zip › Covid-19-Teesside-main/Figures/GLMM/Lin51/Lin51-B1177_Po_AR1-Week-Postcode_No-week-no-temp-FE_No-PC-int-RE_Fit.png]

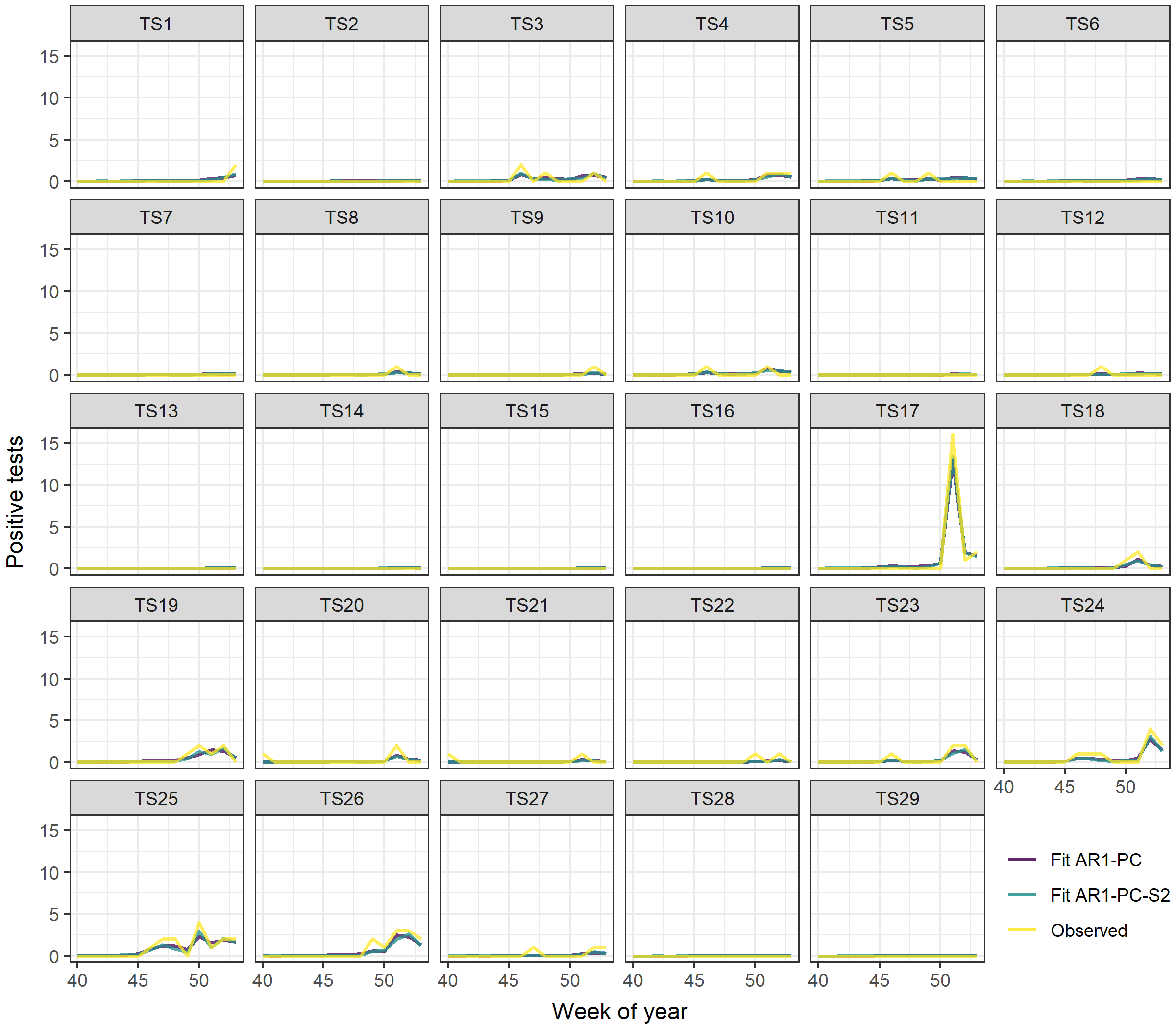

Supplement: Supplementary file: main dataset and code (compressed) [file EMS198536-supplement-Supplementary_file__main_dataset_and_code__compressed_.zip › Covid-19-Teesside-main/Figures/GLMM/Lin52/Lin52-B117710_GLMM_Obs-vs-Fit_AR1PC-AR1PCS2.png]

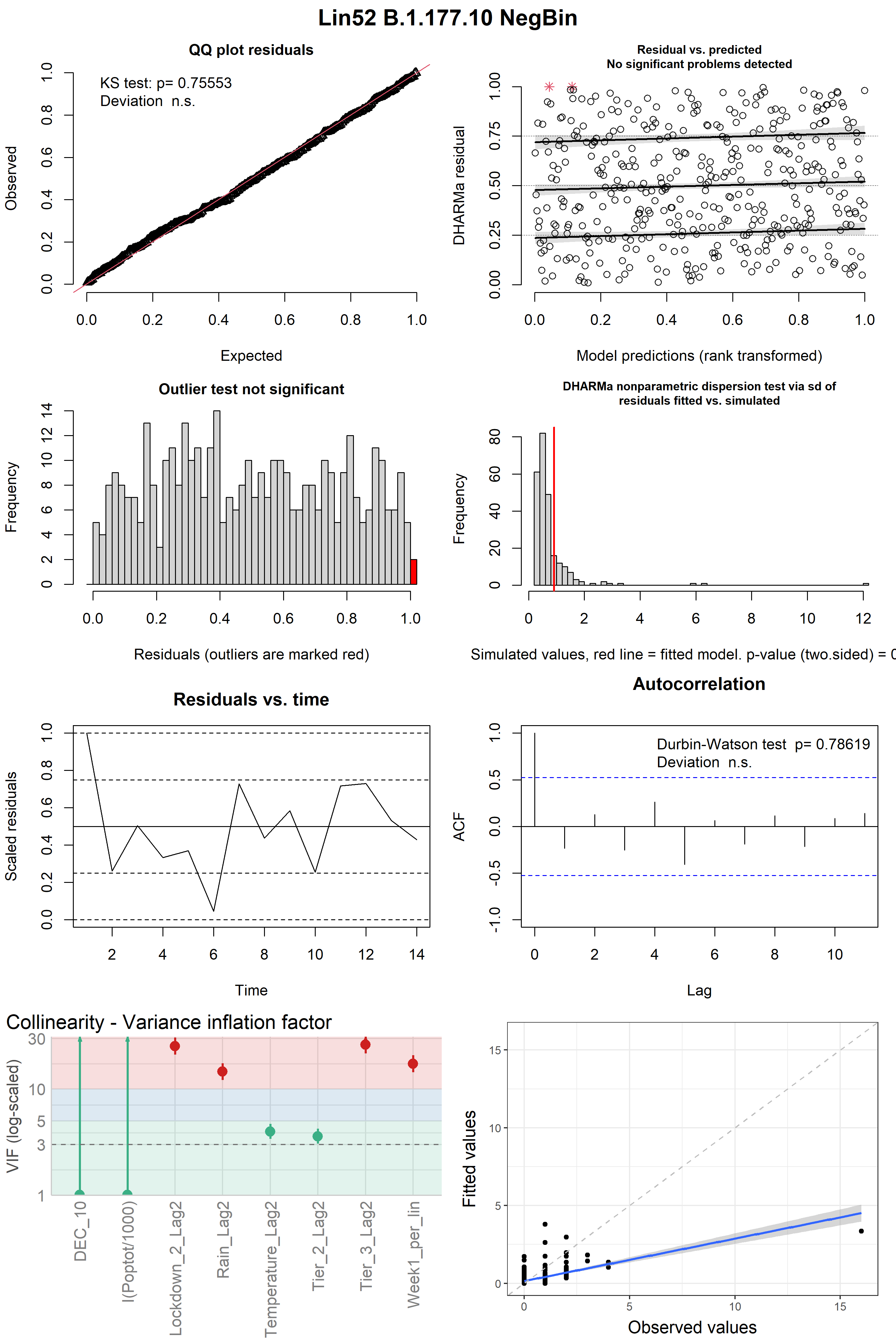

Supplement: Supplementary file: main dataset and code (compressed) [file EMS198536-supplement-Supplementary_file__main_dataset_and_code__compressed_.zip › Covid-19-Teesside-main/Figures/GLMM/Lin52/Lin52-B117710_NB_Full_Fit2.png]

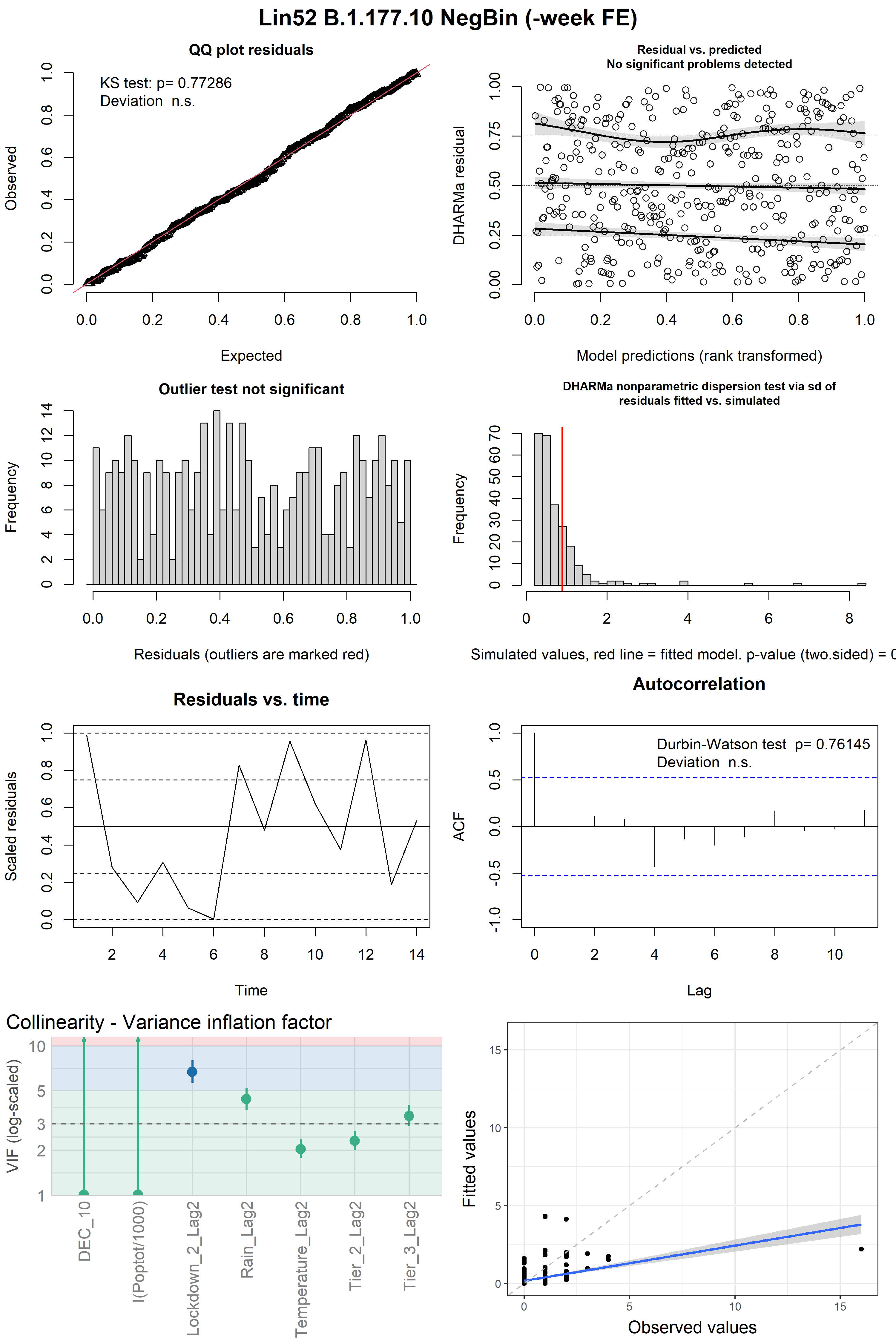

Supplement: Supplementary file: main dataset and code (compressed) [file EMS198536-supplement-Supplementary_file__main_dataset_and_code__compressed_.zip › Covid-19-Teesside-main/Figures/GLMM/Lin52/Lin52-B117710_NB_No-week-FE_Fit.png]

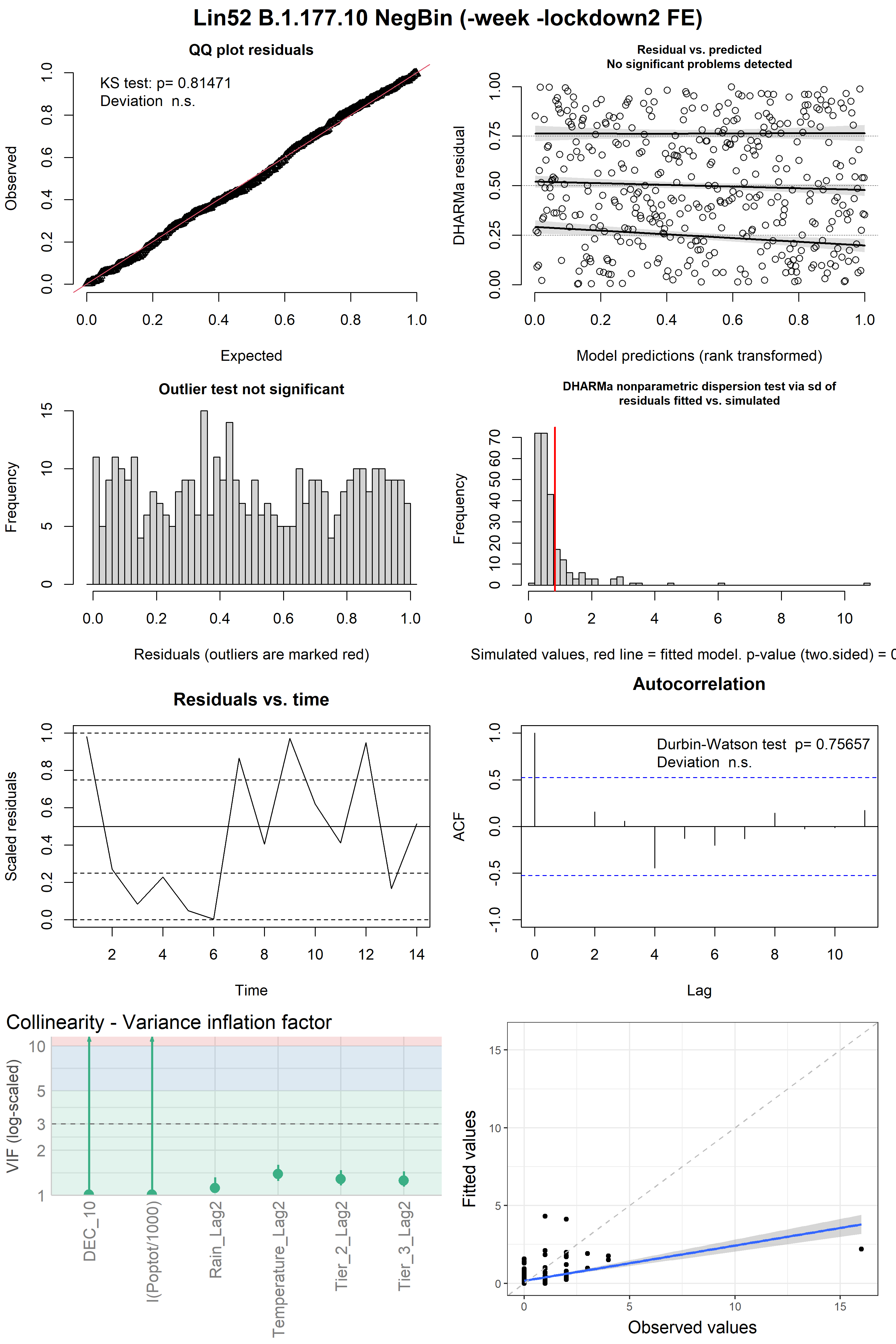

Supplement: Supplementary file: main dataset and code (compressed) [file EMS198536-supplement-Supplementary_file__main_dataset_and_code__compressed_.zip › Covid-19-Teesside-main/Figures/GLMM/Lin52/Lin52-B117710_NB_No-week-no-lockdown2-FE_Fit.png]

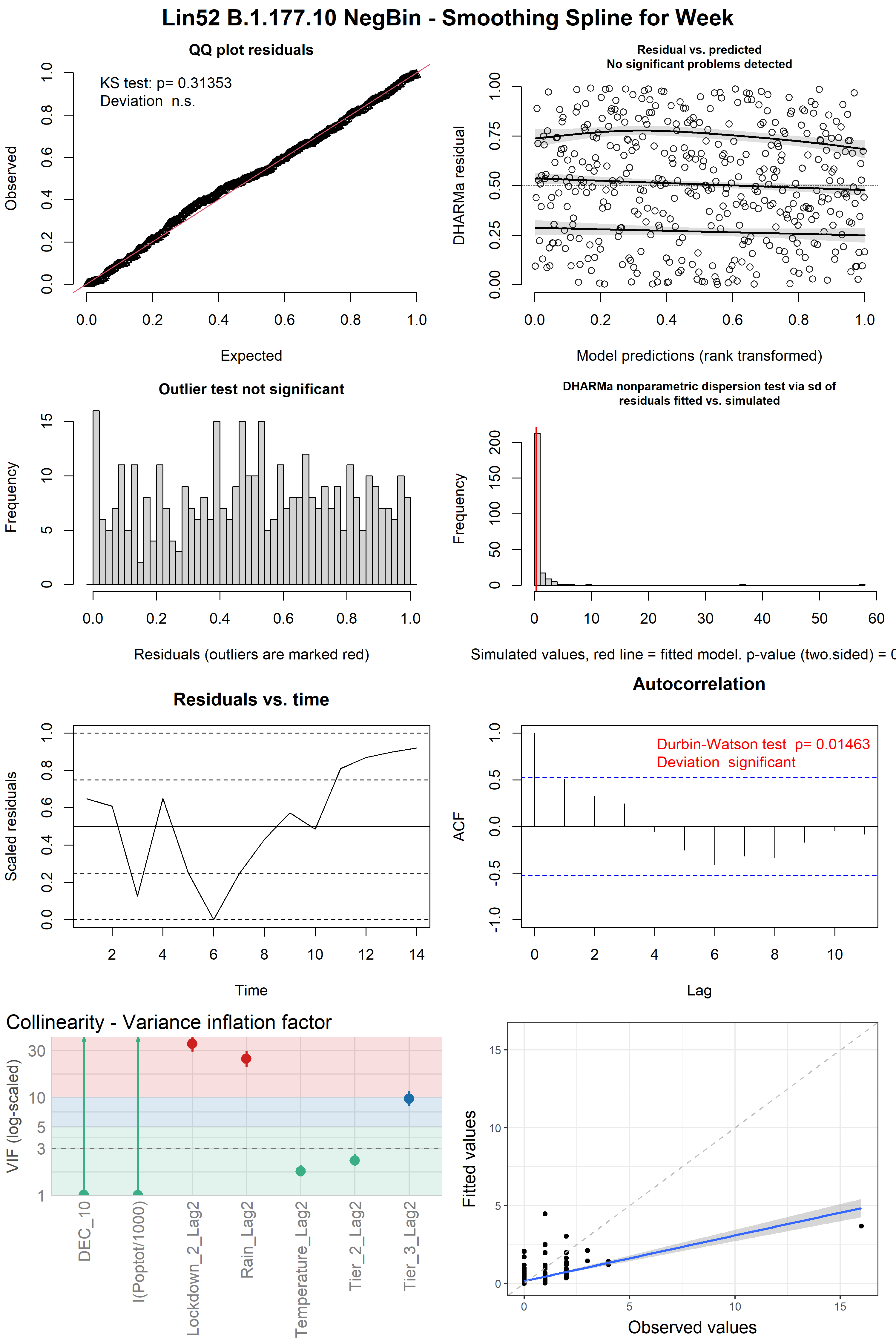

Supplement: Supplementary file: main dataset and code (compressed) [file EMS198536-supplement-Supplementary_file__main_dataset_and_code__compressed_.zip › Covid-19-Teesside-main/Figures/GLMM/Lin52/Lin52-B117710_NB_SmoothSpline-Week-TPS_Fit.png]

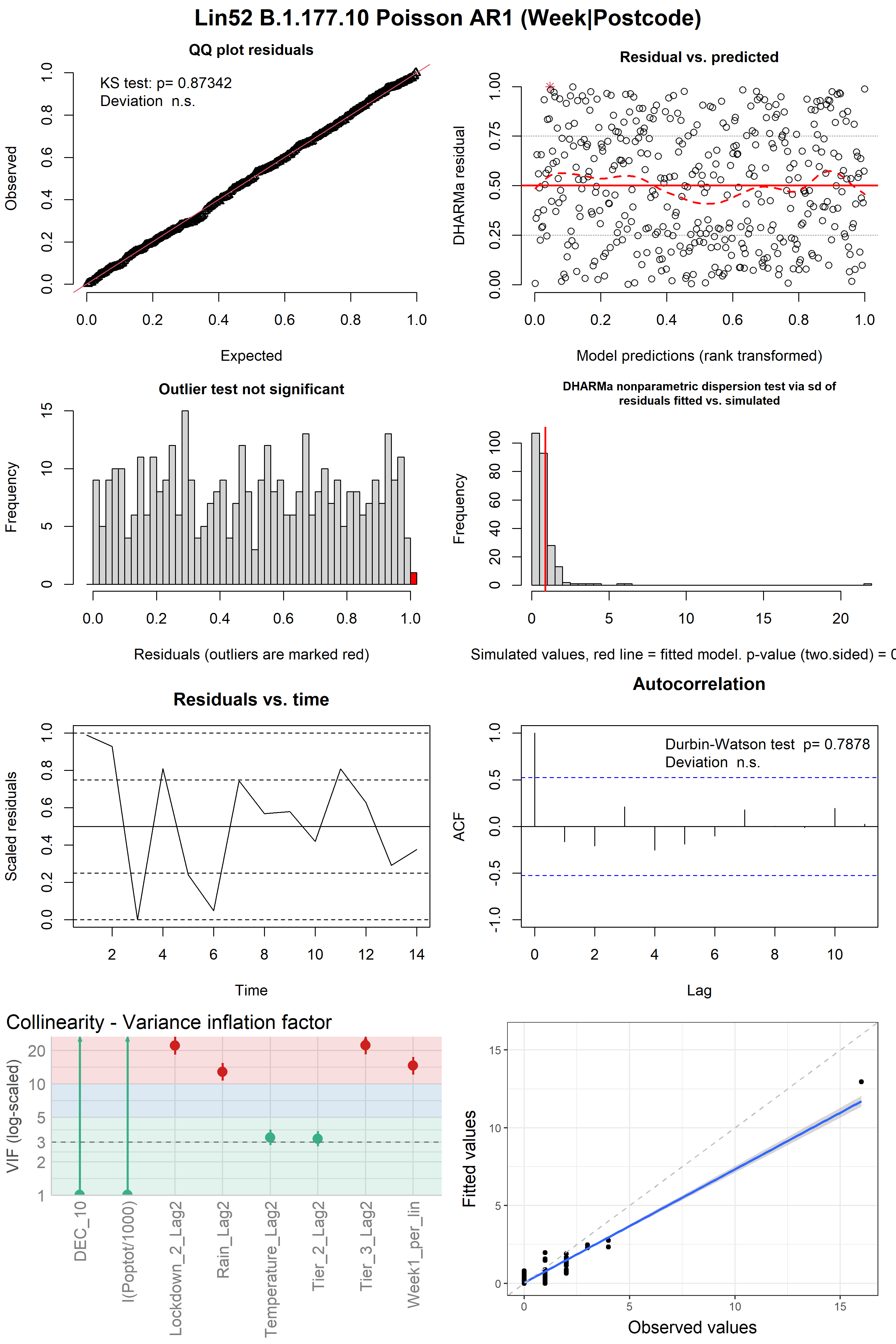

Supplement: Supplementary file: main dataset and code (compressed) [file EMS198536-supplement-Supplementary_file__main_dataset_and_code__compressed_.zip › Covid-19-Teesside-main/Figures/GLMM/Lin52/Lin52-B117710_Po_AR1-Week-Postcode_Fit.png]

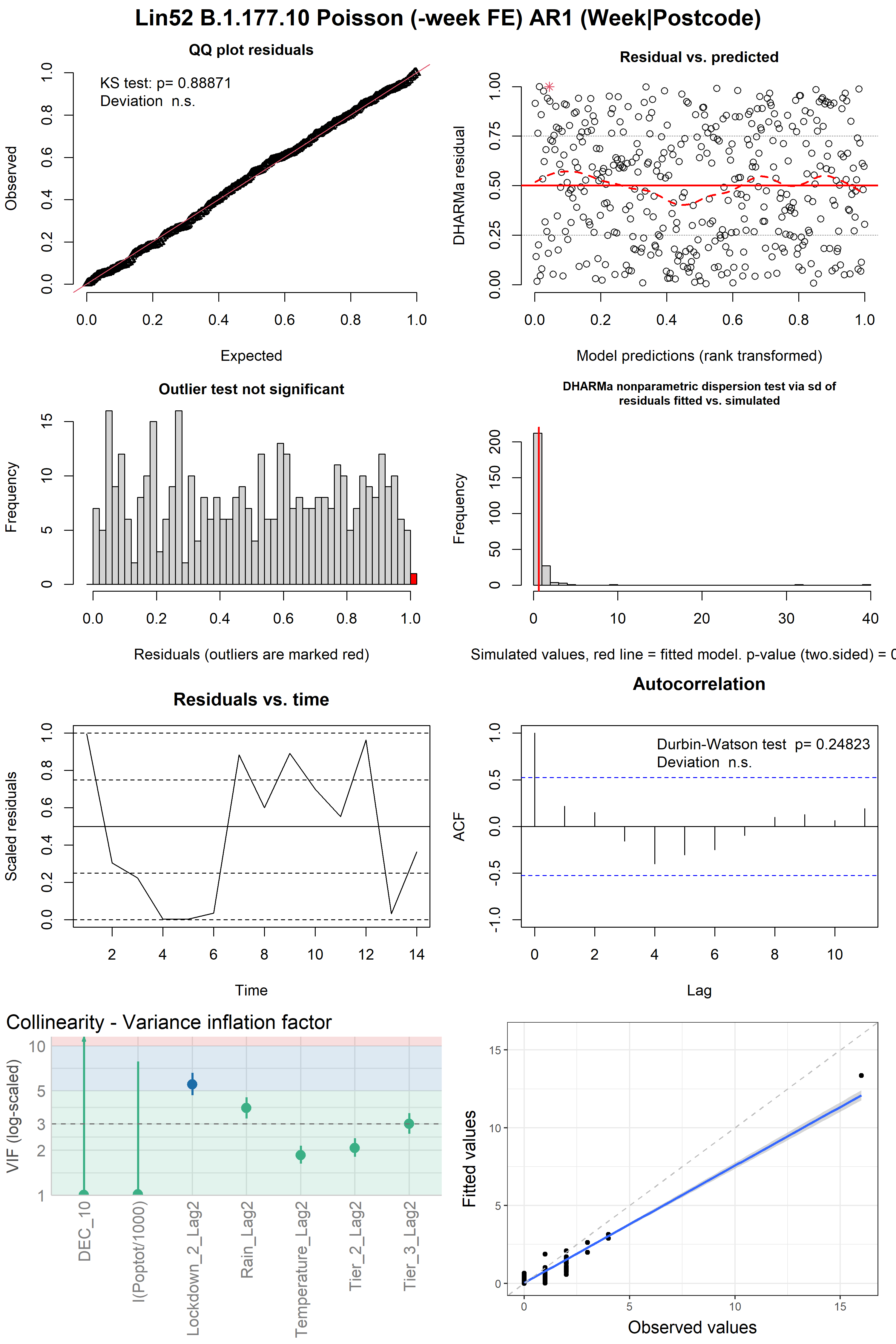

Supplement: Supplementary file: main dataset and code (compressed) [file EMS198536-supplement-Supplementary_file__main_dataset_and_code__compressed_.zip › Covid-19-Teesside-main/Figures/GLMM/Lin52/Lin52-B117710_Po_AR1-Week-Postcode_No-week-FE_Fit.png]

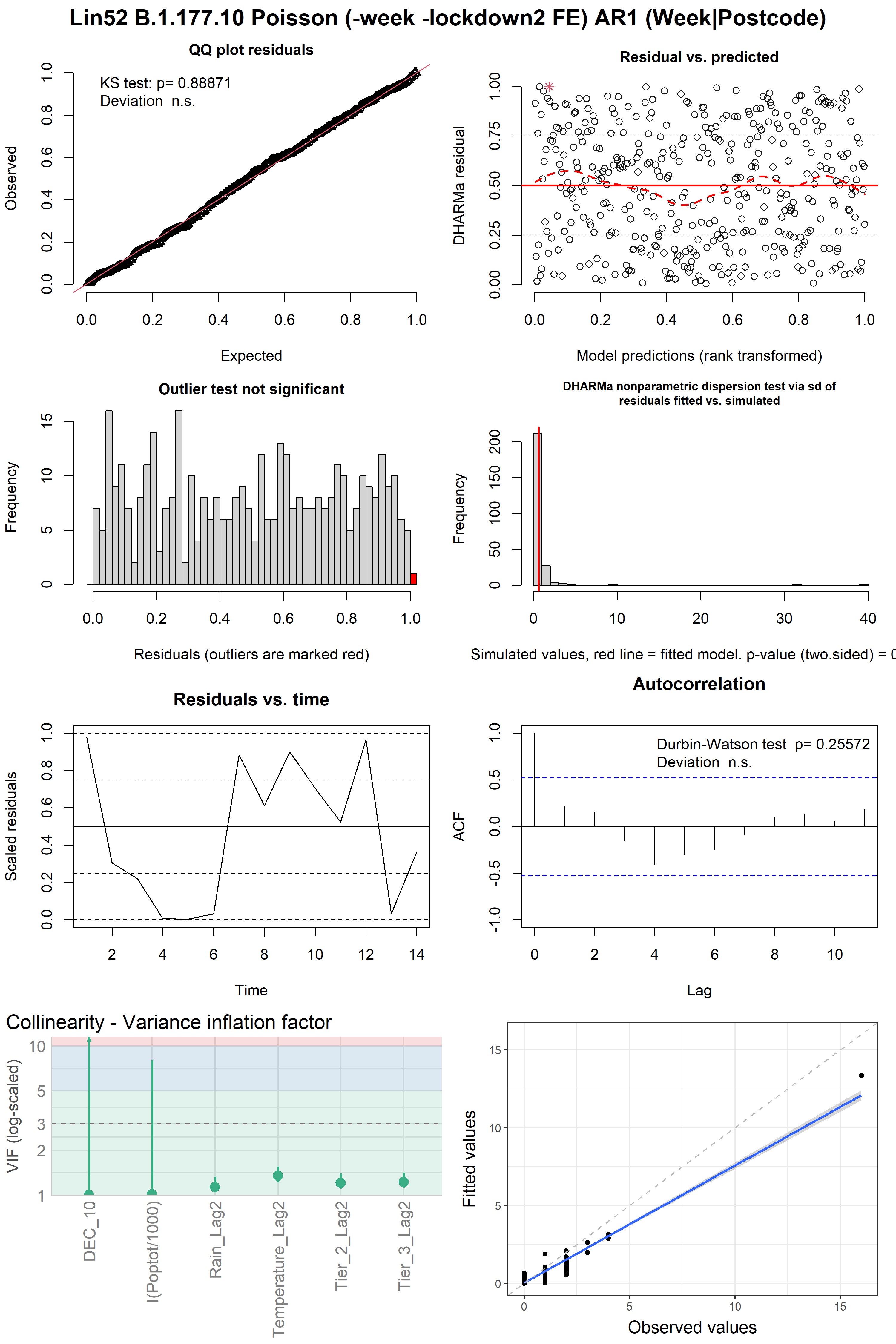

Supplement: Supplementary file: main dataset and code (compressed) [file EMS198536-supplement-Supplementary_file__main_dataset_and_code__compressed_.zip › Covid-19-Teesside-main/Figures/GLMM/Lin52/Lin52-B117710_Po_AR1-Week-Postcode_No-week-no-lockdown2-FE_Fit.png]

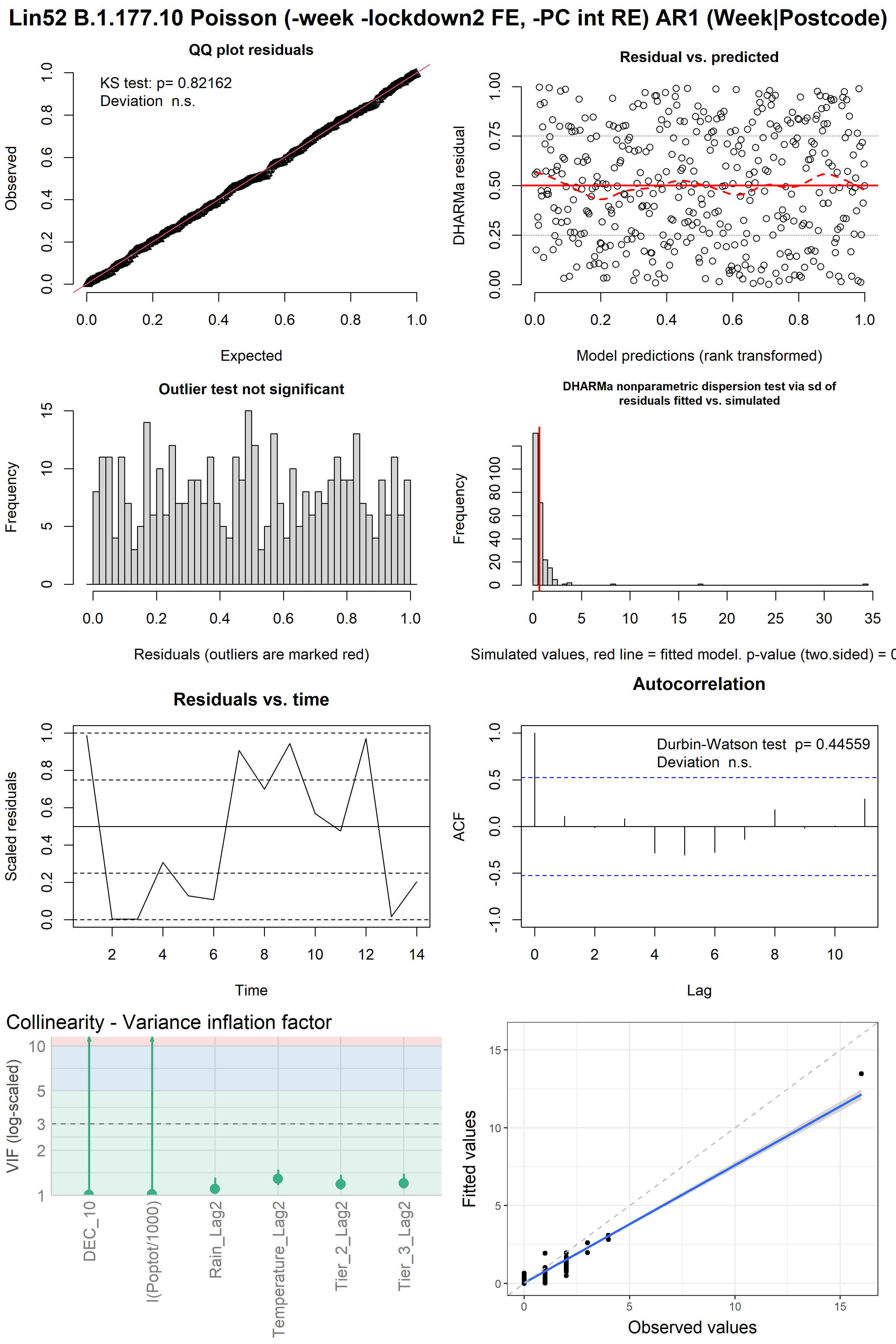

Supplement: Supplementary file: main dataset and code (compressed) [file EMS198536-supplement-Supplementary_file__main_dataset_and_code__compressed_.zip › Covid-19-Teesside-main/Figures/GLMM/Lin52/Lin52-B117710_Po_AR1-Week-Postcode_No-week-no-lockdown2-FE_No-PC-int-RE_Fit.png]

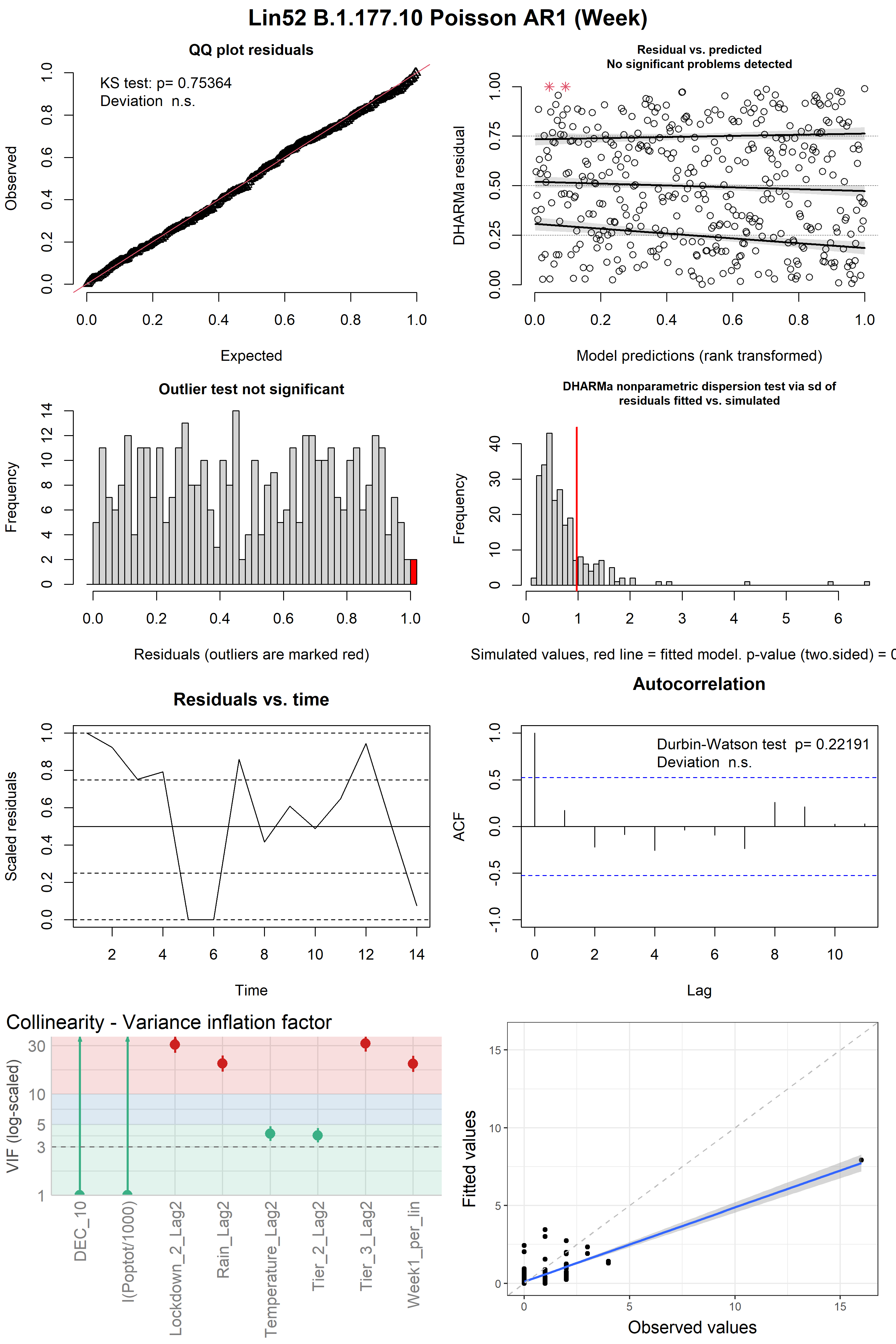

Supplement: Supplementary file: main dataset and code (compressed) [file EMS198536-supplement-Supplementary_file__main_dataset_and_code__compressed_.zip › Covid-19-Teesside-main/Figures/GLMM/Lin52/Lin52-B117710_Po_AR1-Week_Fit.png]

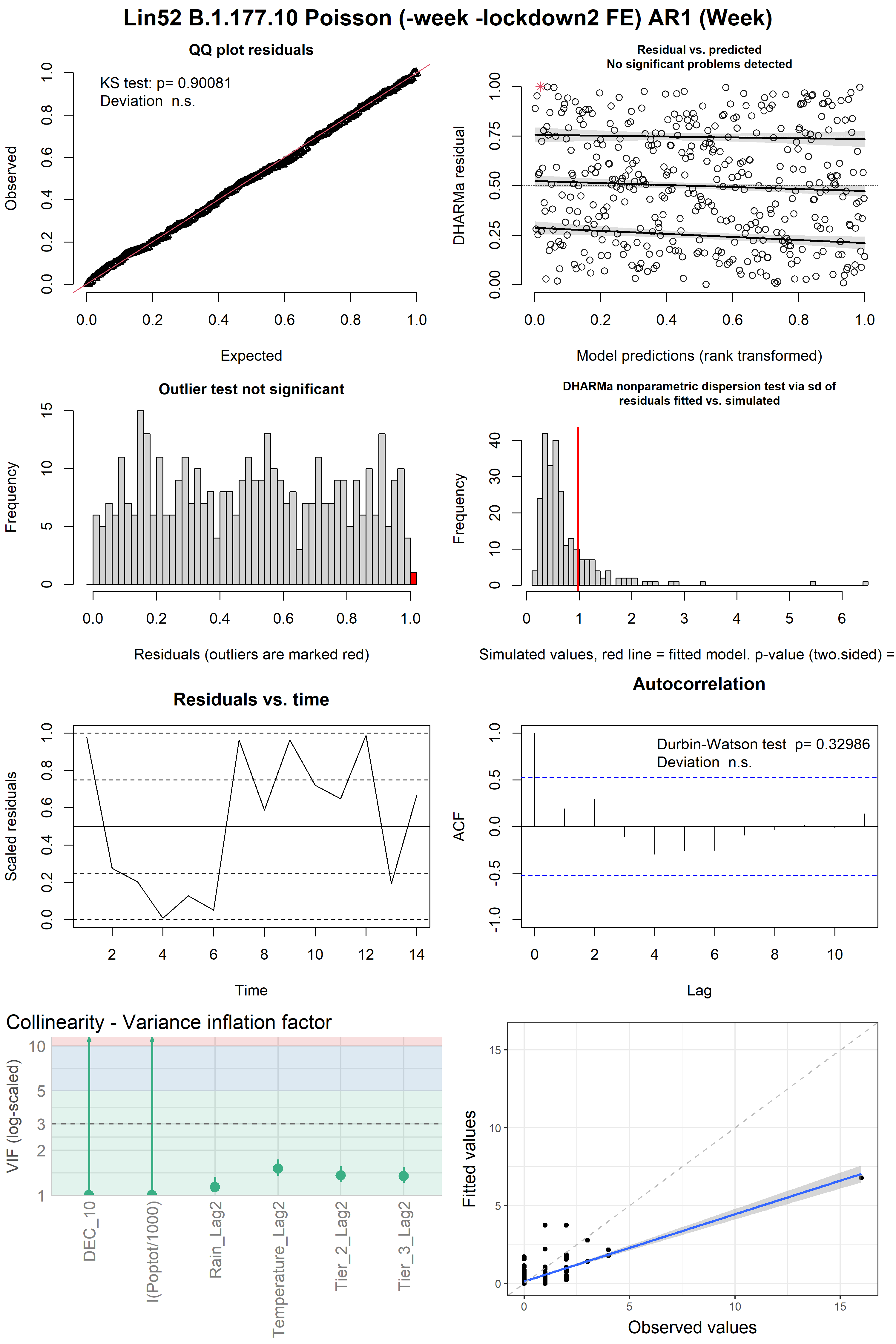

Supplement: Supplementary file: main dataset and code (compressed) [file EMS198536-supplement-Supplementary_file__main_dataset_and_code__compressed_.zip › Covid-19-Teesside-main/Figures/GLMM/Lin52/Lin52-B117710_Po_AR1-Week_No-week-no-lockdown2-FE_Fit.png]

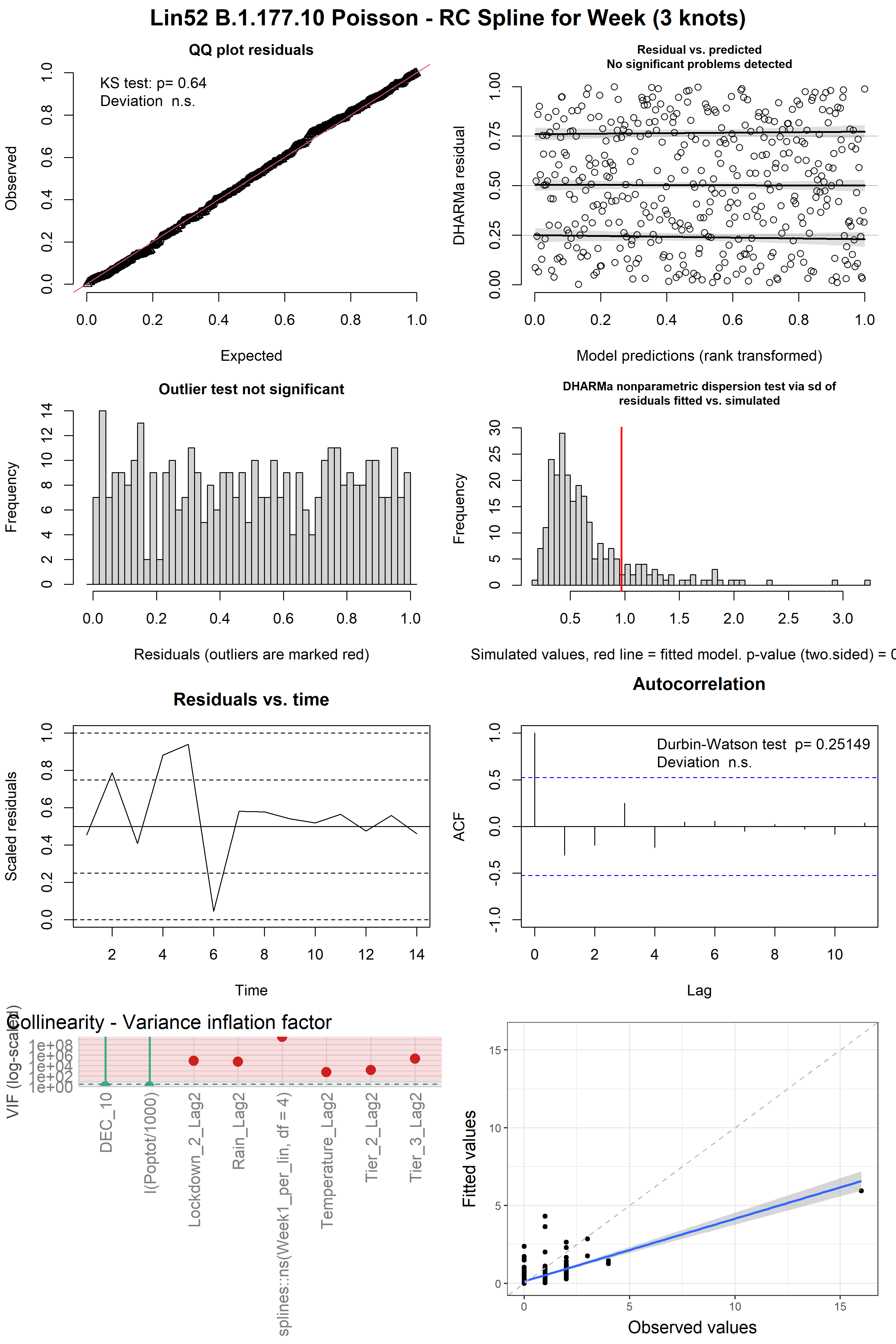

Supplement: Supplementary file: main dataset and code (compressed) [file EMS198536-supplement-Supplementary_file__main_dataset_and_code__compressed_.zip › Covid-19-Teesside-main/Figures/GLMM/Lin52/Lin52-B117710_Po_RCS-Week-3knots_Fit.png]

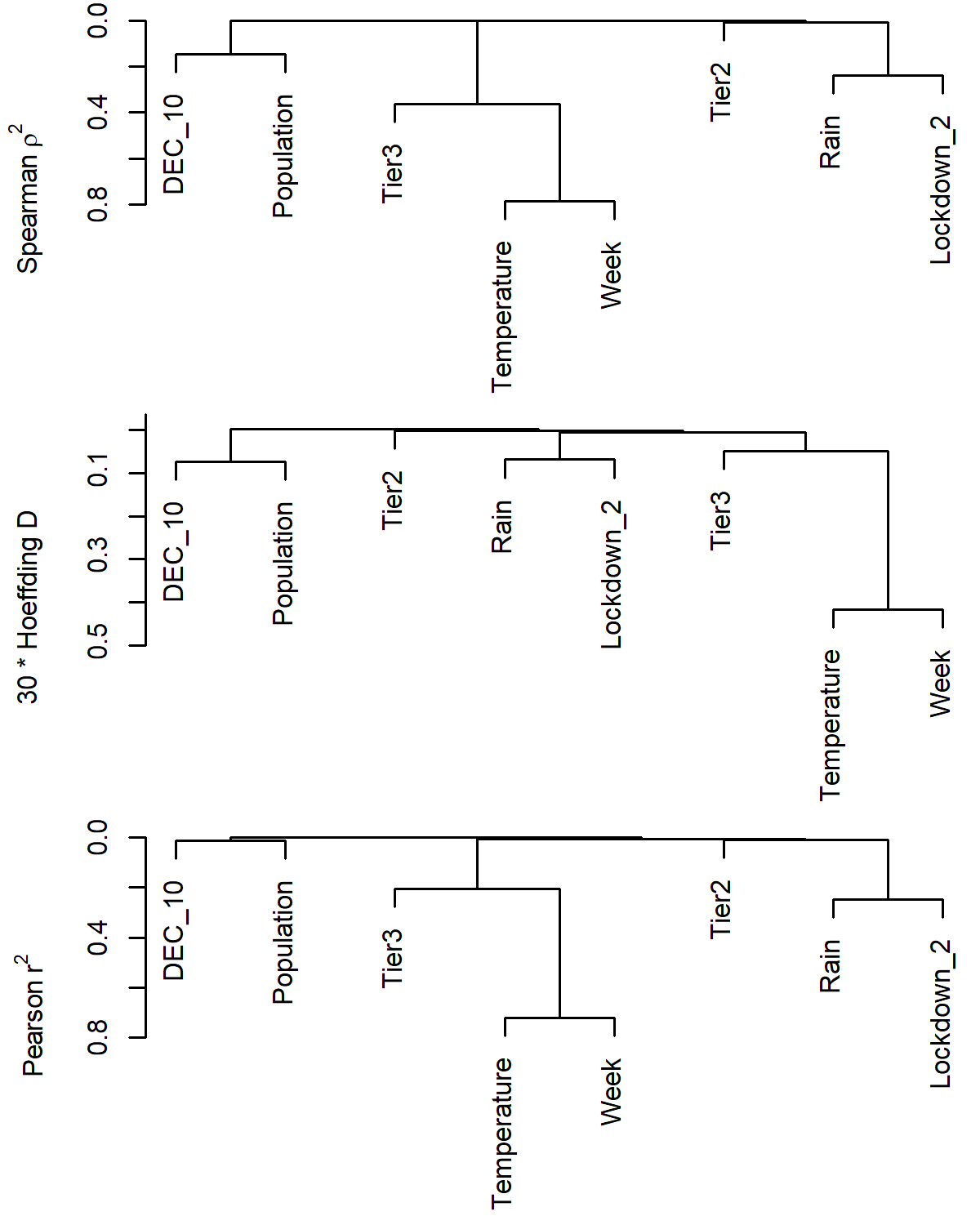

Supplement: Supplementary file: main dataset and code (compressed) [file EMS198536-supplement-Supplementary_file__main_dataset_and_code__compressed_.zip › Covid-19-Teesside-main/Figures/GLMM/Lin52/Lin52-B117710_Variable-Clustering.png]

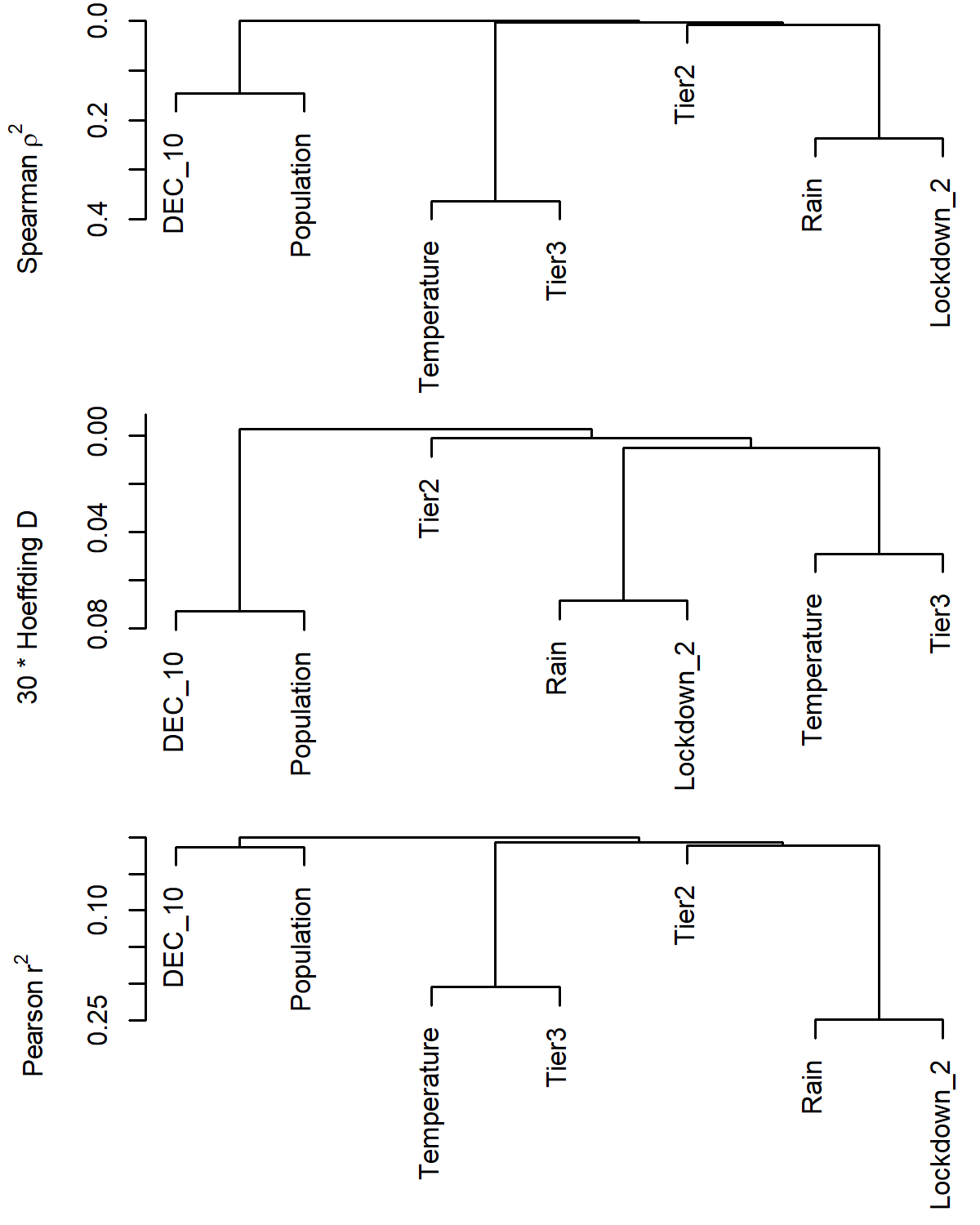

Supplement: Supplementary file: main dataset and code (compressed) [file EMS198536-supplement-Supplementary_file__main_dataset_and_code__compressed_.zip › Covid-19-Teesside-main/Figures/GLMM/Lin52/Lin52-B117710_Variable-Clustering_Without-Week.png]

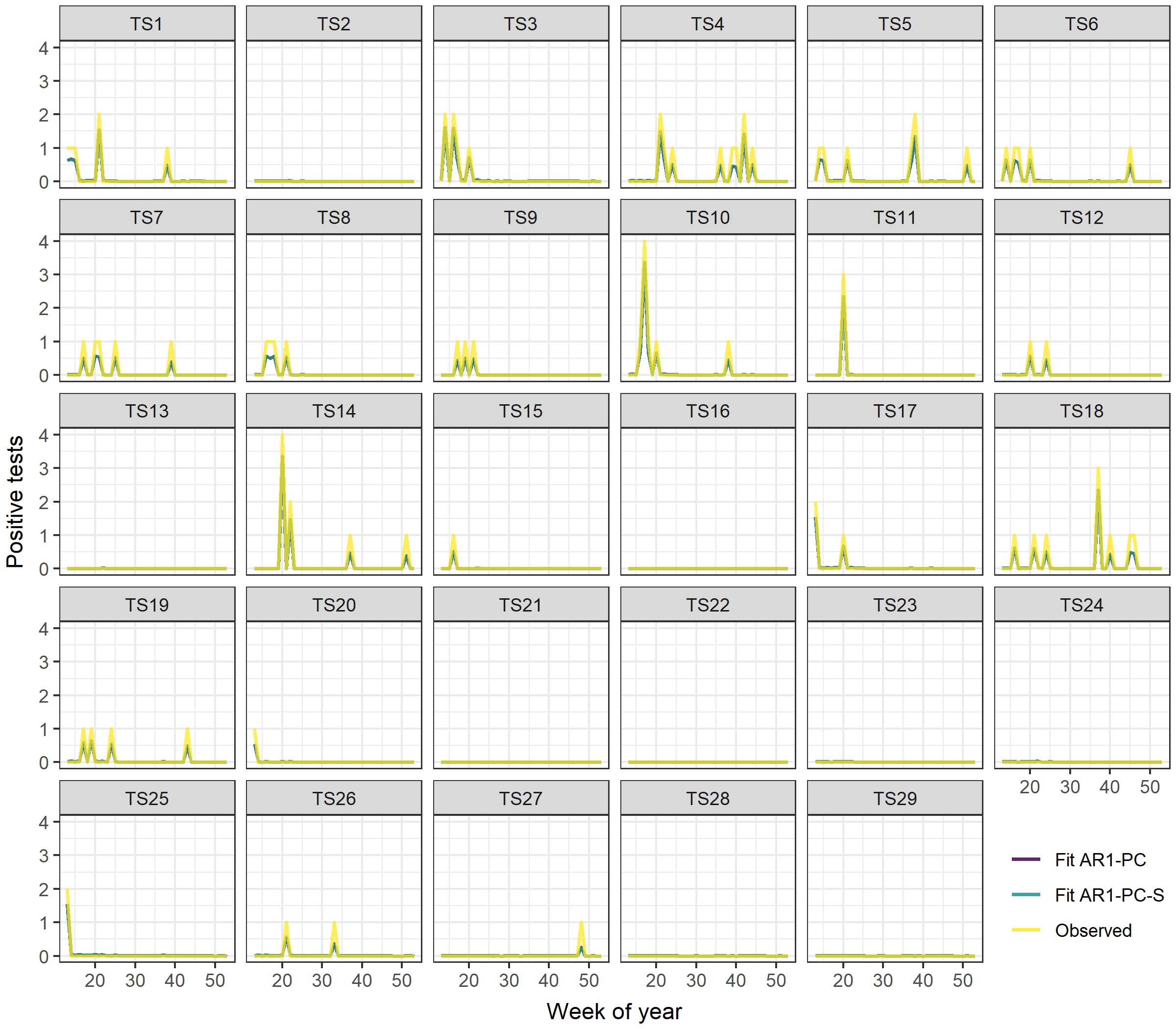

Supplement: Supplementary file: main dataset and code (compressed) [file EMS198536-supplement-Supplementary_file__main_dataset_and_code__compressed_.zip › Covid-19-Teesside-main/Figures/GLMM/Lin6/Lin6-B111_GLMM_Obs-vs-Fit_AR1PC-AR1PCS1.png]

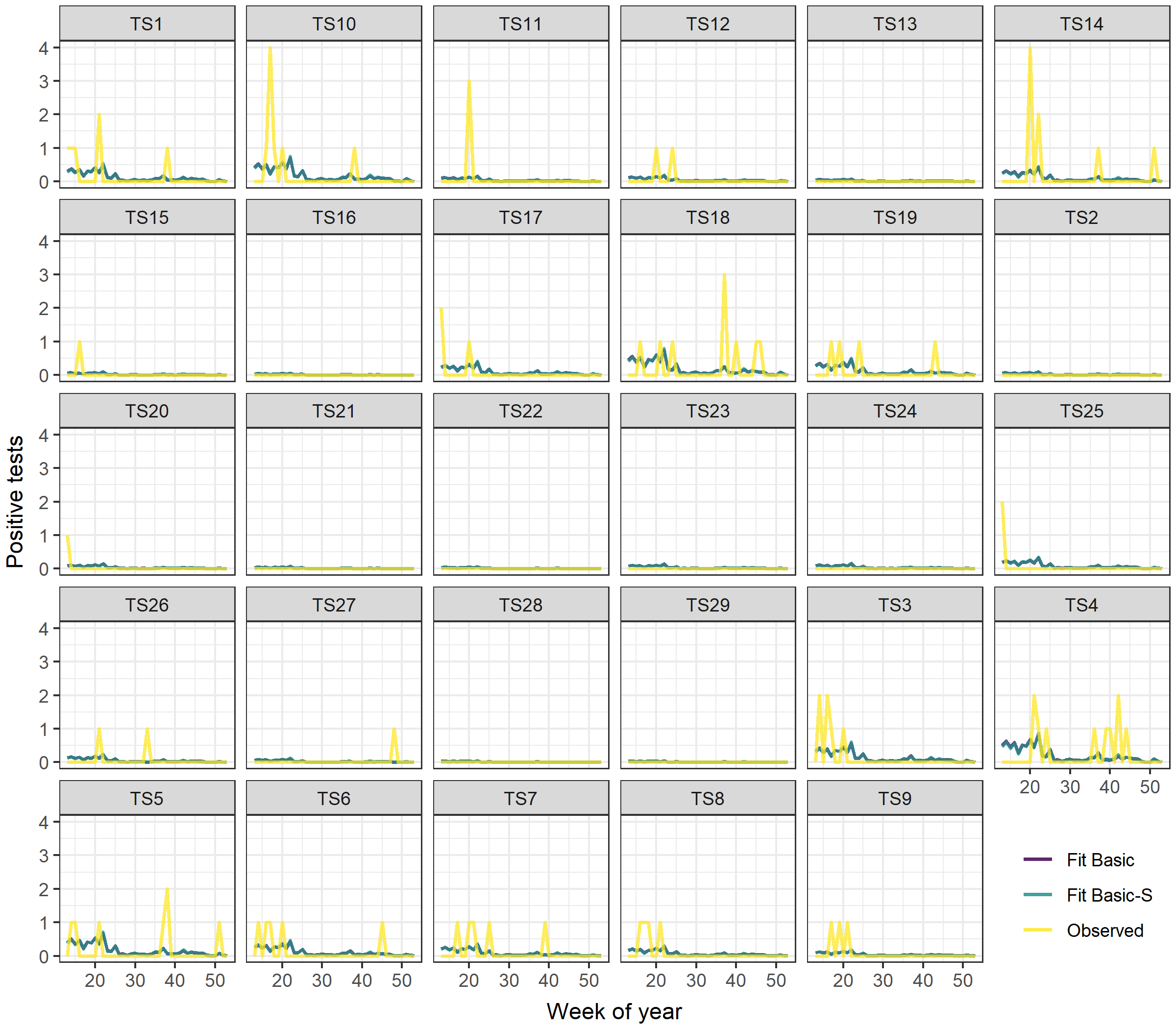

Supplement: Supplementary file: main dataset and code (compressed) [file EMS198536-supplement-Supplementary_file__main_dataset_and_code__compressed_.zip › Covid-19-Teesside-main/Figures/GLMM/Lin6/Lin6-B111_GLMM_Obs-vs-Fit_Basic-BasicS1.png]

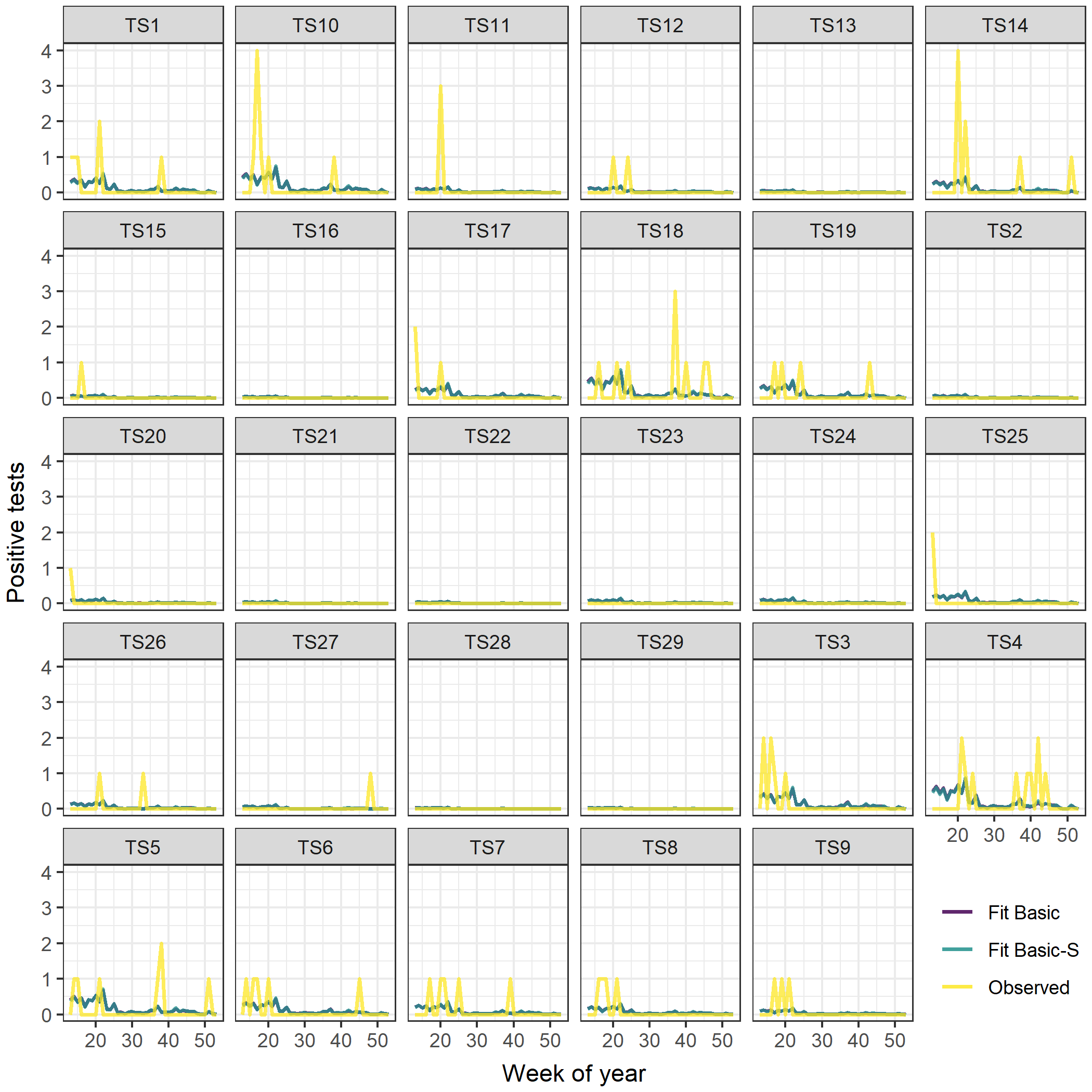

Supplement: Supplementary file: main dataset and code (compressed) [file EMS198536-supplement-Supplementary_file__main_dataset_and_code__compressed_.zip › Covid-19-Teesside-main/Figures/GLMM/Lin6/Lin6-B111_GLMM_Obs-vs-fit_Model-comparison.png]

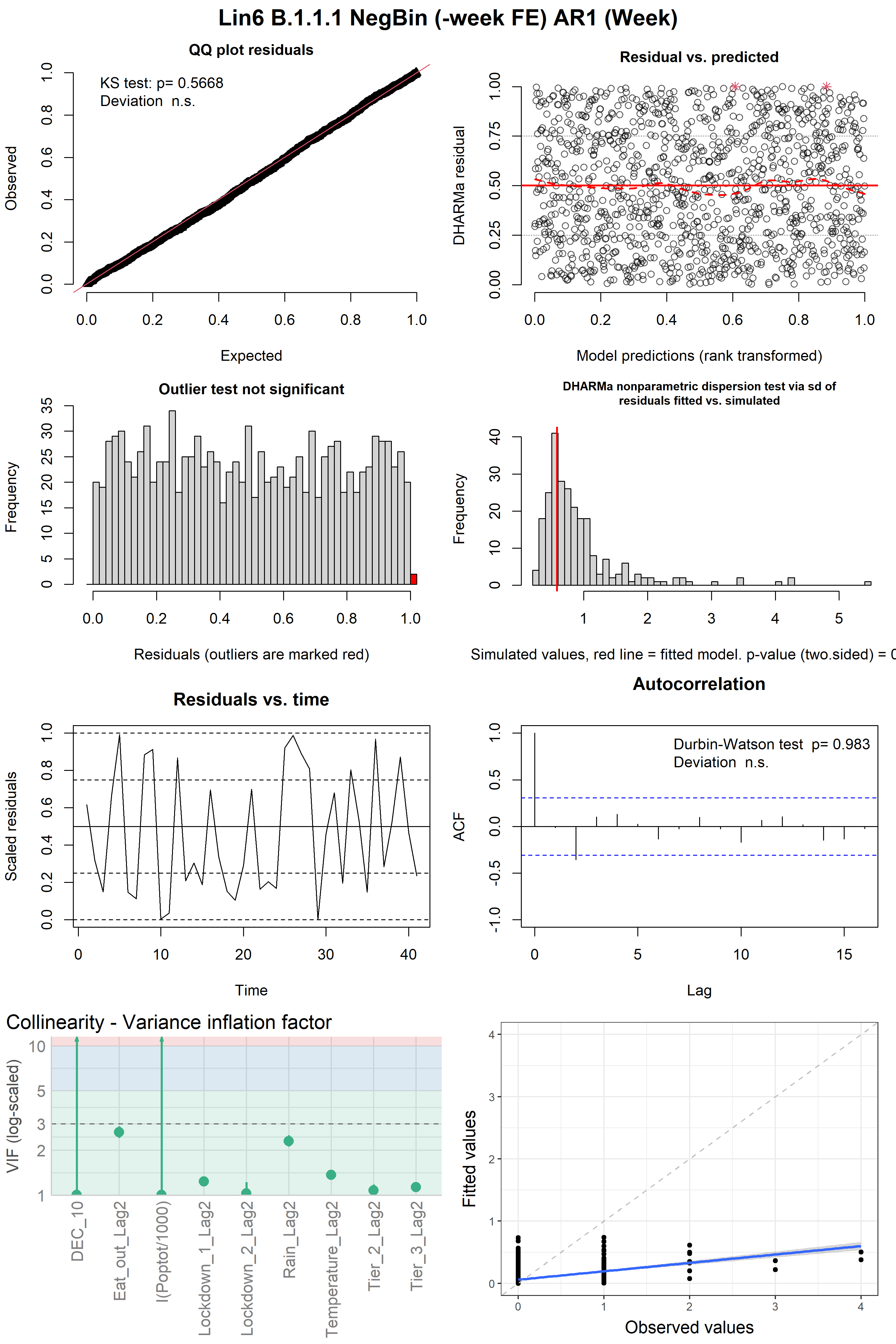

Supplement: Supplementary file: main dataset and code (compressed) [file EMS198536-supplement-Supplementary_file__main_dataset_and_code__compressed_.zip › Covid-19-Teesside-main/Figures/GLMM/Lin6/Lin6-B111_NB_AR1-Week_No-week-FE_Fit.png]

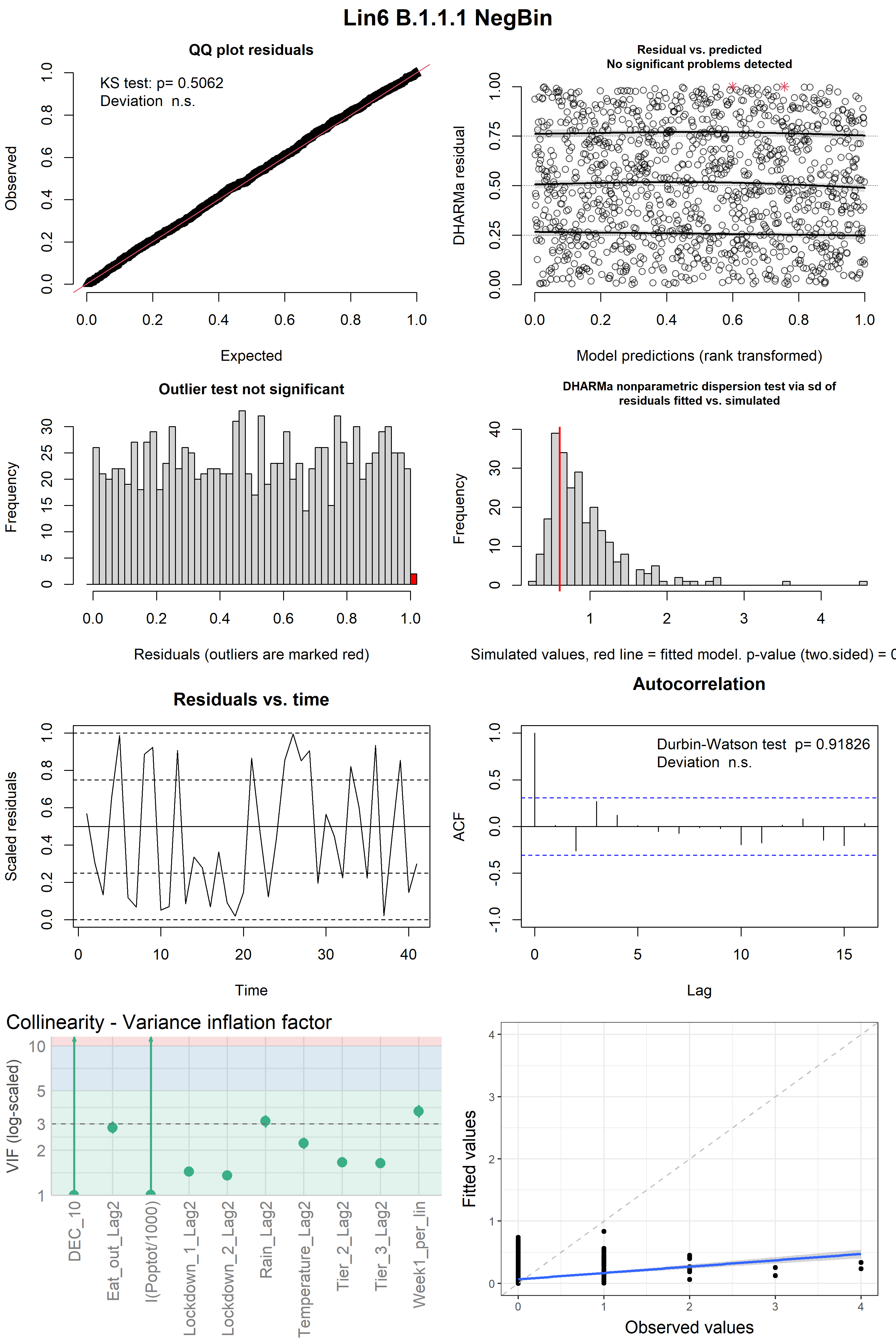

Supplement: Supplementary file: main dataset and code (compressed) [file EMS198536-supplement-Supplementary_file__main_dataset_and_code__compressed_.zip › Covid-19-Teesside-main/Figures/GLMM/Lin6/Lin6-B111_NB_Full_Fit.png]

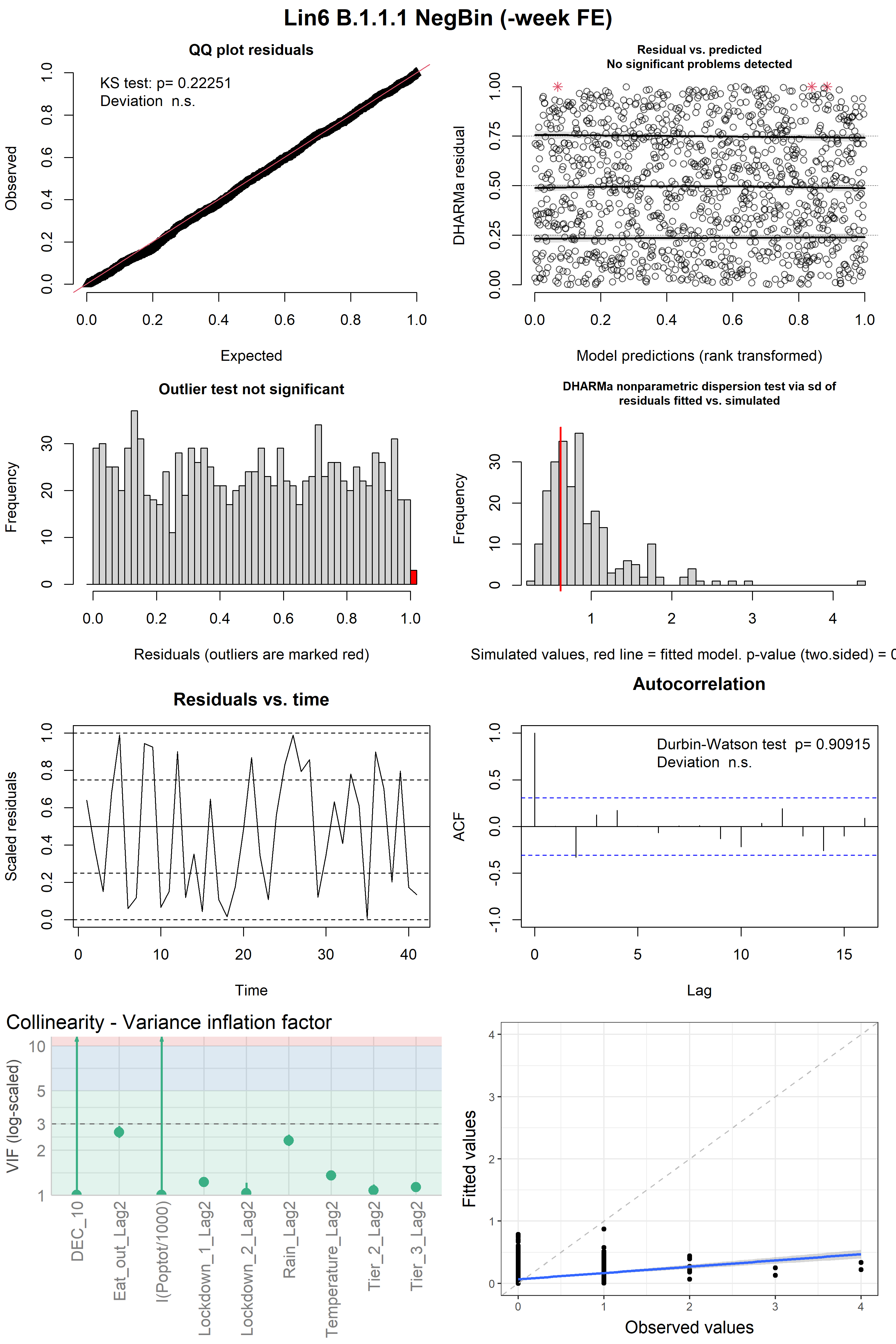

Supplement: Supplementary file: main dataset and code (compressed) [file EMS198536-supplement-Supplementary_file__main_dataset_and_code__compressed_.zip › Covid-19-Teesside-main/Figures/GLMM/Lin6/Lin6-B111_NB_No-week-FE_Fit.png]

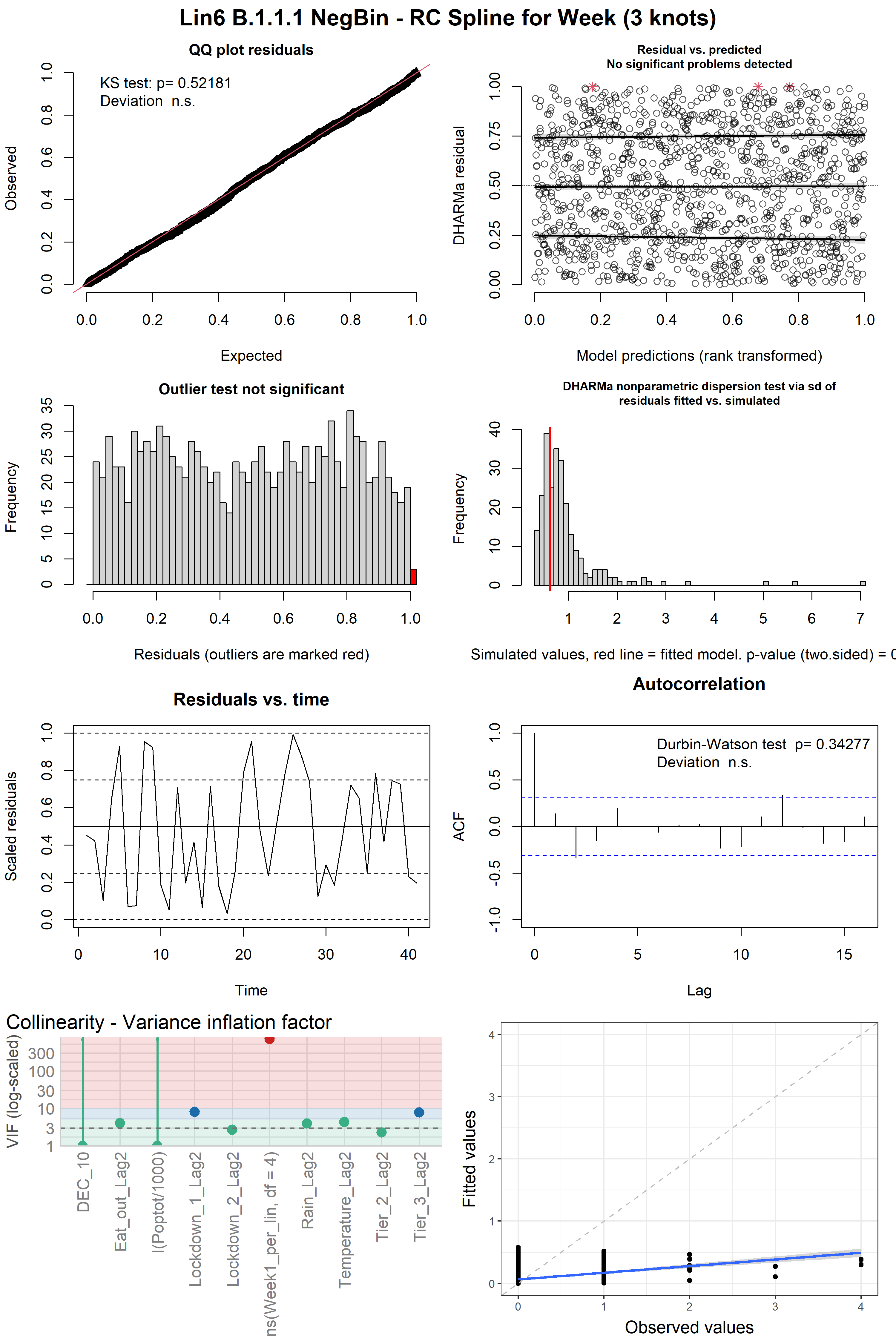

Supplement: Supplementary file: main dataset and code (compressed) [file EMS198536-supplement-Supplementary_file__main_dataset_and_code__compressed_.zip › Covid-19-Teesside-main/Figures/GLMM/Lin6/Lin6-B111_NB_RCS-Week-3knots_Fit.png]

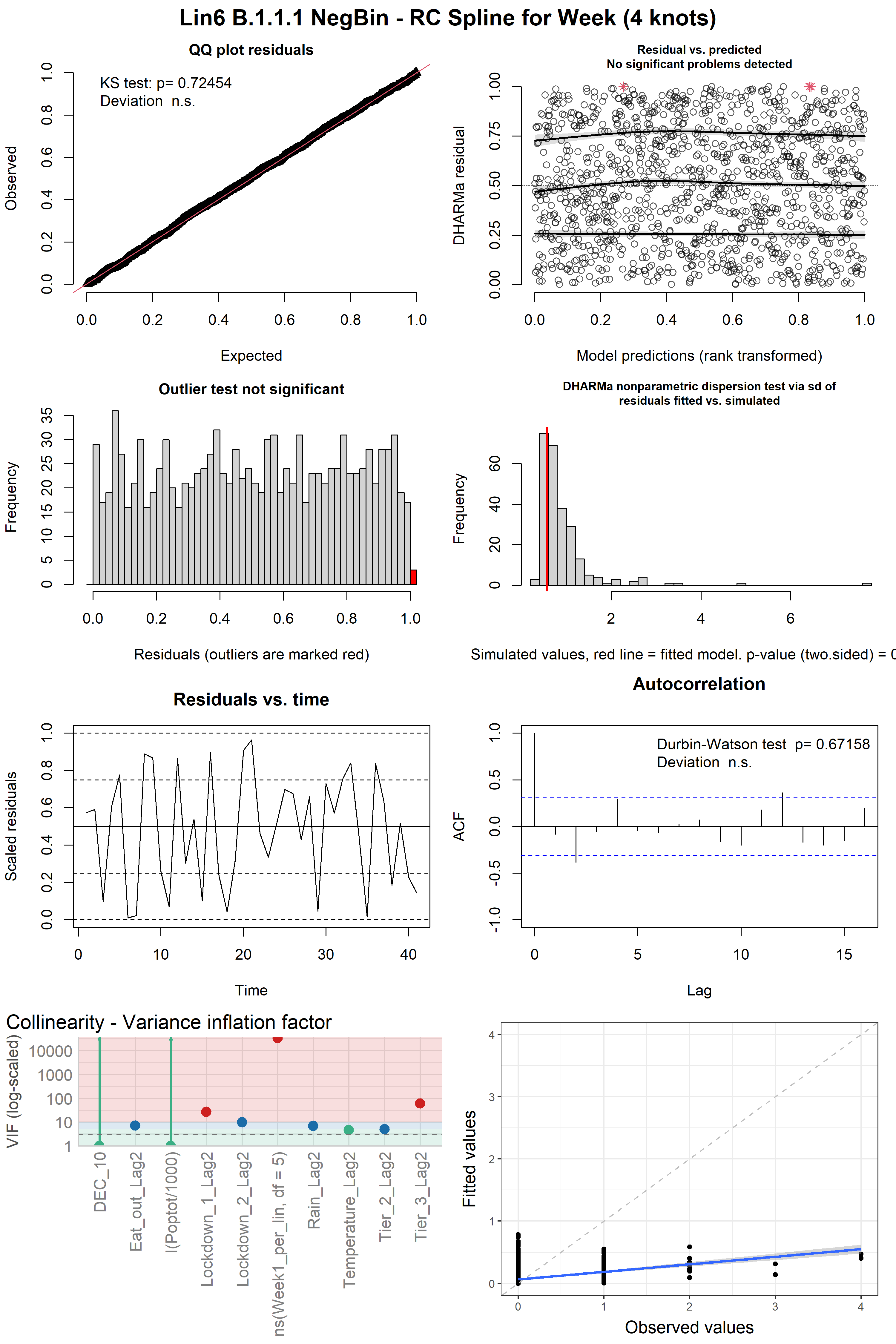

Supplement: Supplementary file: main dataset and code (compressed) [file EMS198536-supplement-Supplementary_file__main_dataset_and_code__compressed_.zip › Covid-19-Teesside-main/Figures/GLMM/Lin6/Lin6-B111_NB_RCS-Week-4knots_Fit.png]

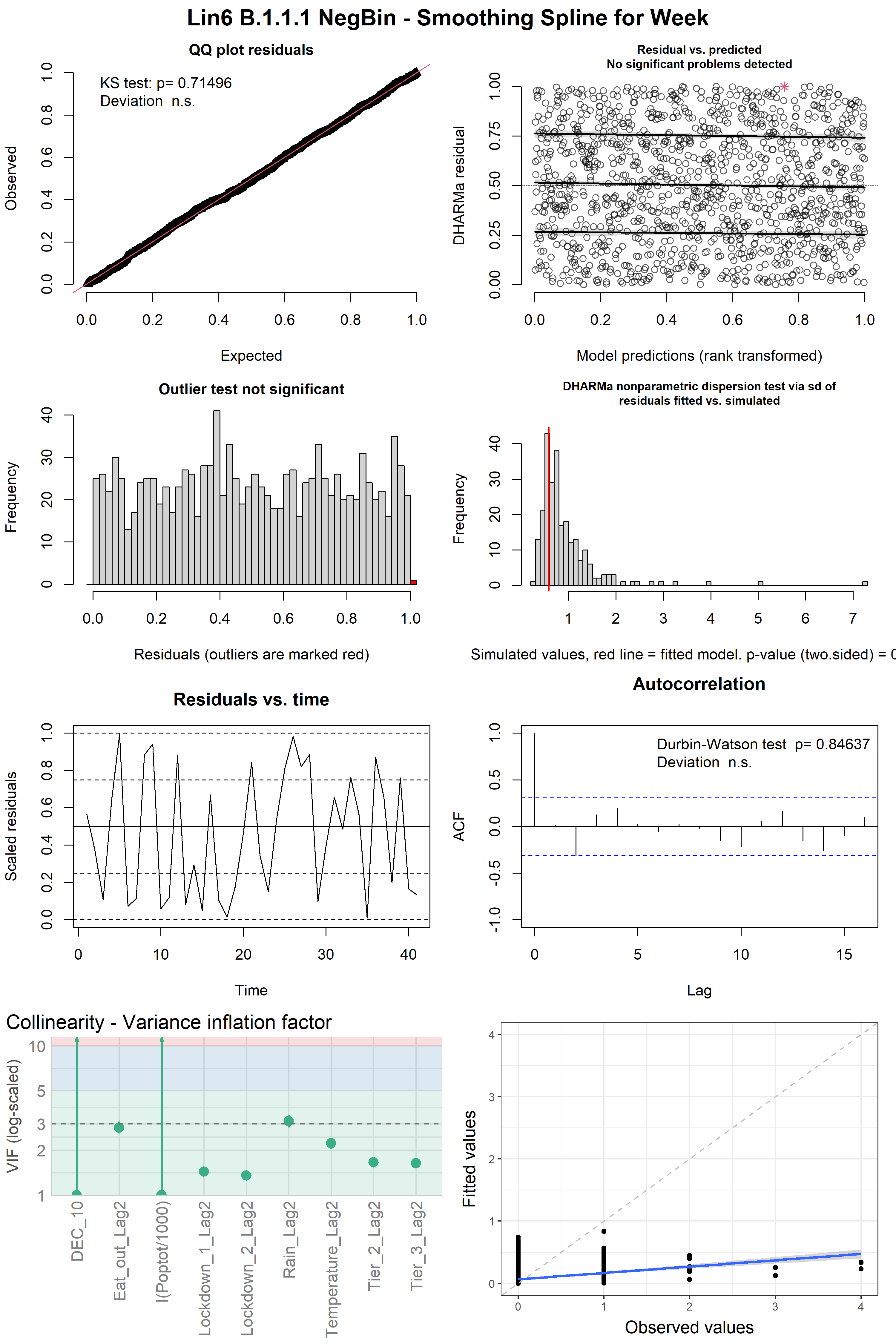

Supplement: Supplementary file: main dataset and code (compressed) [file EMS198536-supplement-Supplementary_file__main_dataset_and_code__compressed_.zip › Covid-19-Teesside-main/Figures/GLMM/Lin6/Lin6-B111_NB_SmoothSpline-Week-TPS_Fit.png]

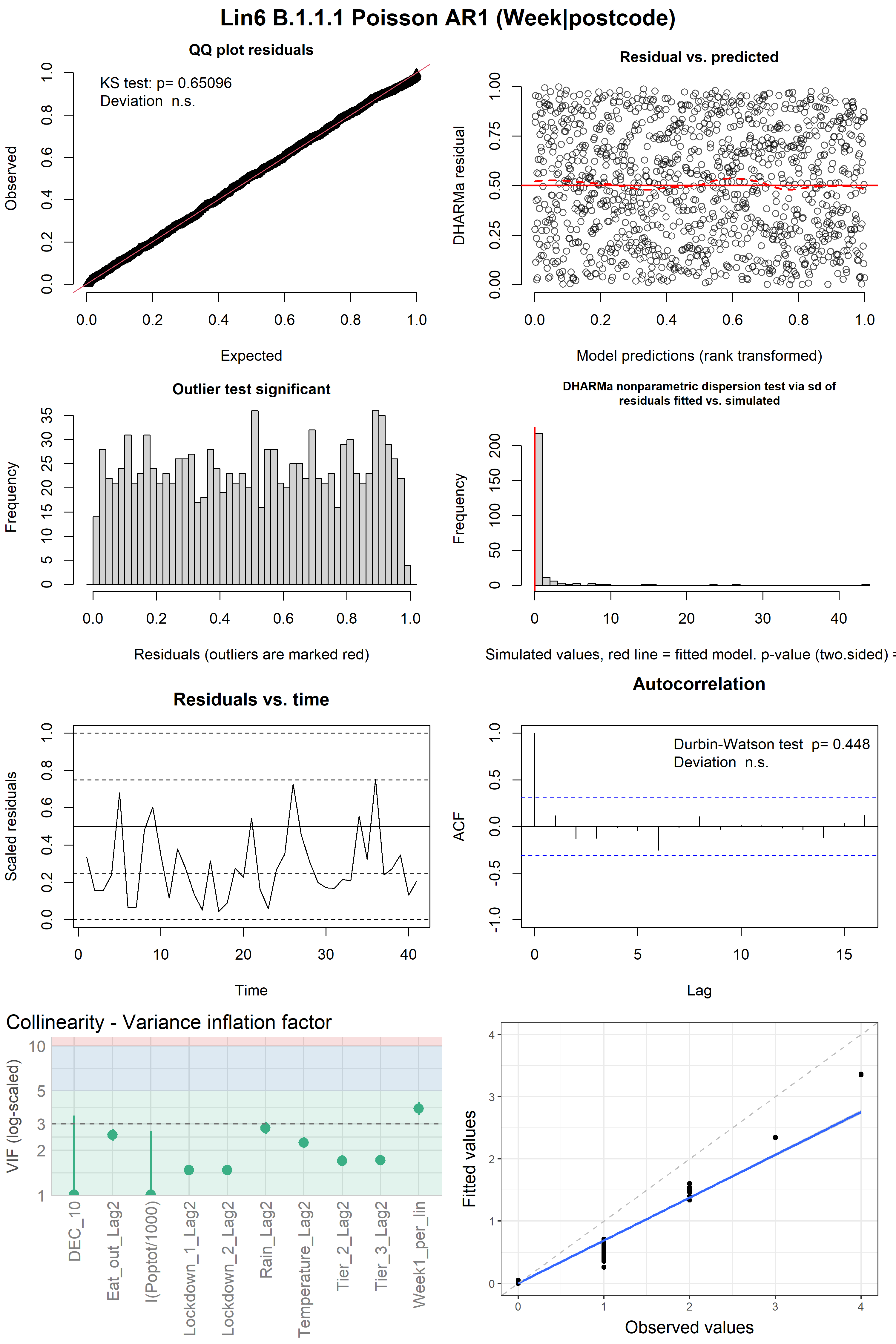

Supplement: Supplementary file: main dataset and code (compressed) [file EMS198536-supplement-Supplementary_file__main_dataset_and_code__compressed_.zip › Covid-19-Teesside-main/Figures/GLMM/Lin6/Lin6-B111_Po_AR1-Week-Postcode_Fit.png]

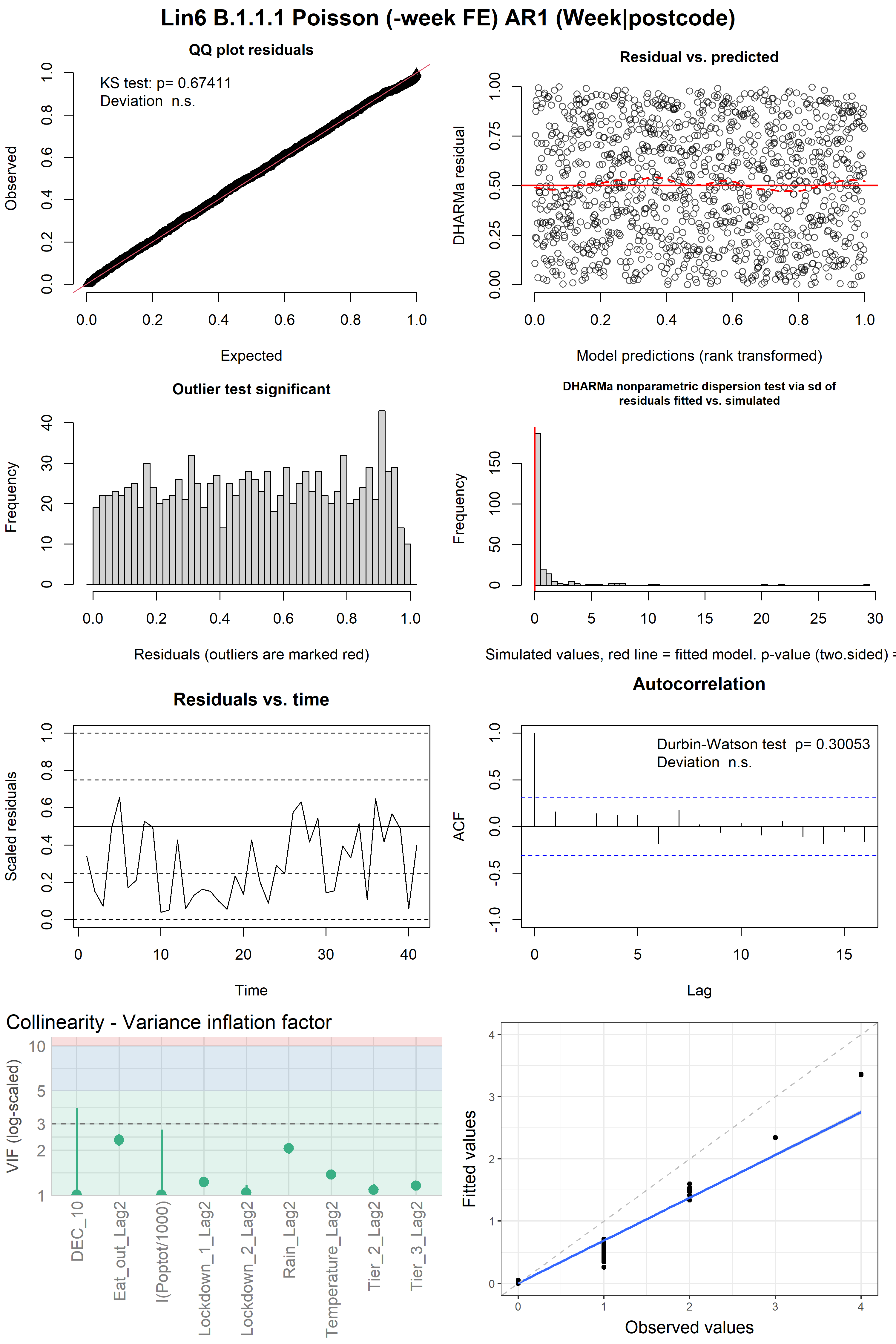

Supplement: Supplementary file: main dataset and code (compressed) [file EMS198536-supplement-Supplementary_file__main_dataset_and_code__compressed_.zip › Covid-19-Teesside-main/Figures/GLMM/Lin6/Lin6-B111_Po_AR1-Week-Postcode_No-week-FE_Fit.png]
