# Supplementary figures and images for: Spatial and temporal epidemiology of SARS-CoV-2 virus lineages in Teesside, UK, in 2020: effects of socio-economic deprivation, weather, and lockdown on lineage dynamics (part 1 of 2)
Source: Peer Community J. Author manuscript; Available in PMC 2024 Sep 23. (PMC7616629; doi:10.24072/pcjournal.461)

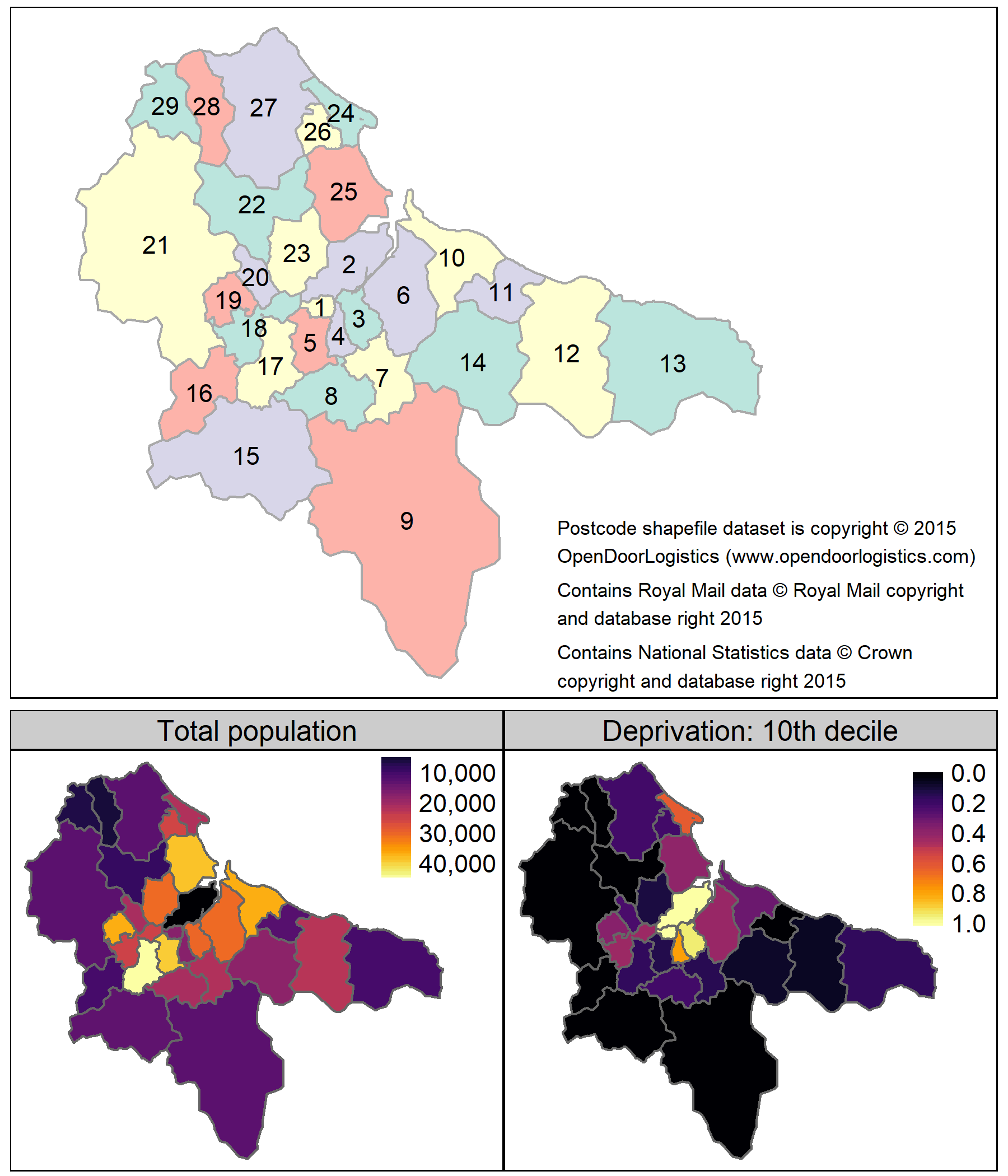

Supplement: Supplementary file: main dataset and code (compressed) [file EMS198536-supplement-Supplementary_file__main_dataset_and_code__compressed_.zip › Covid-19-Teesside-main/Figures/Figure-1.png]

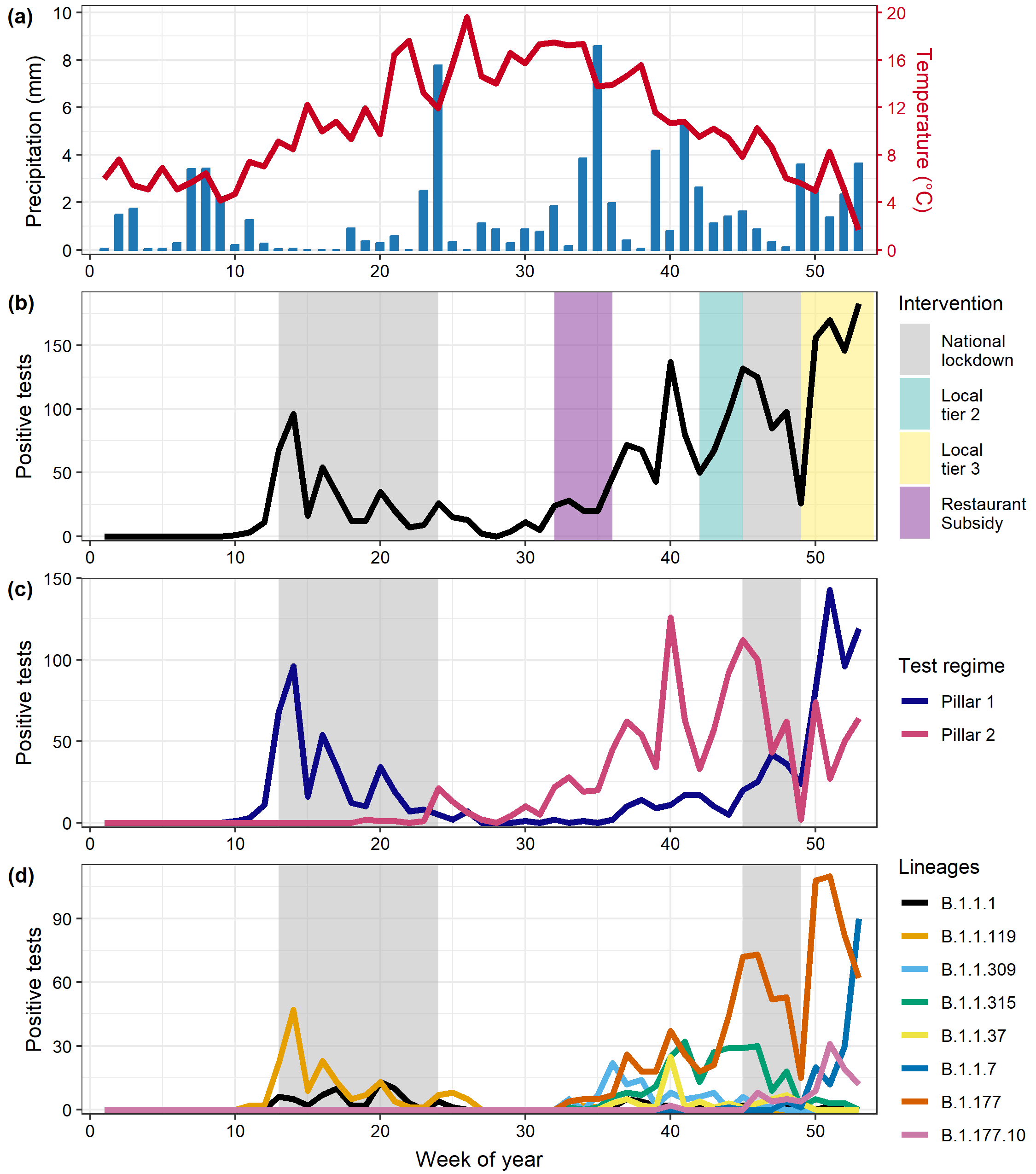

Supplement: Supplementary file: main dataset and code (compressed) [file EMS198536-supplement-Supplementary_file__main_dataset_and_code__compressed_.zip › Covid-19-Teesside-main/Figures/Figure-2.png]

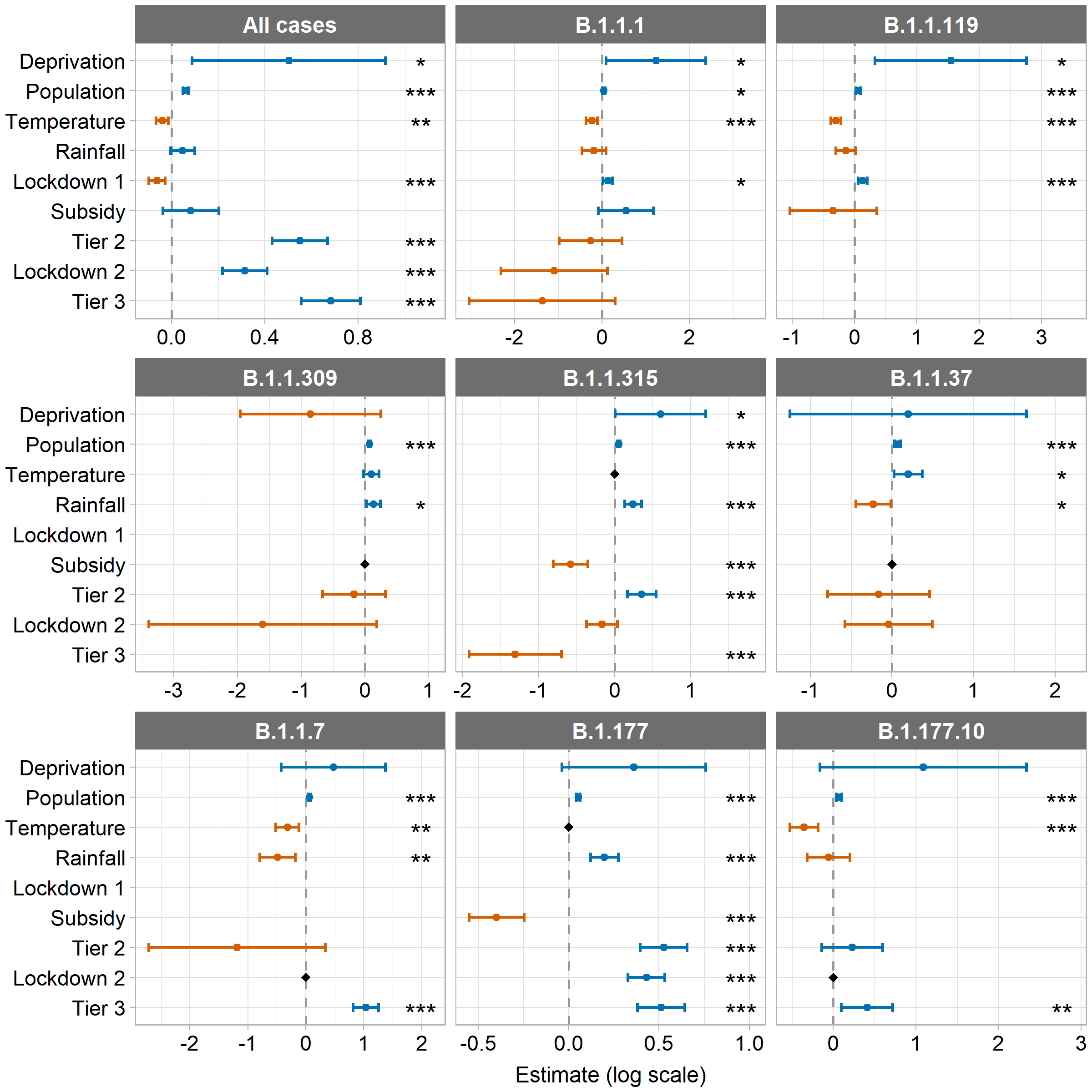

Supplement: Supplementary file: main dataset and code (compressed) [file EMS198536-supplement-Supplementary_file__main_dataset_and_code__compressed_.zip › Covid-19-Teesside-main/Figures/GLMM-Output/All-cases-8-lineages_GLMM-Simplified_Fixed-effects-log_VIF.png]

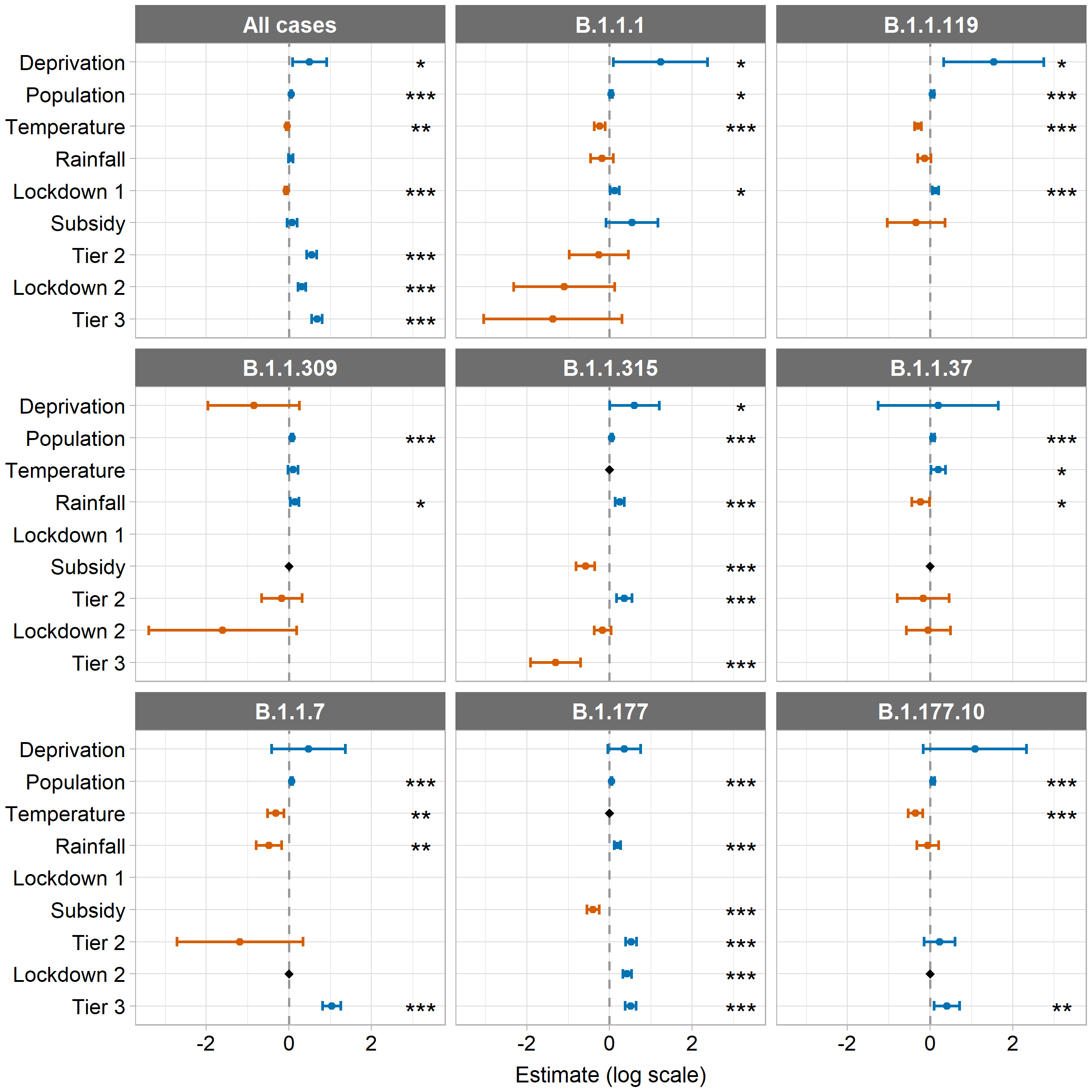

Supplement: Supplementary file: main dataset and code (compressed) [file EMS198536-supplement-Supplementary_file__main_dataset_and_code__compressed_.zip › Covid-19-Teesside-main/Figures/GLMM-Output/All-cases-8-lineages_GLMM-Simplified_Fixed-effects-log_VIF_scales-fixed.png]

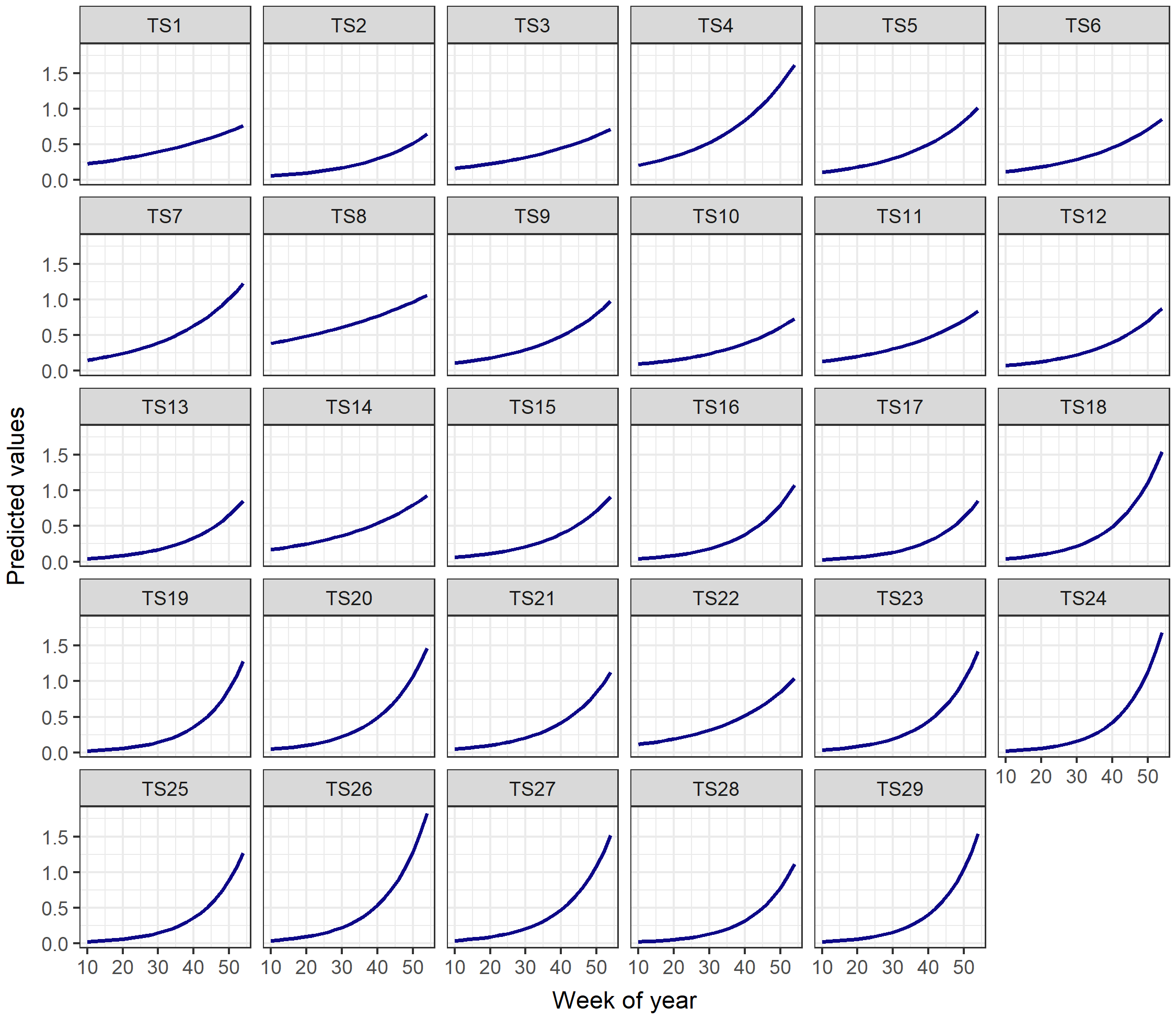

Supplement: Supplementary file: main dataset and code (compressed) [file EMS198536-supplement-Supplementary_file__main_dataset_and_code__compressed_.zip › Covid-19-Teesside-main/Figures/GLMM-Output/All-cases-GLMM-Gradient-F_Pred-Gradients.png]

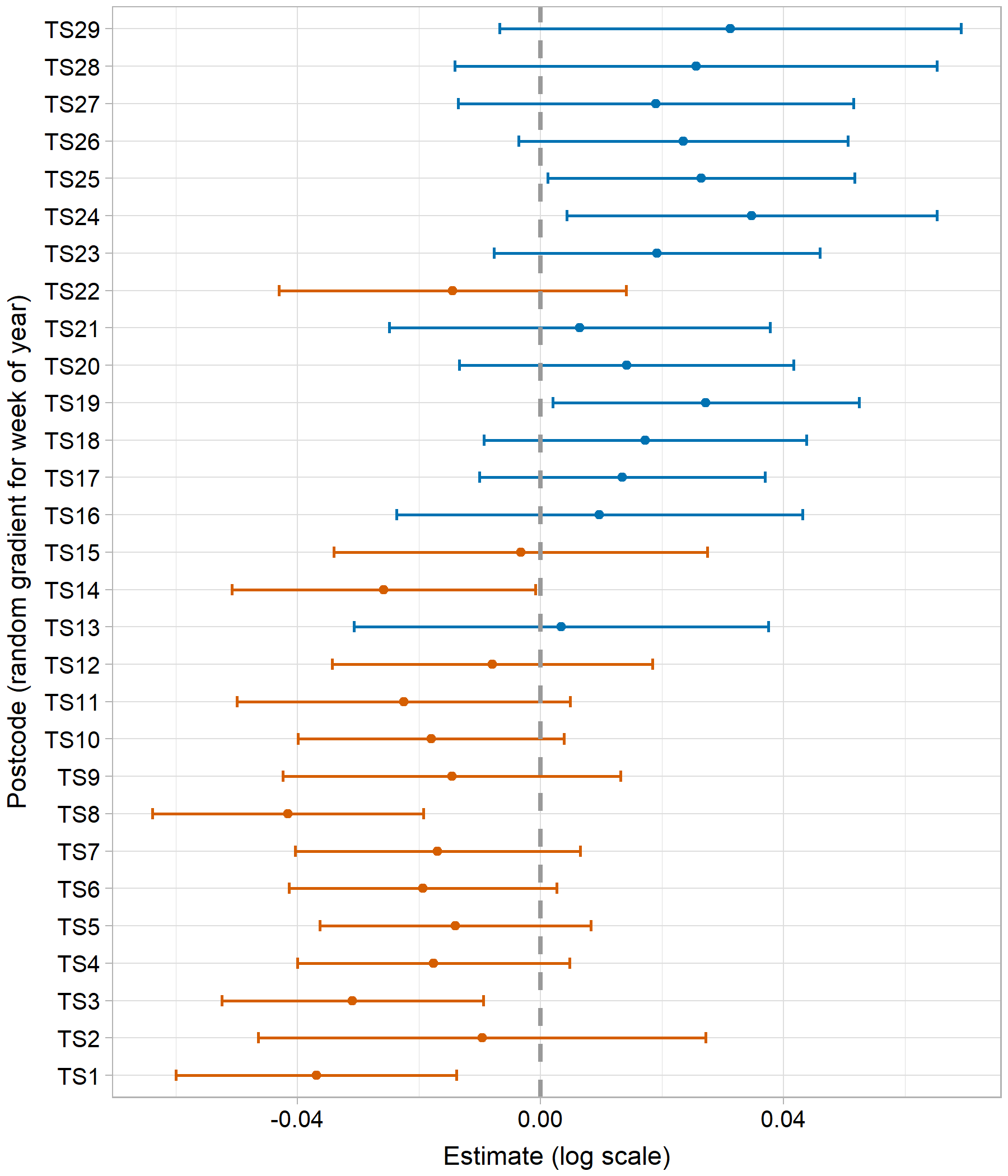

Supplement: Supplementary file: main dataset and code (compressed) [file EMS198536-supplement-Supplementary_file__main_dataset_and_code__compressed_.zip › Covid-19-Teesside-main/Figures/GLMM-Output/All-cases-GLMM-Gradient-F_Random-effects-log.png]

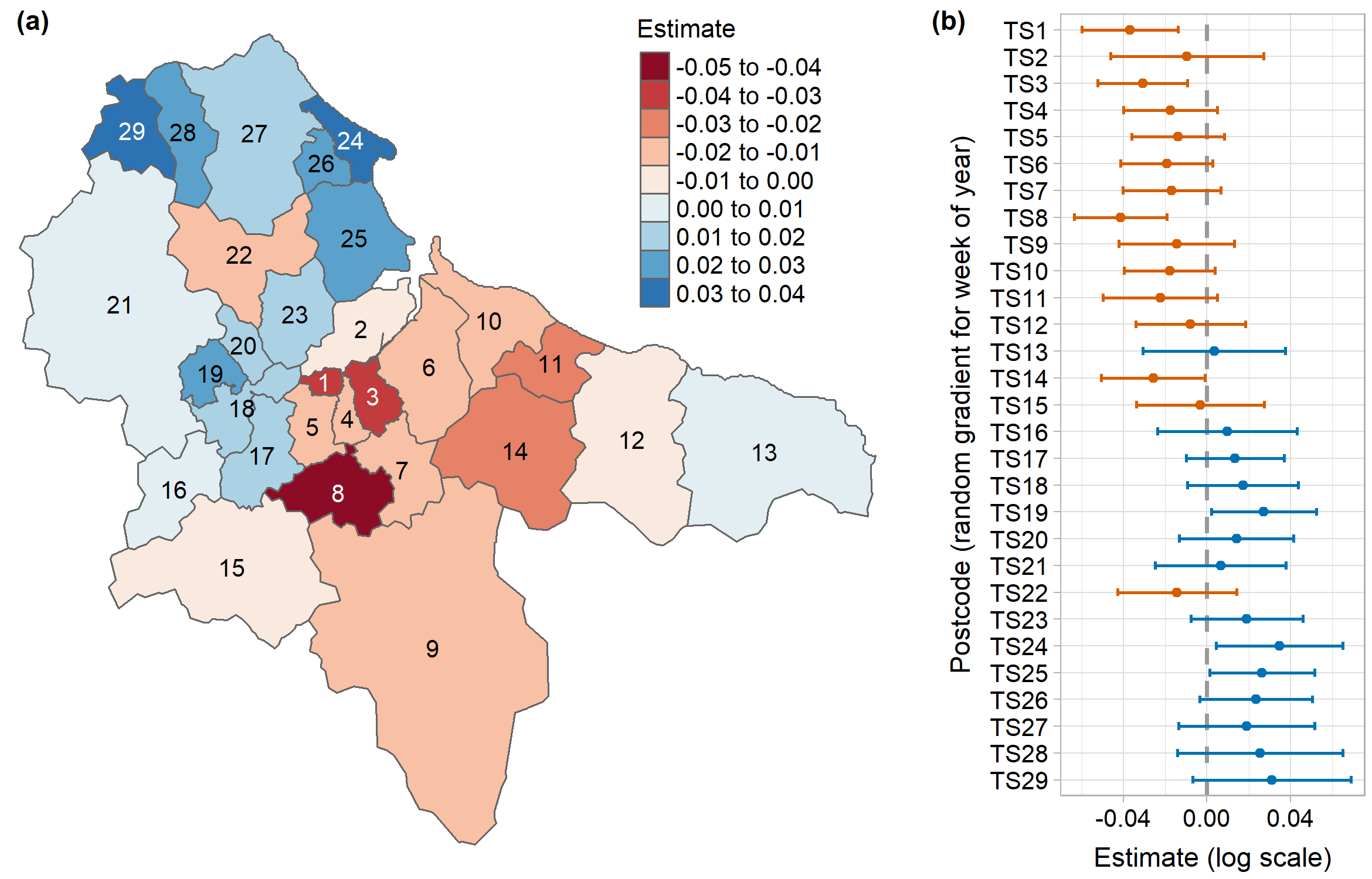

Supplement: Supplementary file: main dataset and code (compressed) [file EMS198536-supplement-Supplementary_file__main_dataset_and_code__compressed_.zip › Covid-19-Teesside-main/Figures/GLMM-Output/All-cases-GLMM-Gradient-F_Random-effects-log_Point-Map.png]

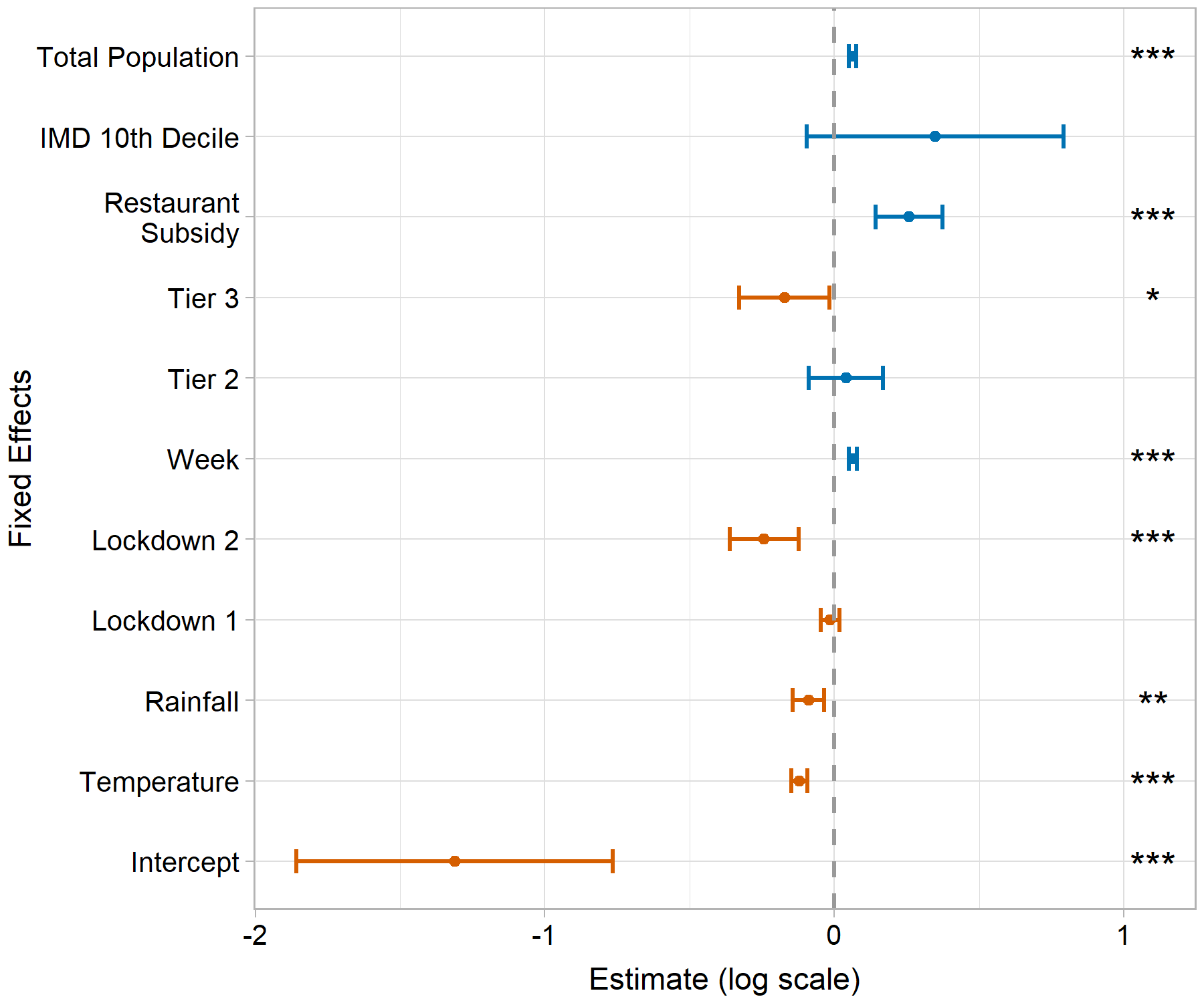

Supplement: Supplementary file: main dataset and code (compressed) [file EMS198536-supplement-Supplementary_file__main_dataset_and_code__compressed_.zip › Covid-19-Teesside-main/Figures/GLMM-Output/All-cases-GLMM-Gradient-Full_Fixed-effects-log.png]

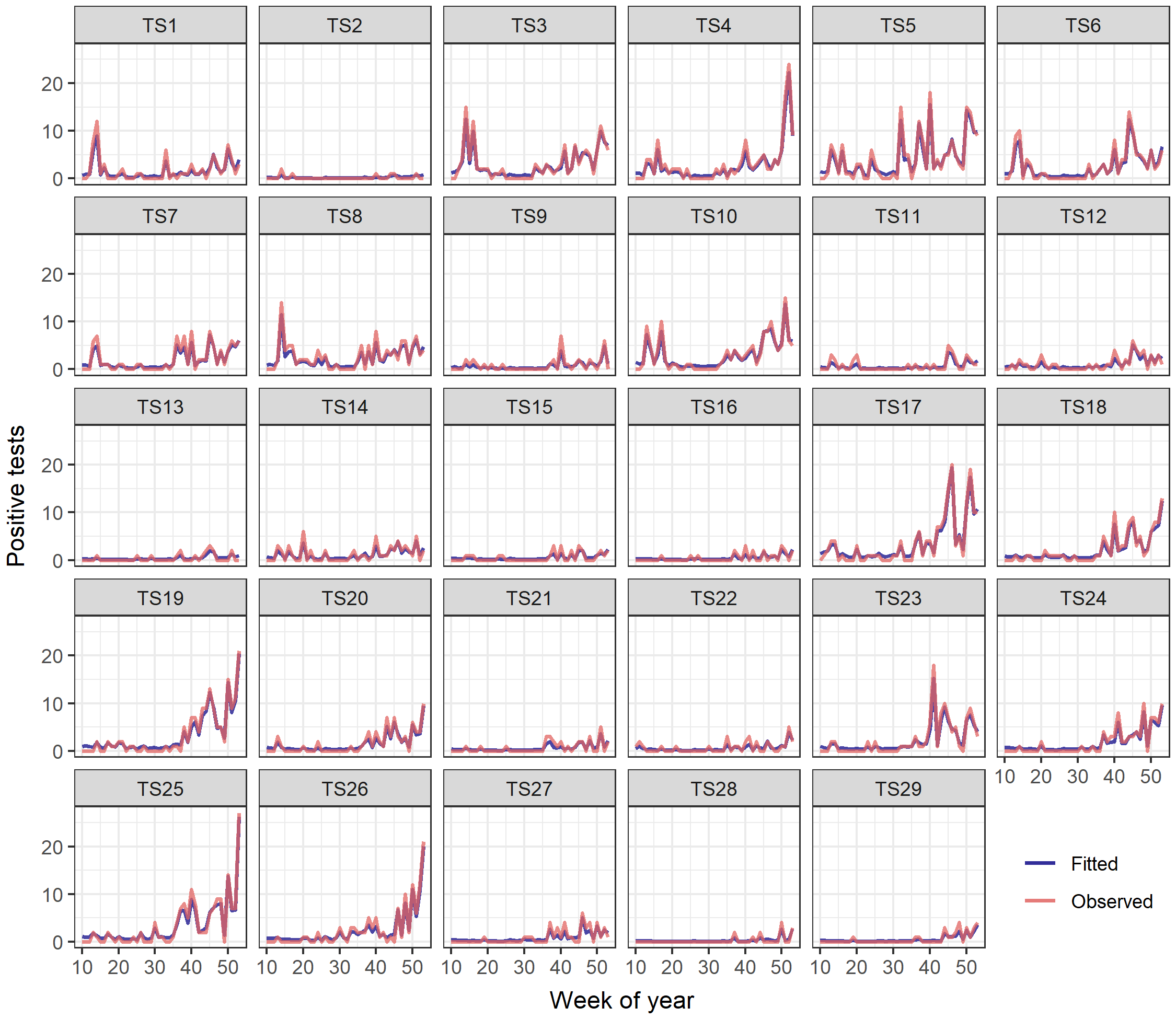

Supplement: Supplementary file: main dataset and code (compressed) [file EMS198536-supplement-Supplementary_file__main_dataset_and_code__compressed_.zip › Covid-19-Teesside-main/Figures/GLMM-Output/All-cases_GLMM_AR1PCS1_Obs-vs-fit.png]

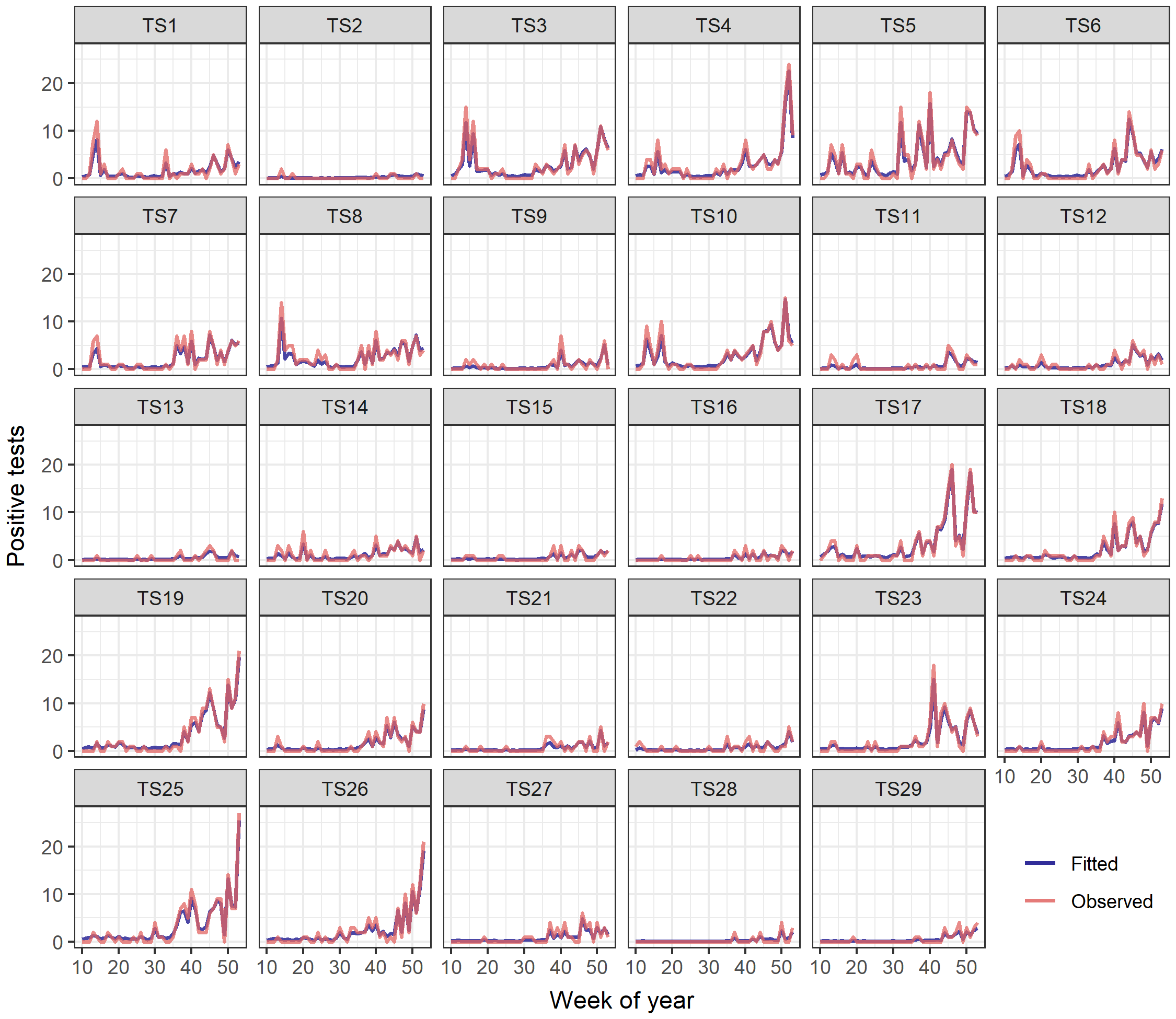

Supplement: Supplementary file: main dataset and code (compressed) [file EMS198536-supplement-Supplementary_file__main_dataset_and_code__compressed_.zip › Covid-19-Teesside-main/Figures/GLMM-Output/All-cases_GLMM_AR1PC_Obs-vs-fit.png]

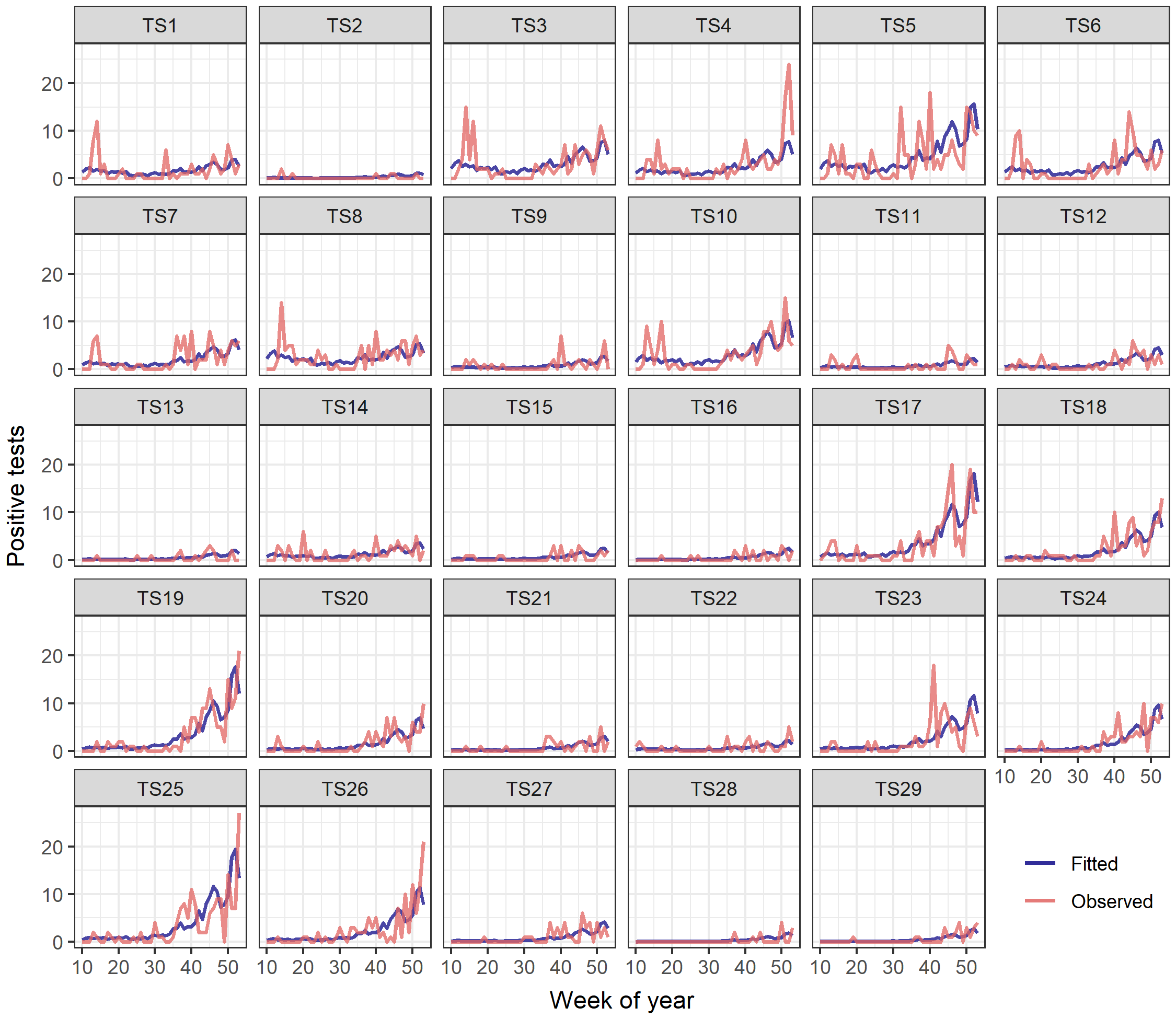

Supplement: Supplementary file: main dataset and code (compressed) [file EMS198536-supplement-Supplementary_file__main_dataset_and_code__compressed_.zip › Covid-19-Teesside-main/Figures/GLMM-Output/All-cases_GLMM_Gradient-F_Obs-vs-fit.png]

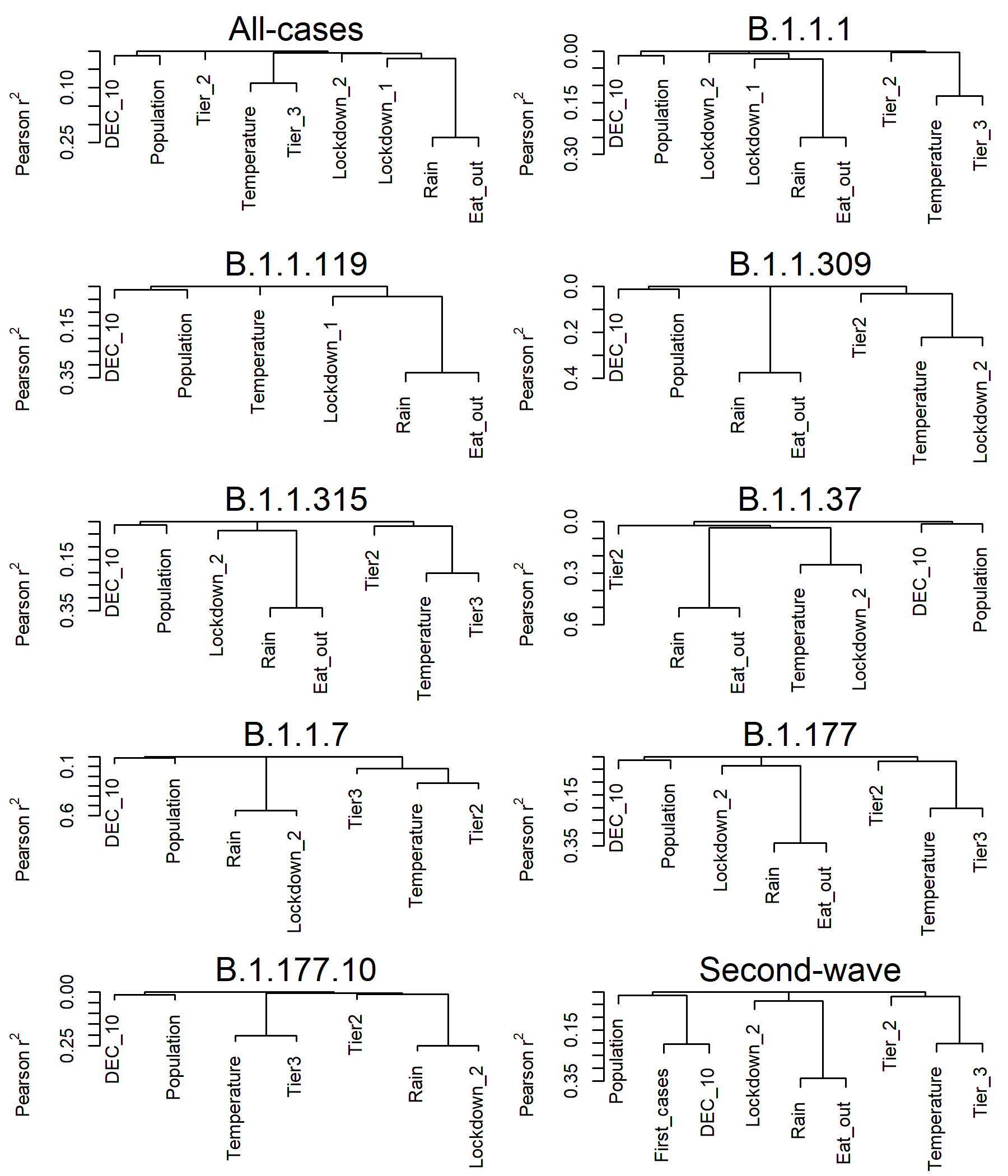

Supplement: Supplementary file: main dataset and code (compressed) [file EMS198536-supplement-Supplementary_file__main_dataset_and_code__compressed_.zip › Covid-19-Teesside-main/Figures/GLMM/All-cases-8-lineages_Variable-Clustering_Pearson_Without-week.png]

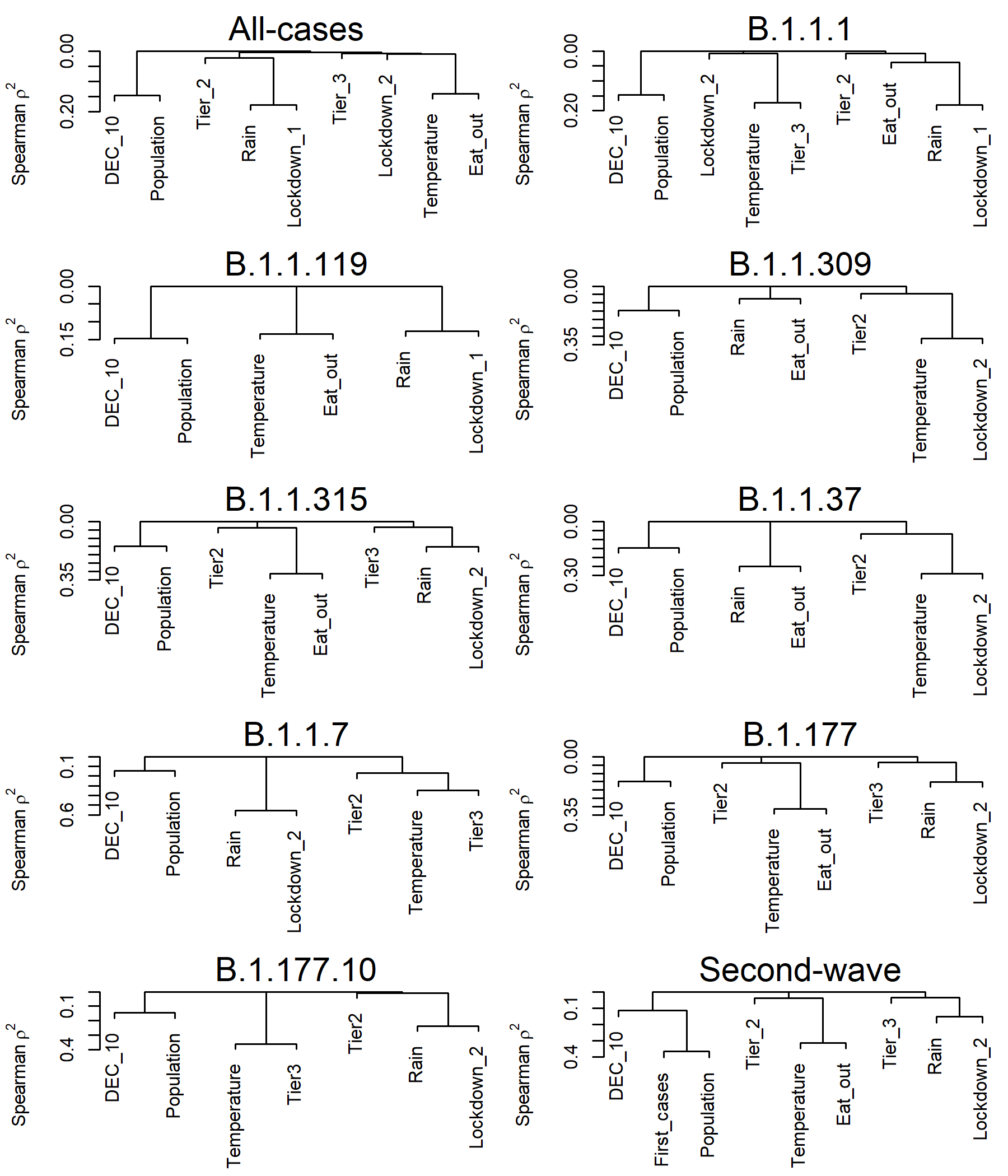

Supplement: Supplementary file: main dataset and code (compressed) [file EMS198536-supplement-Supplementary_file__main_dataset_and_code__compressed_.zip › Covid-19-Teesside-main/Figures/GLMM/All-cases-8-lineages_Variable-Clustering_Spearman_Without-week.png]

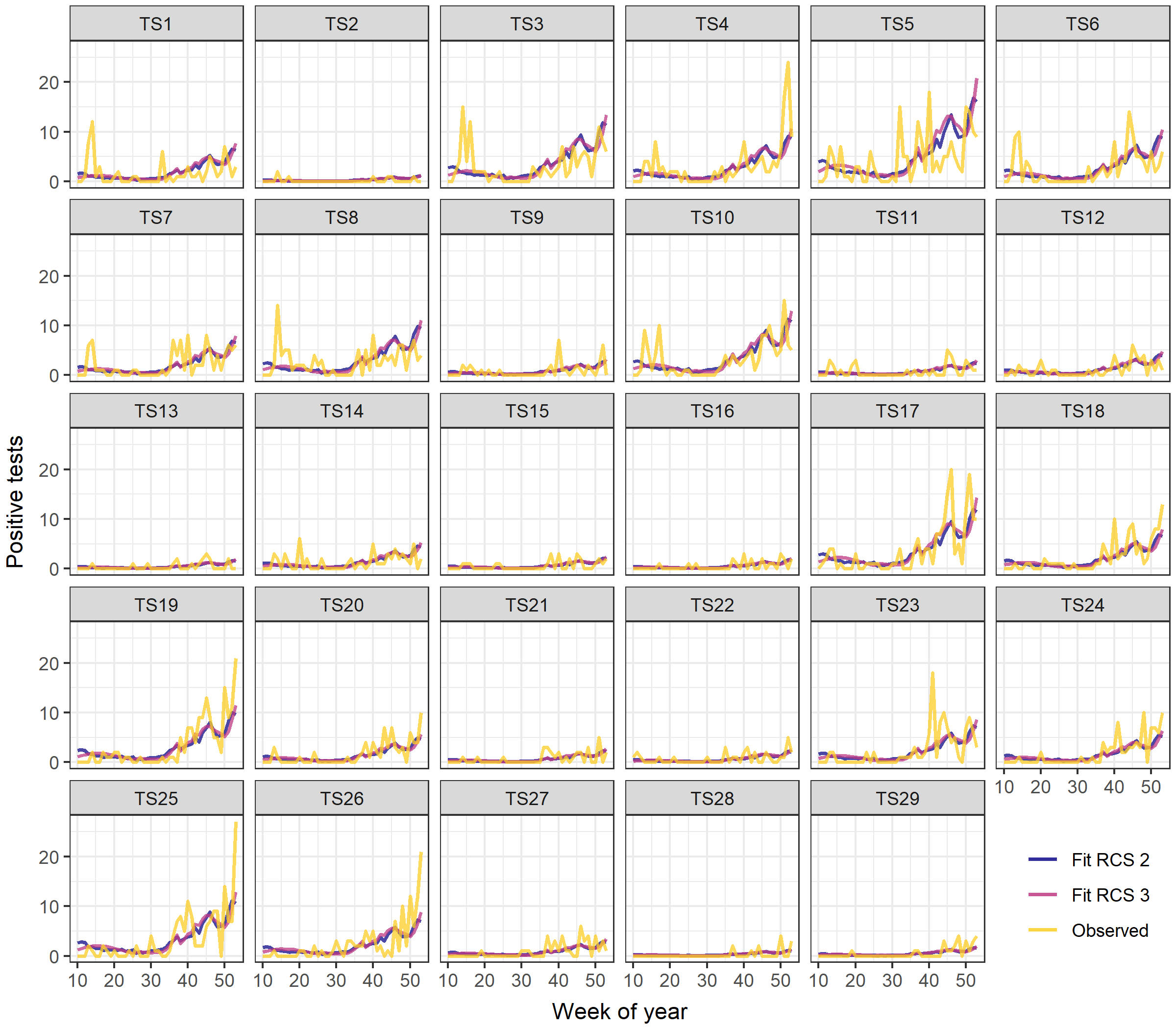

Supplement: Supplementary file: main dataset and code (compressed) [file EMS198536-supplement-Supplementary_file__main_dataset_and_code__compressed_.zip › Covid-19-Teesside-main/Figures/GLMM/All-cases-GLMM-F_Obs-vs-fit_RCS23.png]

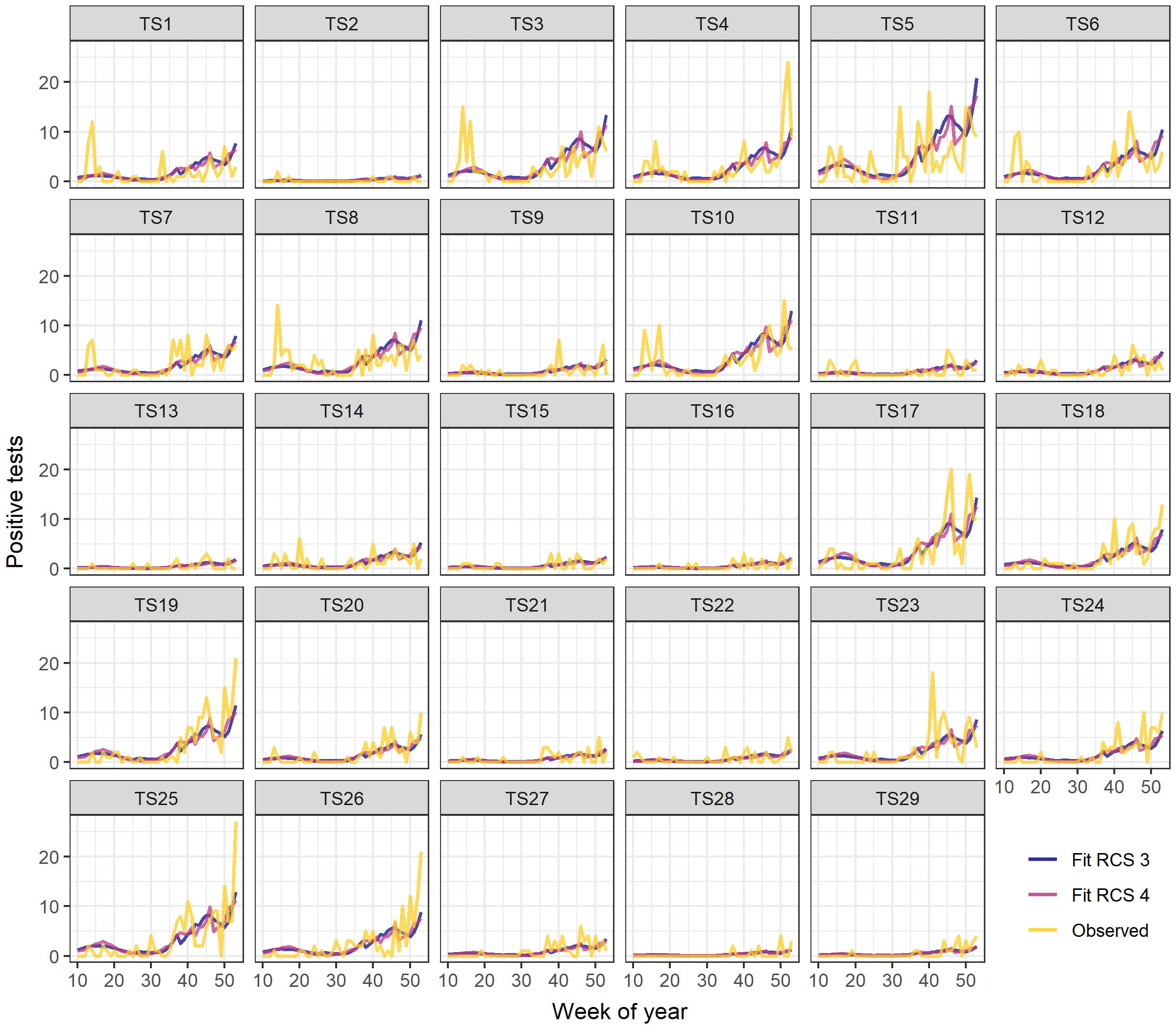

Supplement: Supplementary file: main dataset and code (compressed) [file EMS198536-supplement-Supplementary_file__main_dataset_and_code__compressed_.zip › Covid-19-Teesside-main/Figures/GLMM/All-cases-GLMM-F_Obs-vs-fit_RCS34.png]

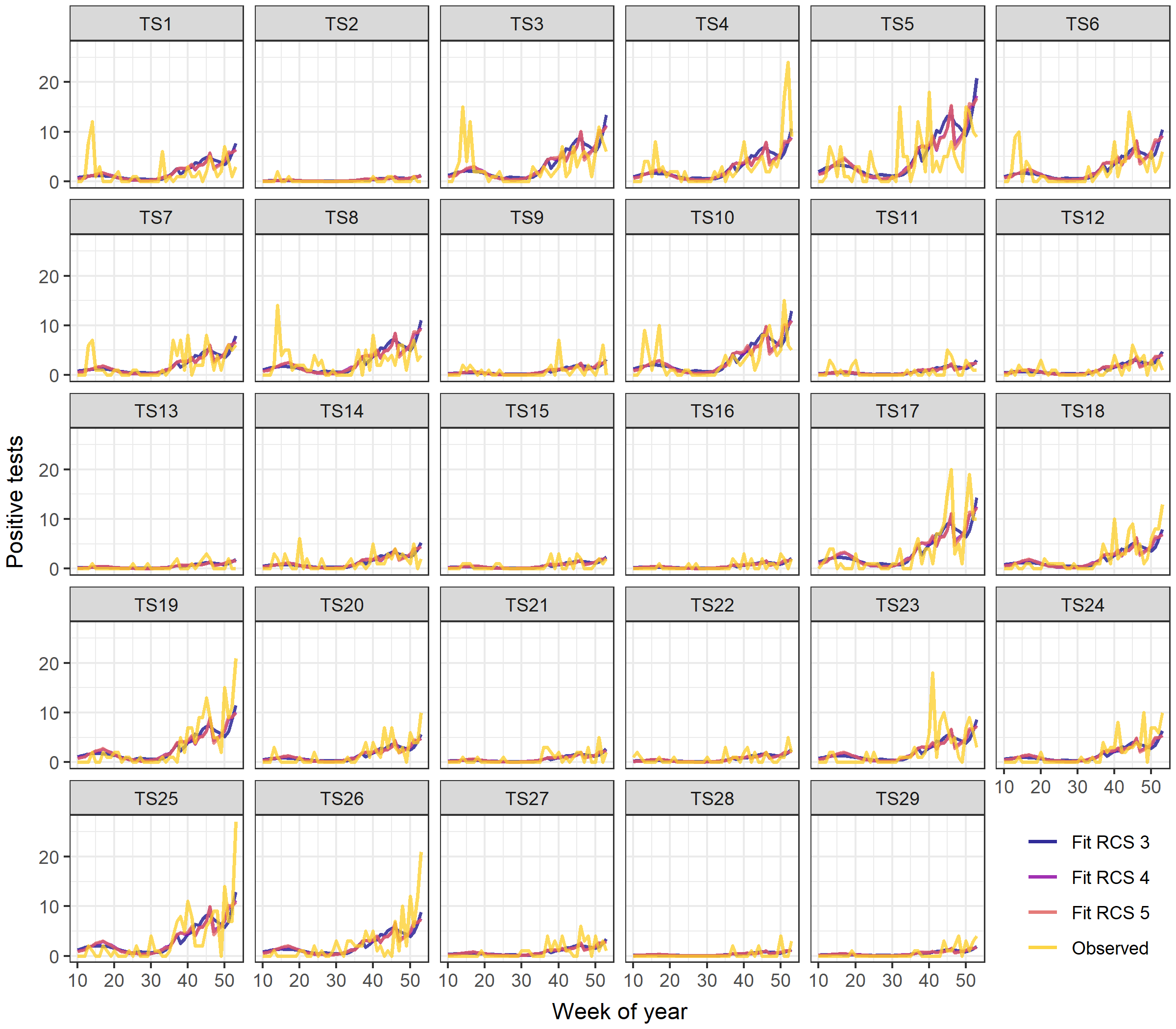

Supplement: Supplementary file: main dataset and code (compressed) [file EMS198536-supplement-Supplementary_file__main_dataset_and_code__compressed_.zip › Covid-19-Teesside-main/Figures/GLMM/All-cases-GLMM-F_Obs-vs-fit_RCS345.png]

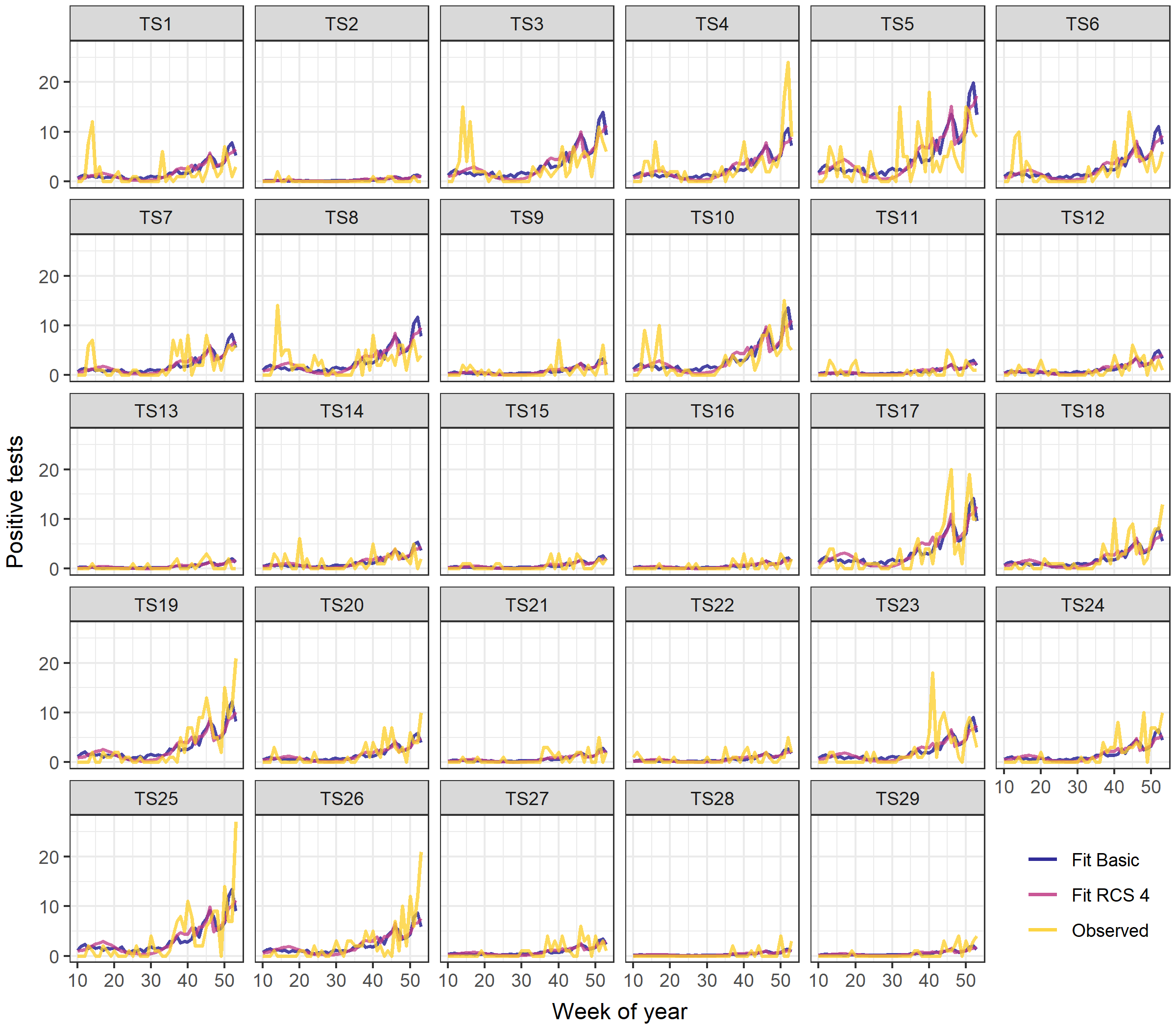

Supplement: Supplementary file: main dataset and code (compressed) [file EMS198536-supplement-Supplementary_file__main_dataset_and_code__compressed_.zip › Covid-19-Teesside-main/Figures/GLMM/All-cases-GLMM-F_Obs-vs-fit_RCS4-basic.png]

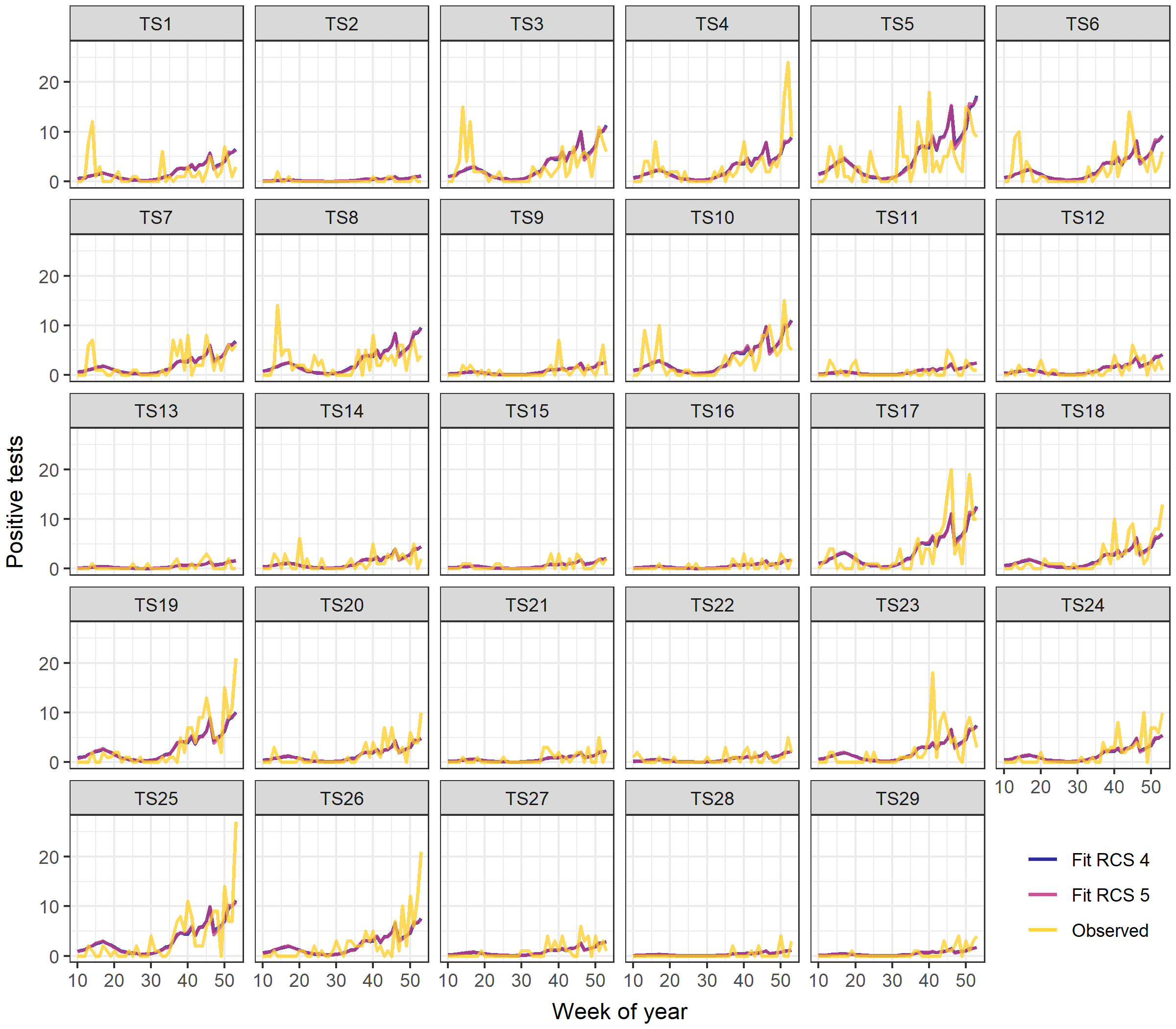

Supplement: Supplementary file: main dataset and code (compressed) [file EMS198536-supplement-Supplementary_file__main_dataset_and_code__compressed_.zip › Covid-19-Teesside-main/Figures/GLMM/All-cases-GLMM-F_Obs-vs-fit_RCS45.png]

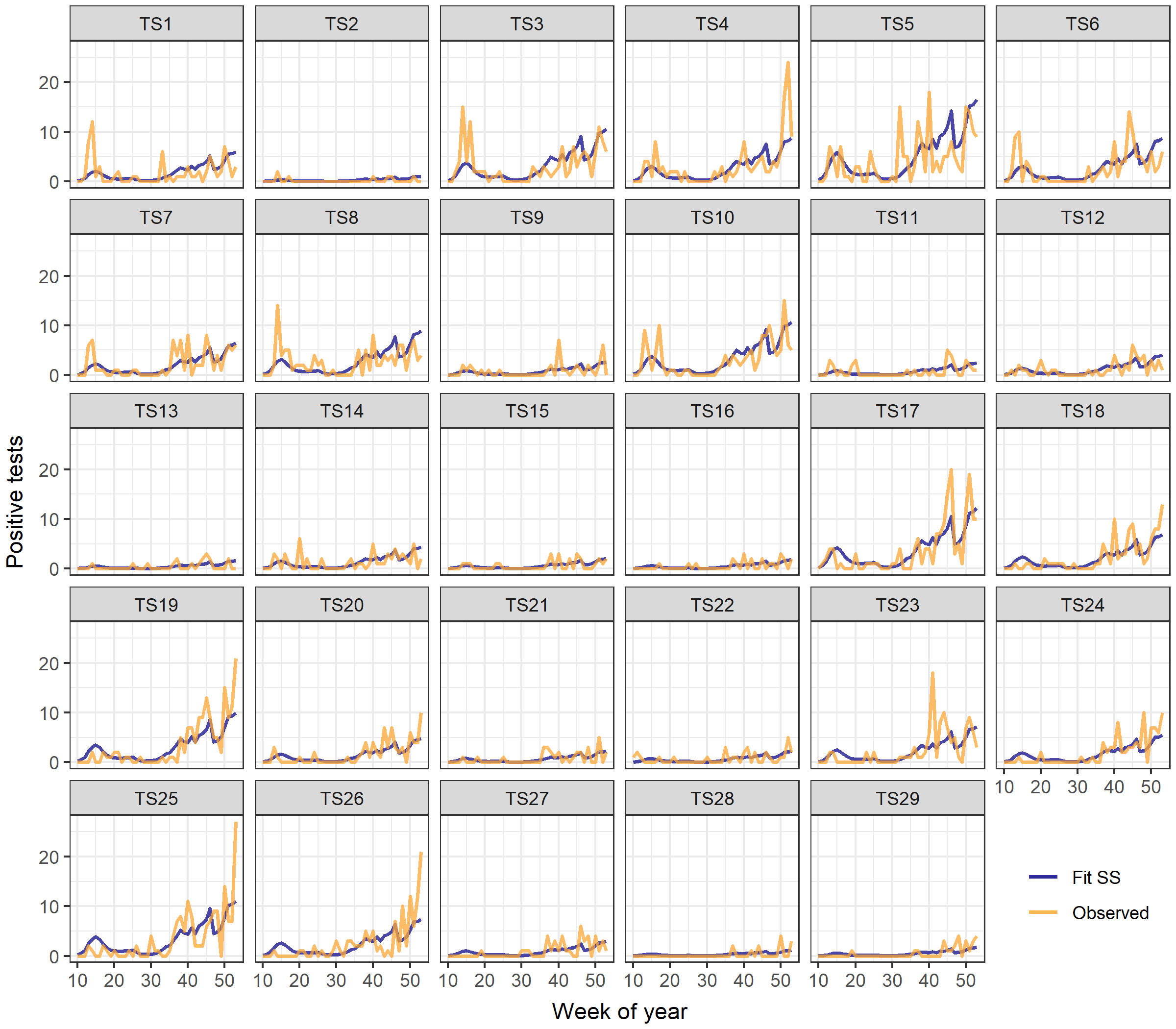

Supplement: Supplementary file: main dataset and code (compressed) [file EMS198536-supplement-Supplementary_file__main_dataset_and_code__compressed_.zip › Covid-19-Teesside-main/Figures/GLMM/All-cases-GLMM-F_Obs-vs-fit_SS.png]

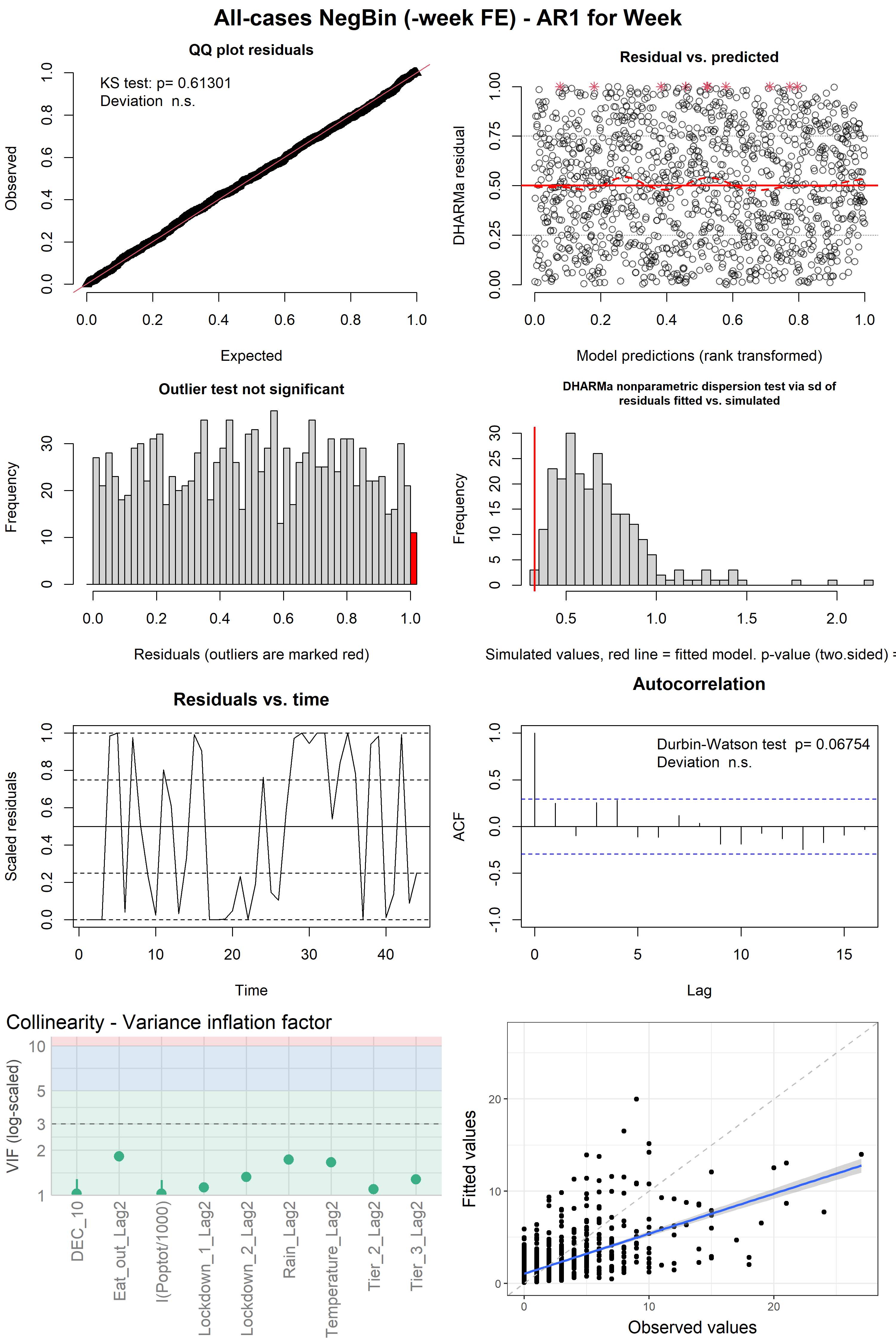

Supplement: Supplementary file: main dataset and code (compressed) [file EMS198536-supplement-Supplementary_file__main_dataset_and_code__compressed_.zip › Covid-19-Teesside-main/Figures/GLMM/All-cases_NB_Ar1-Week_No-Week-FE_Fit.png]

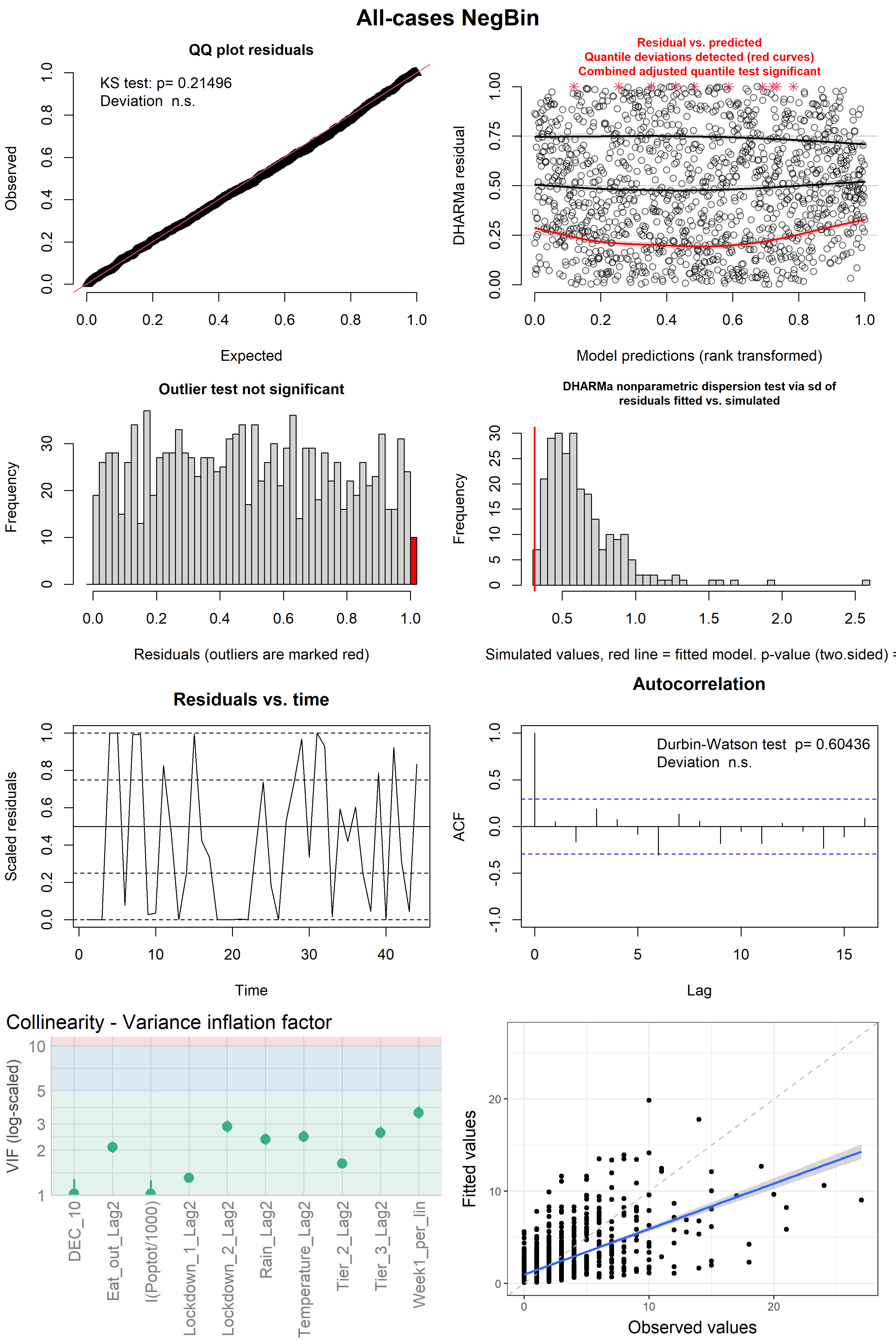

Supplement: Supplementary file: main dataset and code (compressed) [file EMS198536-supplement-Supplementary_file__main_dataset_and_code__compressed_.zip › Covid-19-Teesside-main/Figures/GLMM/All-cases_NB_Full_Fit.png]

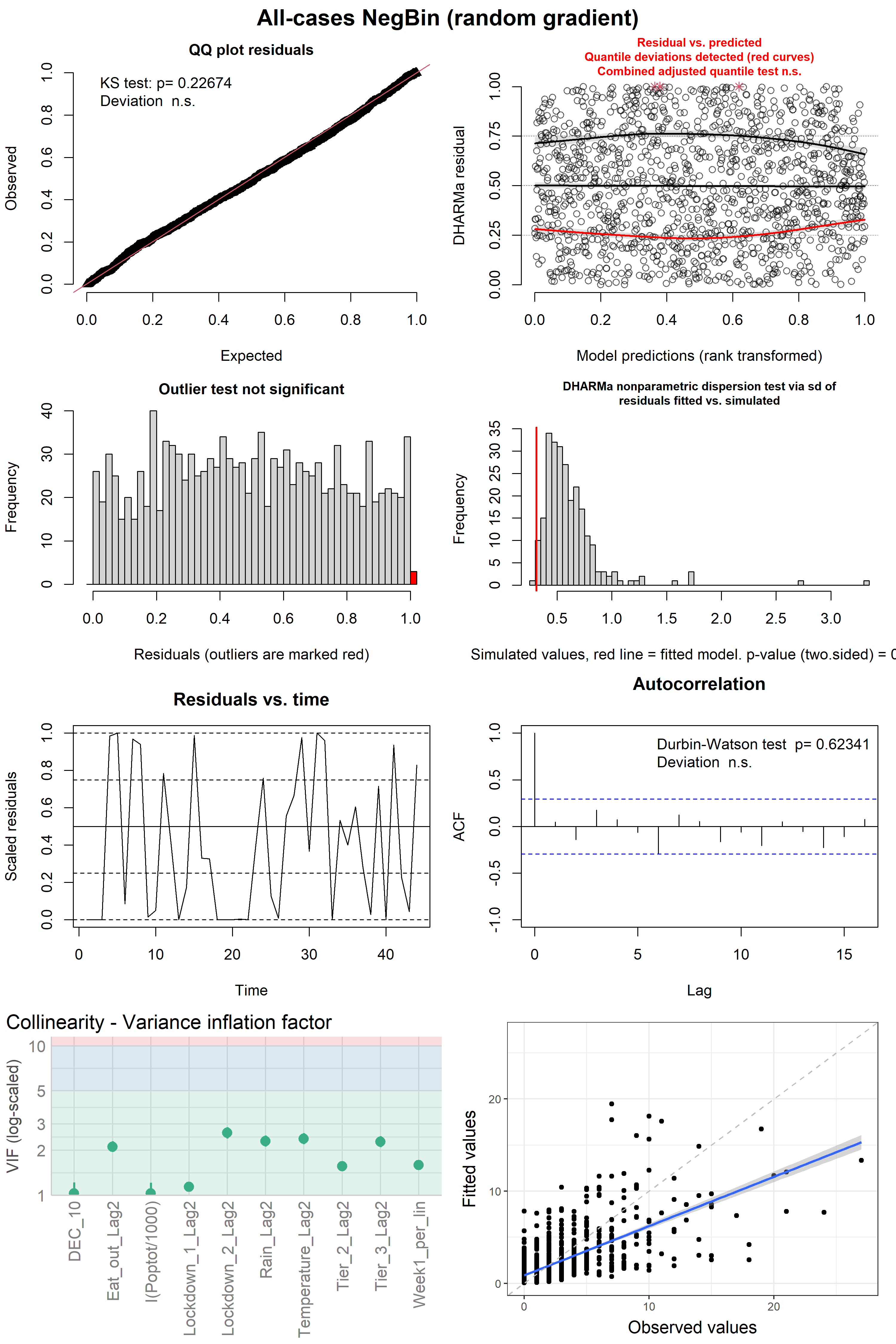

Supplement: Supplementary file: main dataset and code (compressed) [file EMS198536-supplement-Supplementary_file__main_dataset_and_code__compressed_.zip › Covid-19-Teesside-main/Figures/GLMM/All-cases_NB_Gradient-Full_Fit.png]

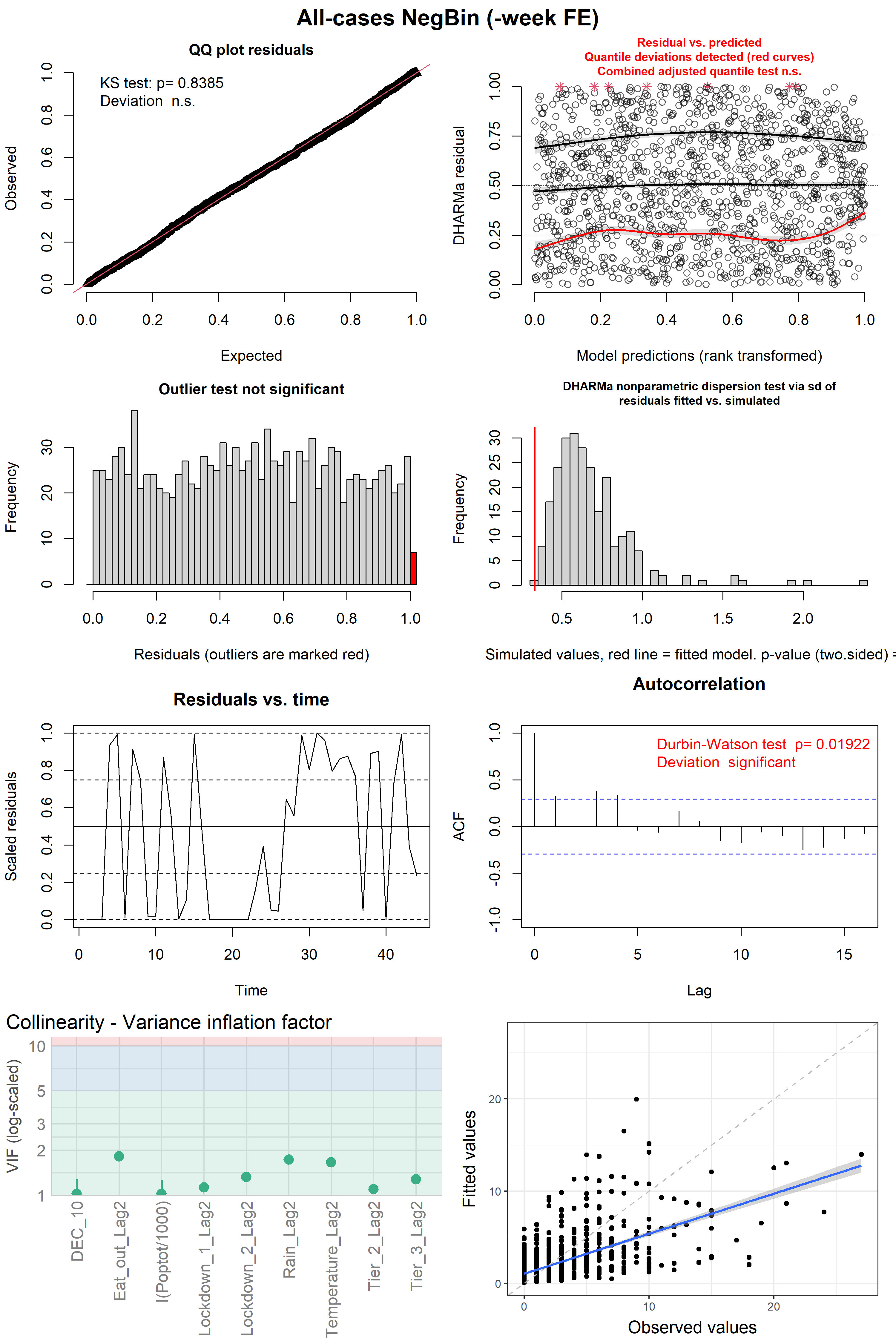

Supplement: Supplementary file: main dataset and code (compressed) [file EMS198536-supplement-Supplementary_file__main_dataset_and_code__compressed_.zip › Covid-19-Teesside-main/Figures/GLMM/All-cases_NB_No-Week-FE_Fit.png]

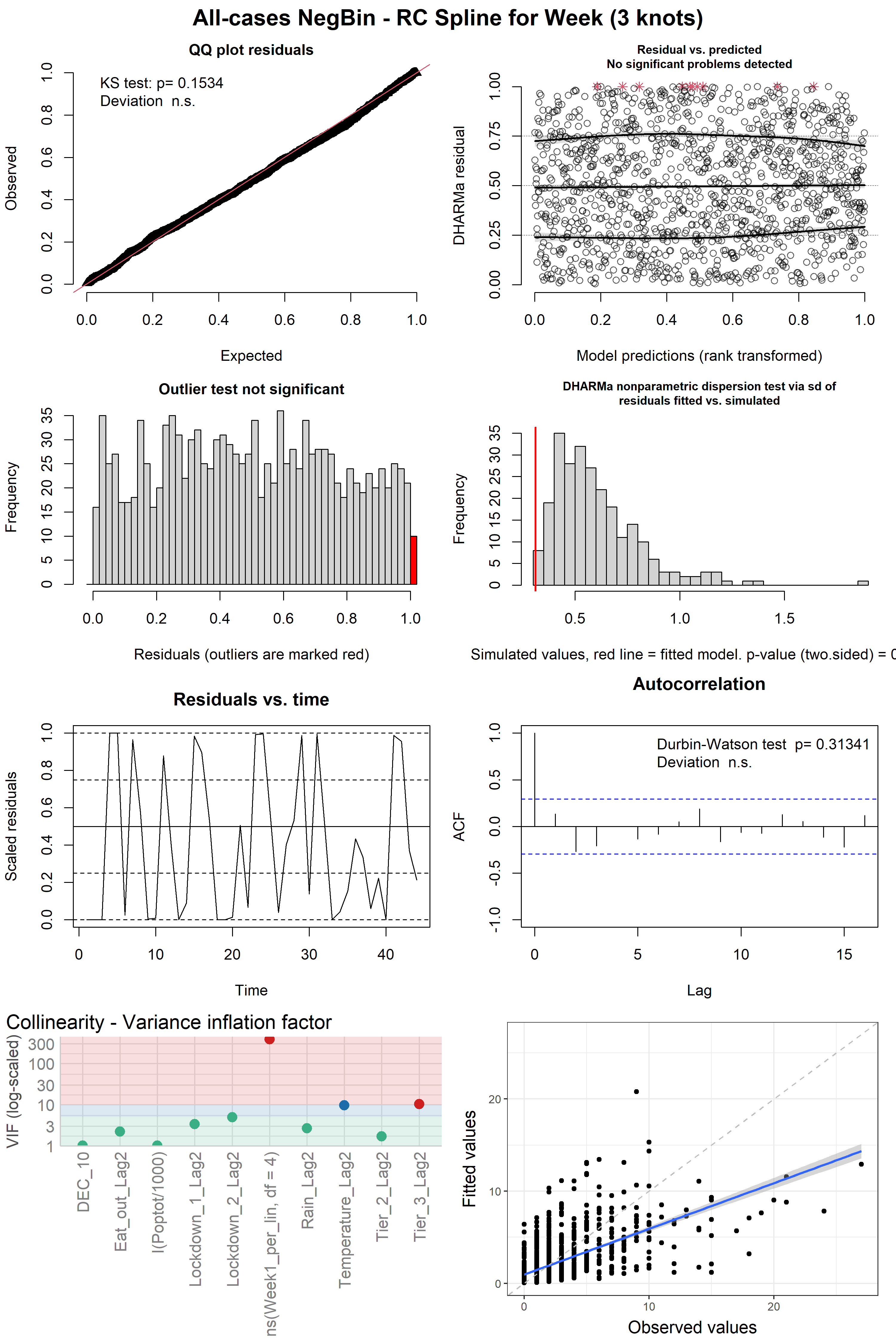

Supplement: Supplementary file: main dataset and code (compressed) [file EMS198536-supplement-Supplementary_file__main_dataset_and_code__compressed_.zip › Covid-19-Teesside-main/Figures/GLMM/All-cases_NB_RCS-Week-3knots_Fit.png]

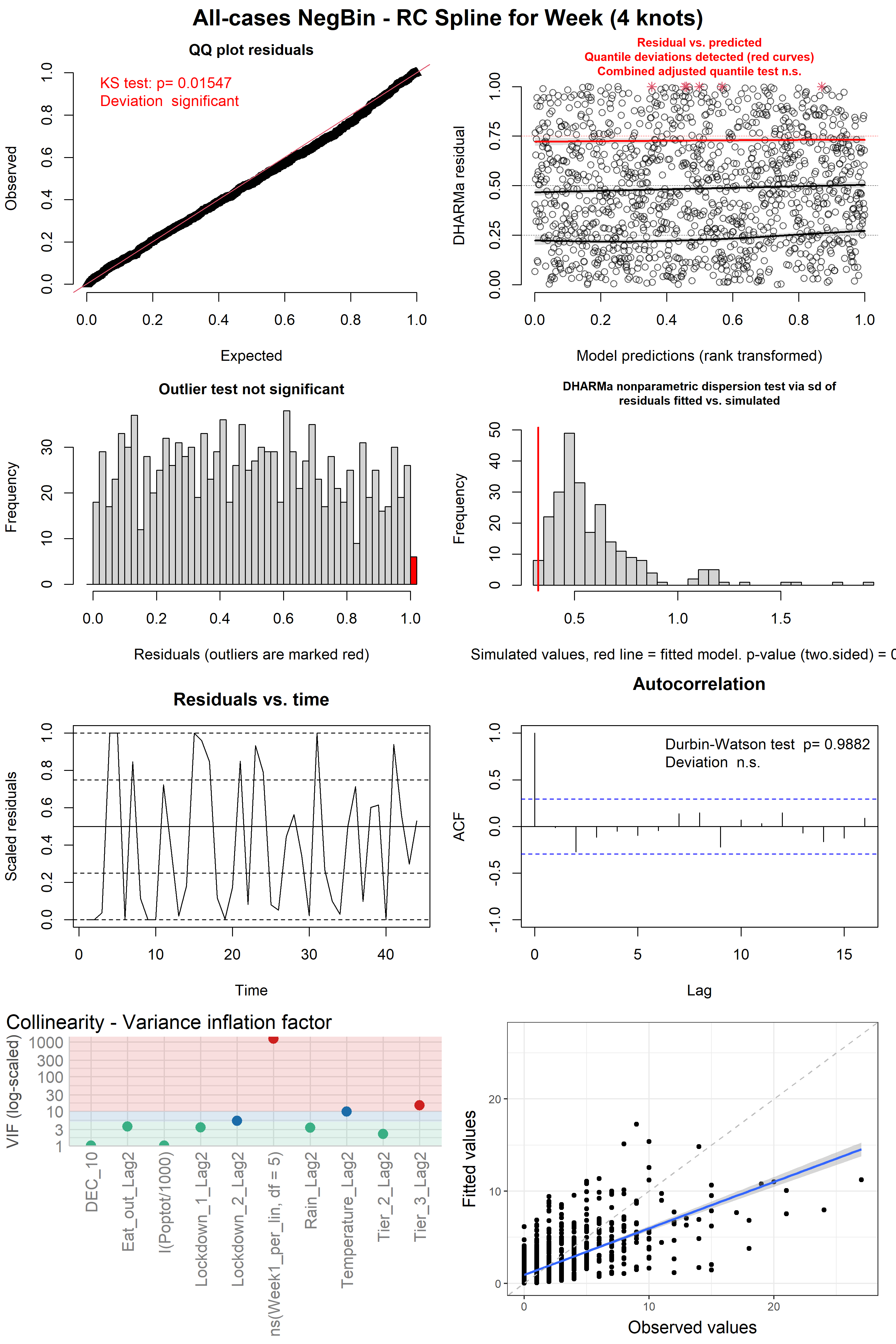

Supplement: Supplementary file: main dataset and code (compressed) [file EMS198536-supplement-Supplementary_file__main_dataset_and_code__compressed_.zip › Covid-19-Teesside-main/Figures/GLMM/All-cases_NB_RCS-Week-4knots_Fit.png]

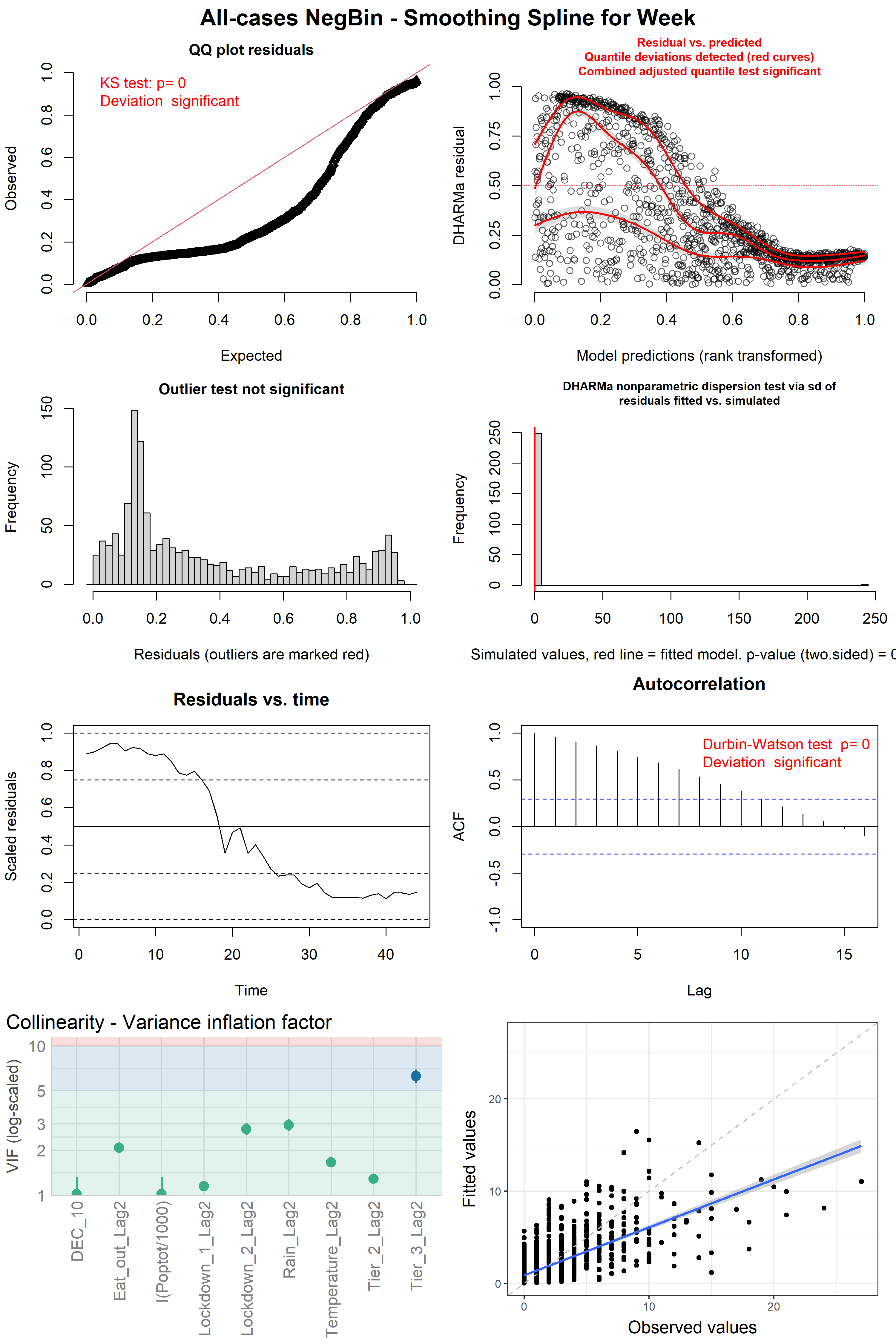

Supplement: Supplementary file: main dataset and code (compressed) [file EMS198536-supplement-Supplementary_file__main_dataset_and_code__compressed_.zip › Covid-19-Teesside-main/Figures/GLMM/All-cases_NB_SmoothSpline-Week-TPS_Fit.png]

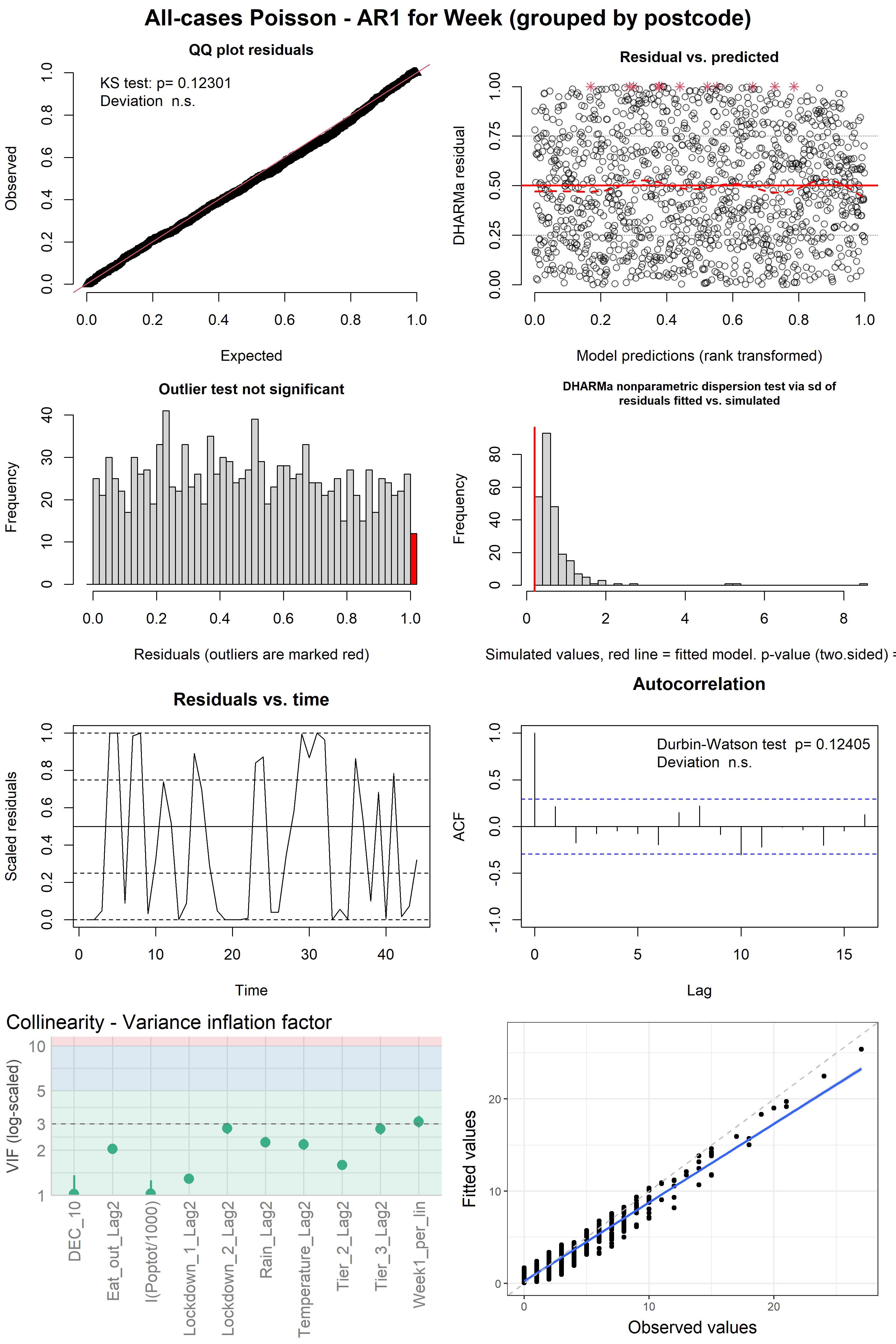

Supplement: Supplementary file: main dataset and code (compressed) [file EMS198536-supplement-Supplementary_file__main_dataset_and_code__compressed_.zip › Covid-19-Teesside-main/Figures/GLMM/All-cases_Po_Ar1-Week-Postcode_Fit.png]

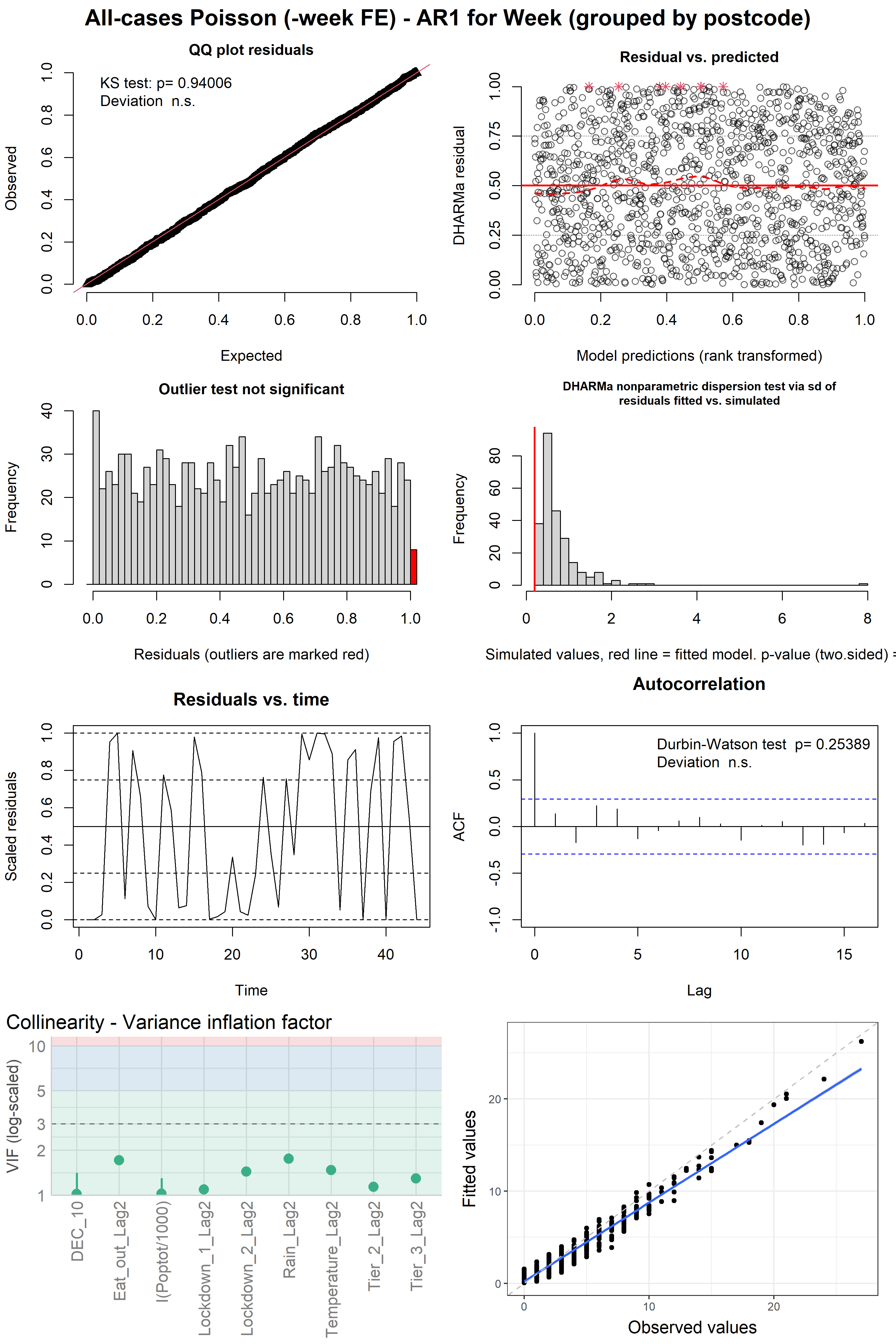

Supplement: Supplementary file: main dataset and code (compressed) [file EMS198536-supplement-Supplementary_file__main_dataset_and_code__compressed_.zip › Covid-19-Teesside-main/Figures/GLMM/All-cases_Po_Ar1-Week-Postcode_No-Week-FE_Fit.png]

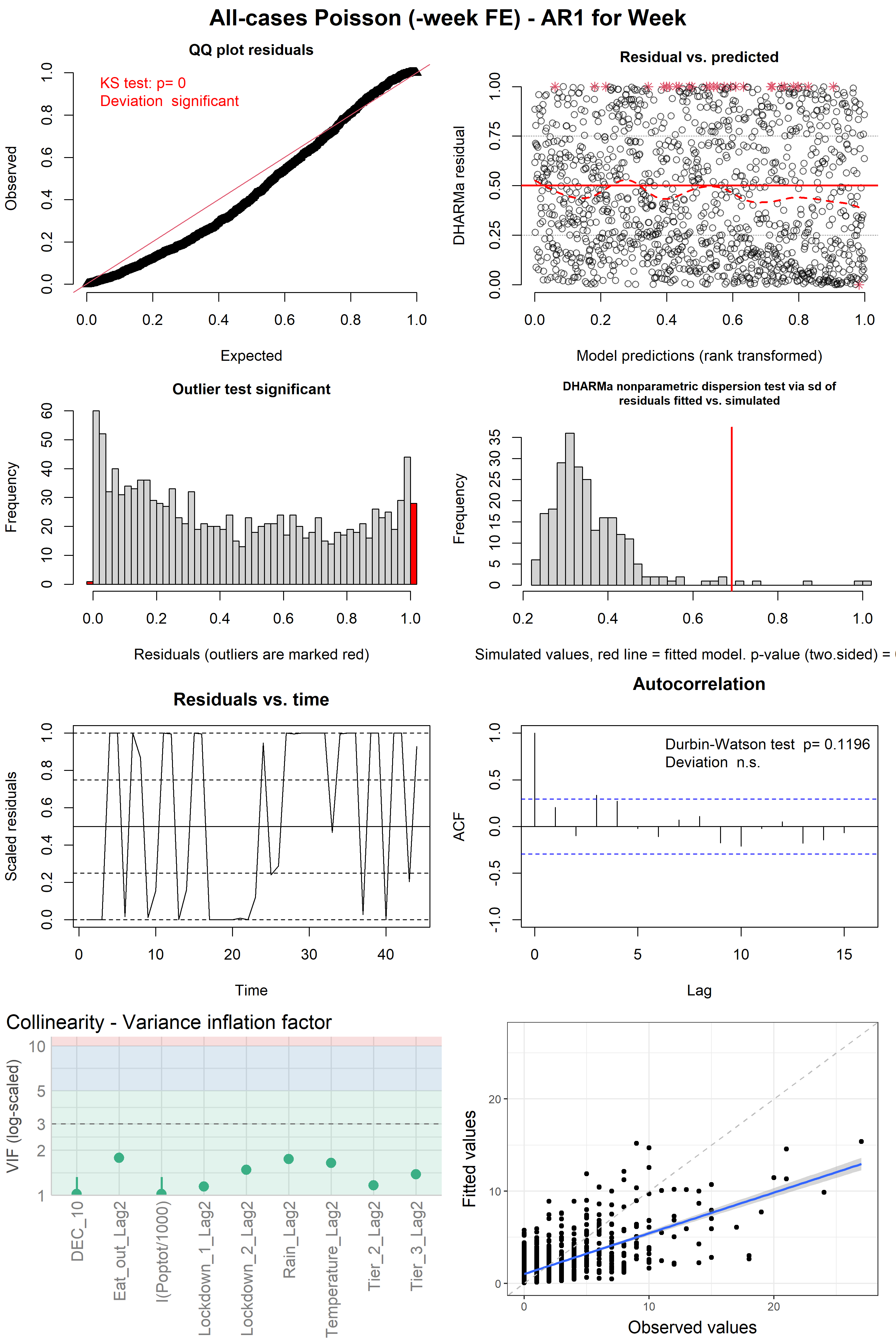

Supplement: Supplementary file: main dataset and code (compressed) [file EMS198536-supplement-Supplementary_file__main_dataset_and_code__compressed_.zip › Covid-19-Teesside-main/Figures/GLMM/All-cases_Po_Ar1-Week_No-Week-FE_Fit.png]

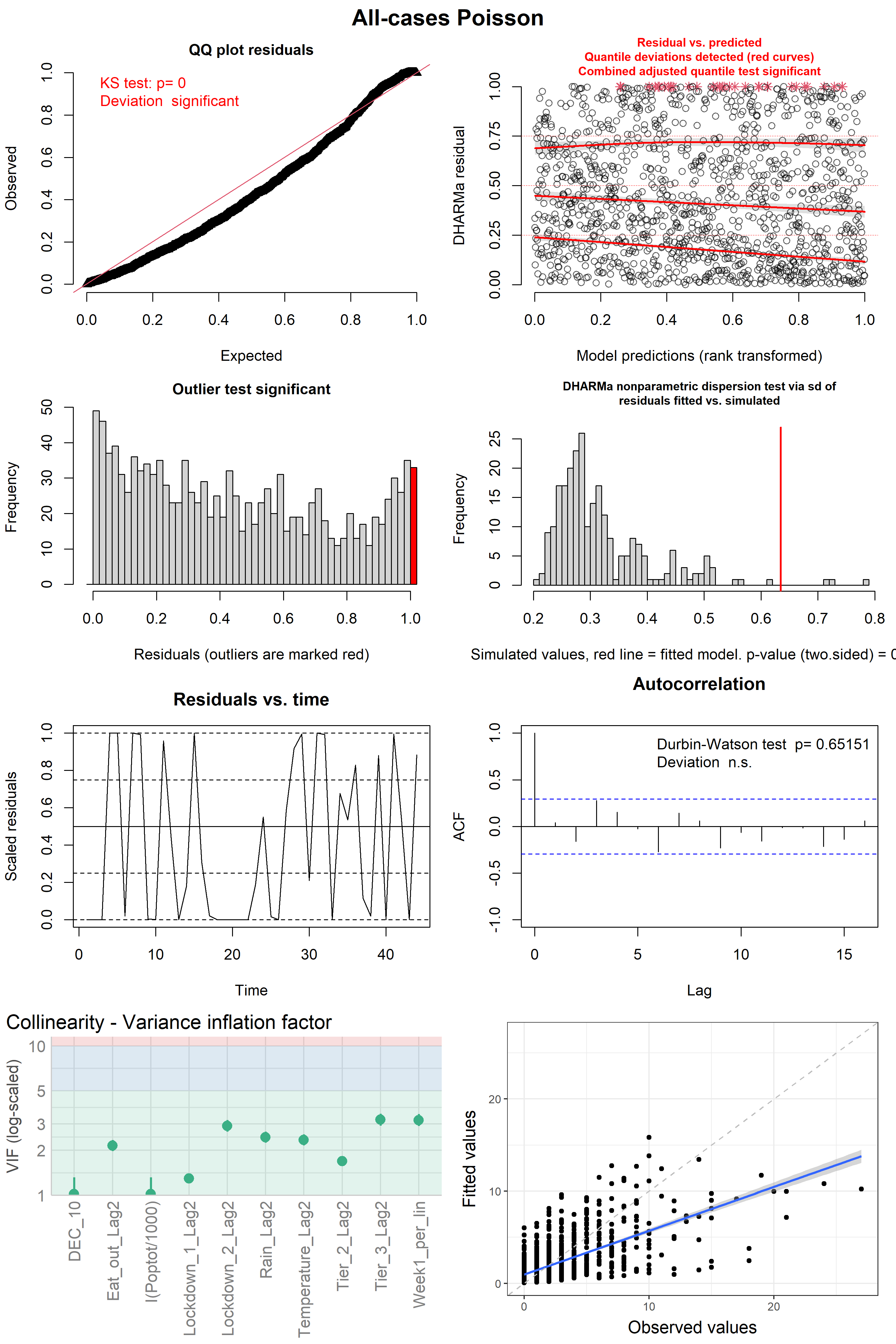

Supplement: Supplementary file: main dataset and code (compressed) [file EMS198536-supplement-Supplementary_file__main_dataset_and_code__compressed_.zip › Covid-19-Teesside-main/Figures/GLMM/All-cases_Po_Full_Fit.png]

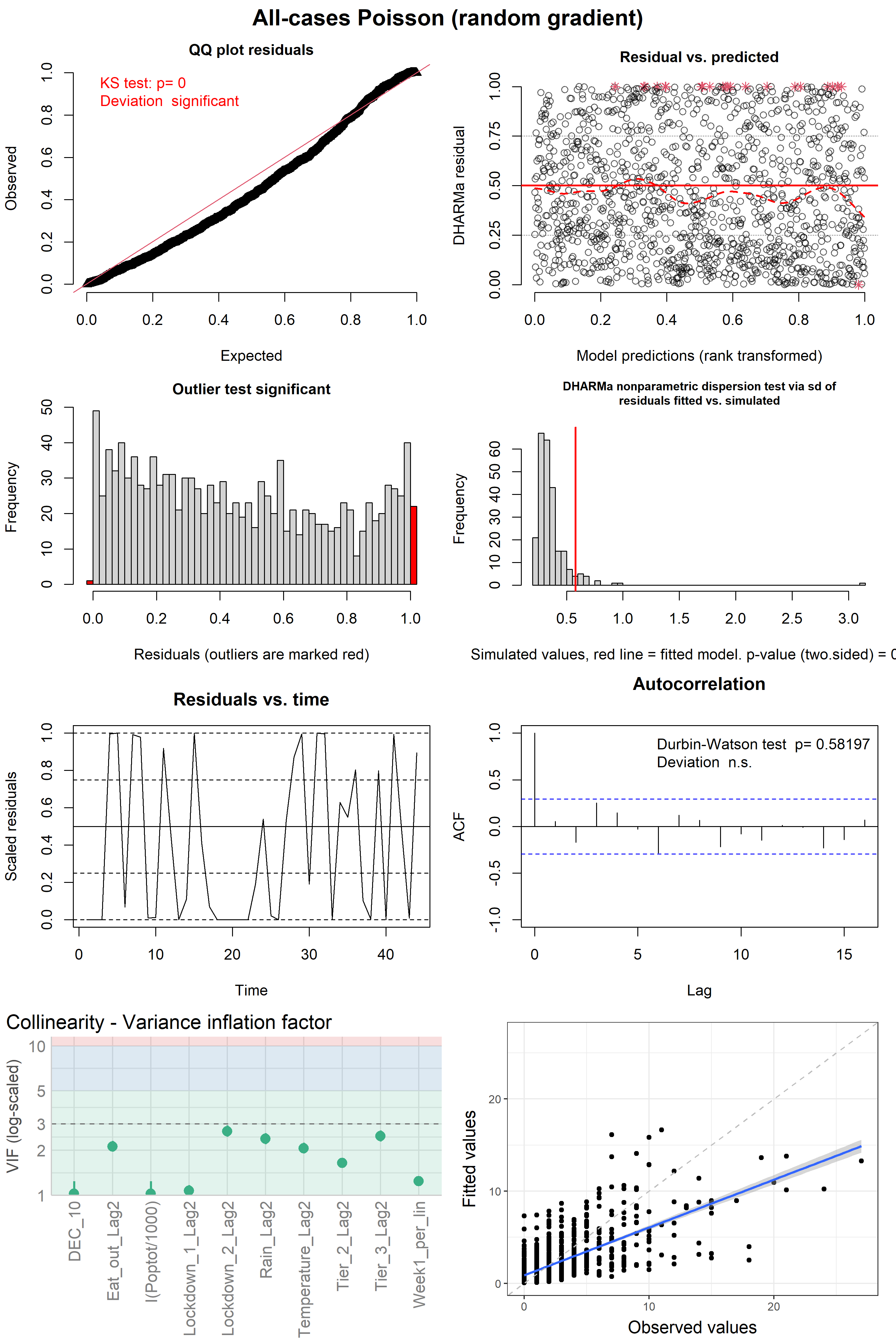

Supplement: Supplementary file: main dataset and code (compressed) [file EMS198536-supplement-Supplementary_file__main_dataset_and_code__compressed_.zip › Covid-19-Teesside-main/Figures/GLMM/All-cases_Po_Gradient-Full_Fit.png]

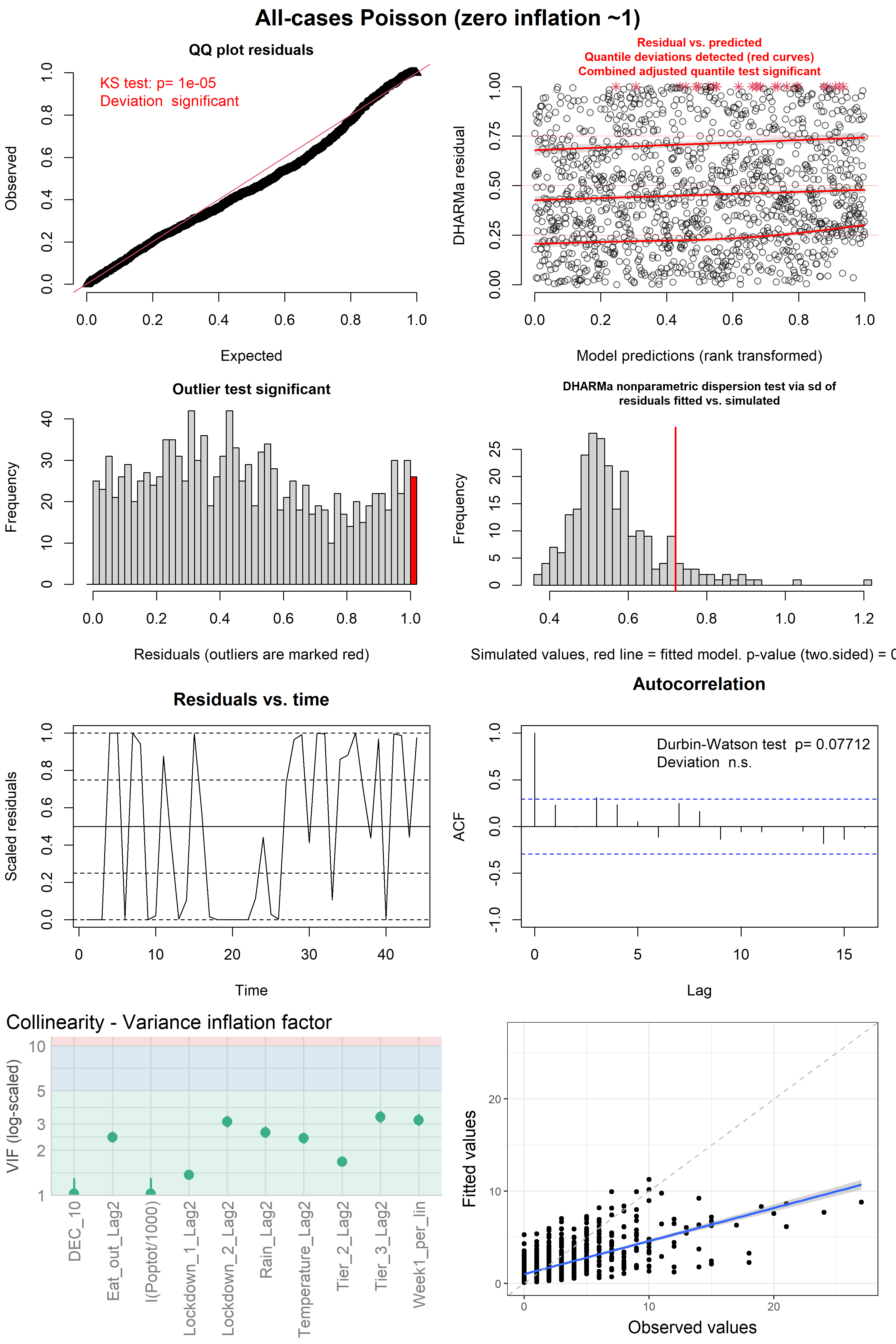

Supplement: Supplementary file: main dataset and code (compressed) [file EMS198536-supplement-Supplementary_file__main_dataset_and_code__compressed_.zip › Covid-19-Teesside-main/Figures/GLMM/All-cases_Po_ZI1-Full_Fit.png]

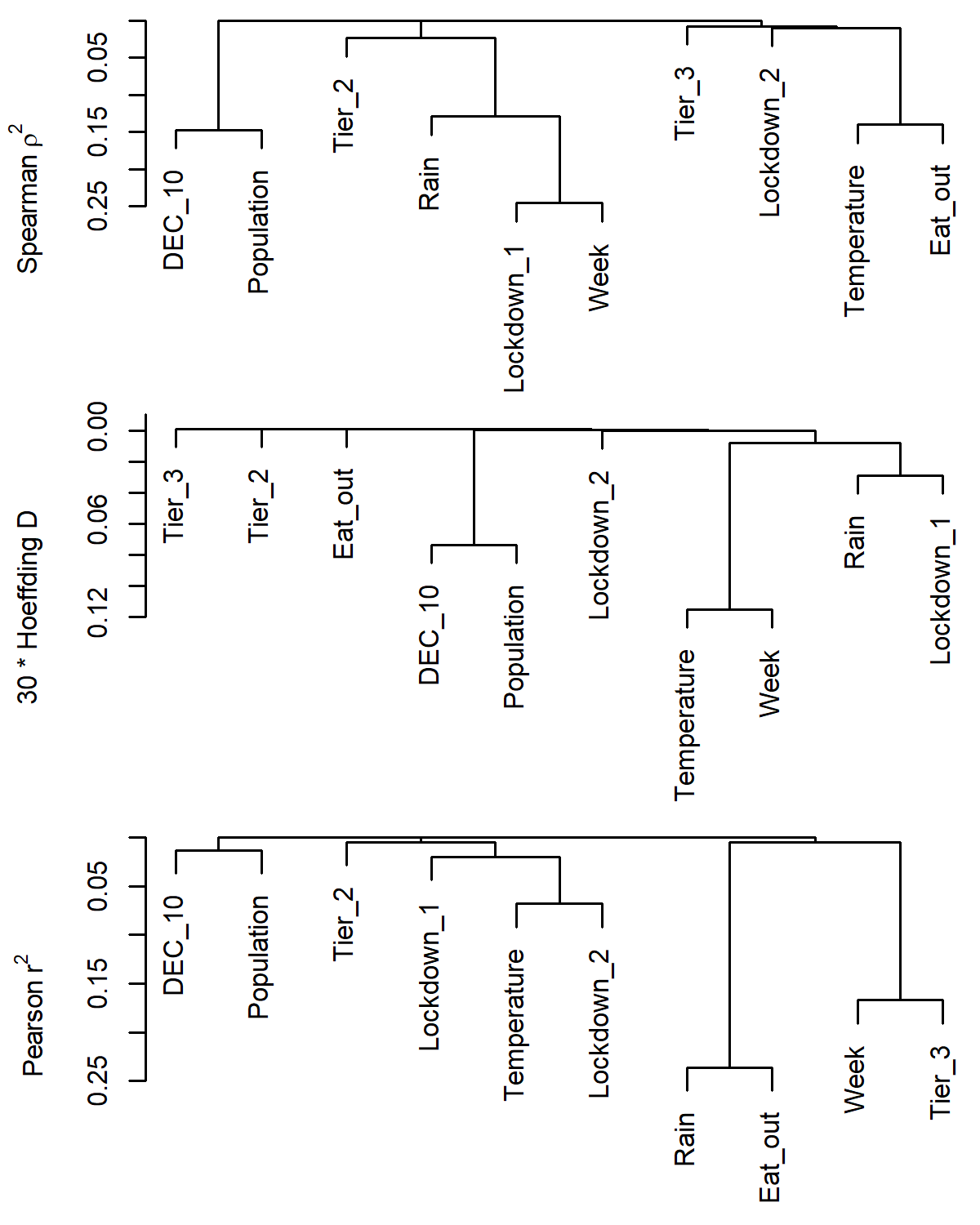

Supplement: Supplementary file: main dataset and code (compressed) [file EMS198536-supplement-Supplementary_file__main_dataset_and_code__compressed_.zip › Covid-19-Teesside-main/Figures/GLMM/All-cases_Variable-Clustering.png]

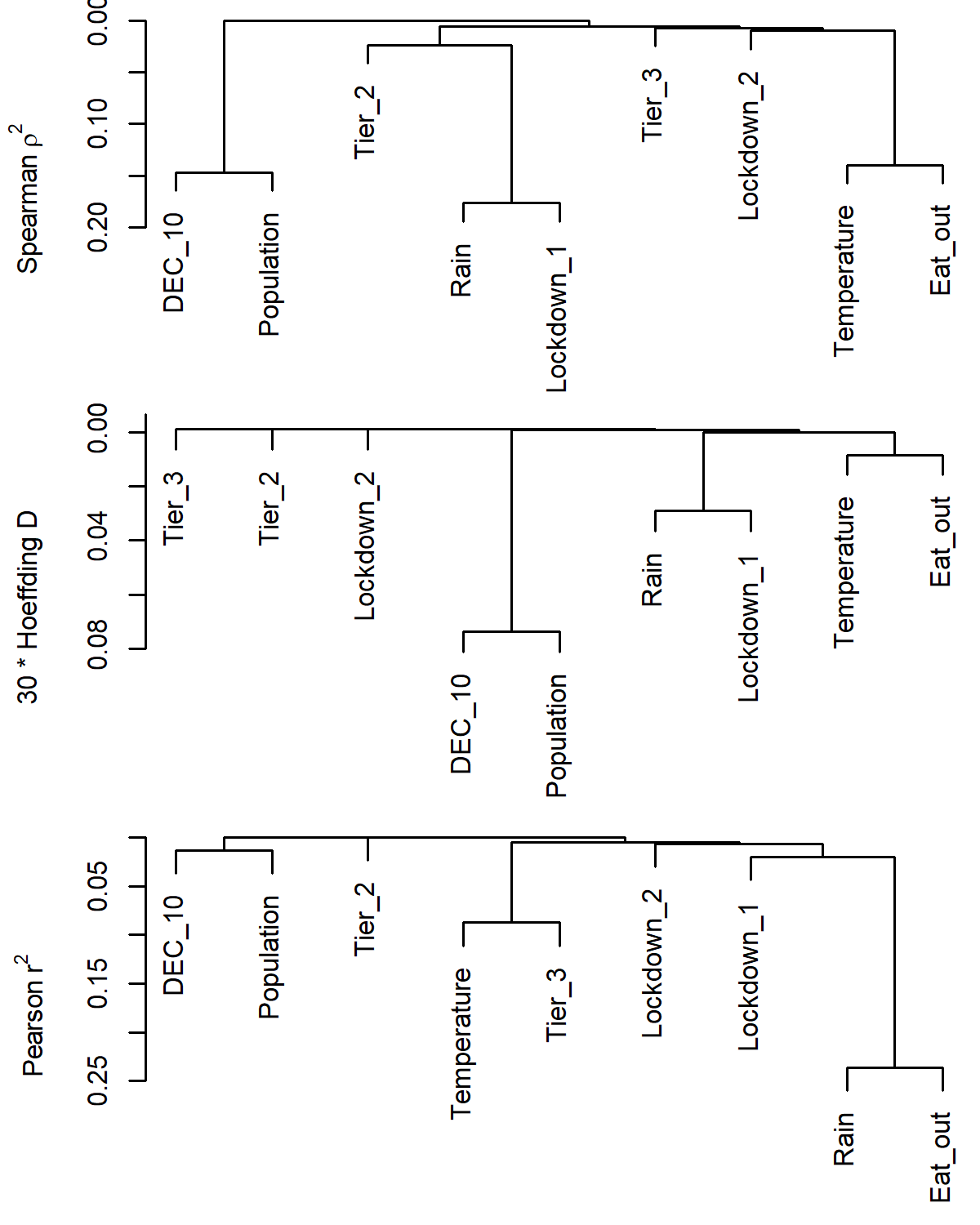

Supplement: Supplementary file: main dataset and code (compressed) [file EMS198536-supplement-Supplementary_file__main_dataset_and_code__compressed_.zip › Covid-19-Teesside-main/Figures/GLMM/All-cases_Variable-Clustering_Without-week.png]

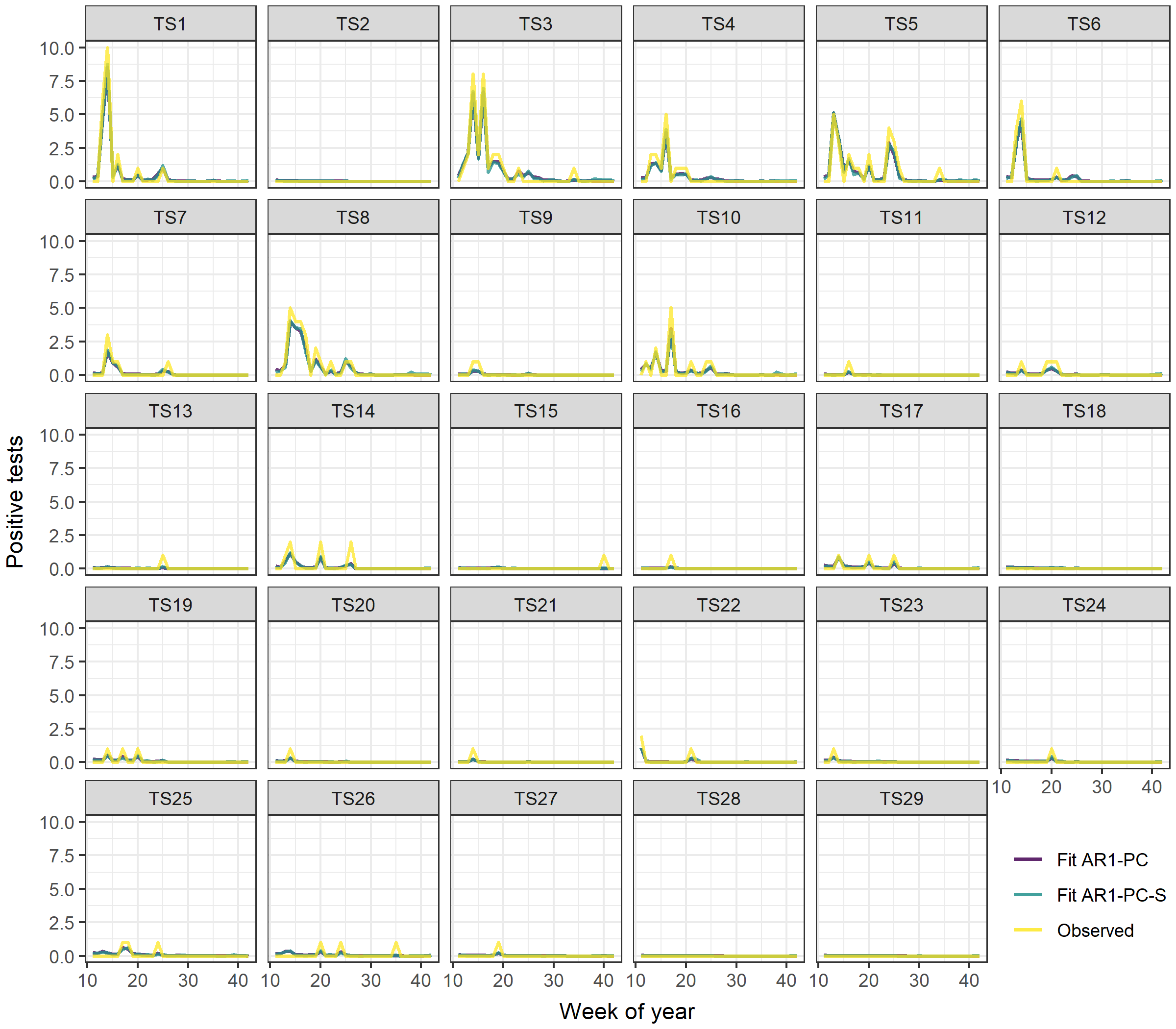

Supplement: Supplementary file: main dataset and code (compressed) [file EMS198536-supplement-Supplementary_file__main_dataset_and_code__compressed_.zip › Covid-19-Teesside-main/Figures/GLMM/Lin10/Lin10-B11119_GLMM_Obs-vs-Fit_AR1PC-AR1PCS1.png]

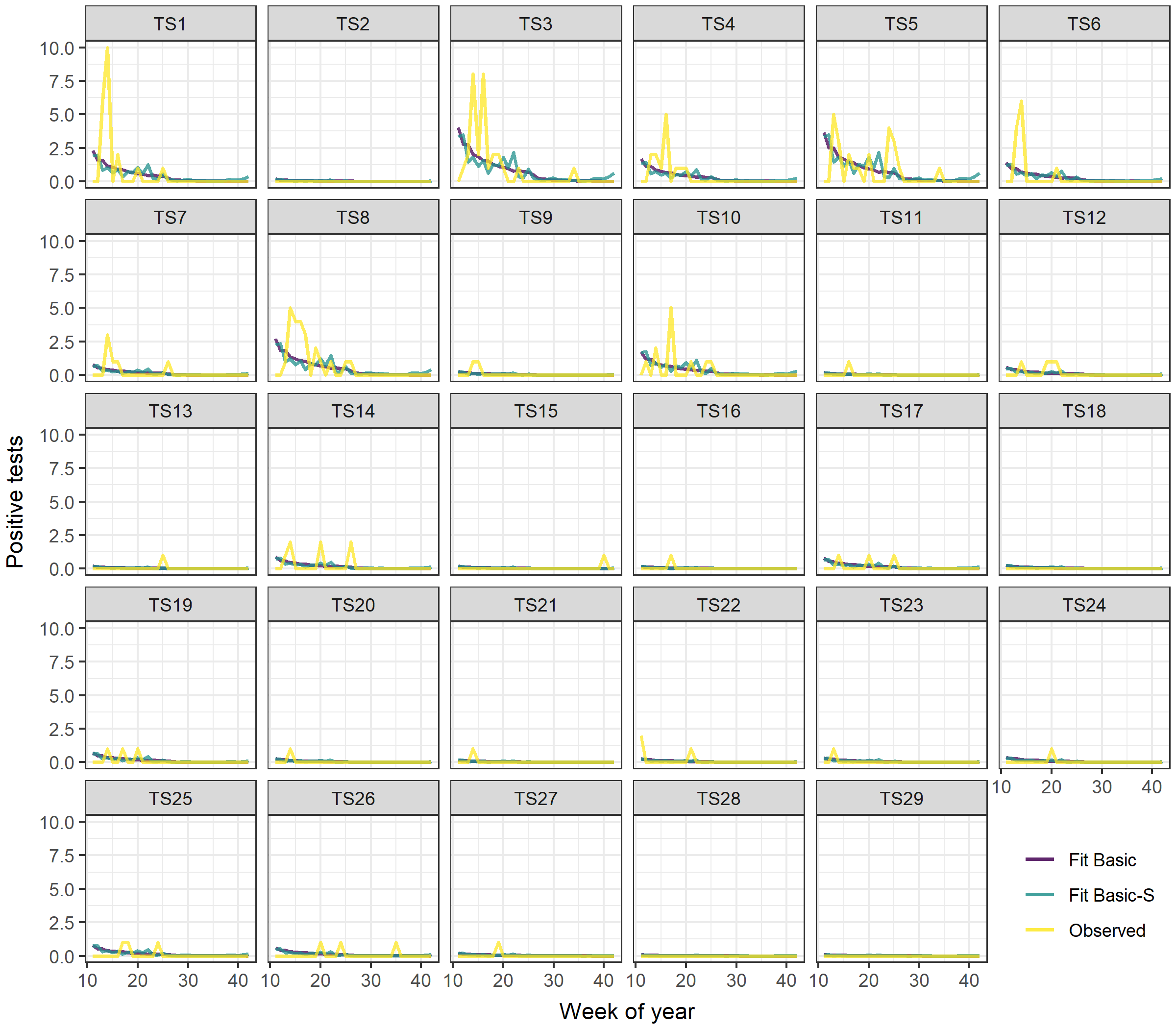

Supplement: Supplementary file: main dataset and code (compressed) [file EMS198536-supplement-Supplementary_file__main_dataset_and_code__compressed_.zip › Covid-19-Teesside-main/Figures/GLMM/Lin10/Lin10-B11119_GLMM_Obs-vs-Fit_Basic-BasicS1.png]

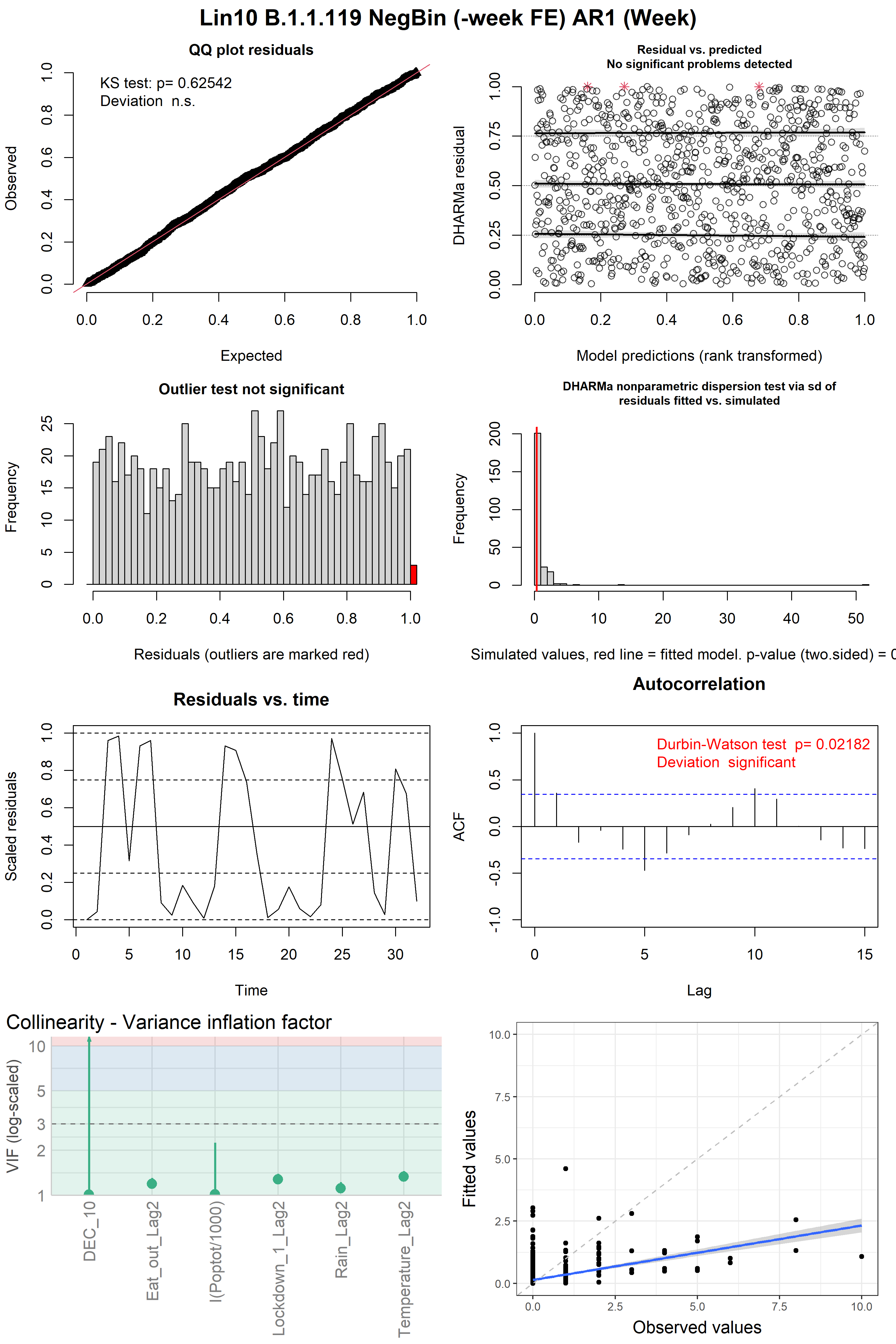

Supplement: Supplementary file: main dataset and code (compressed) [file EMS198536-supplement-Supplementary_file__main_dataset_and_code__compressed_.zip › Covid-19-Teesside-main/Figures/GLMM/Lin10/Lin10-B11119_NB_AR1-Week_No-week-FE_Fit.png]

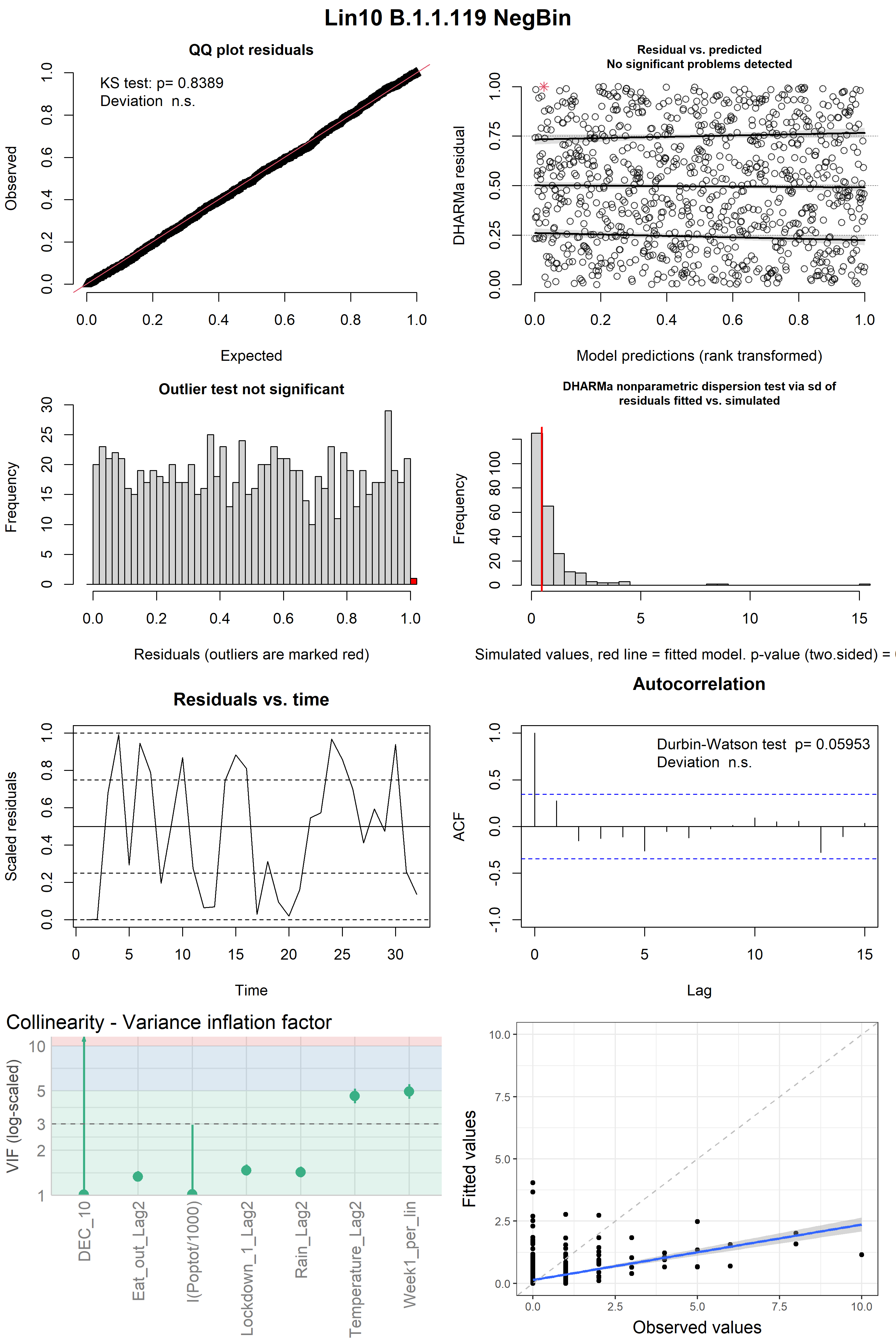

Supplement: Supplementary file: main dataset and code (compressed) [file EMS198536-supplement-Supplementary_file__main_dataset_and_code__compressed_.zip › Covid-19-Teesside-main/Figures/GLMM/Lin10/Lin10-B11119_NB_Full_Fit.png]

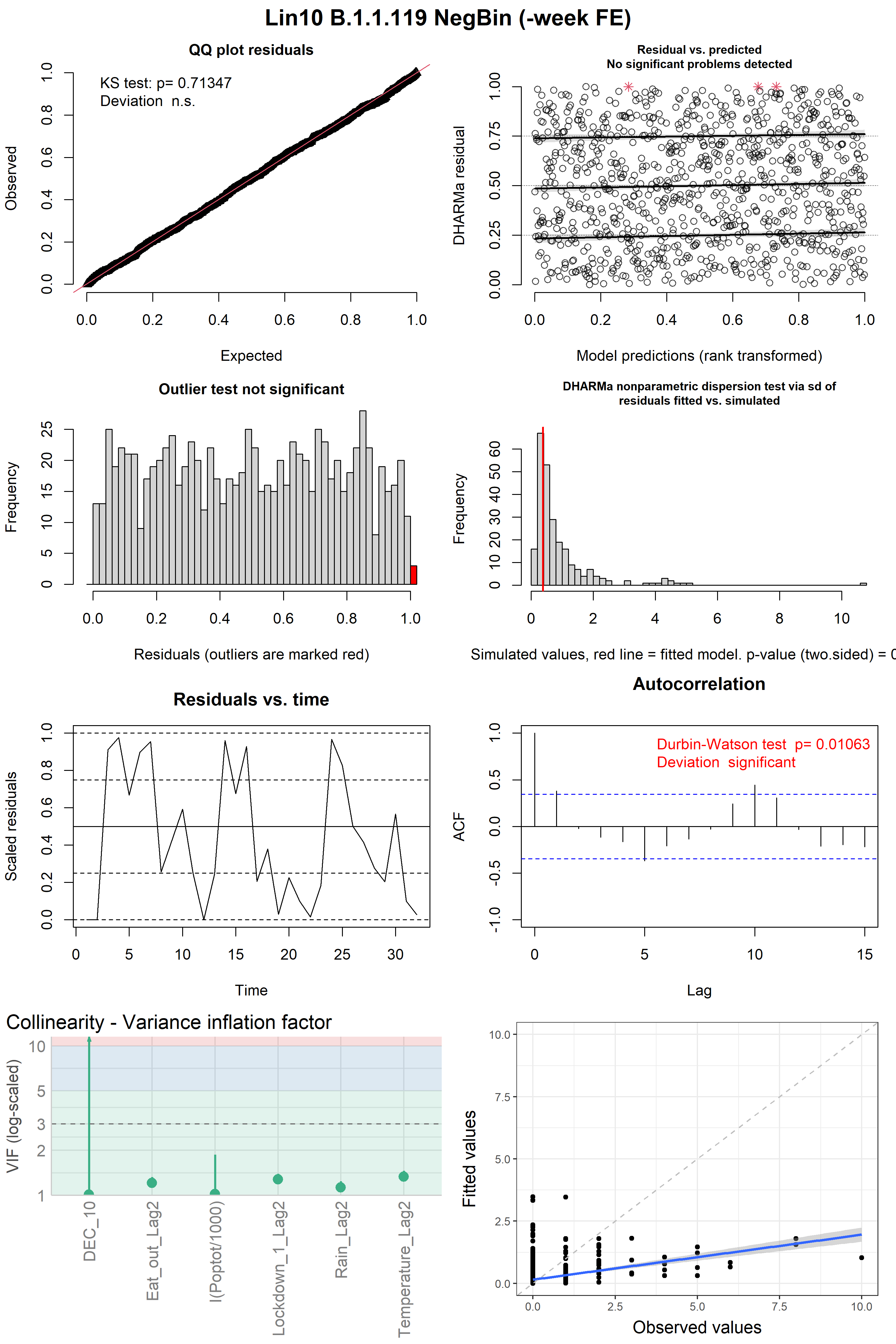

Supplement: Supplementary file: main dataset and code (compressed) [file EMS198536-supplement-Supplementary_file__main_dataset_and_code__compressed_.zip › Covid-19-Teesside-main/Figures/GLMM/Lin10/Lin10-B11119_NB_No-week-FE_Fit.png]

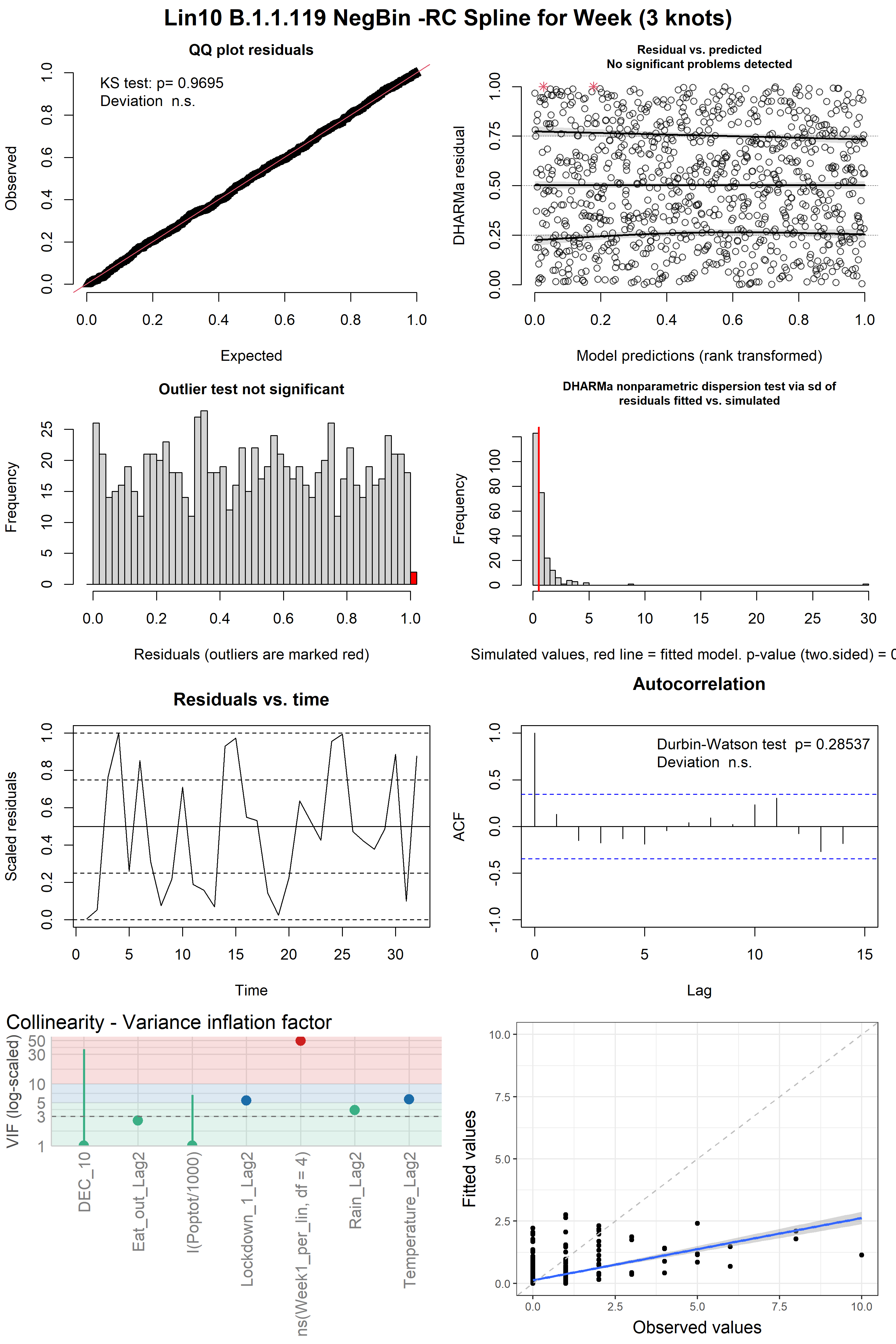

Supplement: Supplementary file: main dataset and code (compressed) [file EMS198536-supplement-Supplementary_file__main_dataset_and_code__compressed_.zip › Covid-19-Teesside-main/Figures/GLMM/Lin10/Lin10-B11119_NB_RCS-Week-3knots_Fit.png]

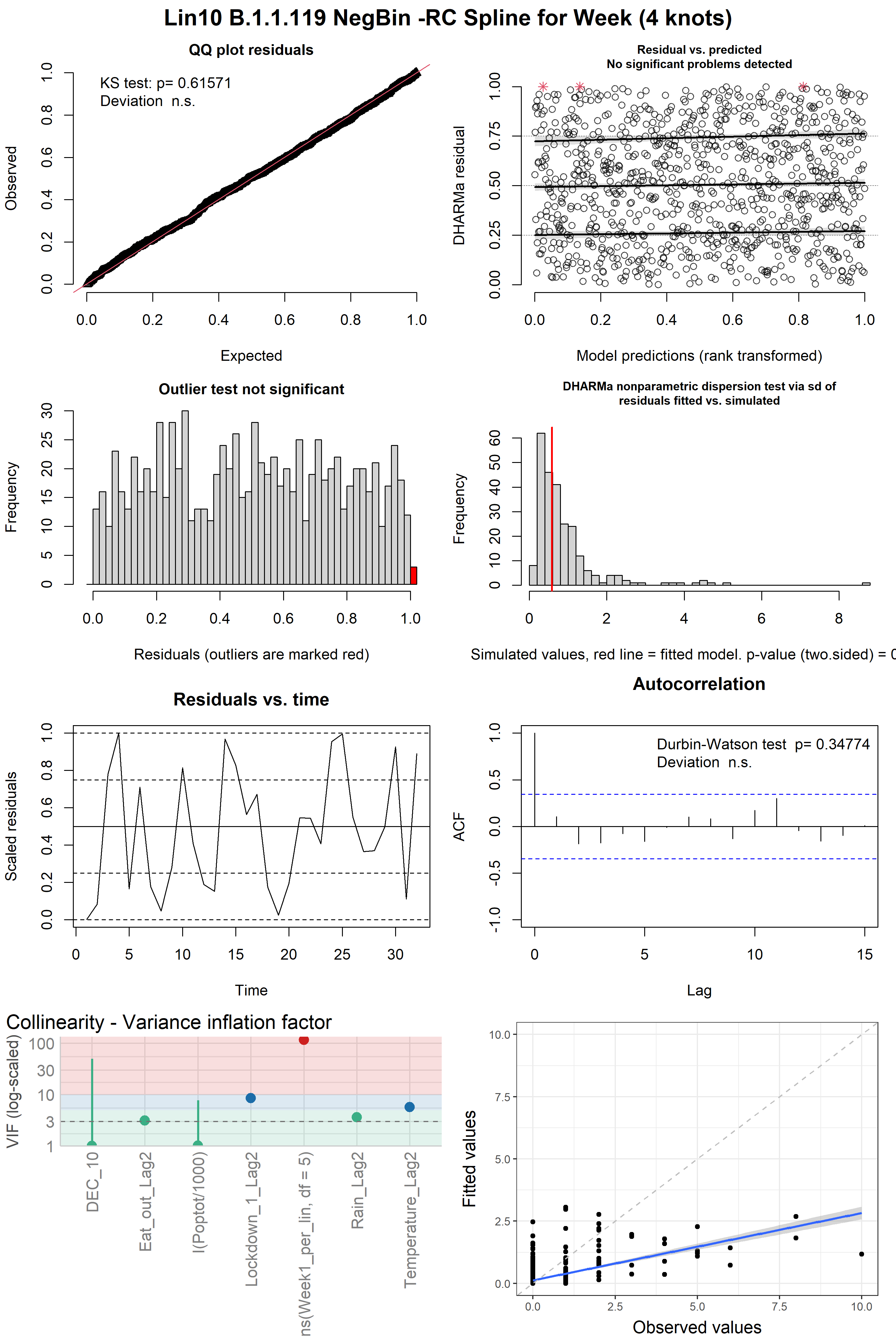

Supplement: Supplementary file: main dataset and code (compressed) [file EMS198536-supplement-Supplementary_file__main_dataset_and_code__compressed_.zip › Covid-19-Teesside-main/Figures/GLMM/Lin10/Lin10-B11119_NB_RCS-Week-4knots_Fit.png]

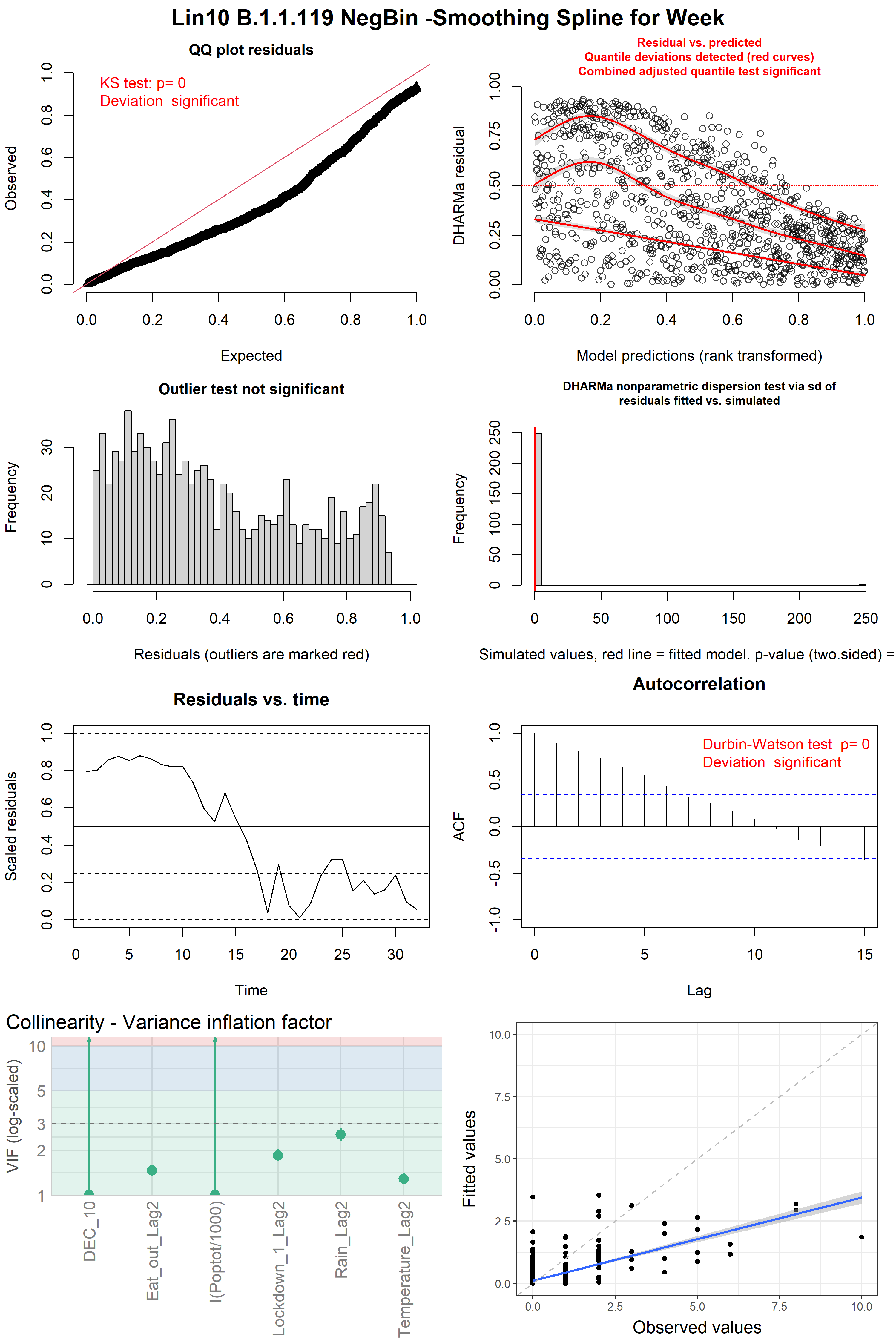

Supplement: Supplementary file: main dataset and code (compressed) [file EMS198536-supplement-Supplementary_file__main_dataset_and_code__compressed_.zip › Covid-19-Teesside-main/Figures/GLMM/Lin10/Lin10-B11119_NB_SmoothSpline-Week-TPS_Fit.png]

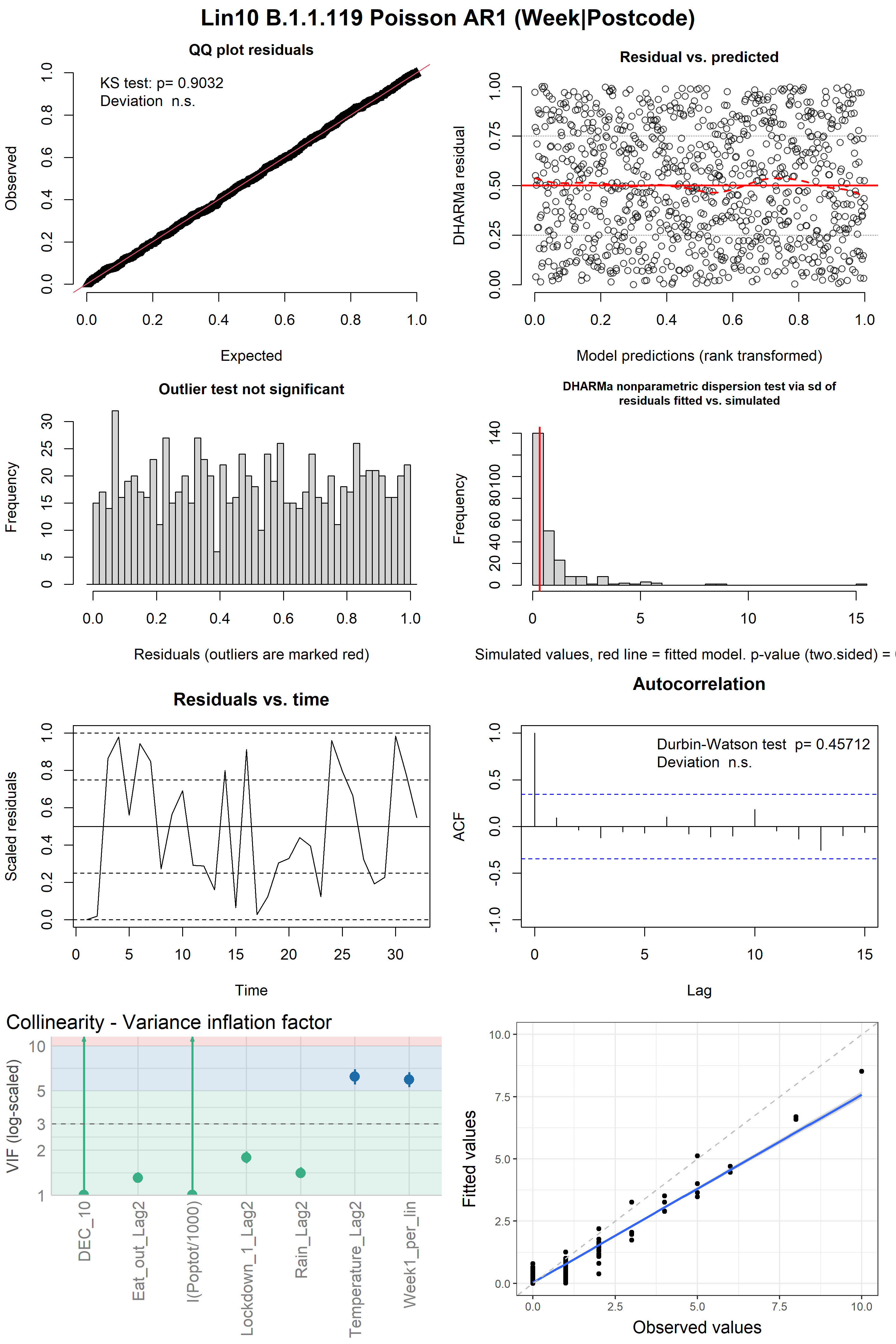

Supplement: Supplementary file: main dataset and code (compressed) [file EMS198536-supplement-Supplementary_file__main_dataset_and_code__compressed_.zip › Covid-19-Teesside-main/Figures/GLMM/Lin10/Lin10-B11119_Po_AR1-Week-Postcode_Fit.png]

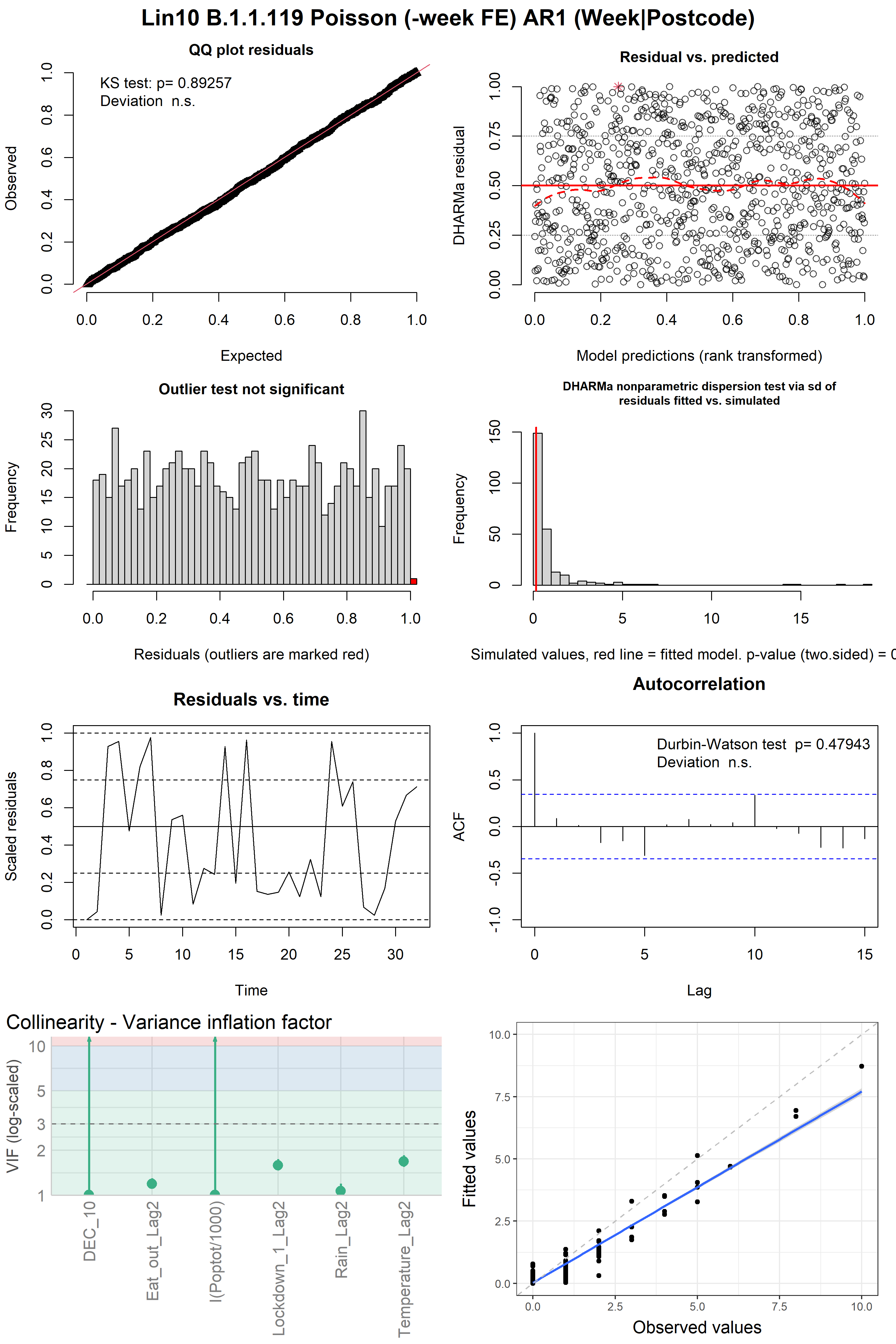

Supplement: Supplementary file: main dataset and code (compressed) [file EMS198536-supplement-Supplementary_file__main_dataset_and_code__compressed_.zip › Covid-19-Teesside-main/Figures/GLMM/Lin10/Lin10-B11119_Po_AR1-Week-Postcode_No-week-FE_Fit.png]

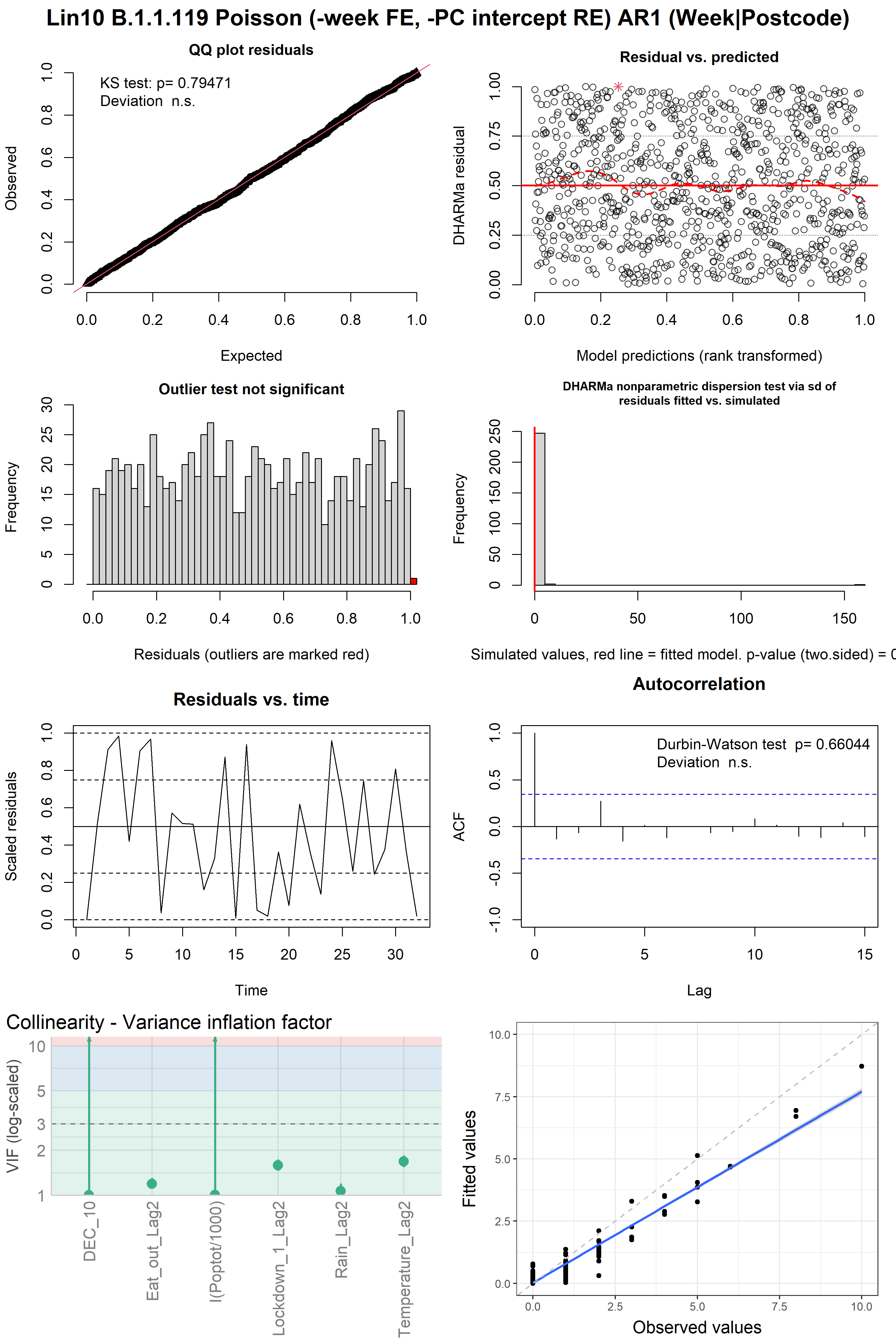

Supplement: Supplementary file: main dataset and code (compressed) [file EMS198536-supplement-Supplementary_file__main_dataset_and_code__compressed_.zip › Covid-19-Teesside-main/Figures/GLMM/Lin10/Lin10-B11119_Po_AR1-Week-Postcode_No-week-FE_No-PC-intercept-RE_Fit.png]

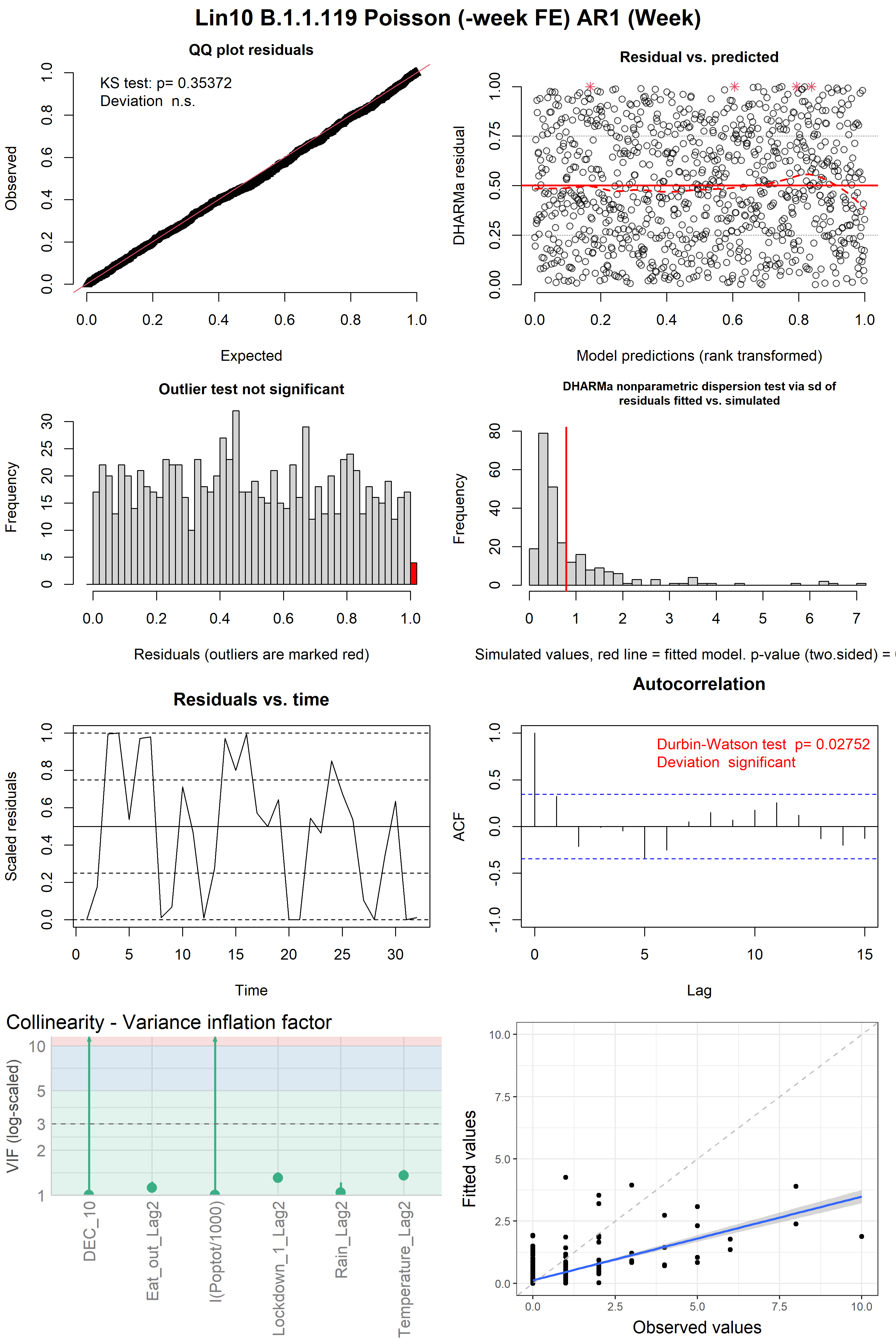

Supplement: Supplementary file: main dataset and code (compressed) [file EMS198536-supplement-Supplementary_file__main_dataset_and_code__compressed_.zip › Covid-19-Teesside-main/Figures/GLMM/Lin10/Lin10-B11119_Po_AR1-Week_No-week-FE_Fit.png]

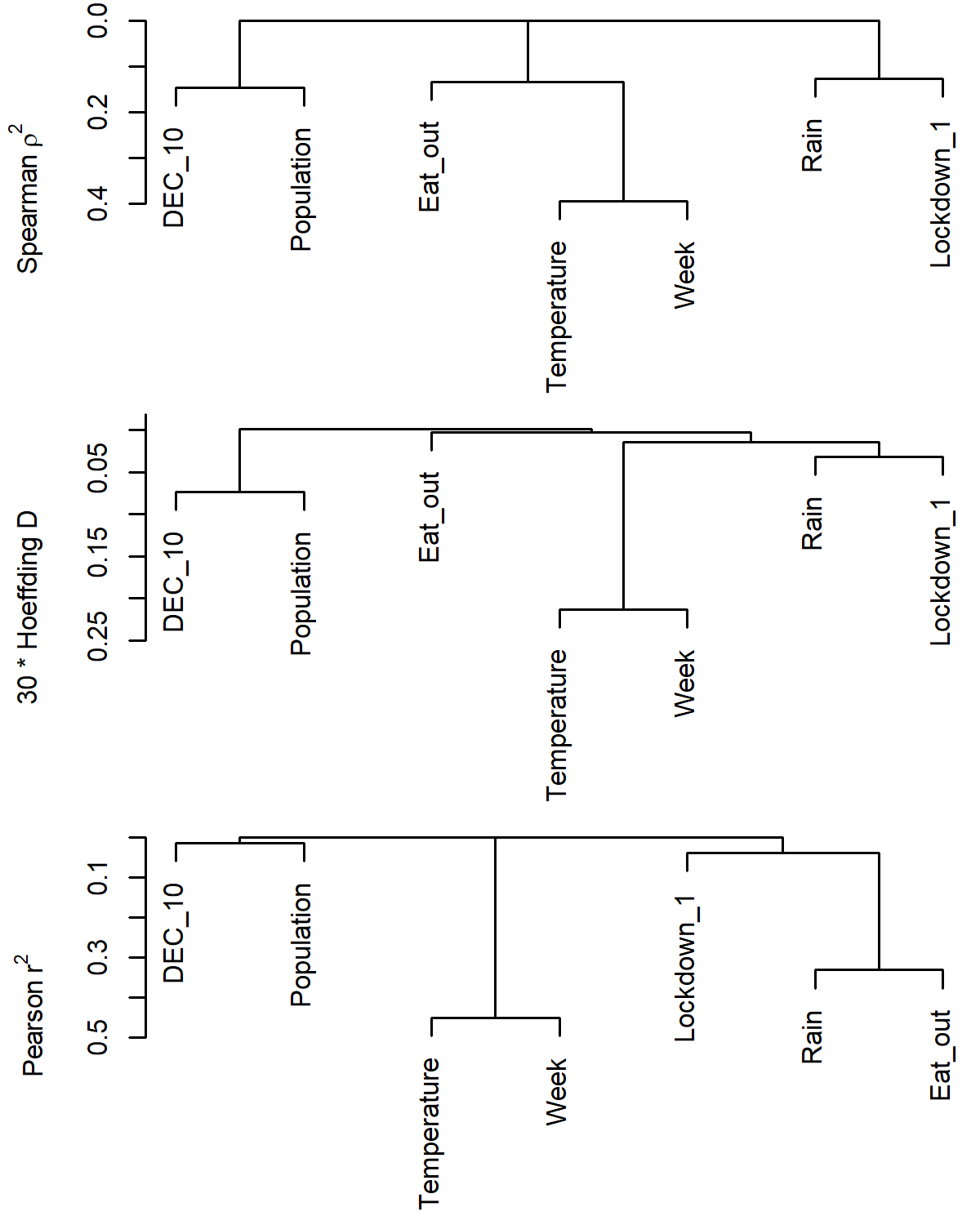

Supplement: Supplementary file: main dataset and code (compressed) [file EMS198536-supplement-Supplementary_file__main_dataset_and_code__compressed_.zip › Covid-19-Teesside-main/Figures/GLMM/Lin10/Lin10-B11119_Variable-Clustering.png]

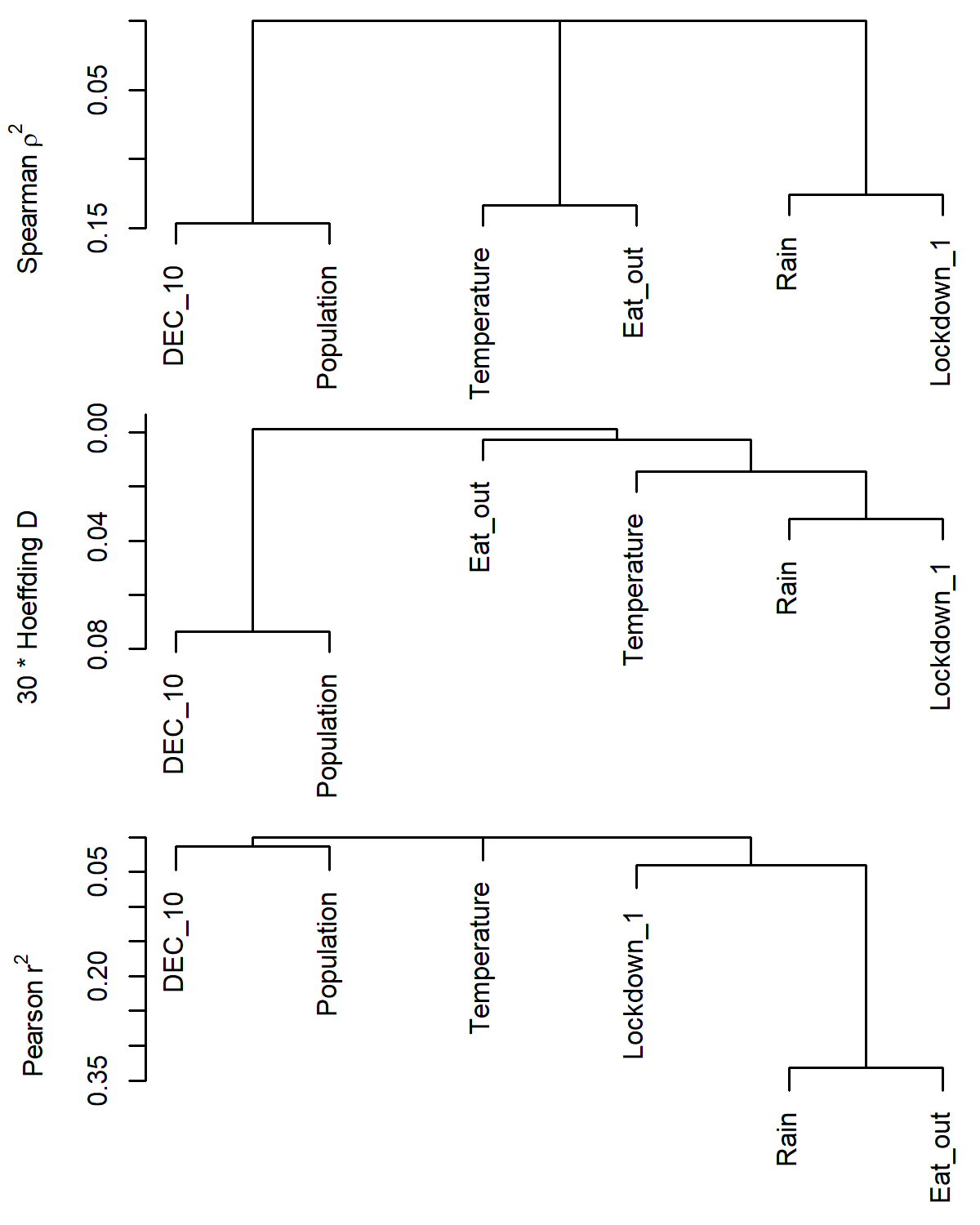

Supplement: Supplementary file: main dataset and code (compressed) [file EMS198536-supplement-Supplementary_file__main_dataset_and_code__compressed_.zip › Covid-19-Teesside-main/Figures/GLMM/Lin10/Lin10-B11119_Variable-Clustering_Without-week.png]

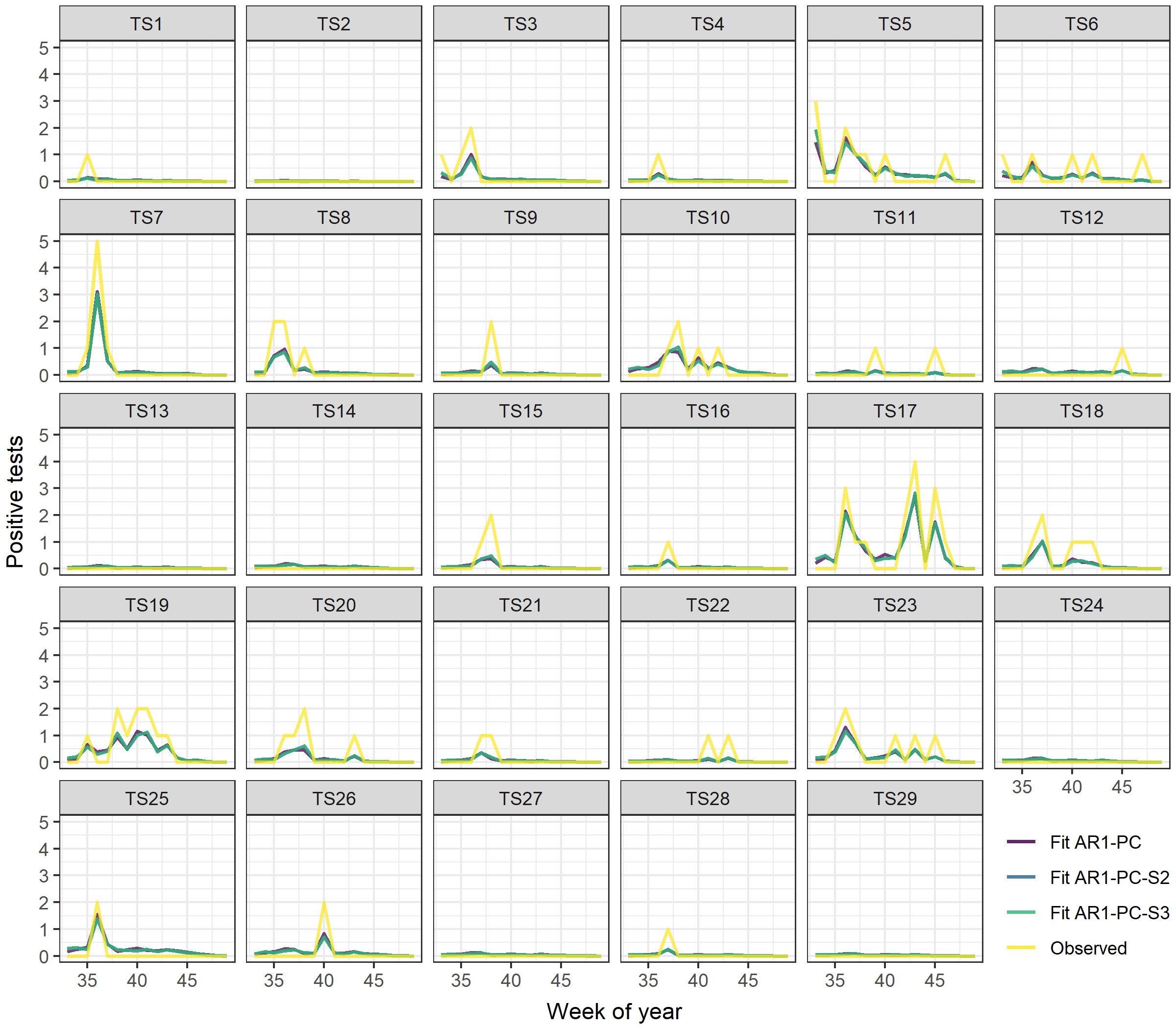

Supplement: Supplementary file: main dataset and code (compressed) [file EMS198536-supplement-Supplementary_file__main_dataset_and_code__compressed_.zip › Covid-19-Teesside-main/Figures/GLMM/Lin35/Lin35-B11309_GLMM_Obs-vs-Fit_AR1PC-AR1PCS2-AR1PCS3.png]

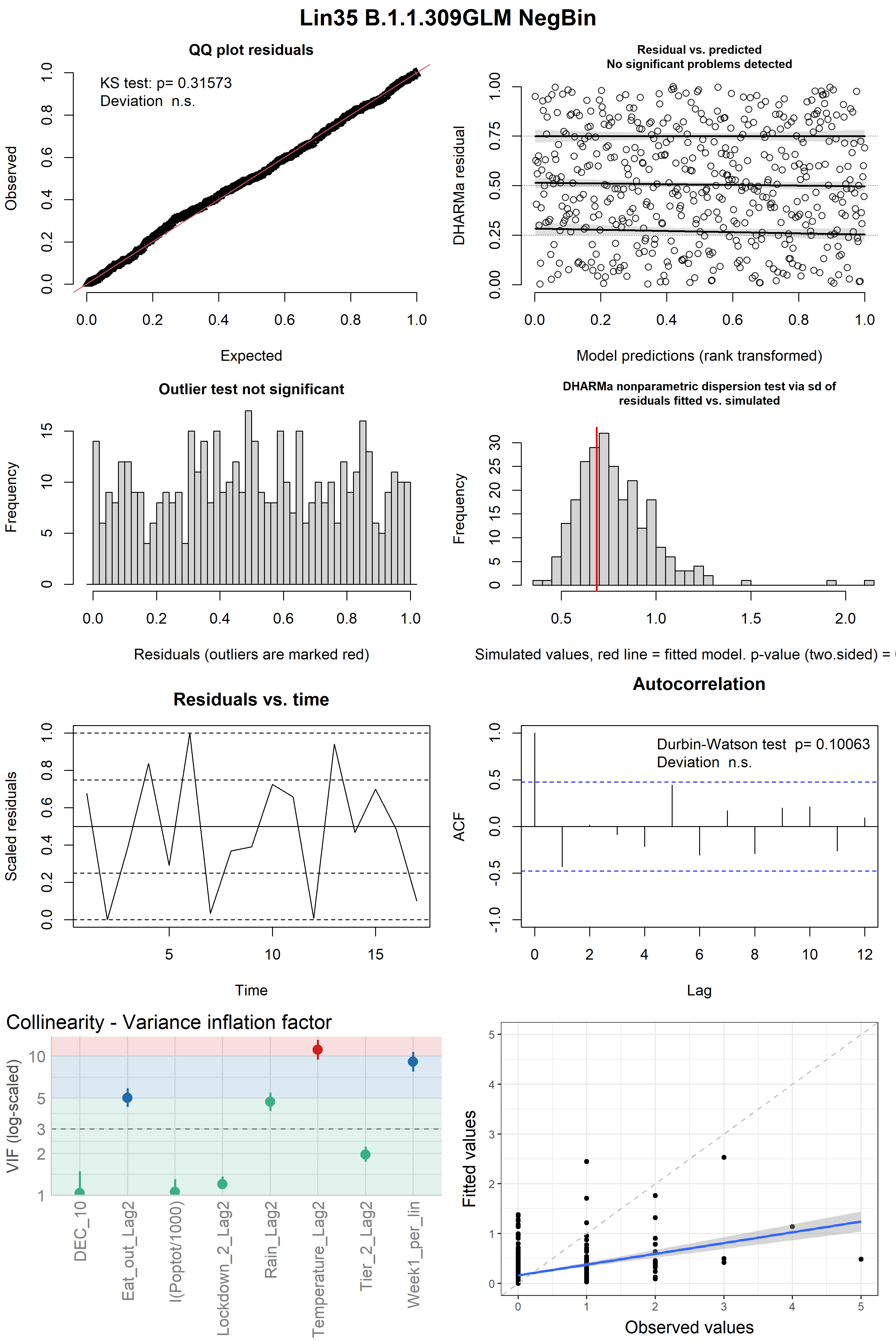

Supplement: Supplementary file: main dataset and code (compressed) [file EMS198536-supplement-Supplementary_file__main_dataset_and_code__compressed_.zip › Covid-19-Teesside-main/Figures/GLMM/Lin35/Lin35-B11309_NB_GLM_Full_Fit.png]

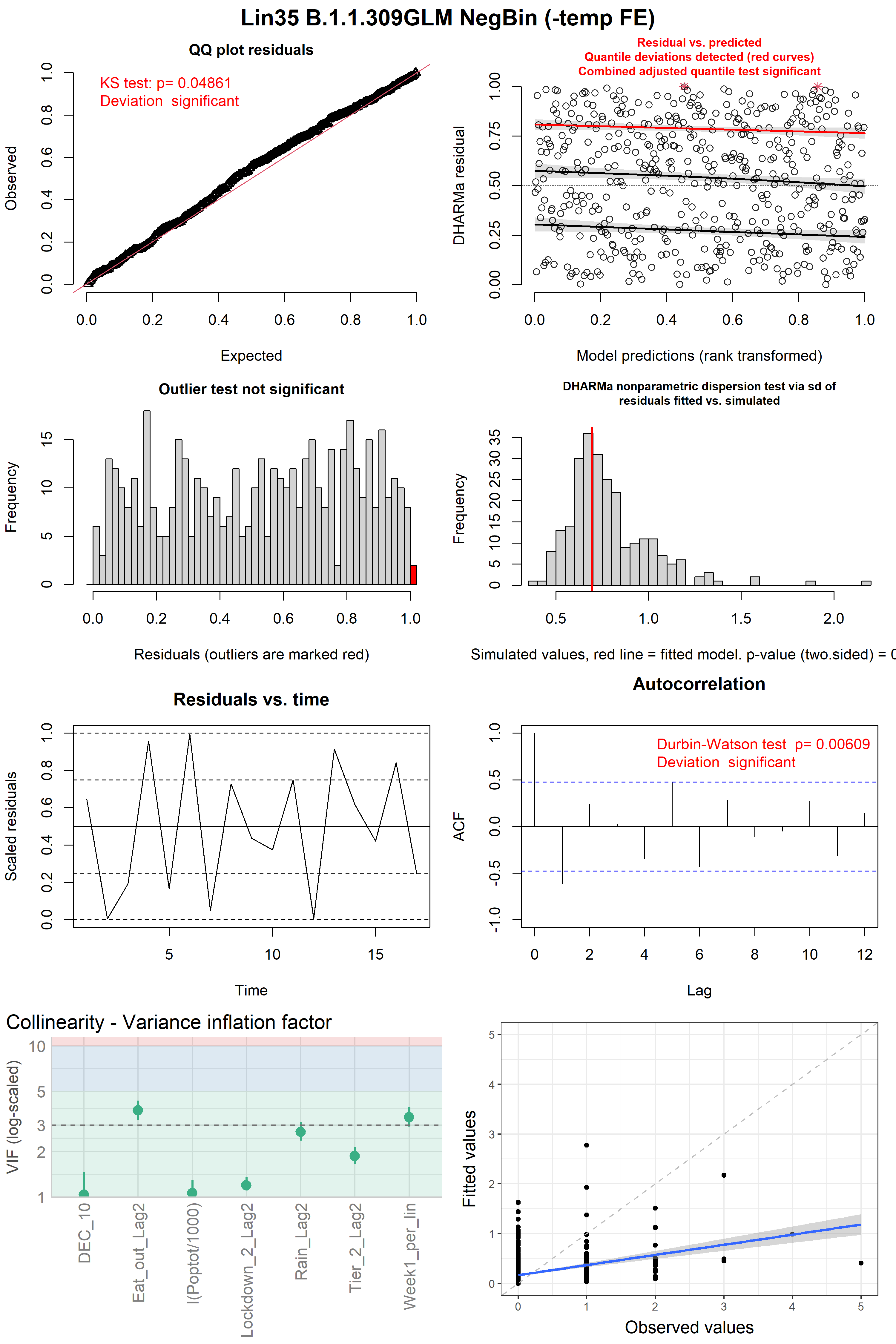

Supplement: Supplementary file: main dataset and code (compressed) [file EMS198536-supplement-Supplementary_file__main_dataset_and_code__compressed_.zip › Covid-19-Teesside-main/Figures/GLMM/Lin35/Lin35-B11309_NB_GLM_No-temp-FE_Fit.png]

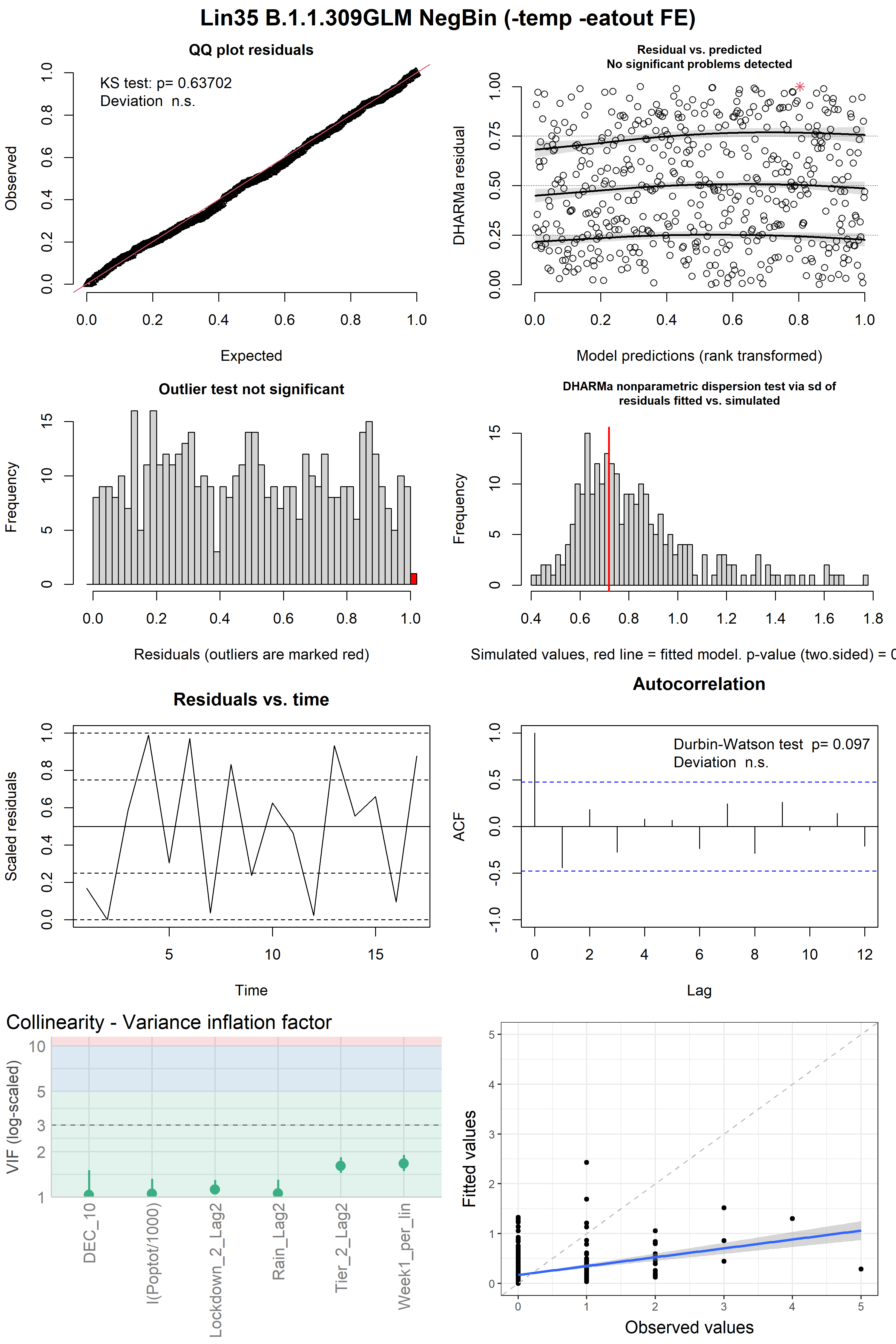

Supplement: Supplementary file: main dataset and code (compressed) [file EMS198536-supplement-Supplementary_file__main_dataset_and_code__compressed_.zip › Covid-19-Teesside-main/Figures/GLMM/Lin35/Lin35-B11309_NB_GLM_No-temp-no-eatout-FE_Fit.png]

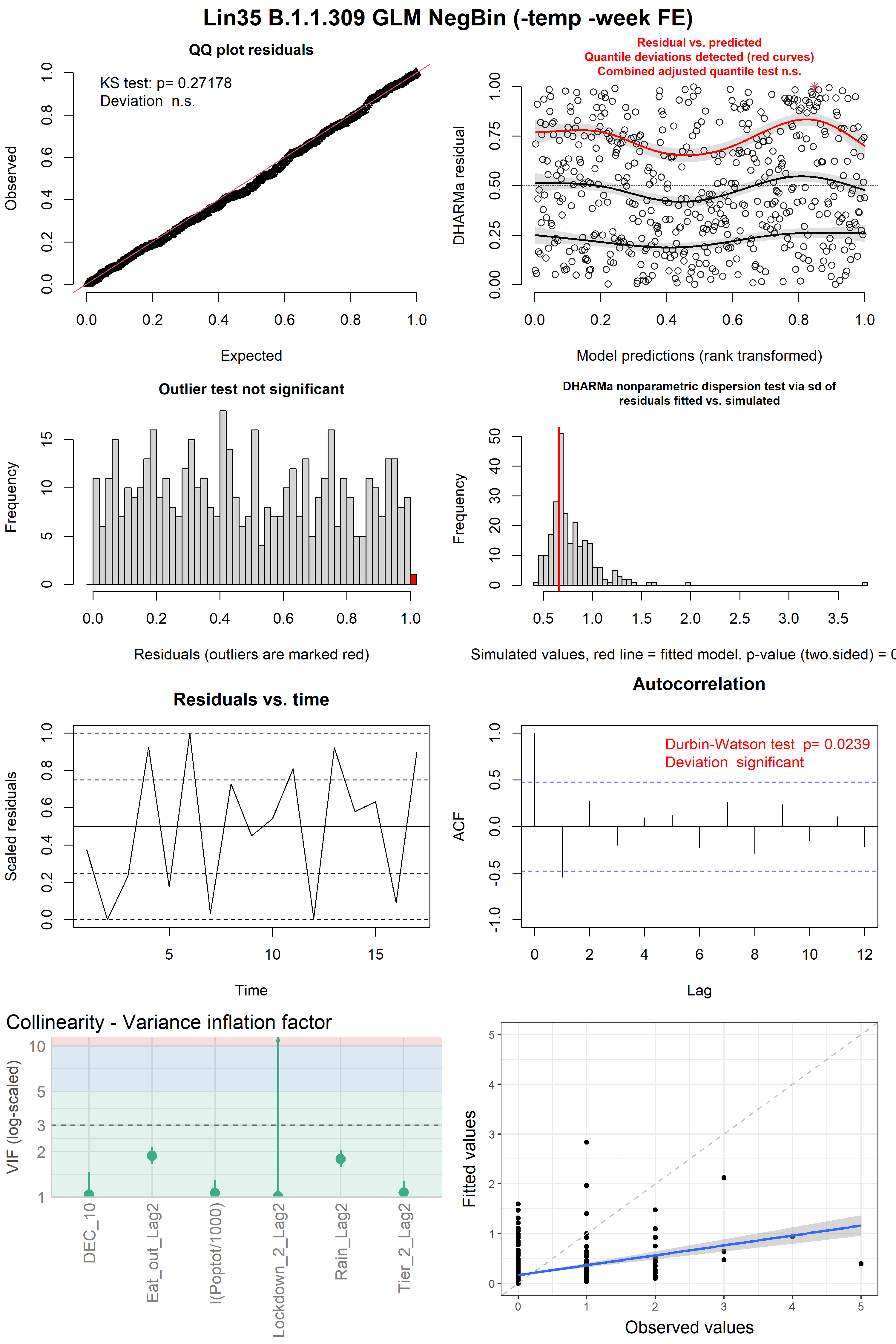

Supplement: Supplementary file: main dataset and code (compressed) [file EMS198536-supplement-Supplementary_file__main_dataset_and_code__compressed_.zip › Covid-19-Teesside-main/Figures/GLMM/Lin35/Lin35-B11309_NB_GLM_No-temp-no-week-FE_Fit.png]

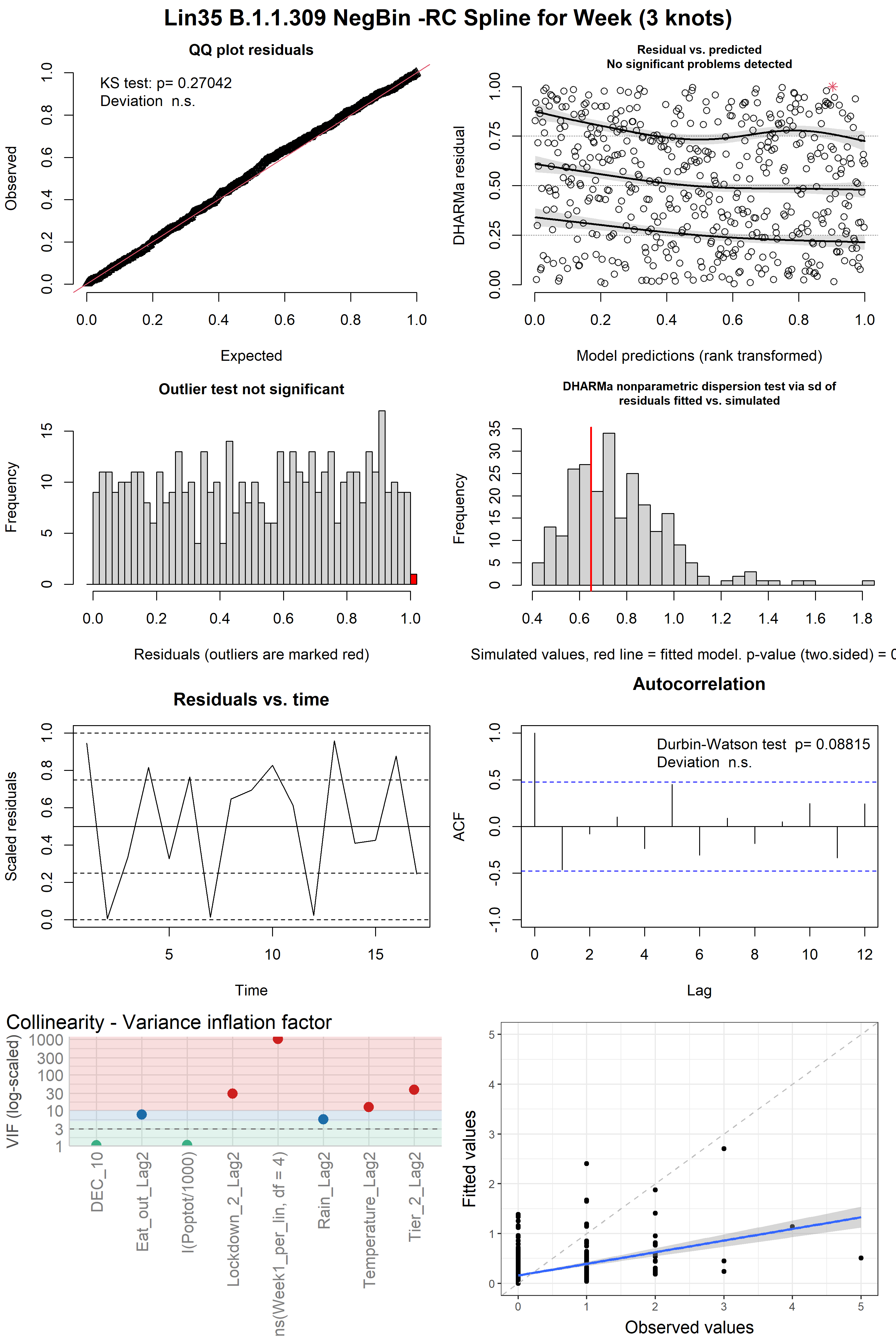

Supplement: Supplementary file: main dataset and code (compressed) [file EMS198536-supplement-Supplementary_file__main_dataset_and_code__compressed_.zip › Covid-19-Teesside-main/Figures/GLMM/Lin35/Lin35-B11309_NB_RCS-Week-3knots_Fit.png]

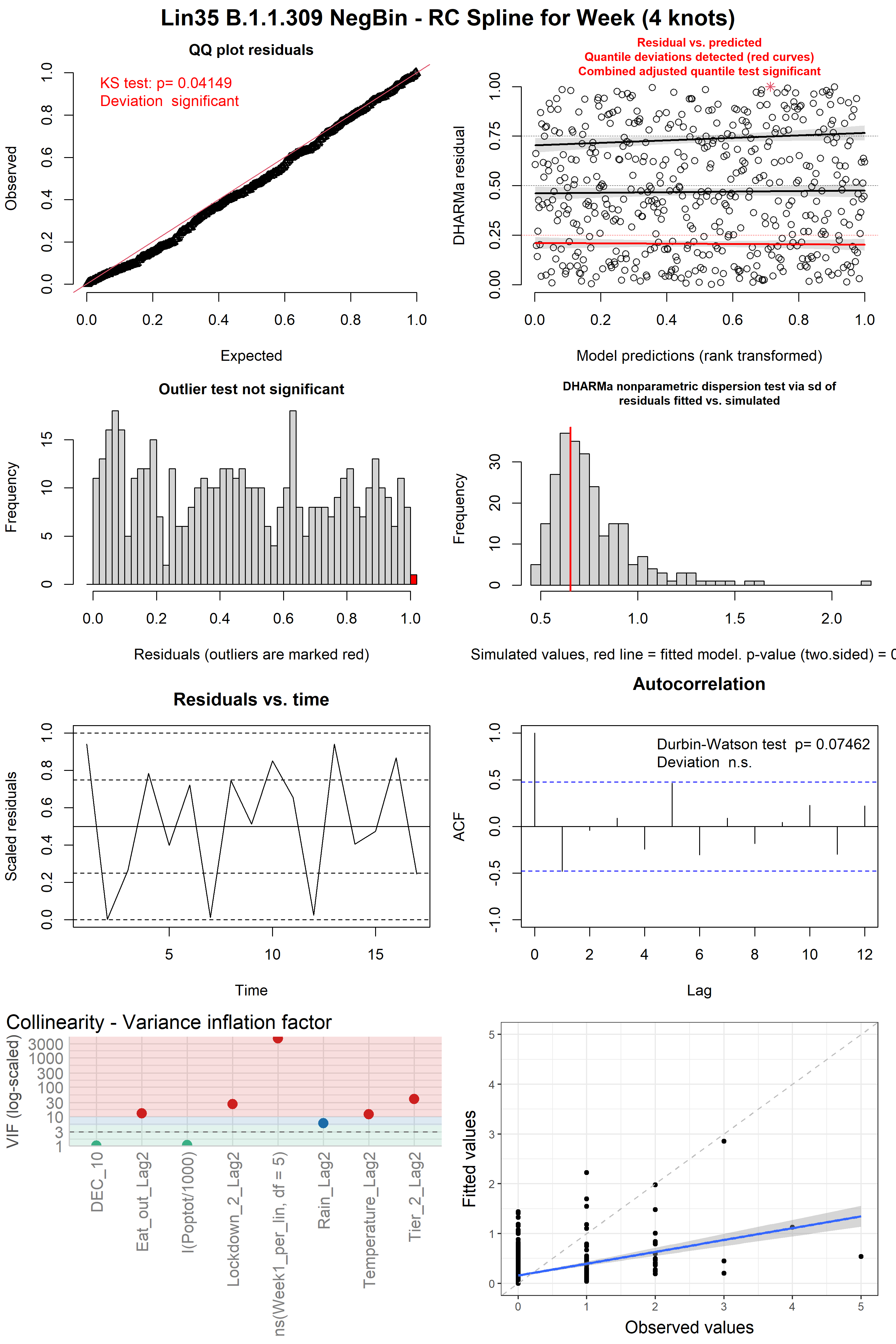

Supplement: Supplementary file: main dataset and code (compressed) [file EMS198536-supplement-Supplementary_file__main_dataset_and_code__compressed_.zip › Covid-19-Teesside-main/Figures/GLMM/Lin35/Lin35-B11309_NB_RCS-Week-4knots_Fit.png]

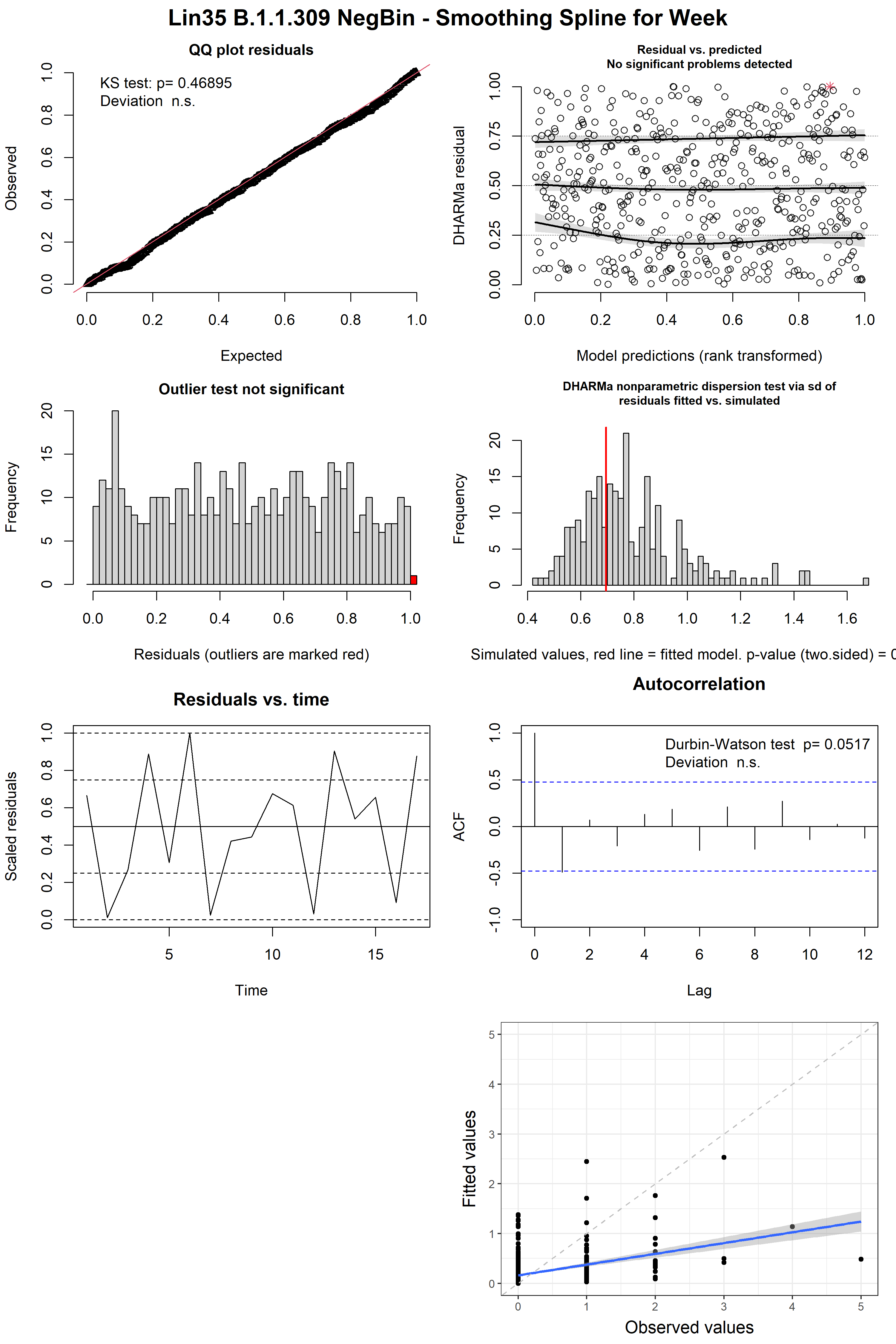

Supplement: Supplementary file: main dataset and code (compressed) [file EMS198536-supplement-Supplementary_file__main_dataset_and_code__compressed_.zip › Covid-19-Teesside-main/Figures/GLMM/Lin35/Lin35-B11309_NB_SmoothSpline-Week-TPS_Fit.png]

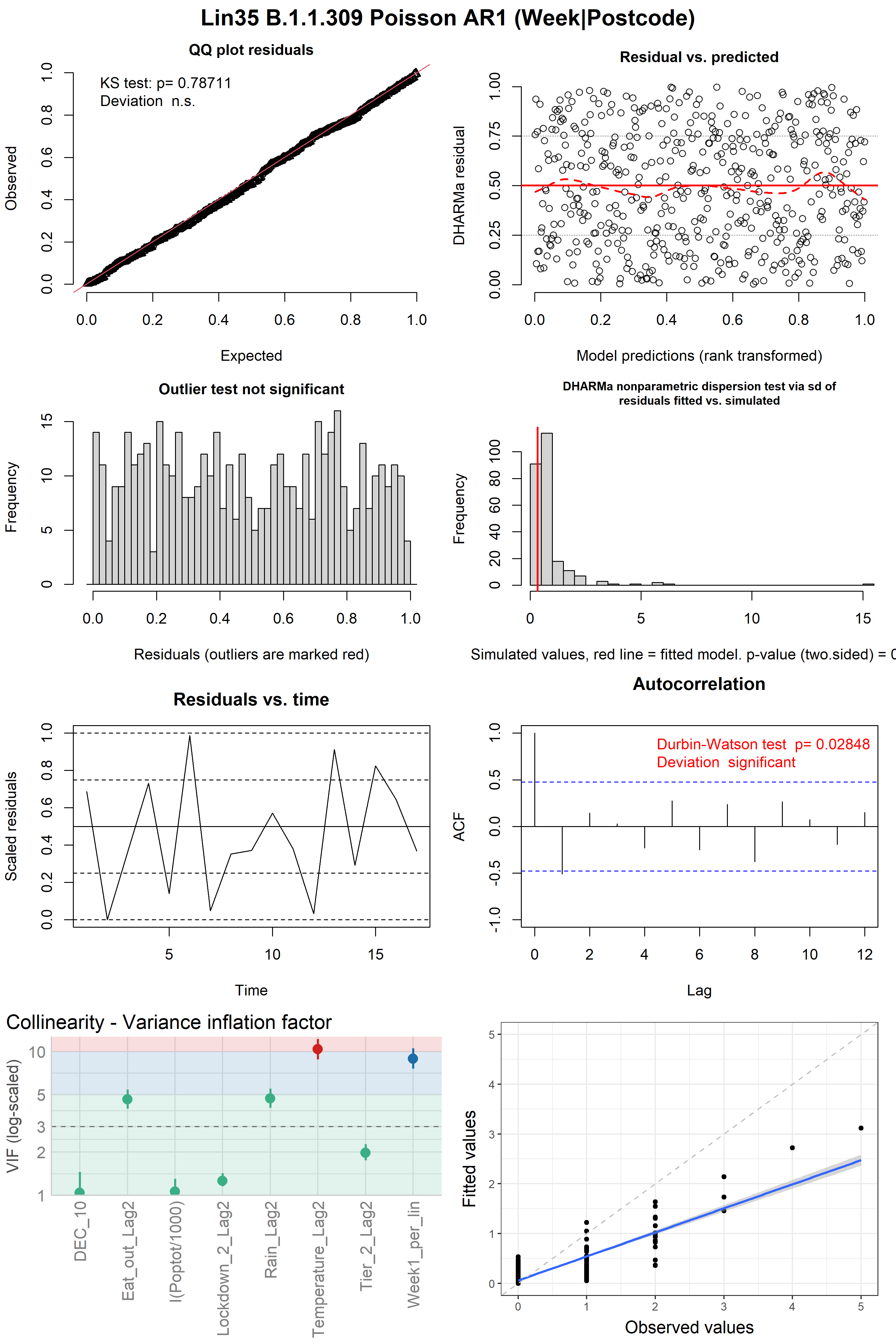

Supplement: Supplementary file: main dataset and code (compressed) [file EMS198536-supplement-Supplementary_file__main_dataset_and_code__compressed_.zip › Covid-19-Teesside-main/Figures/GLMM/Lin35/Lin35-B11309_Po_AR1-Week-Postcode_Fit.png]

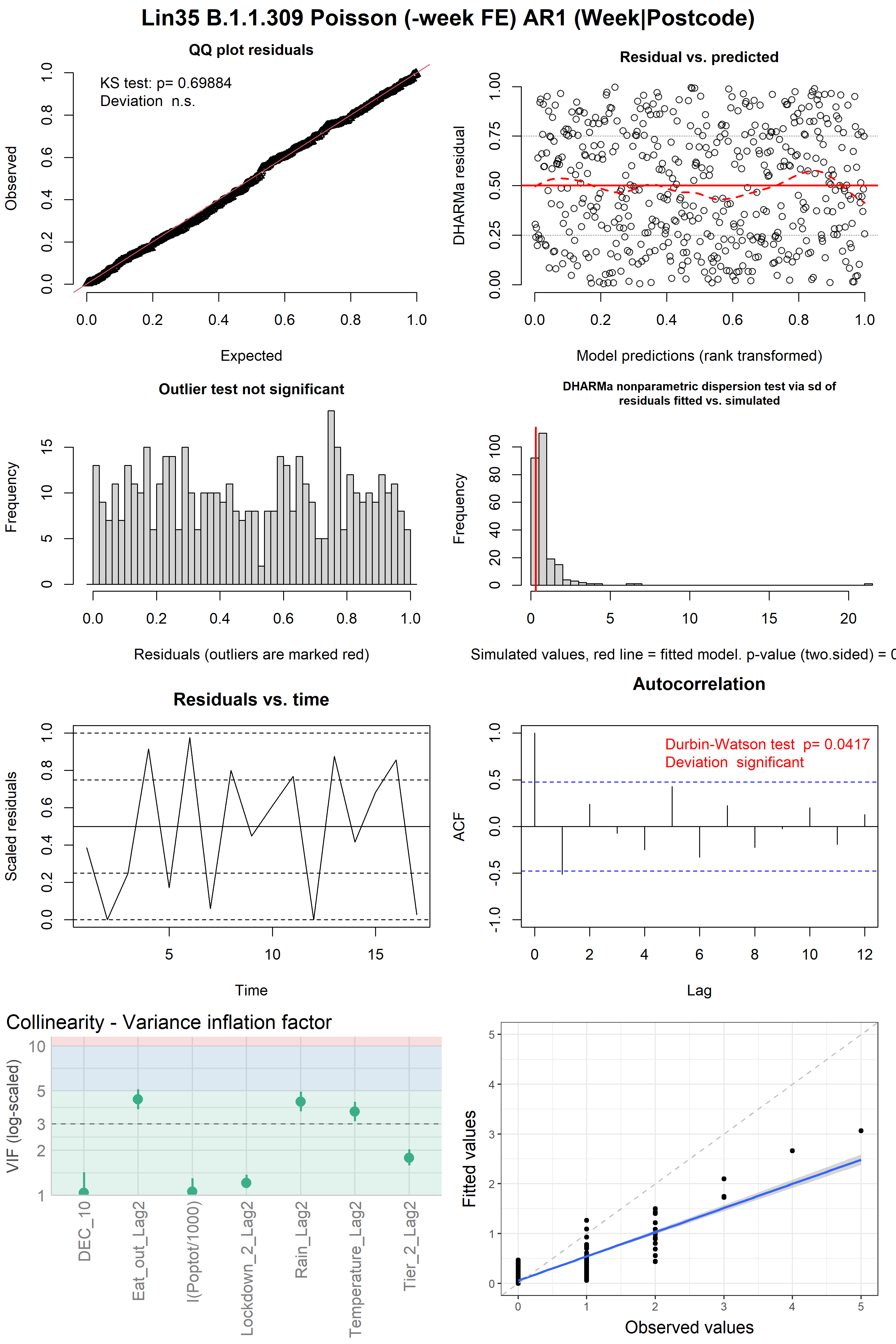

Supplement: Supplementary file: main dataset and code (compressed) [file EMS198536-supplement-Supplementary_file__main_dataset_and_code__compressed_.zip › Covid-19-Teesside-main/Figures/GLMM/Lin35/Lin35-B11309_Po_AR1-Week-Postcode_No-week-FE_Fit.png]

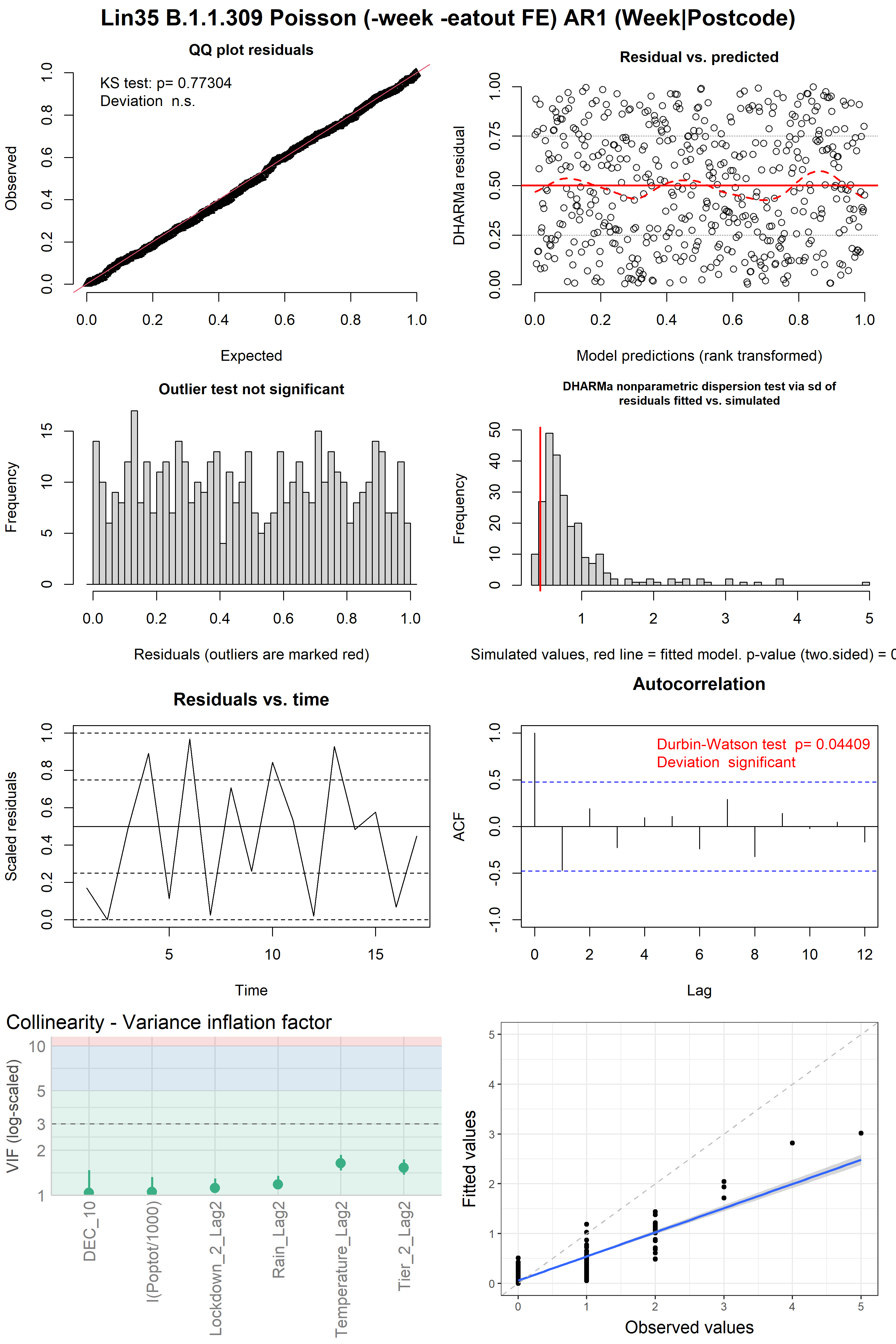

Supplement: Supplementary file: main dataset and code (compressed) [file EMS198536-supplement-Supplementary_file__main_dataset_and_code__compressed_.zip › Covid-19-Teesside-main/Figures/GLMM/Lin35/Lin35-B11309_Po_AR1-Week-Postcode_No-week-no-eatout-FE_Fit.png]

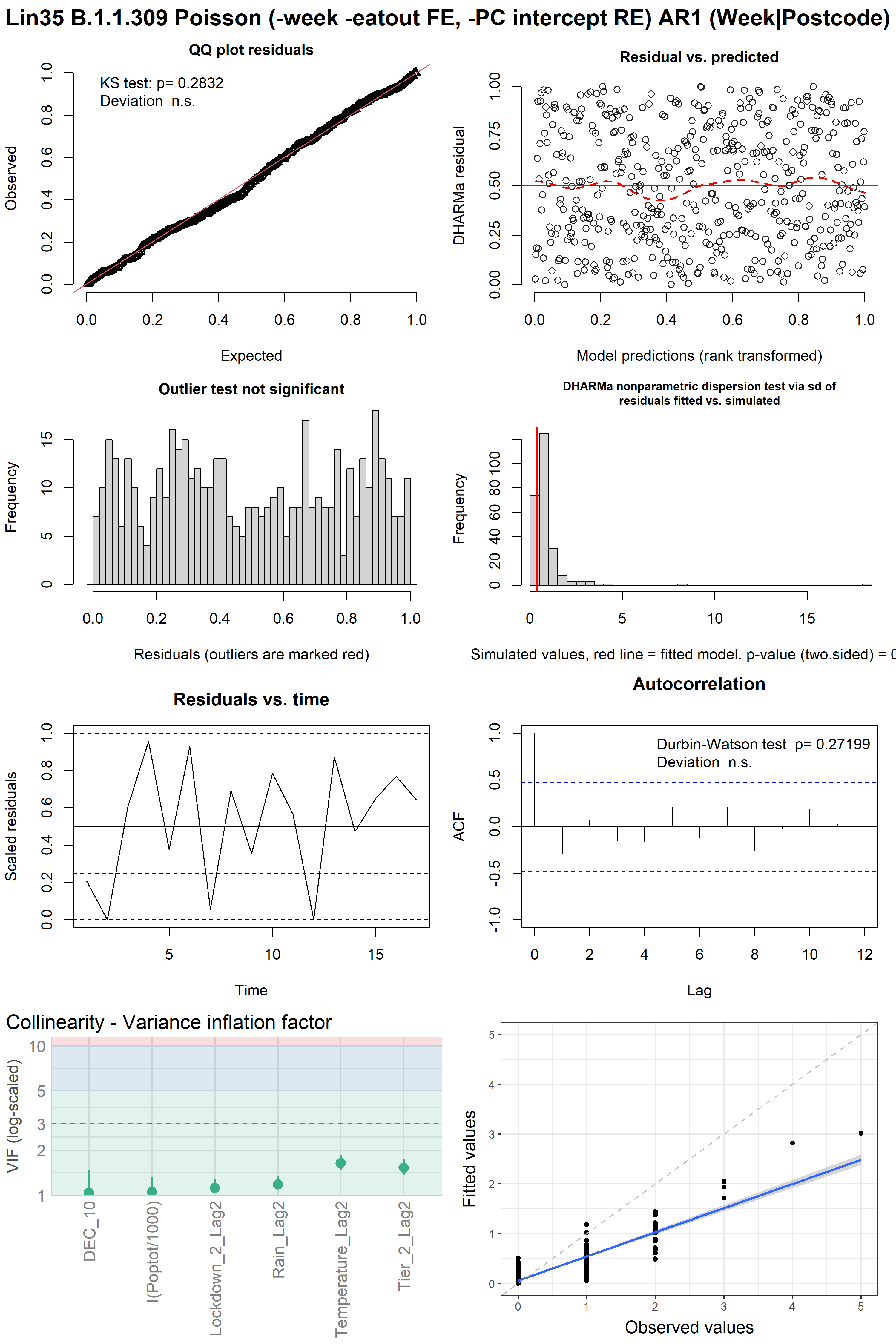

Supplement: Supplementary file: main dataset and code (compressed) [file EMS198536-supplement-Supplementary_file__main_dataset_and_code__compressed_.zip › Covid-19-Teesside-main/Figures/GLMM/Lin35/Lin35-B11309_Po_AR1-Week-Postcode_No-week-no-eatout-FE_No-PC-intercept-RE_Fit.png]

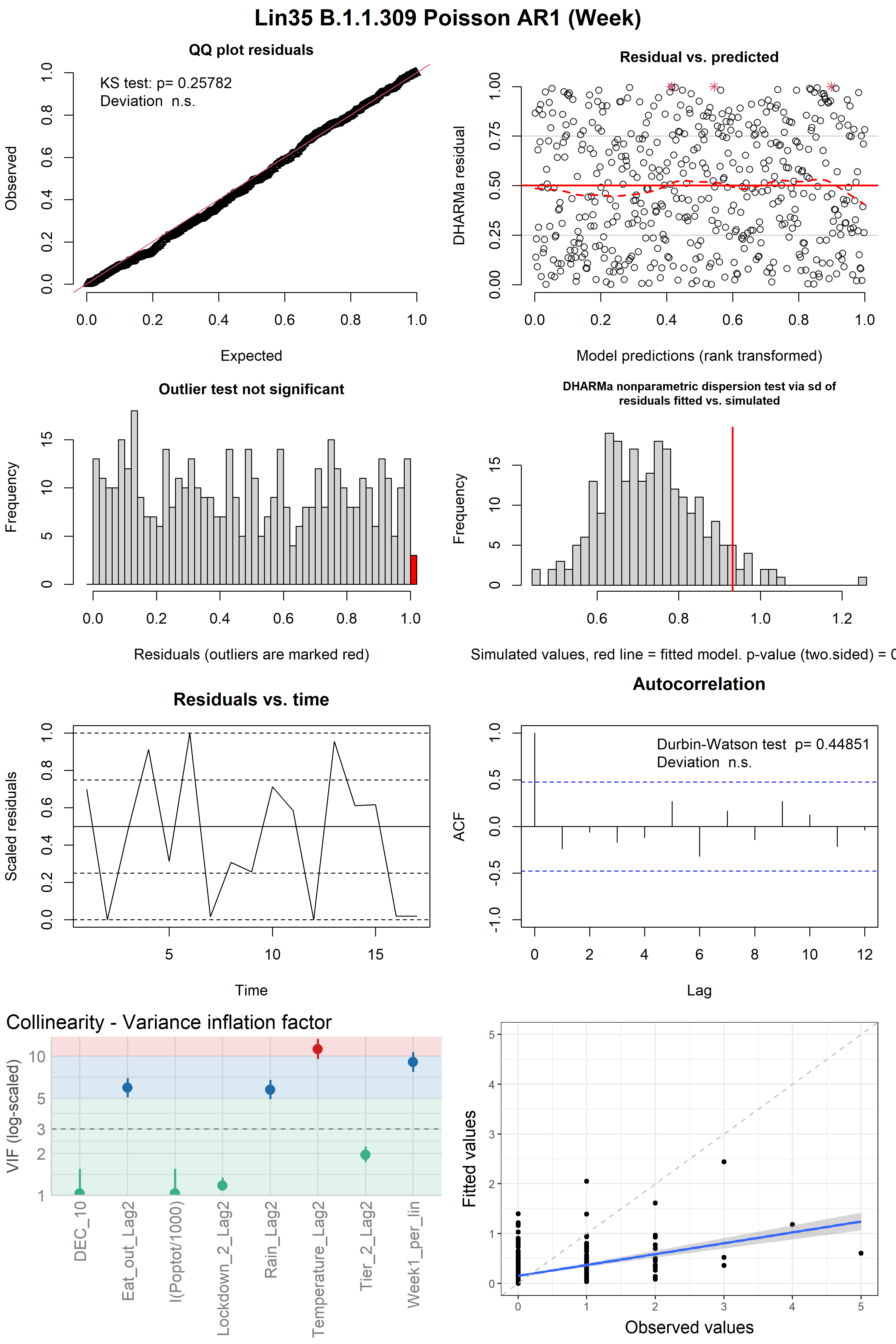

Supplement: Supplementary file: main dataset and code (compressed) [file EMS198536-supplement-Supplementary_file__main_dataset_and_code__compressed_.zip › Covid-19-Teesside-main/Figures/GLMM/Lin35/Lin35-B11309_Po_AR1-Week_Fit.png]

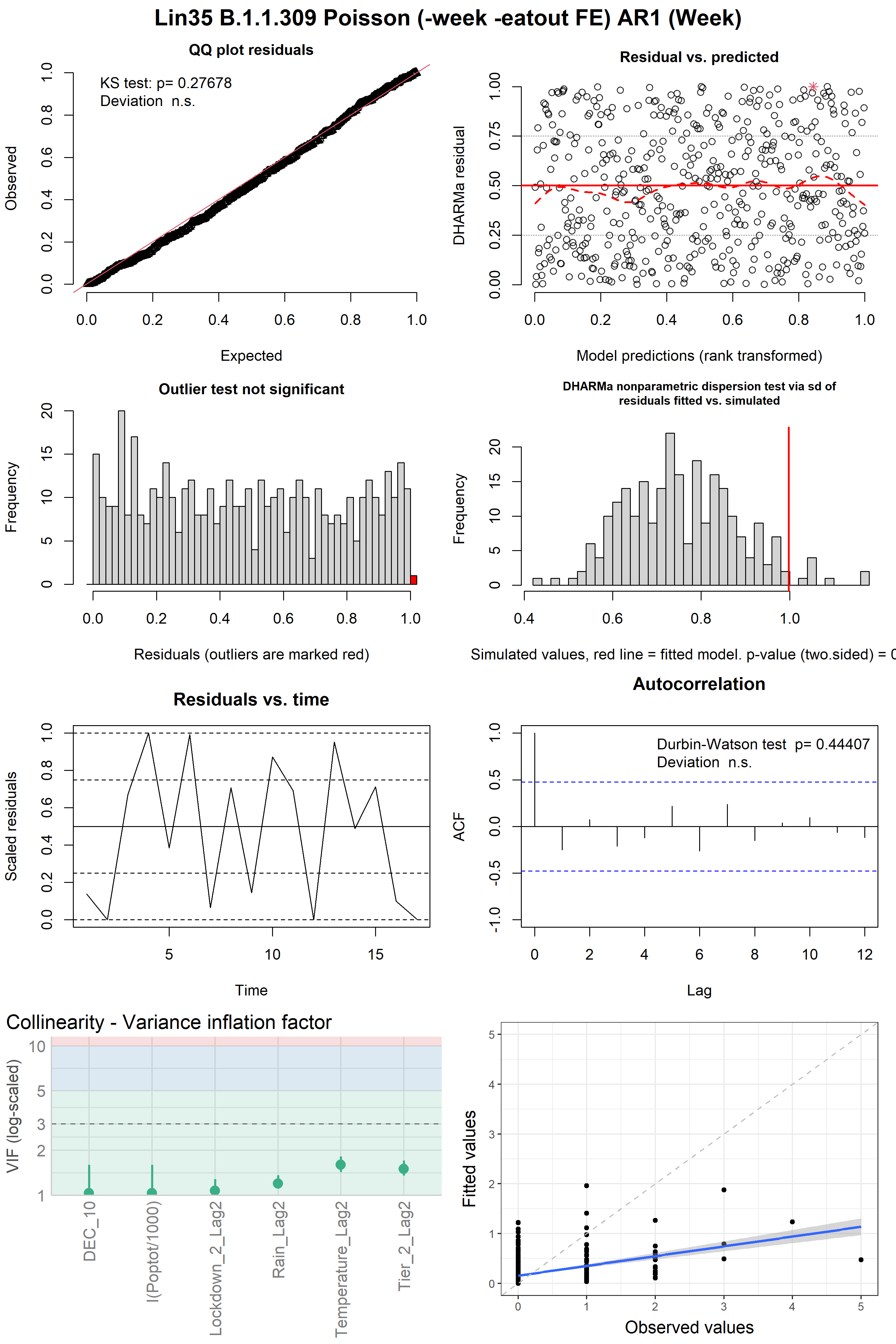

Supplement: Supplementary file: main dataset and code (compressed) [file EMS198536-supplement-Supplementary_file__main_dataset_and_code__compressed_.zip › Covid-19-Teesside-main/Figures/GLMM/Lin35/Lin35-B11309_Po_AR1-Week_No-week-no-eatout-FE_Fit.png]

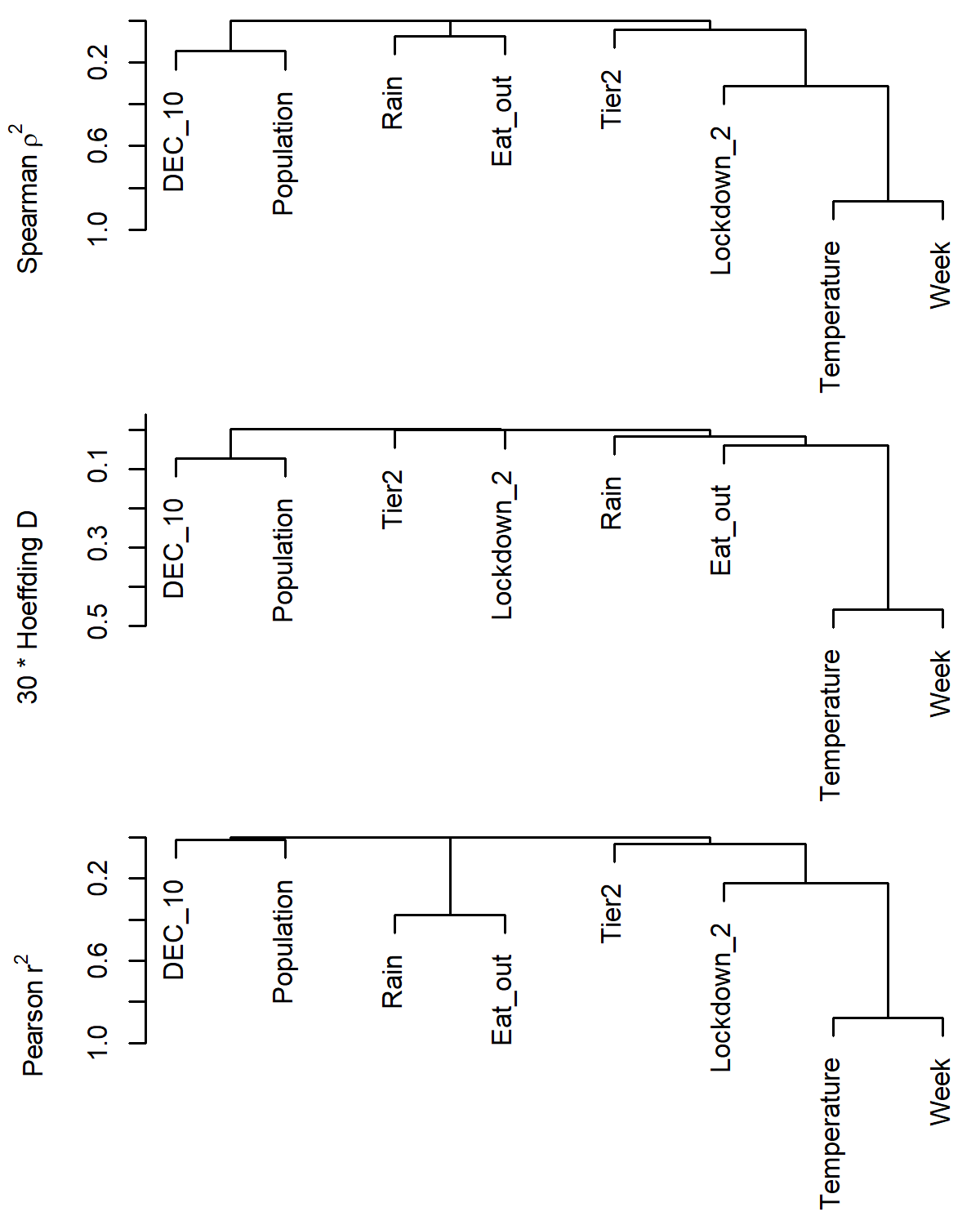

Supplement: Supplementary file: main dataset and code (compressed) [file EMS198536-supplement-Supplementary_file__main_dataset_and_code__compressed_.zip › Covid-19-Teesside-main/Figures/GLMM/Lin35/Lin35-B11309_Variable-Clustering.png]

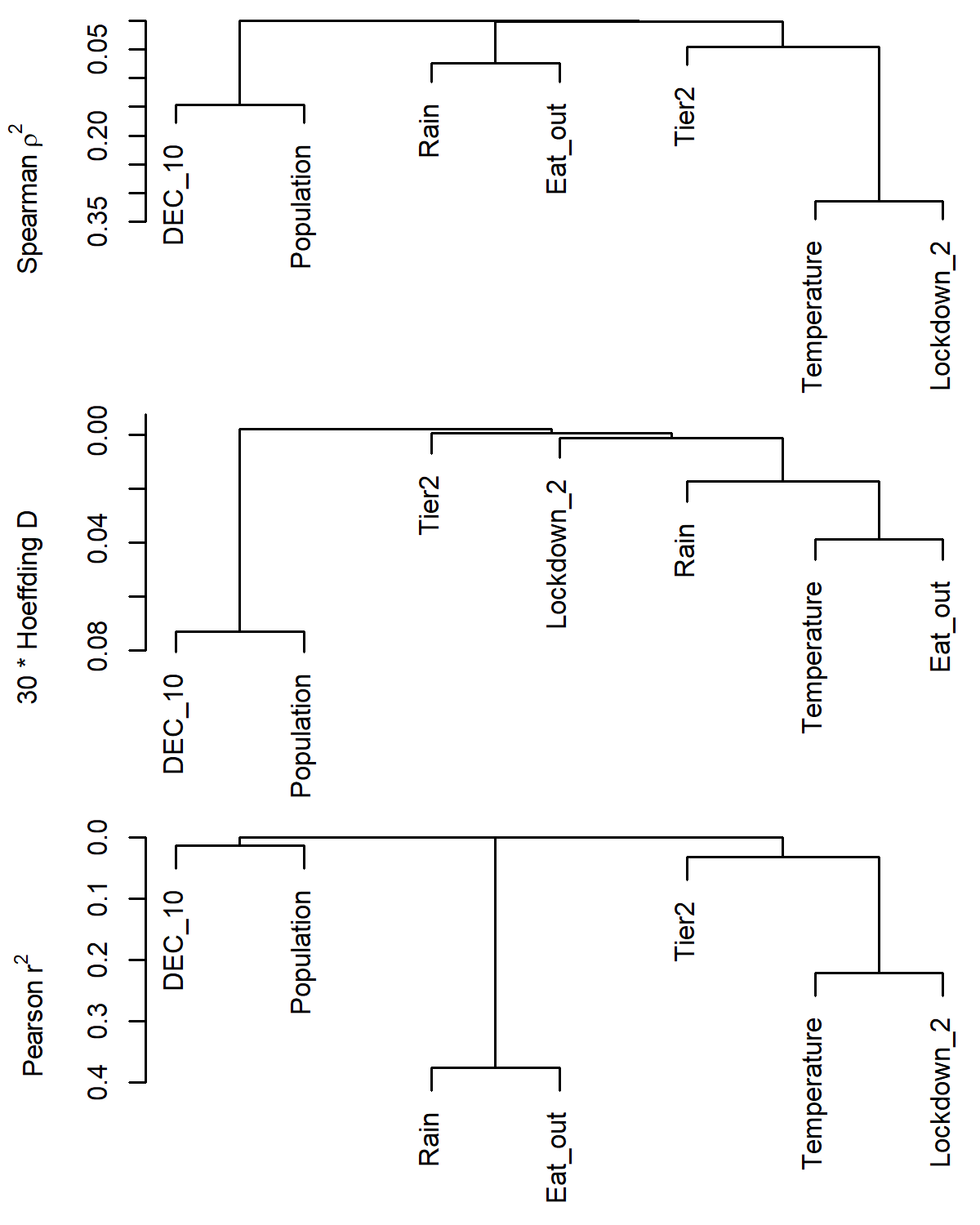

Supplement: Supplementary file: main dataset and code (compressed) [file EMS198536-supplement-Supplementary_file__main_dataset_and_code__compressed_.zip › Covid-19-Teesside-main/Figures/GLMM/Lin35/Lin35-B11309_Variable-Clustering_Without-Week.png]

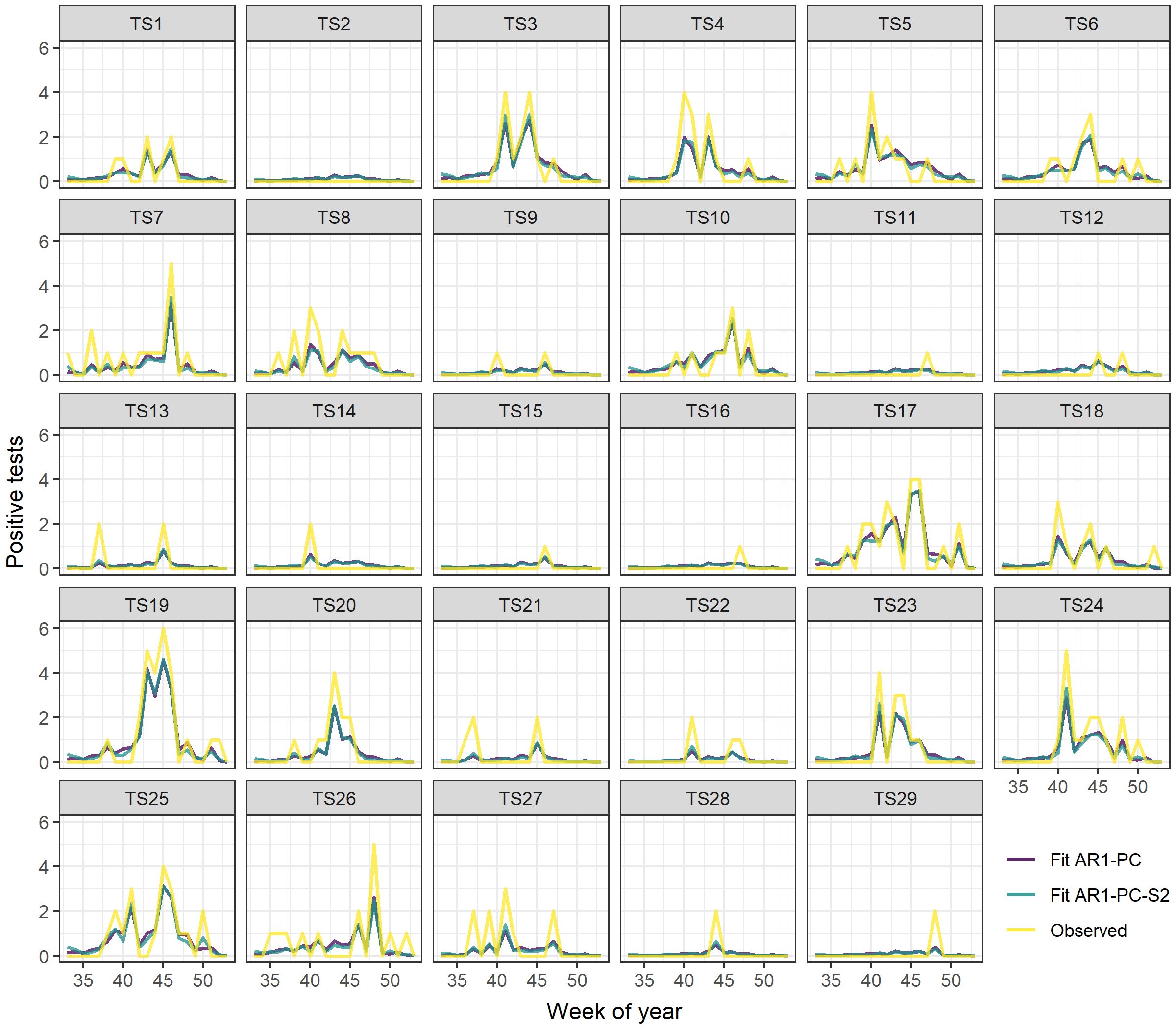

Supplement: Supplementary file: main dataset and code (compressed) [file EMS198536-supplement-Supplementary_file__main_dataset_and_code__compressed_.zip › Covid-19-Teesside-main/Figures/GLMM/Lin37/Lin37-B11315_GLMM_Obs-vs-Fit_AR1PC-AR1PCS2.png]

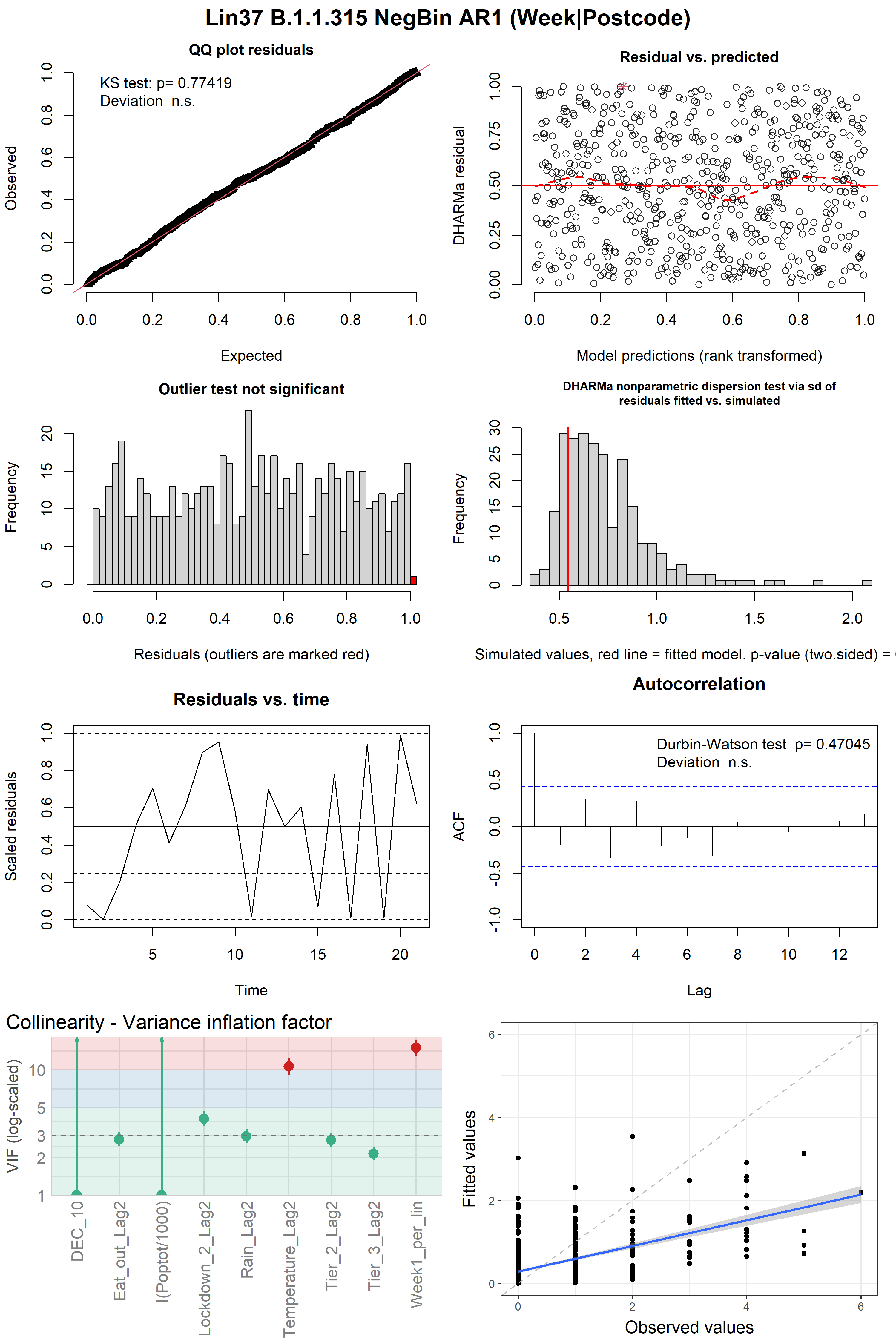

Supplement: Supplementary file: main dataset and code (compressed) [file EMS198536-supplement-Supplementary_file__main_dataset_and_code__compressed_.zip › Covid-19-Teesside-main/Figures/GLMM/Lin37/Lin37-B11315_NB_AR1-Week-Postcode_Fit.png]

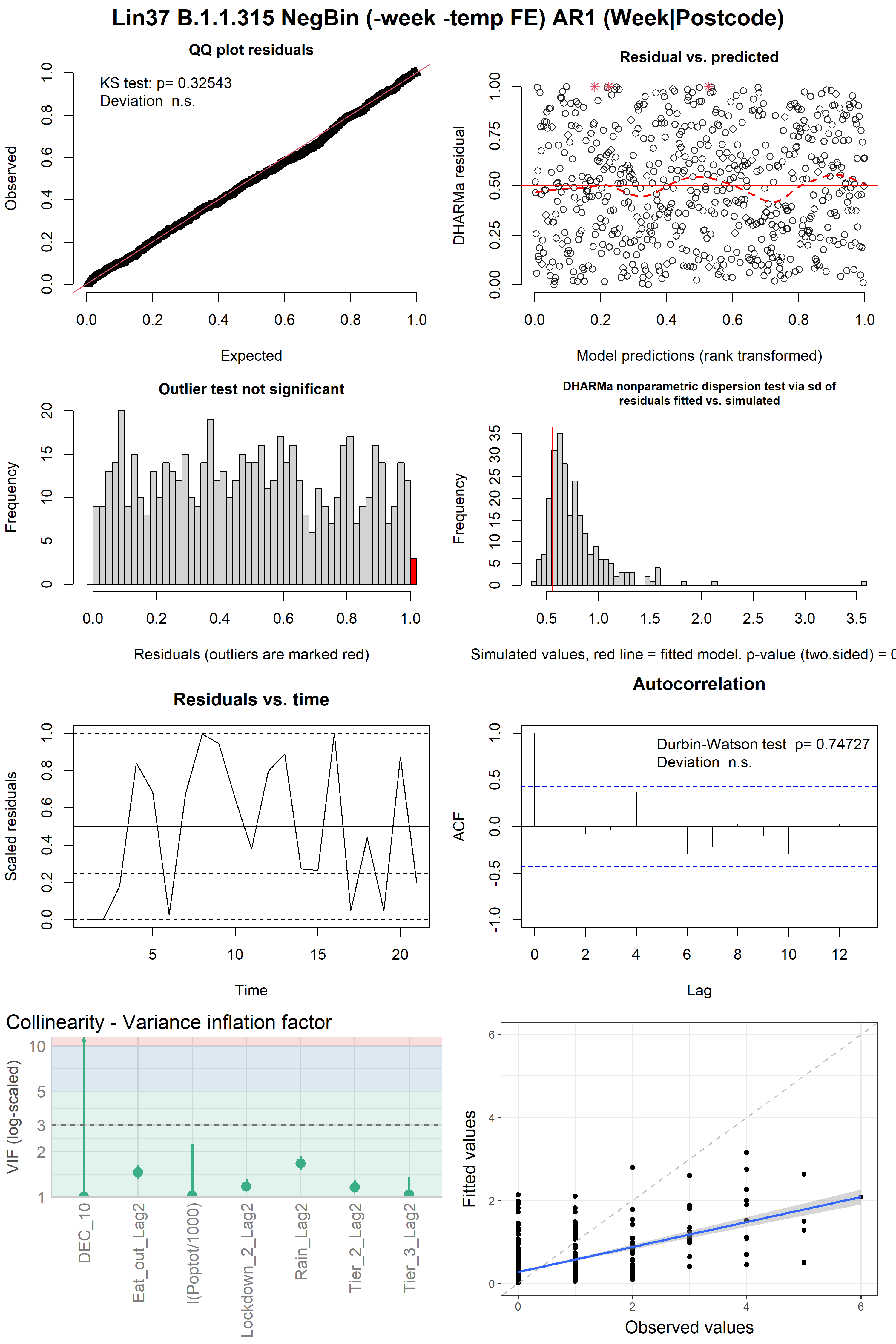

Supplement: Supplementary file: main dataset and code (compressed) [file EMS198536-supplement-Supplementary_file__main_dataset_and_code__compressed_.zip › Covid-19-Teesside-main/Figures/GLMM/Lin37/Lin37-B11315_NB_AR1-Week-Postcode_No-week-no-temp-FE_Fit.png]

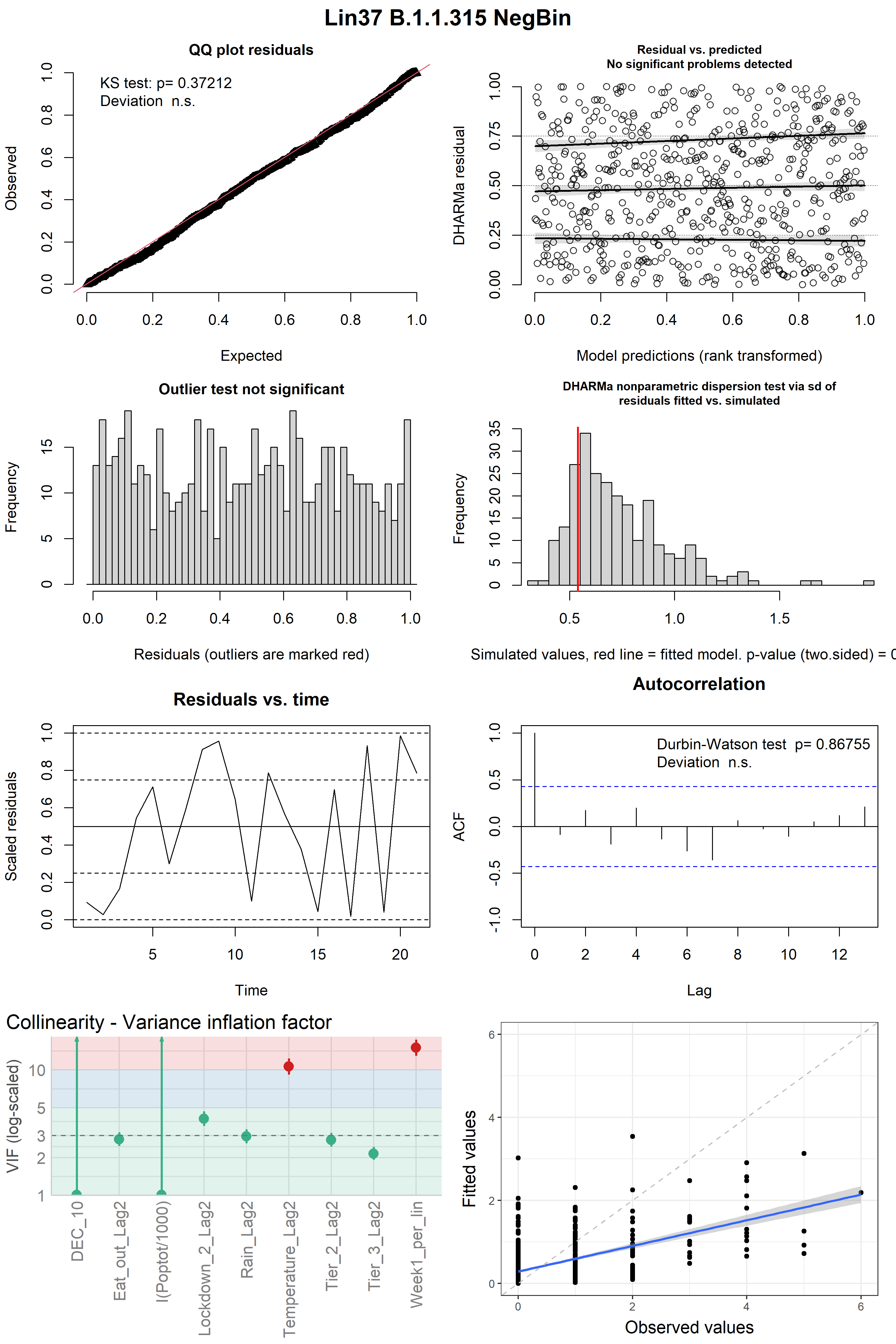

Supplement: Supplementary file: main dataset and code (compressed) [file EMS198536-supplement-Supplementary_file__main_dataset_and_code__compressed_.zip › Covid-19-Teesside-main/Figures/GLMM/Lin37/Lin37-B11315_NB_Full_Fit.png]

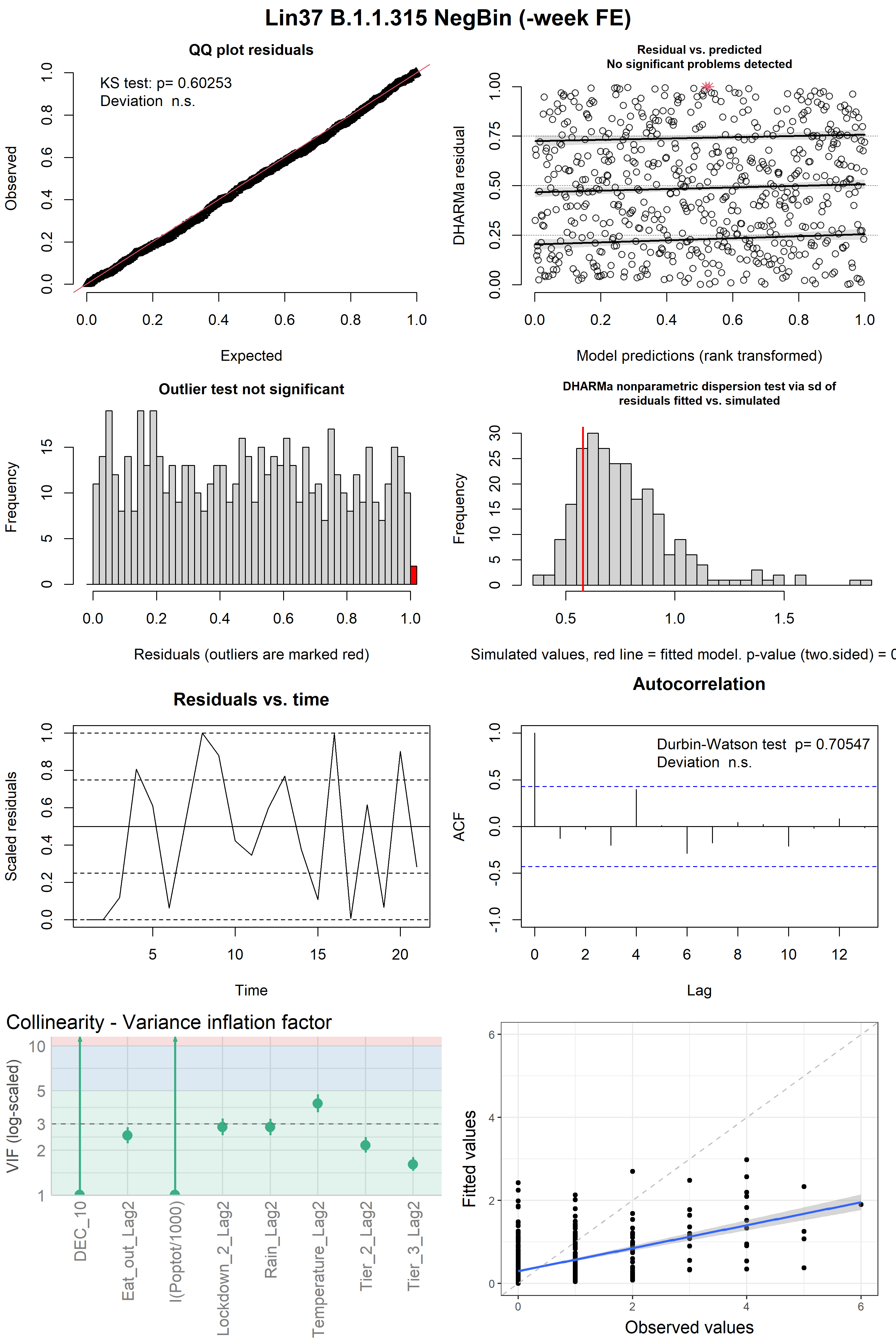

Supplement: Supplementary file: main dataset and code (compressed) [file EMS198536-supplement-Supplementary_file__main_dataset_and_code__compressed_.zip › Covid-19-Teesside-main/Figures/GLMM/Lin37/Lin37-B11315_NB_No-week-FE_Fit.png]

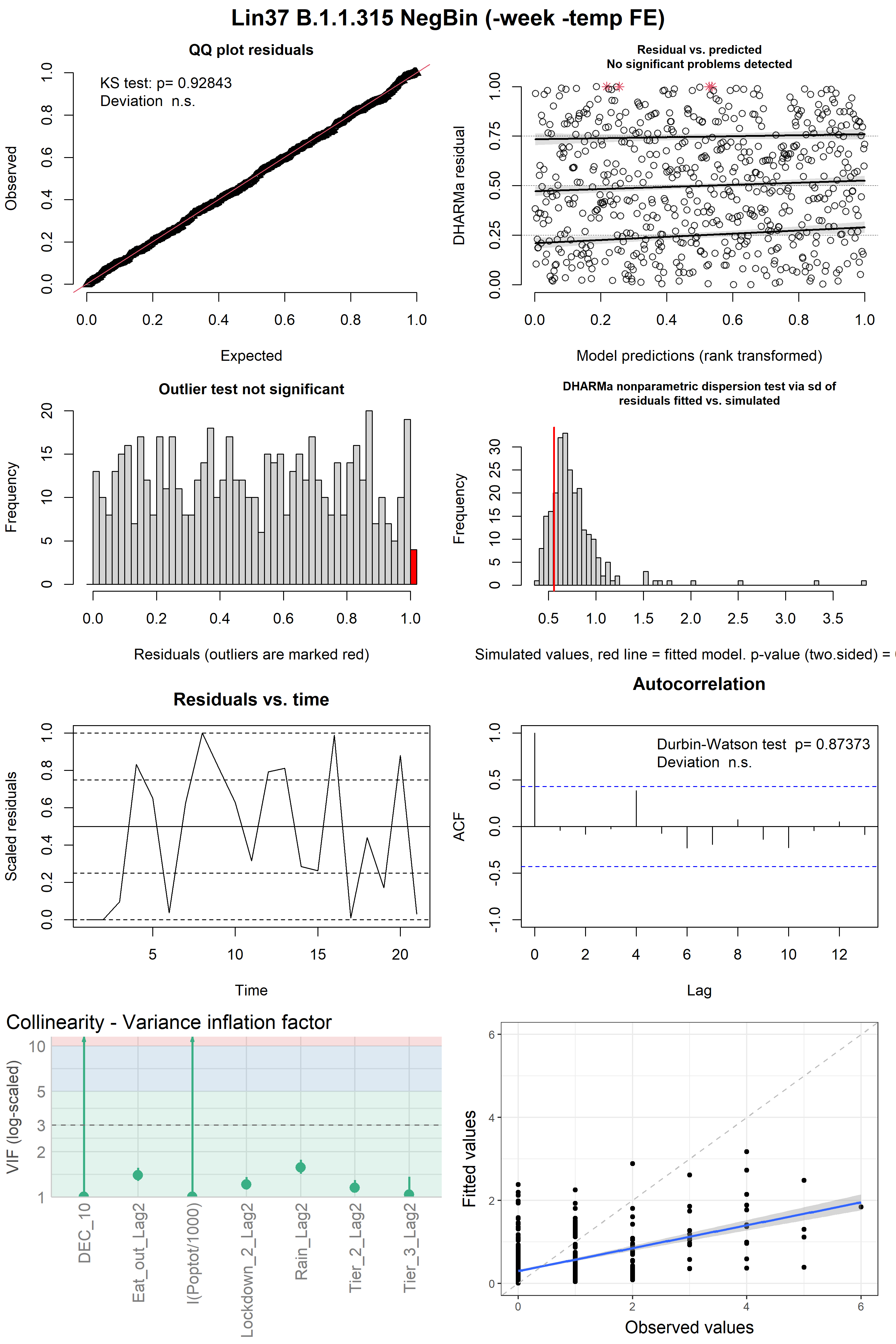

Supplement: Supplementary file: main dataset and code (compressed) [file EMS198536-supplement-Supplementary_file__main_dataset_and_code__compressed_.zip › Covid-19-Teesside-main/Figures/GLMM/Lin37/Lin37-B11315_NB_No-week-no-temp-FE_Fit.png]

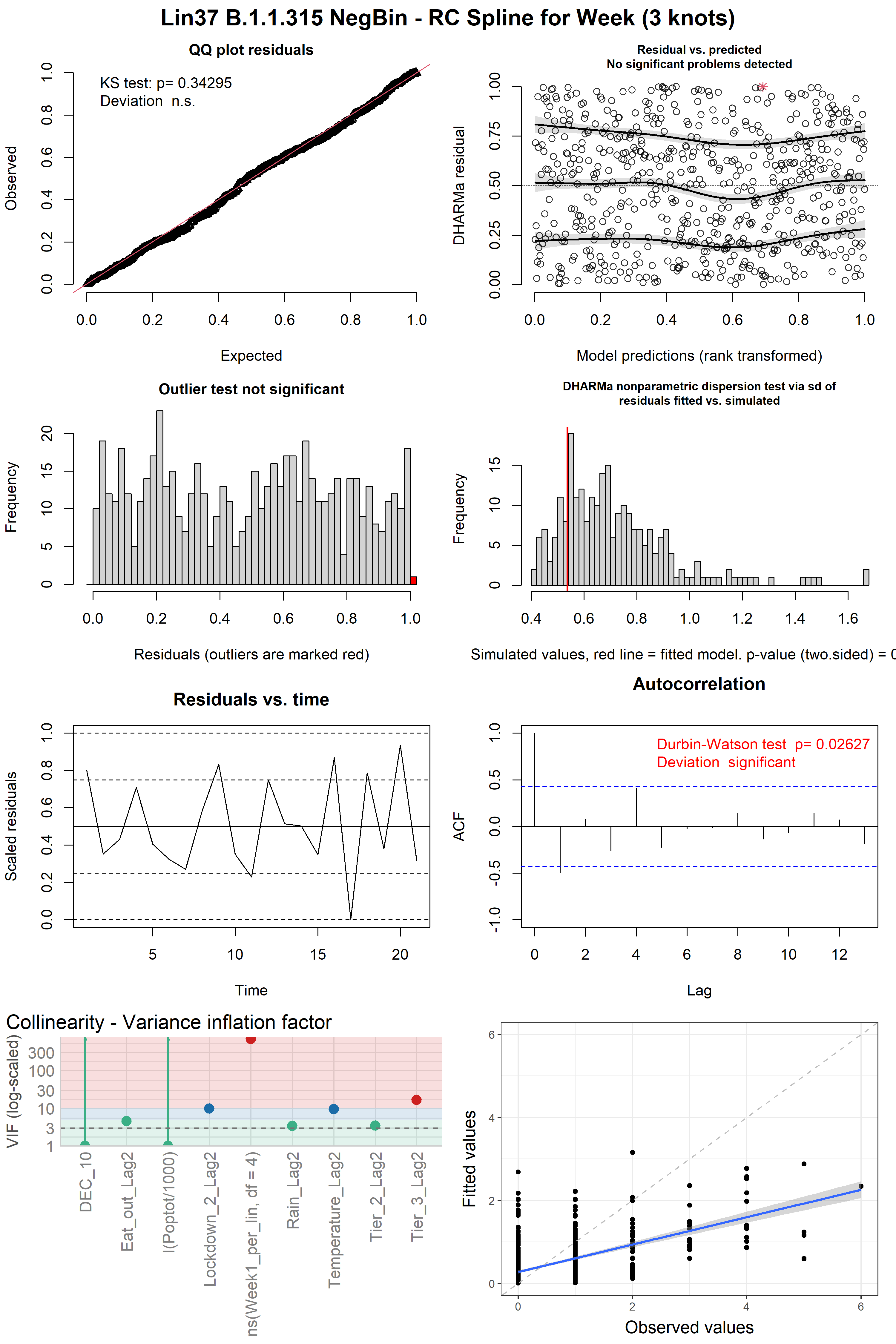

Supplement: Supplementary file: main dataset and code (compressed) [file EMS198536-supplement-Supplementary_file__main_dataset_and_code__compressed_.zip › Covid-19-Teesside-main/Figures/GLMM/Lin37/Lin37-B11315_NB_RCS-Week-3knots_Fit.png]

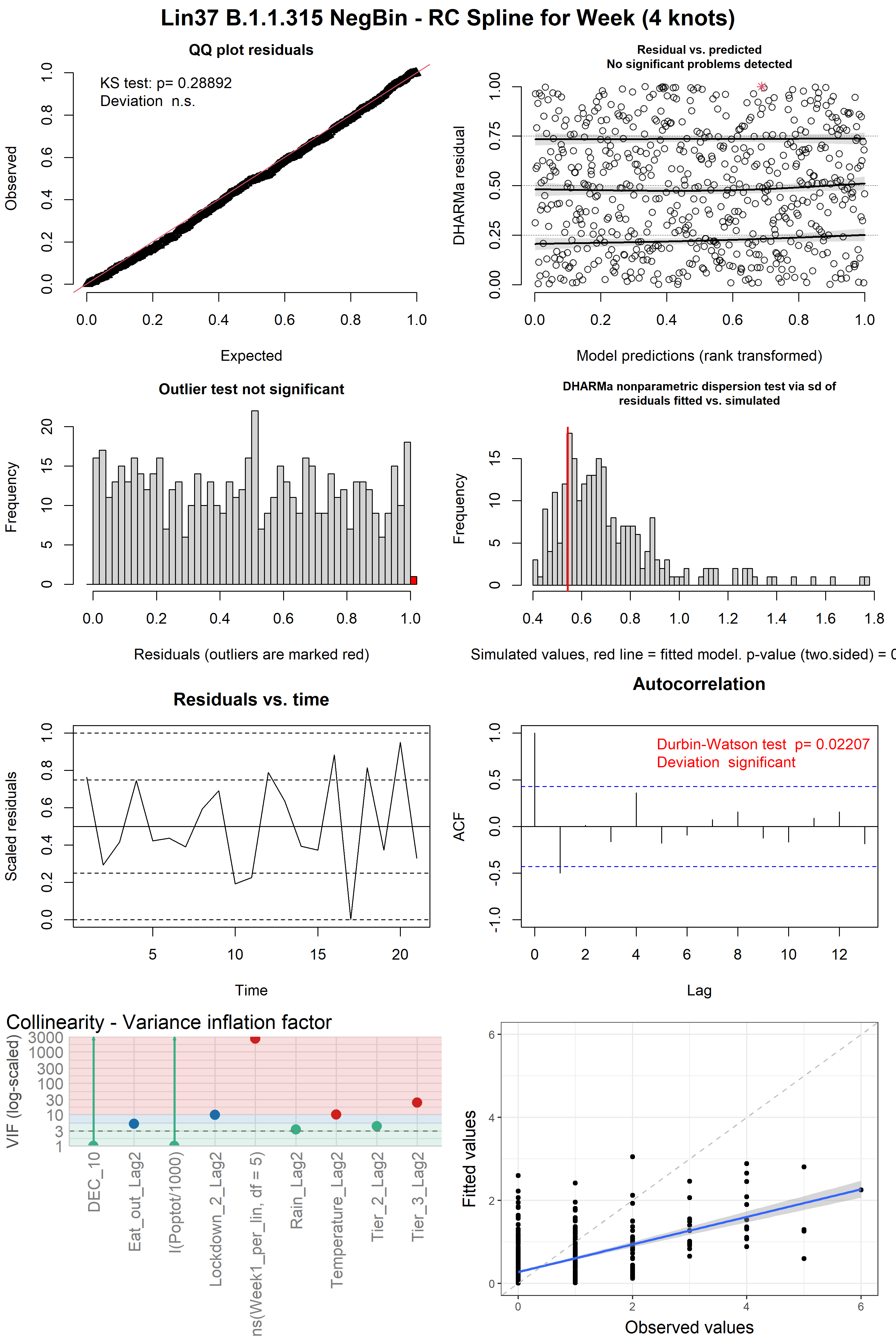

Supplement: Supplementary file: main dataset and code (compressed) [file EMS198536-supplement-Supplementary_file__main_dataset_and_code__compressed_.zip › Covid-19-Teesside-main/Figures/GLMM/Lin37/Lin37-B11315_NB_RCS-Week-4knots_Fit.png]

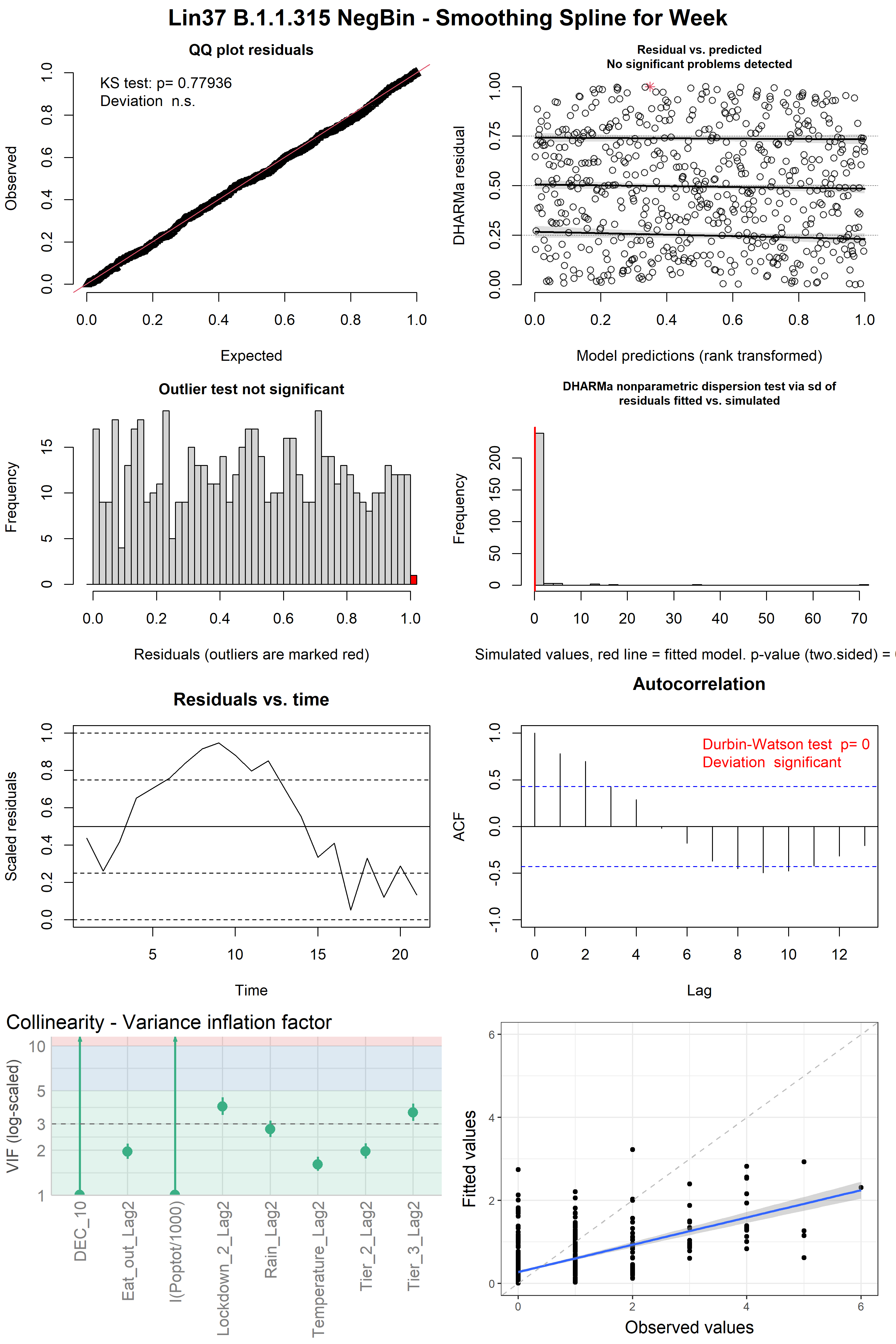

Supplement: Supplementary file: main dataset and code (compressed) [file EMS198536-supplement-Supplementary_file__main_dataset_and_code__compressed_.zip › Covid-19-Teesside-main/Figures/GLMM/Lin37/Lin37-B11315_NB_SmoothSpline-Week-TPS_Fit.png]

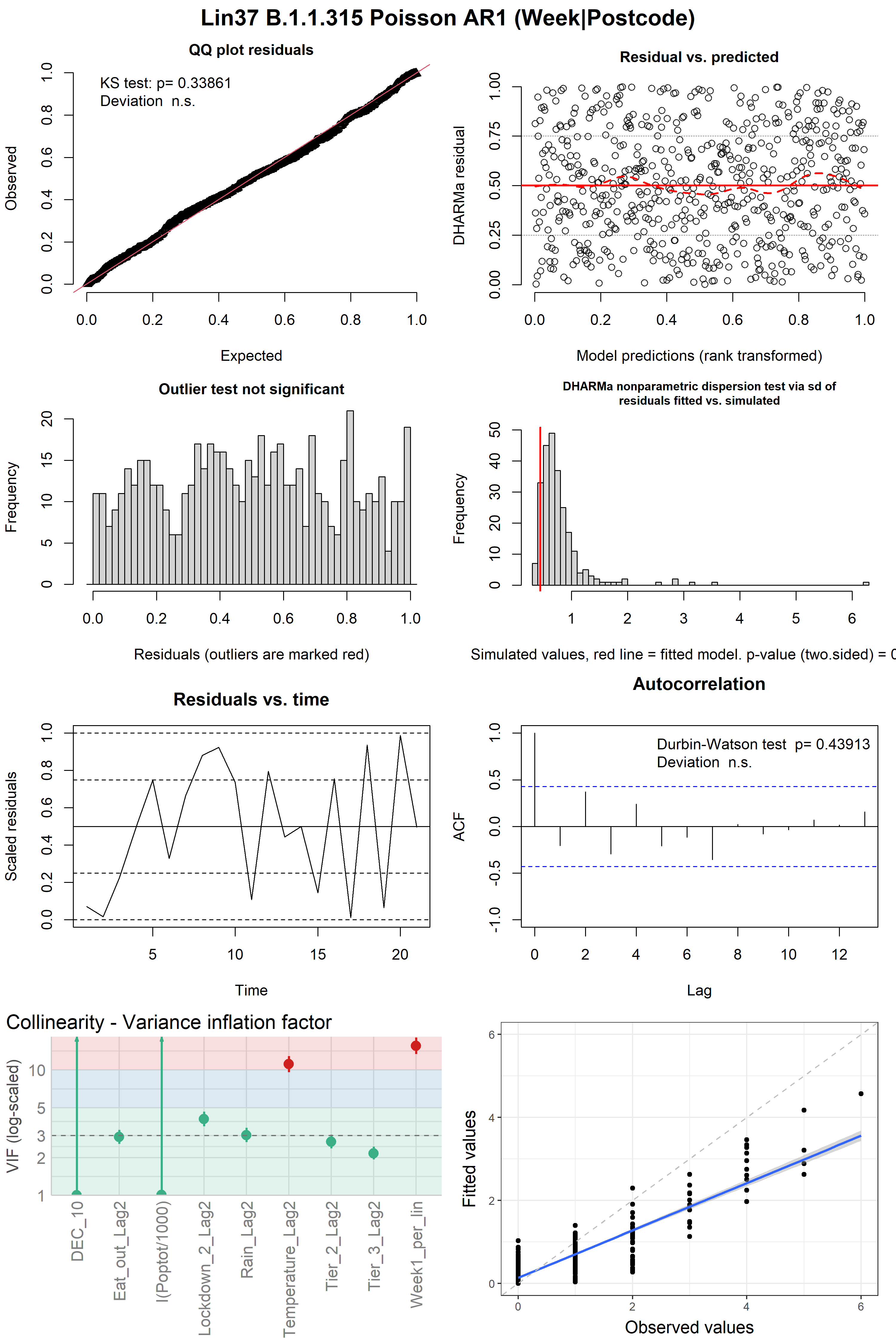

Supplement: Supplementary file: main dataset and code (compressed) [file EMS198536-supplement-Supplementary_file__main_dataset_and_code__compressed_.zip › Covid-19-Teesside-main/Figures/GLMM/Lin37/Lin37-B11315_Po_AR1-Week-Postcode_Fit.png]

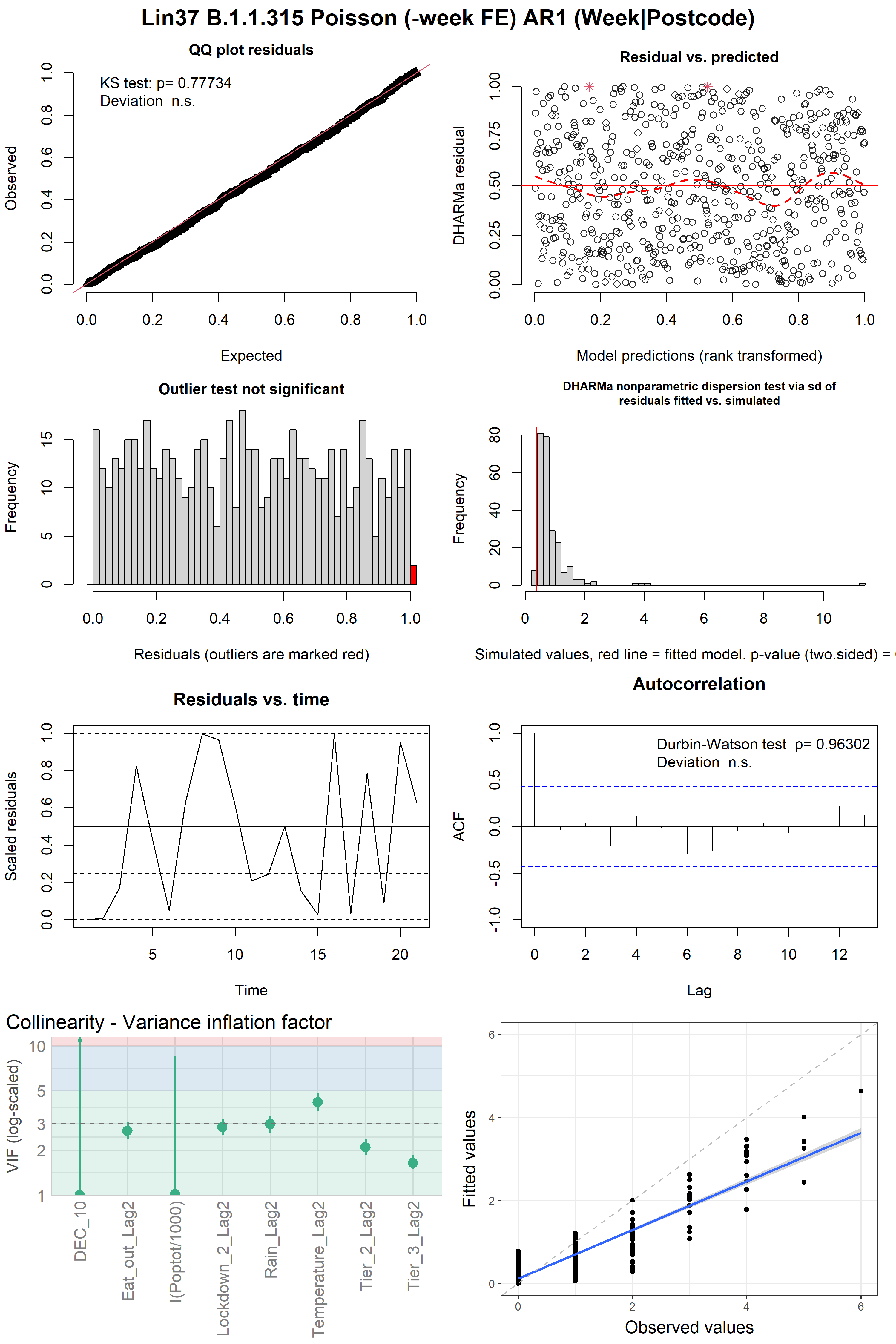

Supplement: Supplementary file: main dataset and code (compressed) [file EMS198536-supplement-Supplementary_file__main_dataset_and_code__compressed_.zip › Covid-19-Teesside-main/Figures/GLMM/Lin37/Lin37-B11315_Po_AR1-Week-Postcode_No-week-FE_Fit.png]

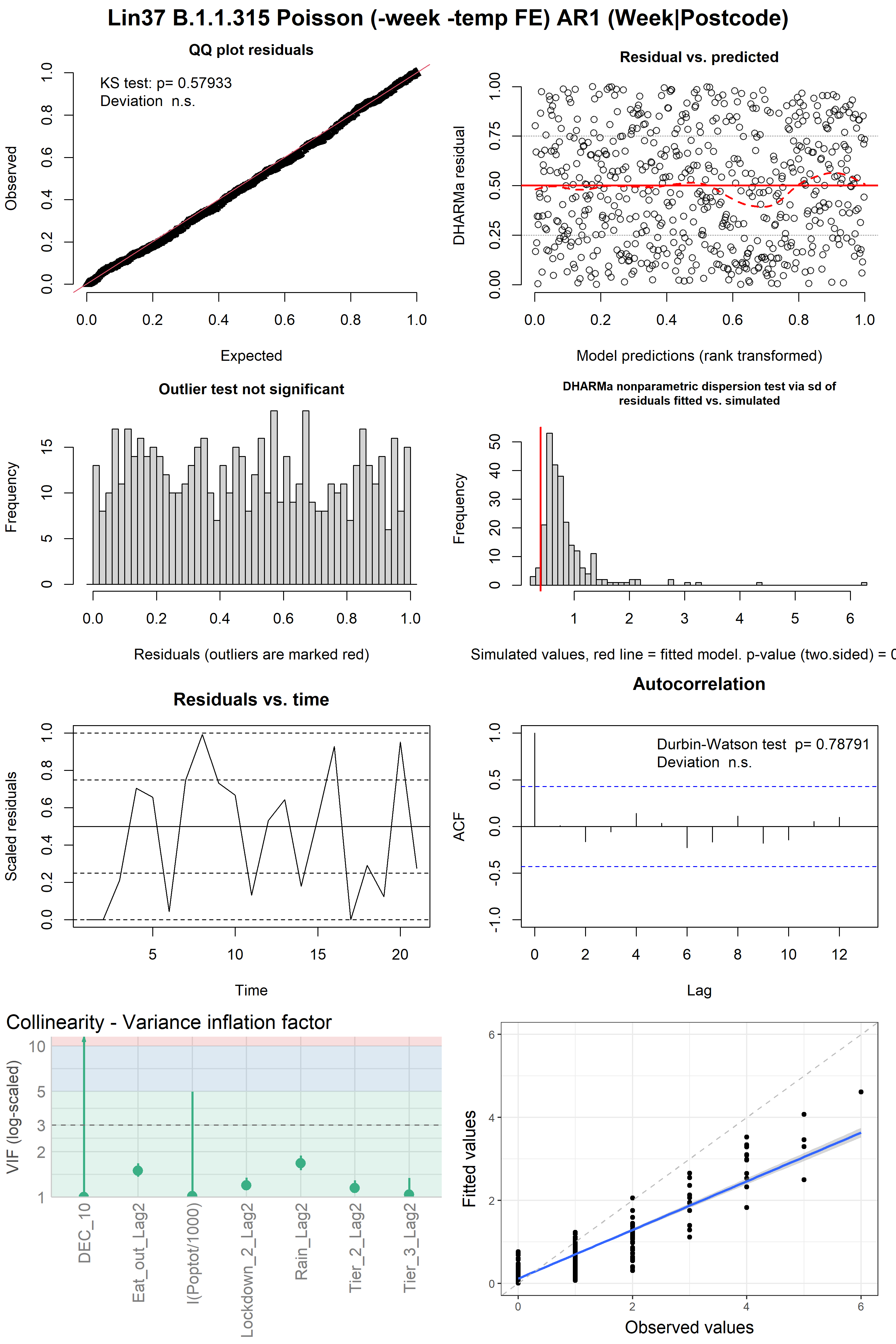

Supplement: Supplementary file: main dataset and code (compressed) [file EMS198536-supplement-Supplementary_file__main_dataset_and_code__compressed_.zip › Covid-19-Teesside-main/Figures/GLMM/Lin37/Lin37-B11315_Po_AR1-Week-Postcode_No-week-no-temp-FE_Fit.png]

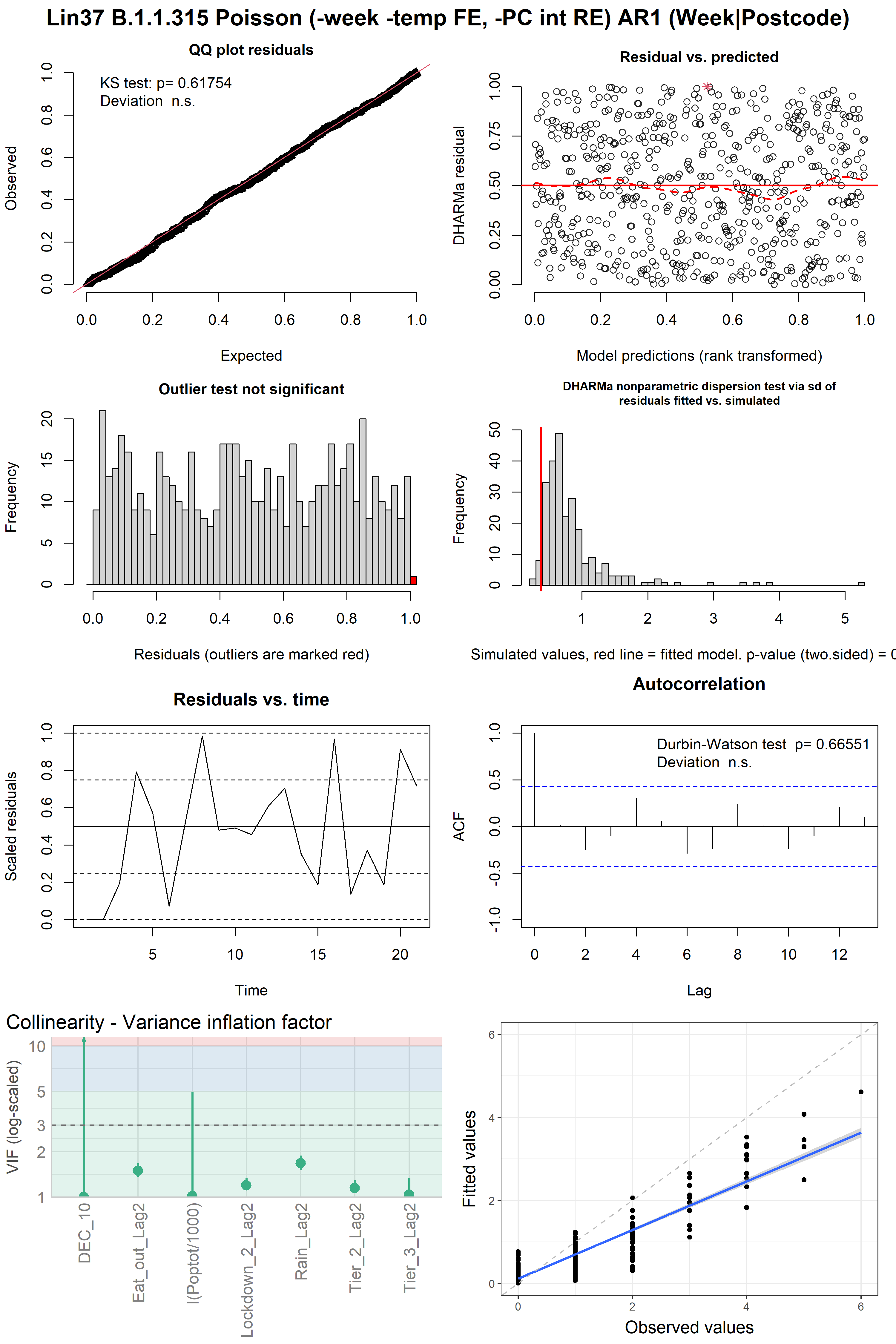

Supplement: Supplementary file: main dataset and code (compressed) [file EMS198536-supplement-Supplementary_file__main_dataset_and_code__compressed_.zip › Covid-19-Teesside-main/Figures/GLMM/Lin37/Lin37-B11315_Po_AR1-Week-Postcode_No-week-no-temp-FE_No-PC-int-RE_Fit.png]

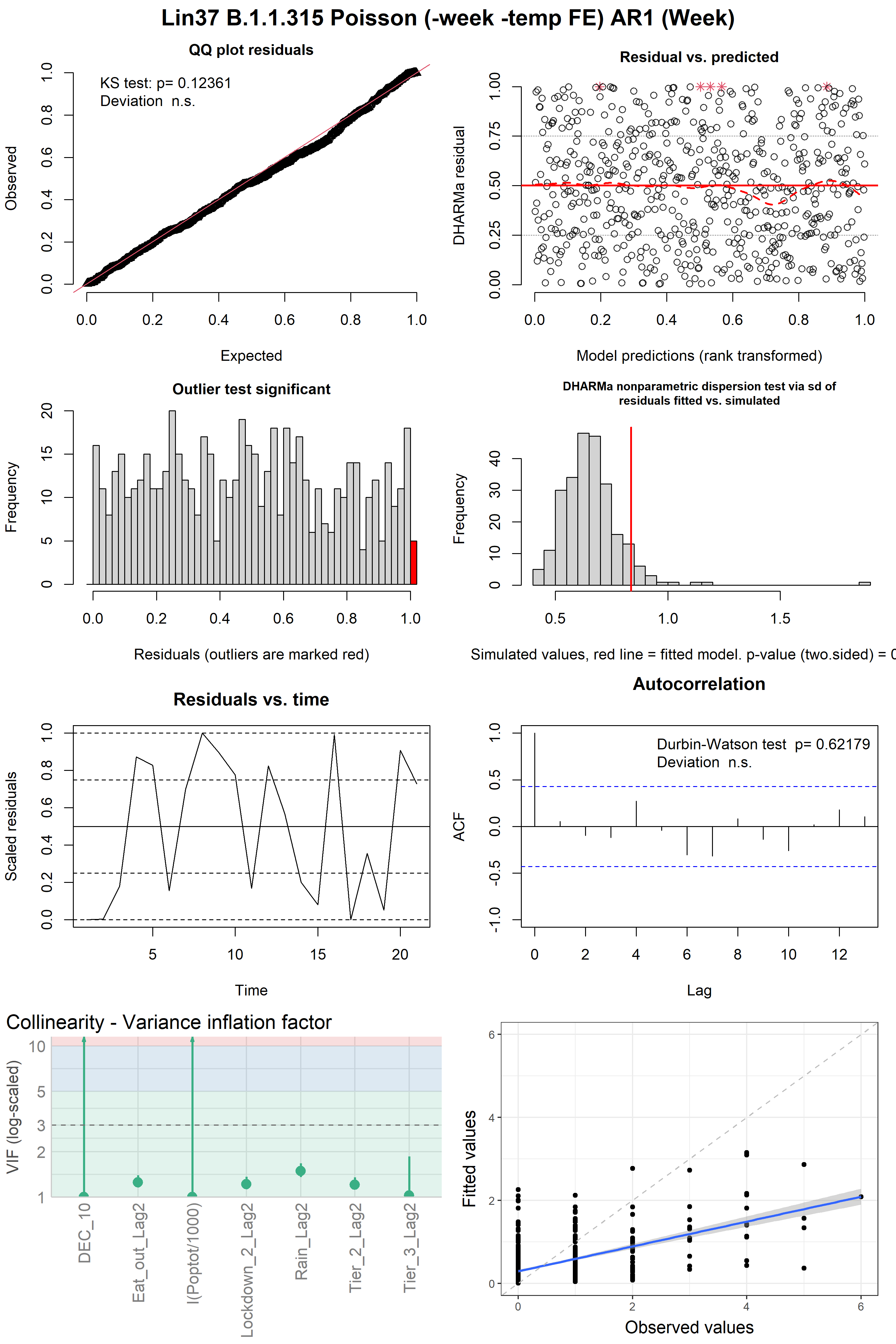

Supplement: Supplementary file: main dataset and code (compressed) [file EMS198536-supplement-Supplementary_file__main_dataset_and_code__compressed_.zip › Covid-19-Teesside-main/Figures/GLMM/Lin37/Lin37-B11315_Po_AR1-Week_No-week-no-temp-FE_Fit.png]

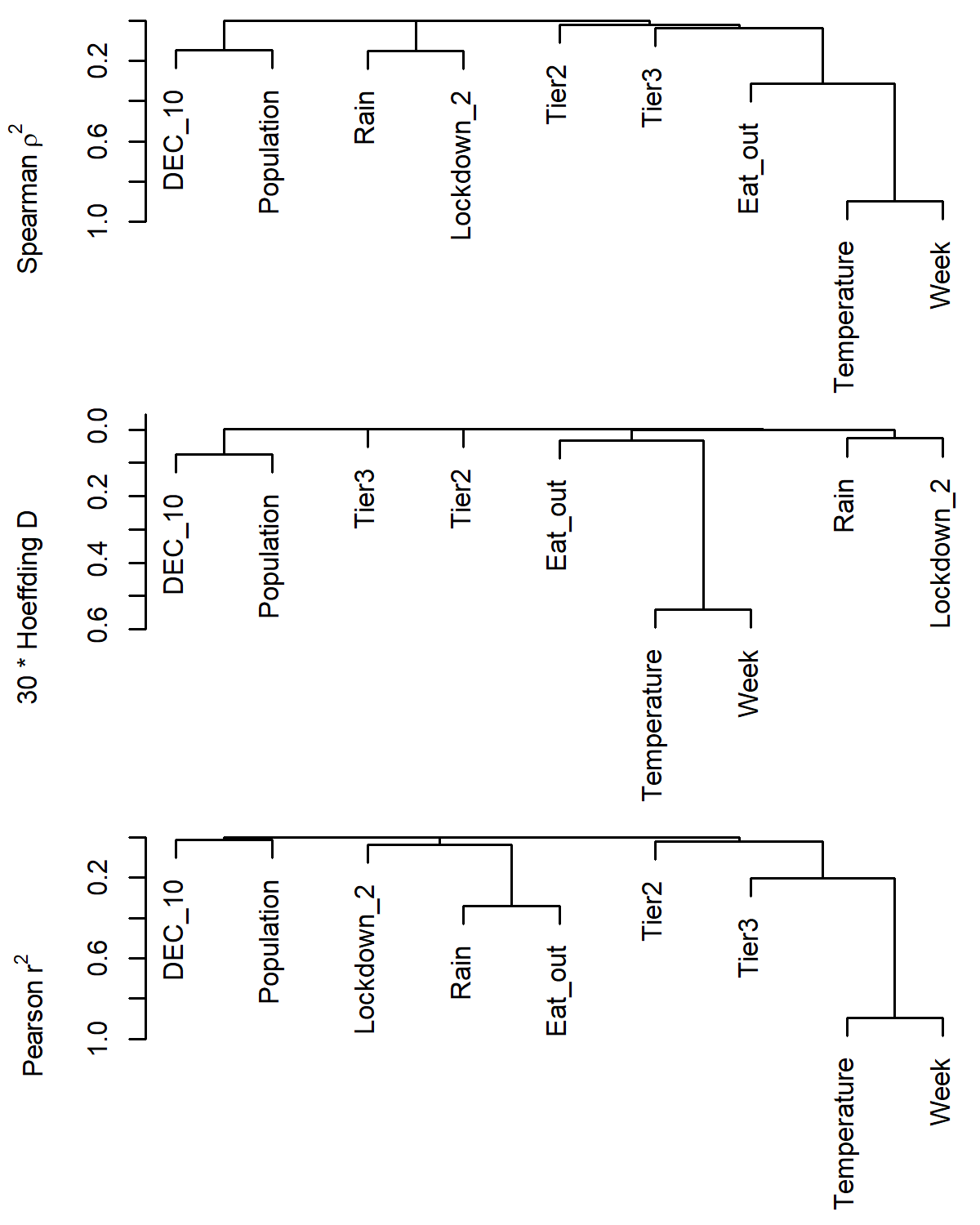

Supplement: Supplementary file: main dataset and code (compressed) [file EMS198536-supplement-Supplementary_file__main_dataset_and_code__compressed_.zip › Covid-19-Teesside-main/Figures/GLMM/Lin37/Lin37-B11315_Variable-Clustering.png]

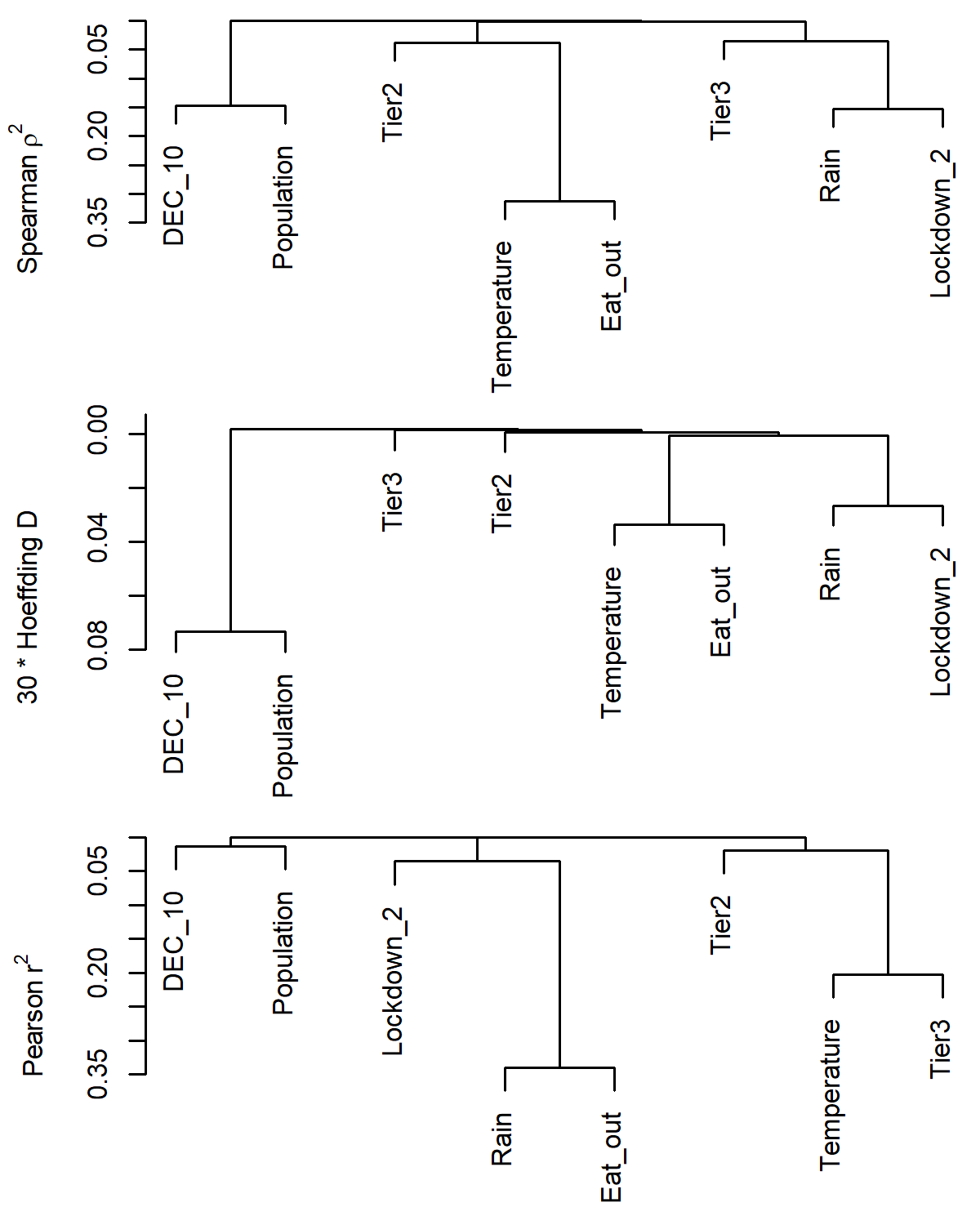

Supplement: Supplementary file: main dataset and code (compressed) [file EMS198536-supplement-Supplementary_file__main_dataset_and_code__compressed_.zip › Covid-19-Teesside-main/Figures/GLMM/Lin37/Lin37-B11315_Variable-Clustering_Without-Week.png]

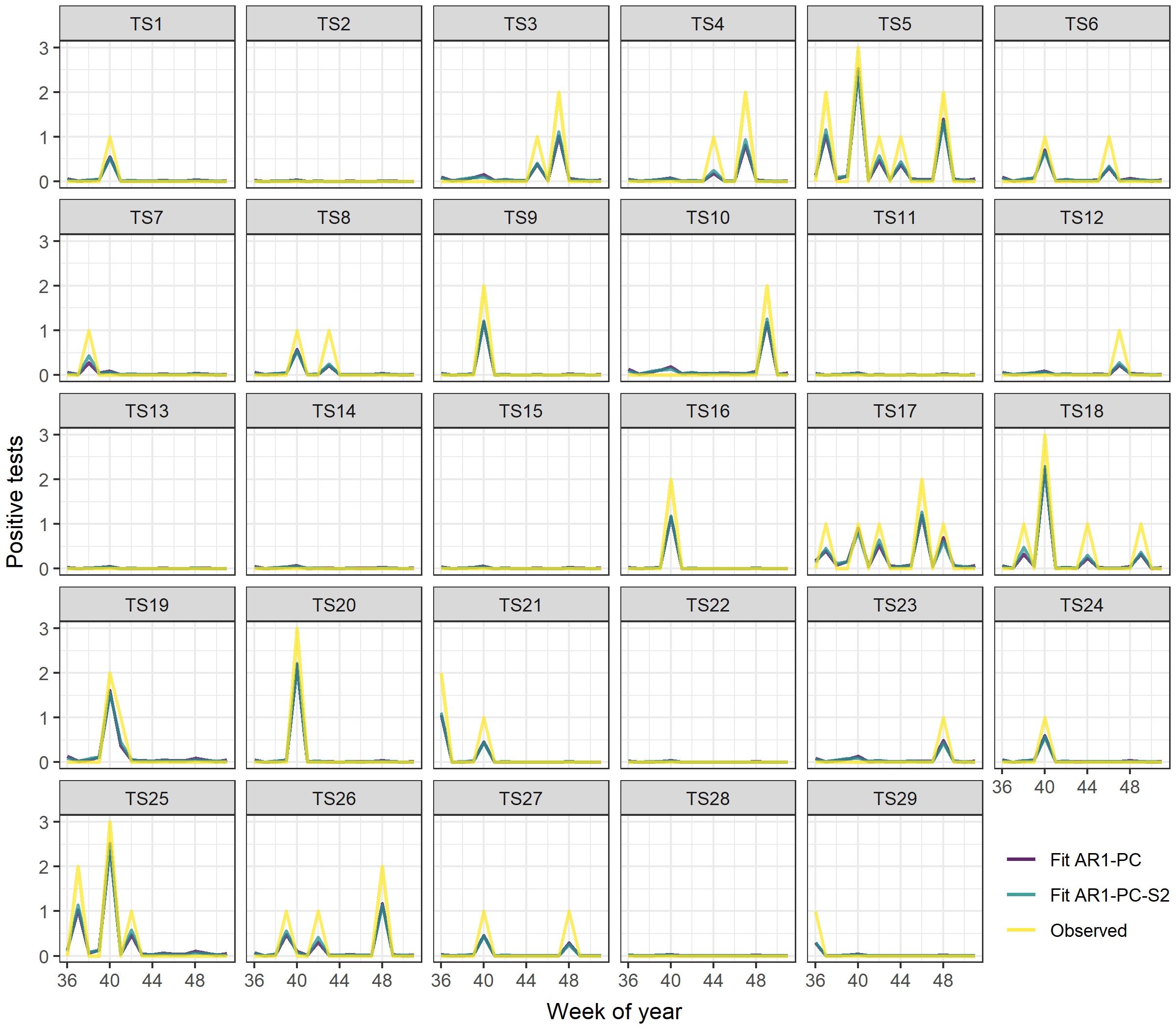

Supplement: Supplementary file: main dataset and code (compressed) [file EMS198536-supplement-Supplementary_file__main_dataset_and_code__compressed_.zip › Covid-19-Teesside-main/Figures/GLMM/Lin39/Lin39-B1137_GLMM_Obs-vs-Fit_AR1PC-AR1PCS2.png]

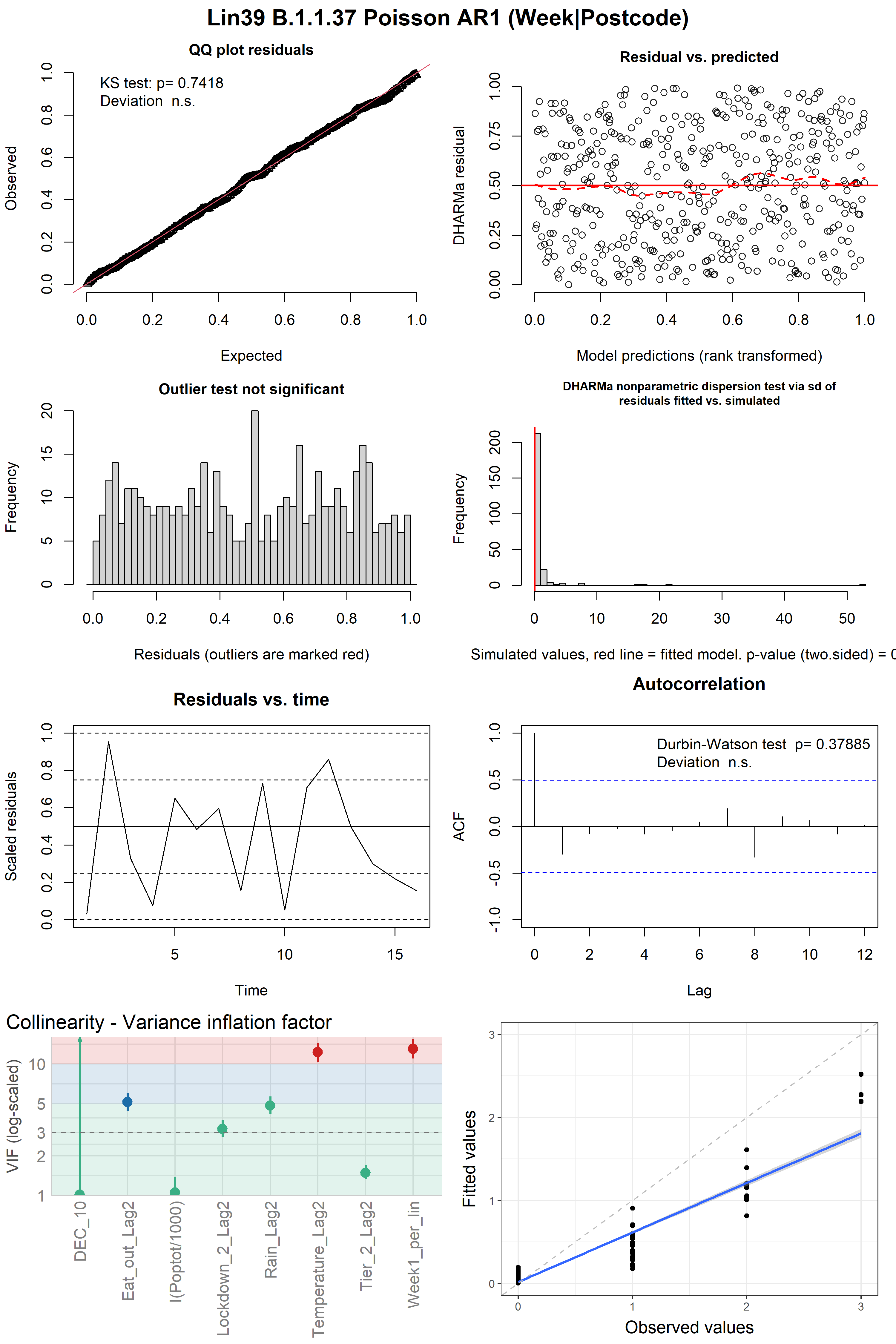

Supplement: Supplementary file: main dataset and code (compressed) [file EMS198536-supplement-Supplementary_file__main_dataset_and_code__compressed_.zip › Covid-19-Teesside-main/Figures/GLMM/Lin39/Lin39-B1137_Po_AR1-Week-Postcode_Fit.png]

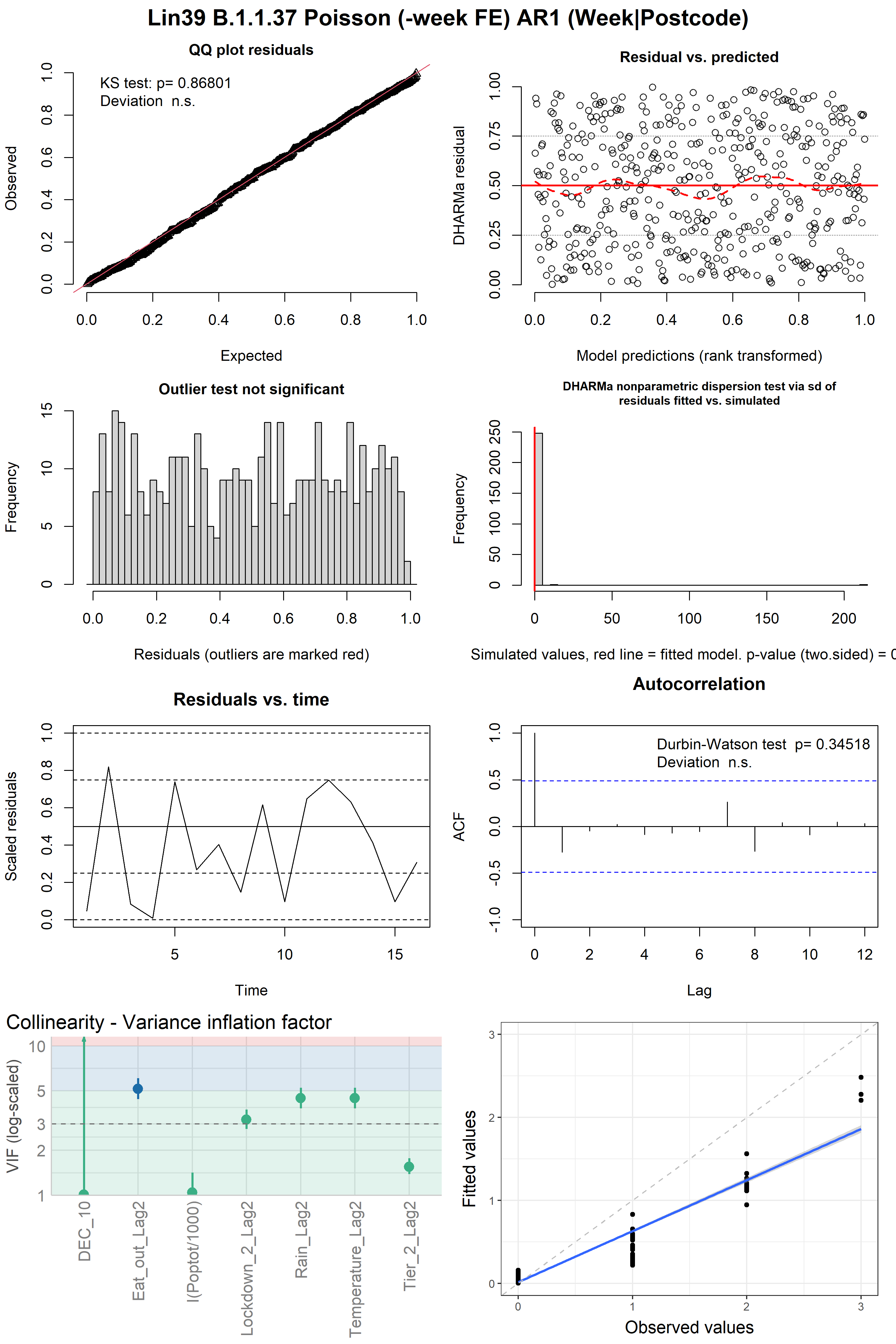

Supplement: Supplementary file: main dataset and code (compressed) [file EMS198536-supplement-Supplementary_file__main_dataset_and_code__compressed_.zip › Covid-19-Teesside-main/Figures/GLMM/Lin39/Lin39-B1137_Po_AR1-Week-Postcode_No-week-FE_Fit.png]

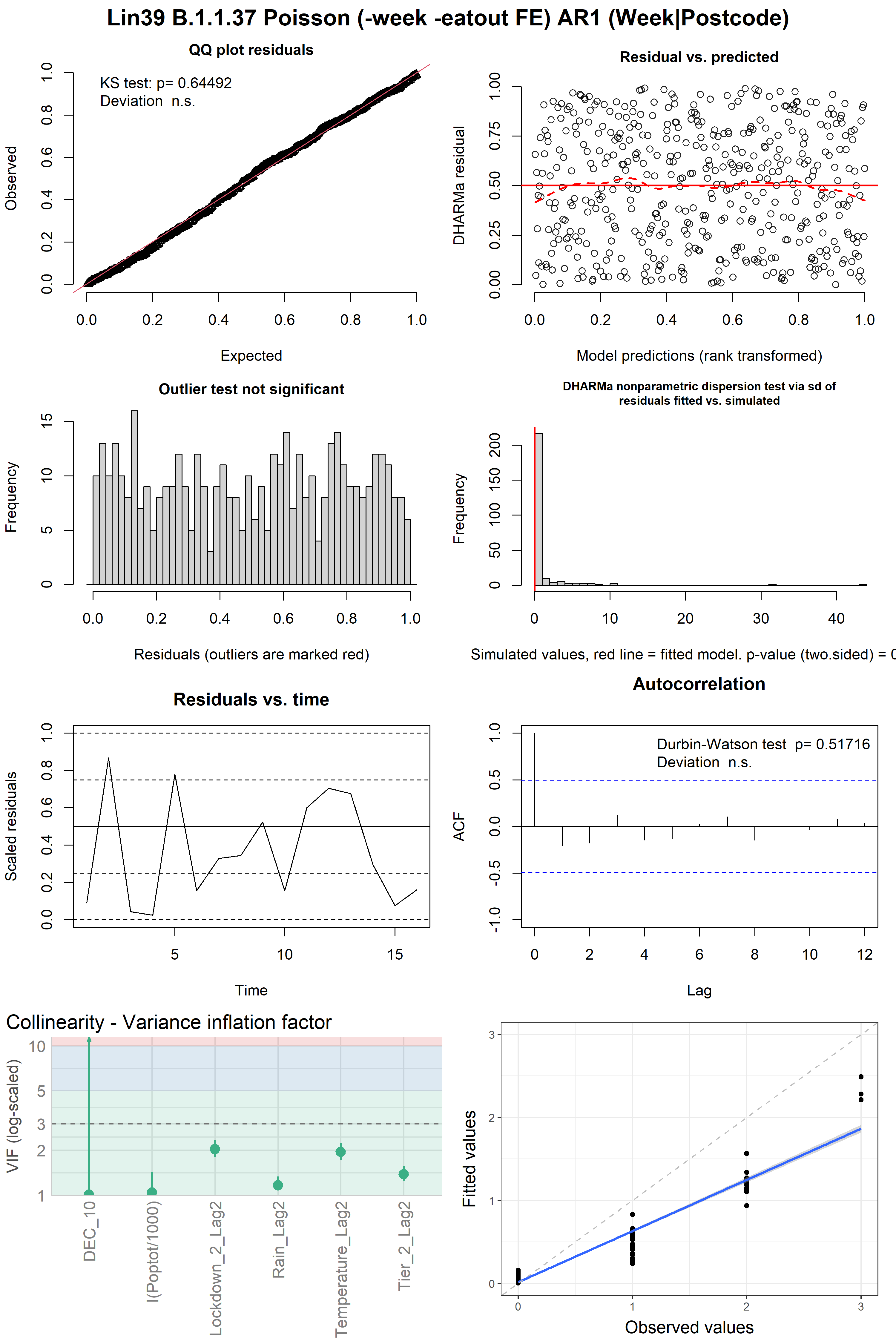

Supplement: Supplementary file: main dataset and code (compressed) [file EMS198536-supplement-Supplementary_file__main_dataset_and_code__compressed_.zip › Covid-19-Teesside-main/Figures/GLMM/Lin39/Lin39-B1137_Po_AR1-Week-Postcode_No-week-no-eatout-FE_Fit.png]

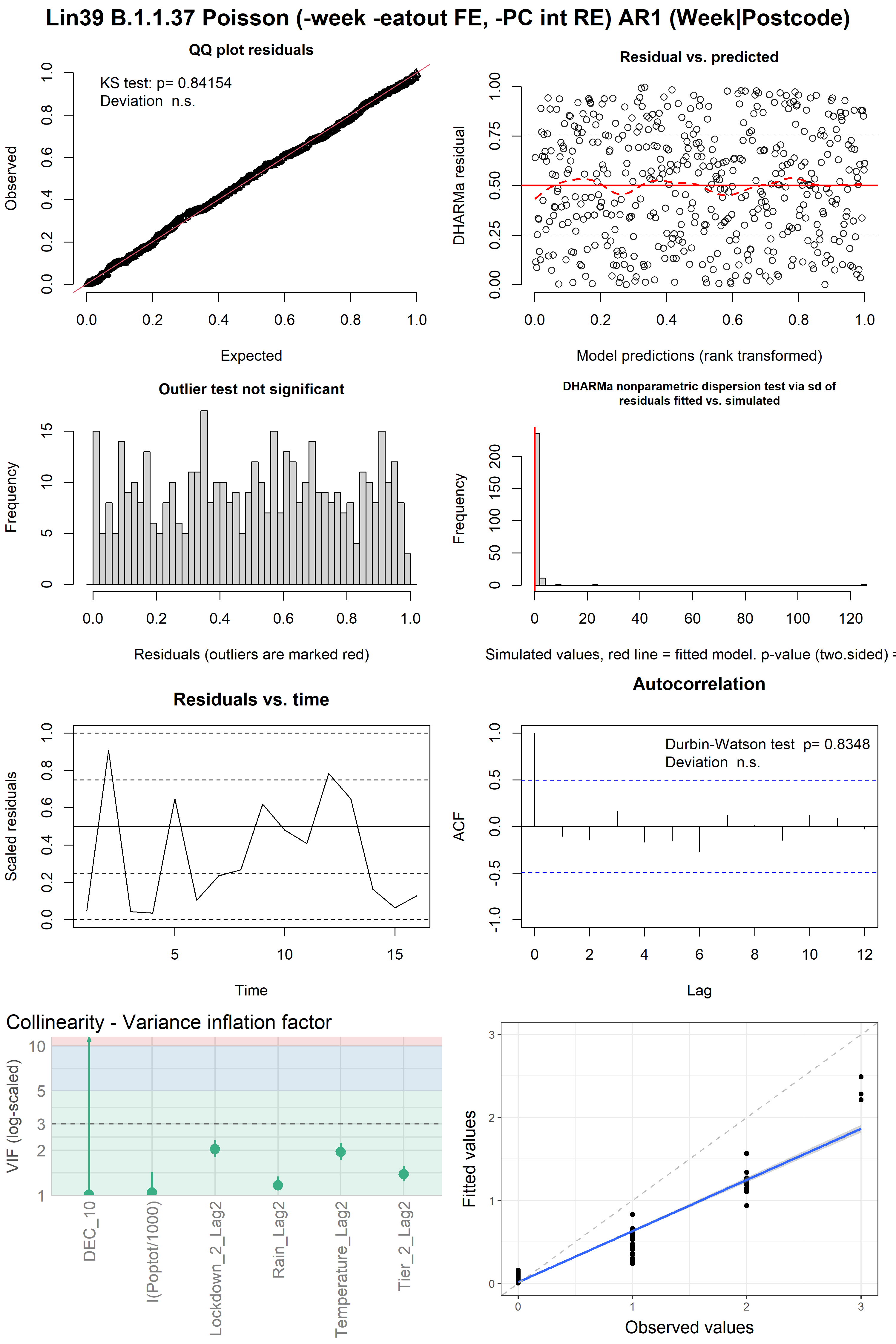

Supplement: Supplementary file: main dataset and code (compressed) [file EMS198536-supplement-Supplementary_file__main_dataset_and_code__compressed_.zip › Covid-19-Teesside-main/Figures/GLMM/Lin39/Lin39-B1137_Po_AR1-Week-Postcode_No-week-no-eatout-FE_No-PC-int-RE_Fit.png]

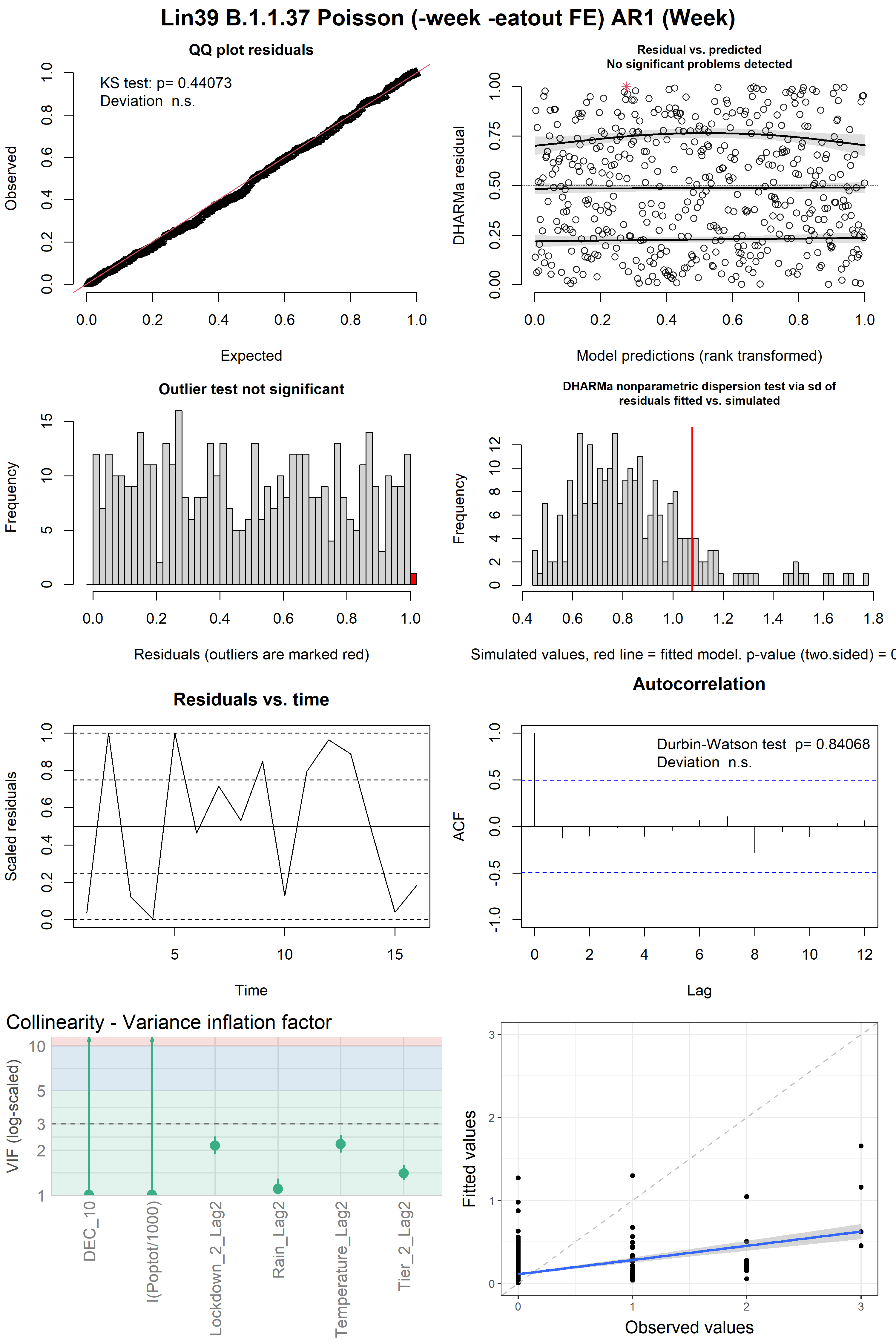

Supplement: Supplementary file: main dataset and code (compressed) [file EMS198536-supplement-Supplementary_file__main_dataset_and_code__compressed_.zip › Covid-19-Teesside-main/Figures/GLMM/Lin39/Lin39-B1137_Po_AR1-Week_No-week-no-eatout-FE_Fit.png]

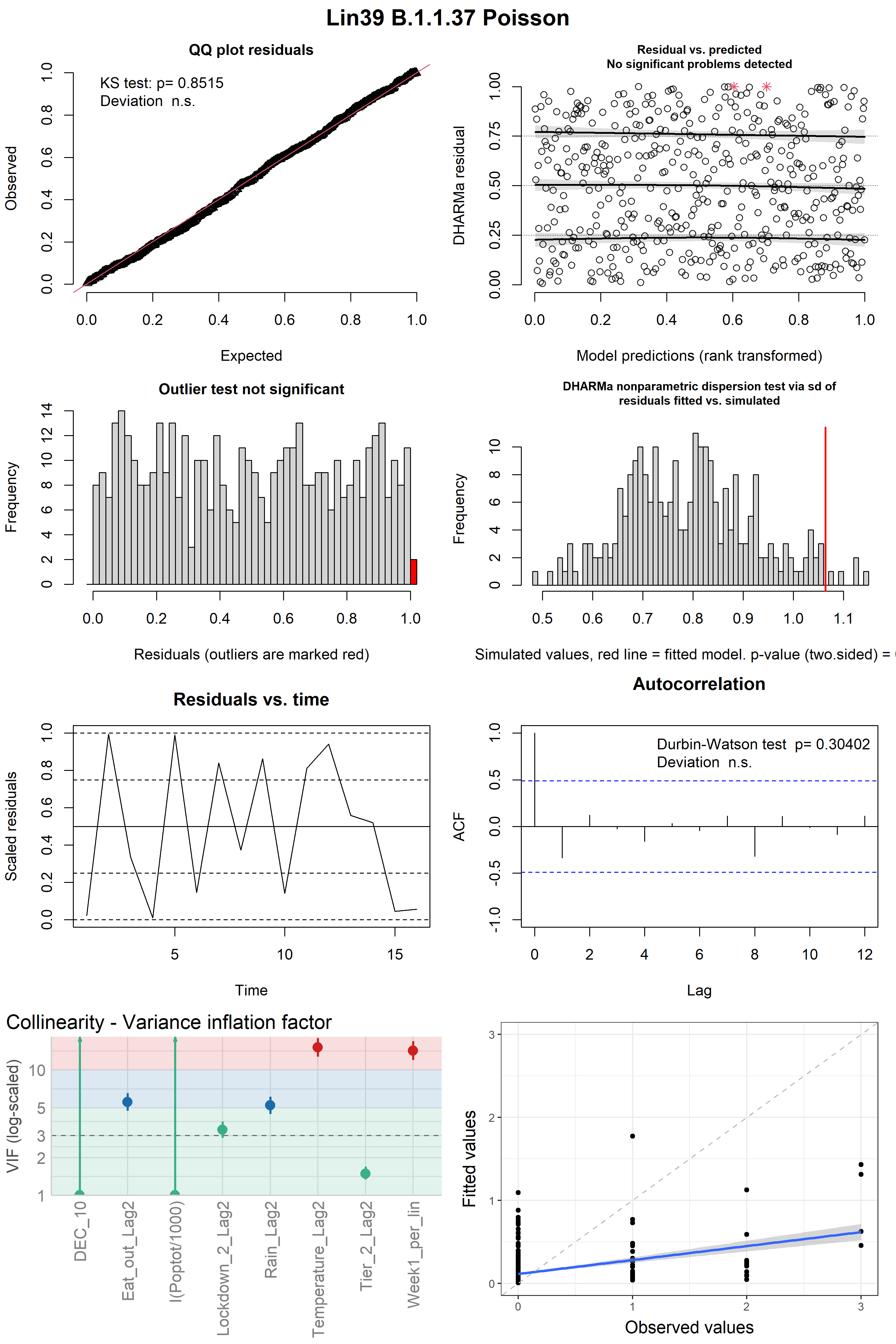

Supplement: Supplementary file: main dataset and code (compressed) [file EMS198536-supplement-Supplementary_file__main_dataset_and_code__compressed_.zip › Covid-19-Teesside-main/Figures/GLMM/Lin39/Lin39-B1137_Po_Full_Fit.png]

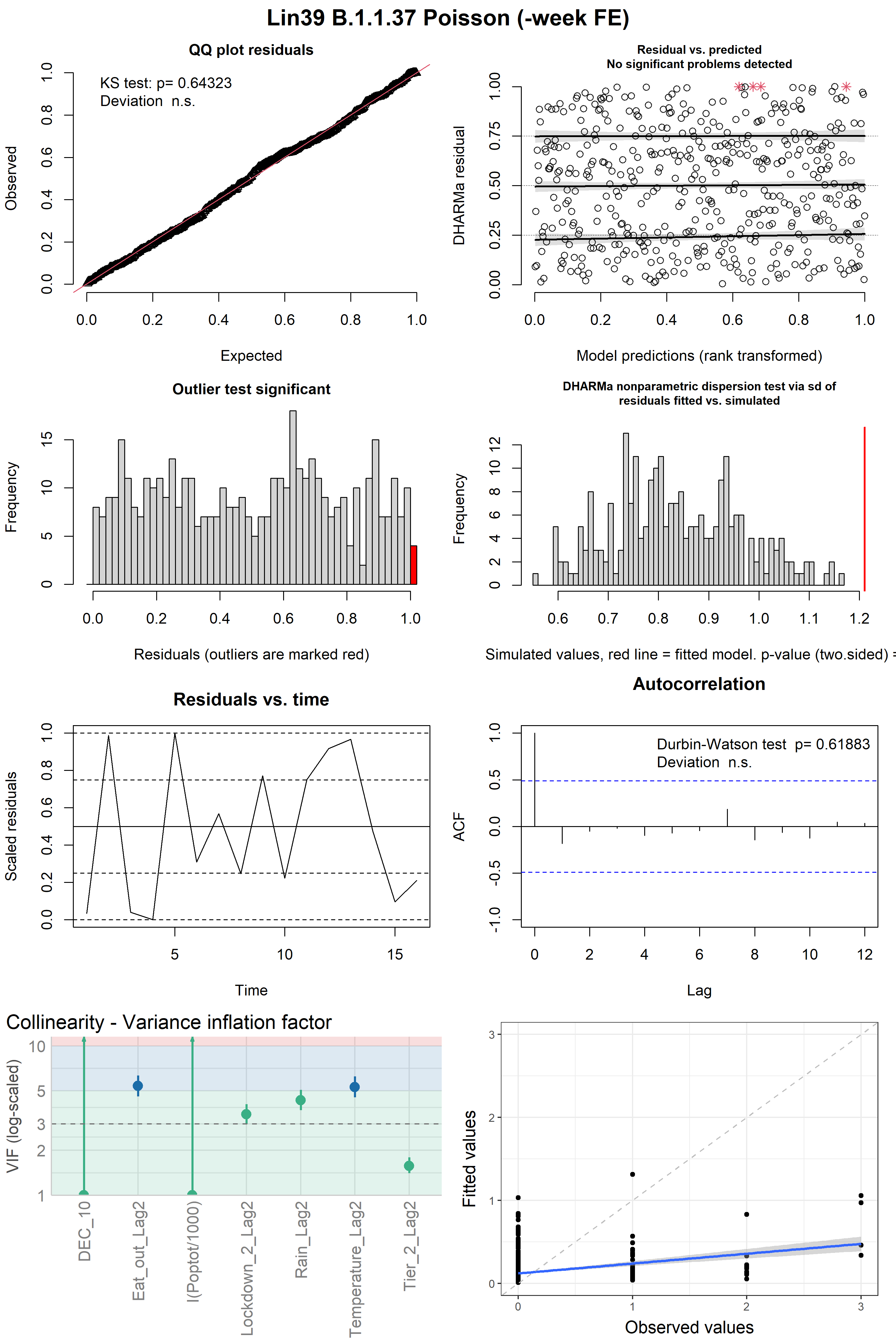

Supplement: Supplementary file: main dataset and code (compressed) [file EMS198536-supplement-Supplementary_file__main_dataset_and_code__compressed_.zip › Covid-19-Teesside-main/Figures/GLMM/Lin39/Lin39-B1137_Po_No-week-FE_Fit.png]

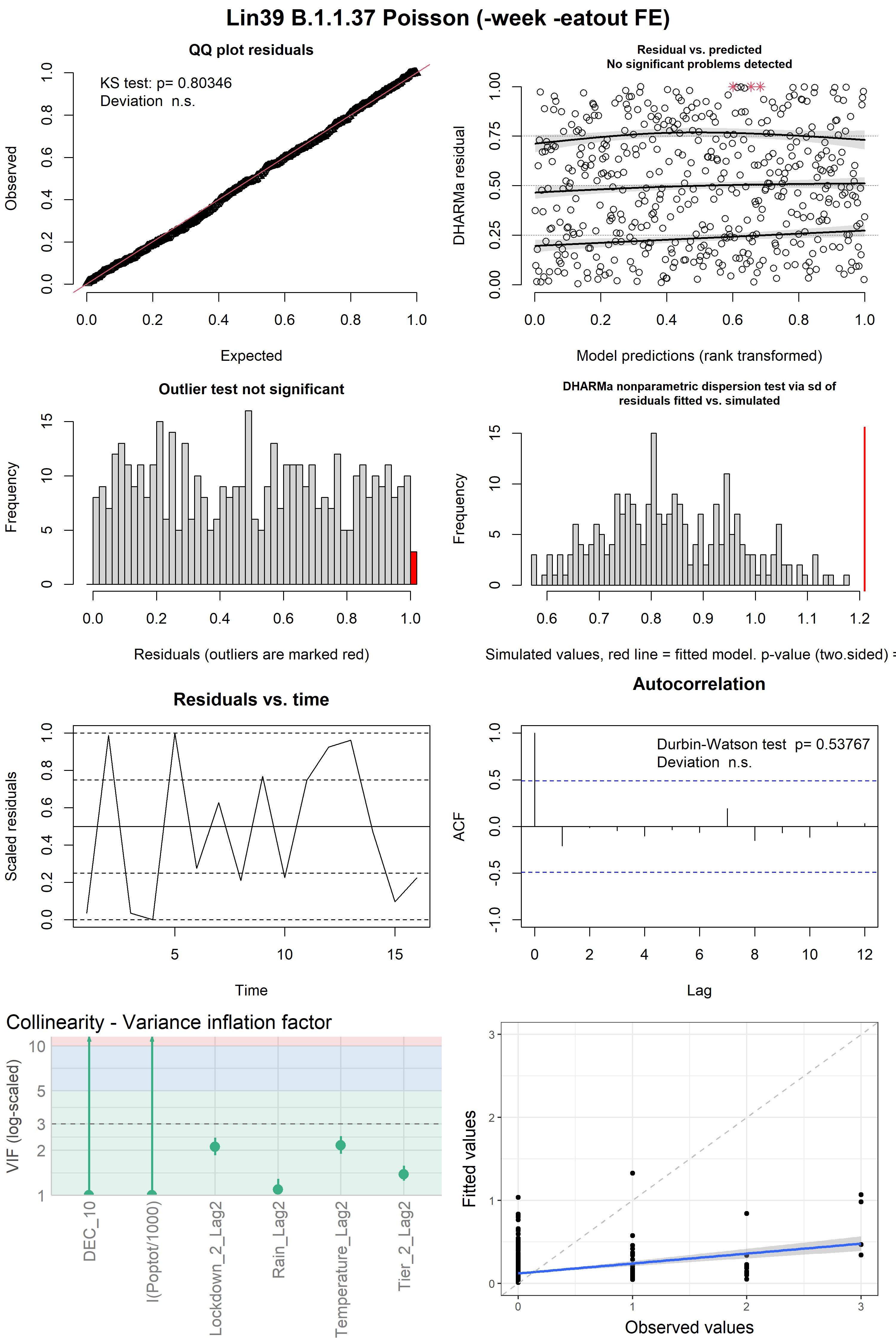

Supplement: Supplementary file: main dataset and code (compressed) [file EMS198536-supplement-Supplementary_file__main_dataset_and_code__compressed_.zip › Covid-19-Teesside-main/Figures/GLMM/Lin39/Lin39-B1137_Po_No-week-no-eatout-FE_Fit.png]

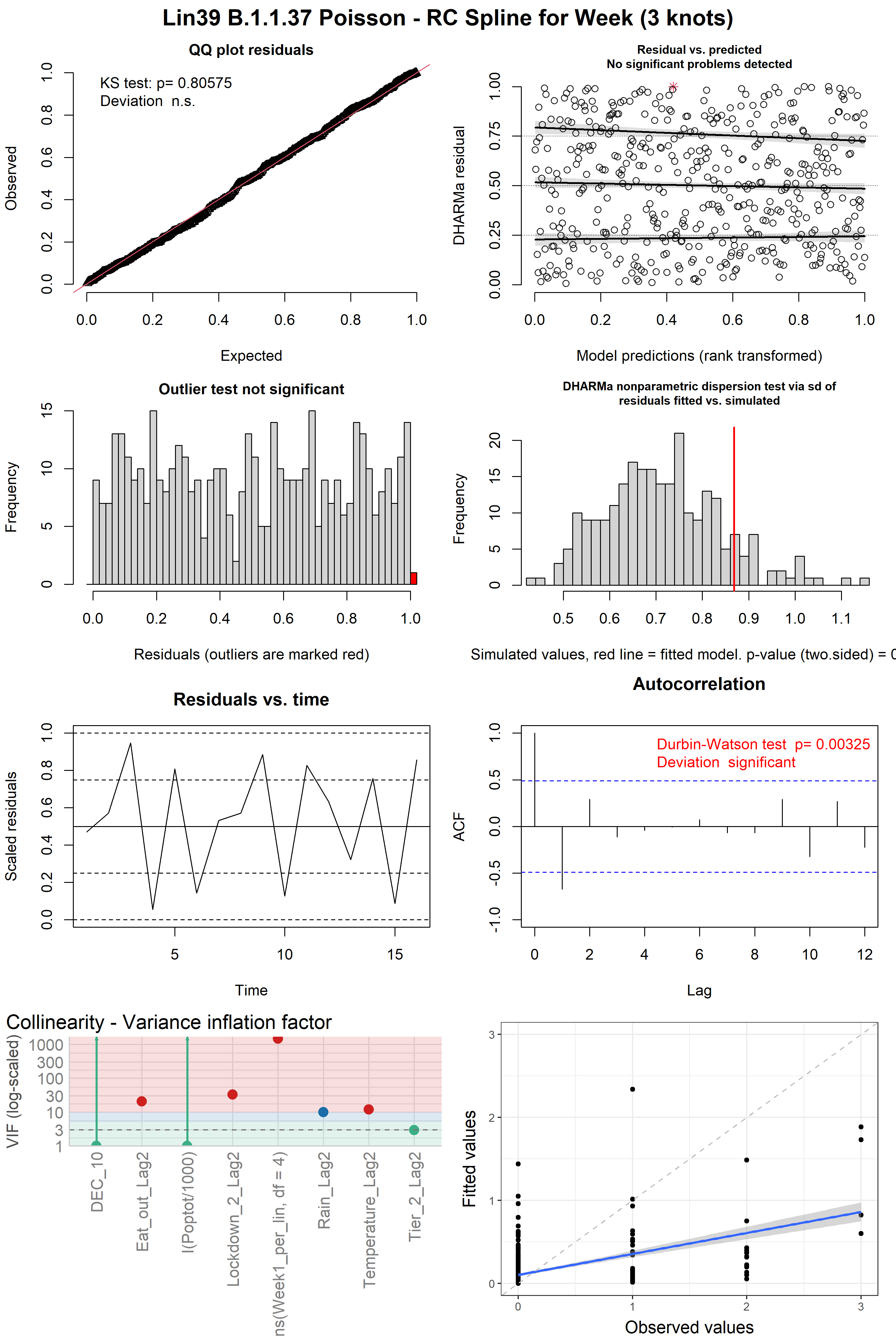

Supplement: Supplementary file: main dataset and code (compressed) [file EMS198536-supplement-Supplementary_file__main_dataset_and_code__compressed_.zip › Covid-19-Teesside-main/Figures/GLMM/Lin39/Lin39-B1137_Po_RCS-Week-3knots_Fit.png]

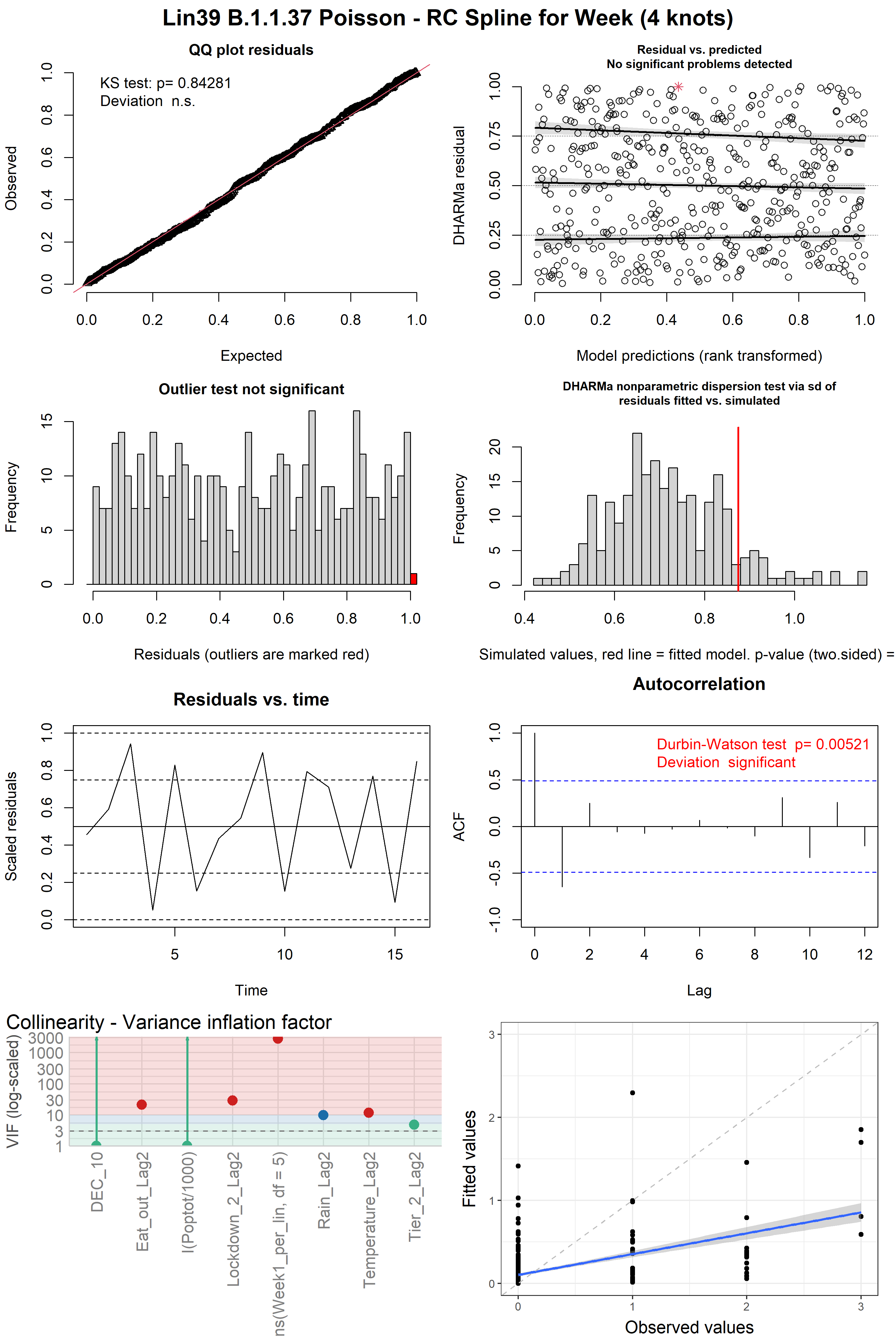

Supplement: Supplementary file: main dataset and code (compressed) [file EMS198536-supplement-Supplementary_file__main_dataset_and_code__compressed_.zip › Covid-19-Teesside-main/Figures/GLMM/Lin39/Lin39-B1137_Po_RCS-Week-4knots_Fit.png]

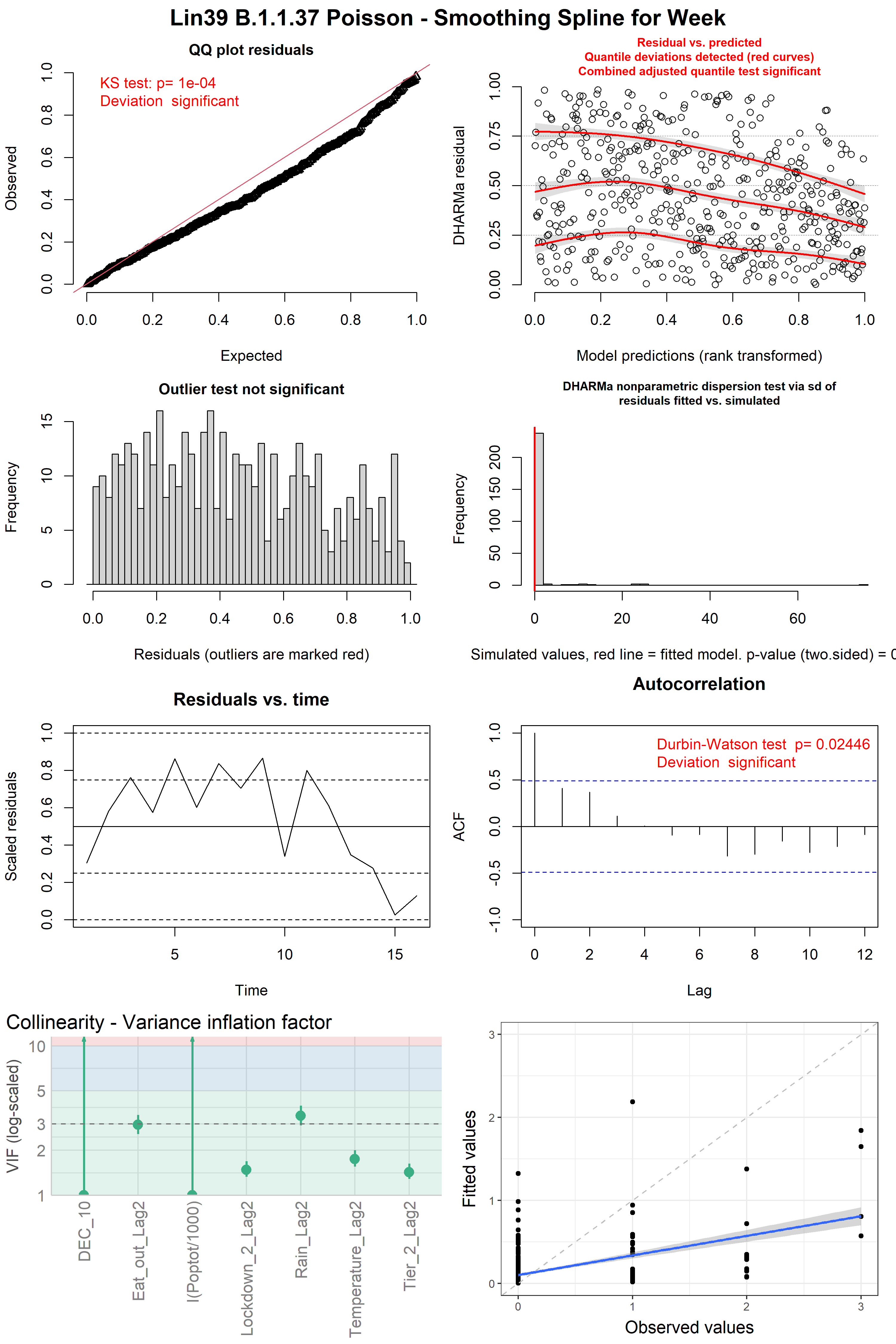

Supplement: Supplementary file: main dataset and code (compressed) [file EMS198536-supplement-Supplementary_file__main_dataset_and_code__compressed_.zip › Covid-19-Teesside-main/Figures/GLMM/Lin39/Lin39-B1137_Po_SmoothSpline-Week-TPS_Fit.png]

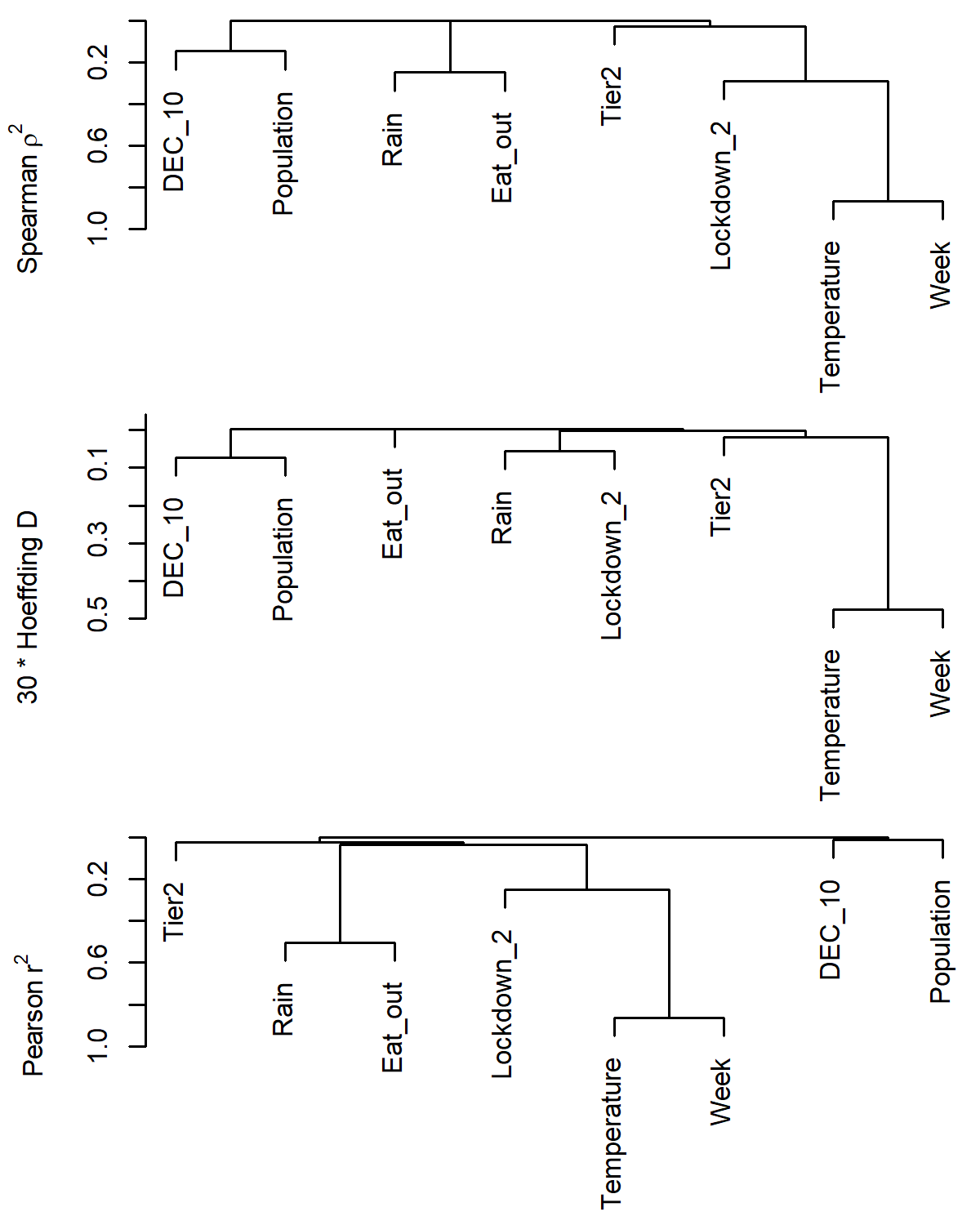

Supplement: Supplementary file: main dataset and code (compressed) [file EMS198536-supplement-Supplementary_file__main_dataset_and_code__compressed_.zip › Covid-19-Teesside-main/Figures/GLMM/Lin39/Lin39-B1137_Variable-Clustering.png]

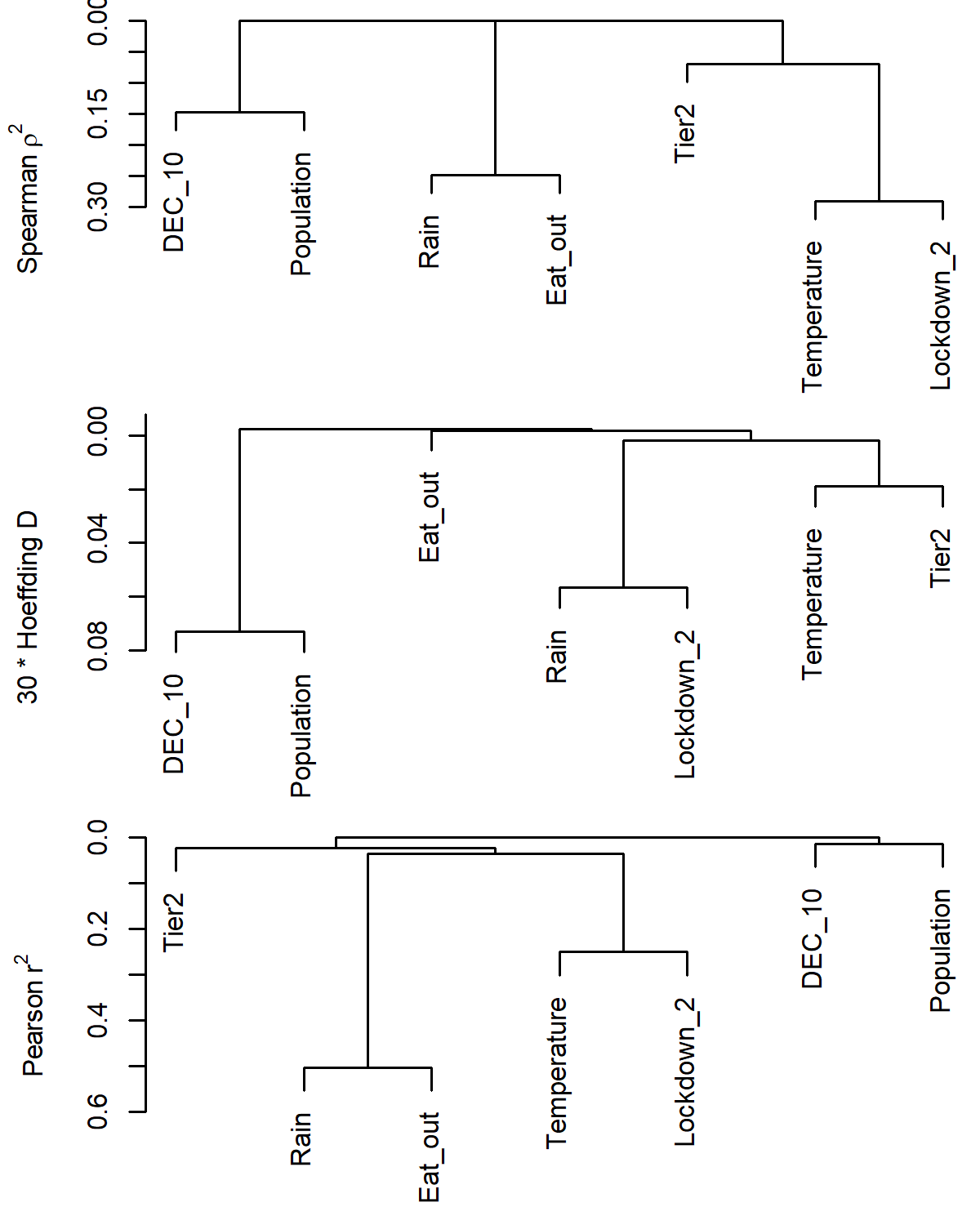

Supplement: Supplementary file: main dataset and code (compressed) [file EMS198536-supplement-Supplementary_file__main_dataset_and_code__compressed_.zip › Covid-19-Teesside-main/Figures/GLMM/Lin39/Lin39-B1137_Variable-Clustering_Without-Week.png]

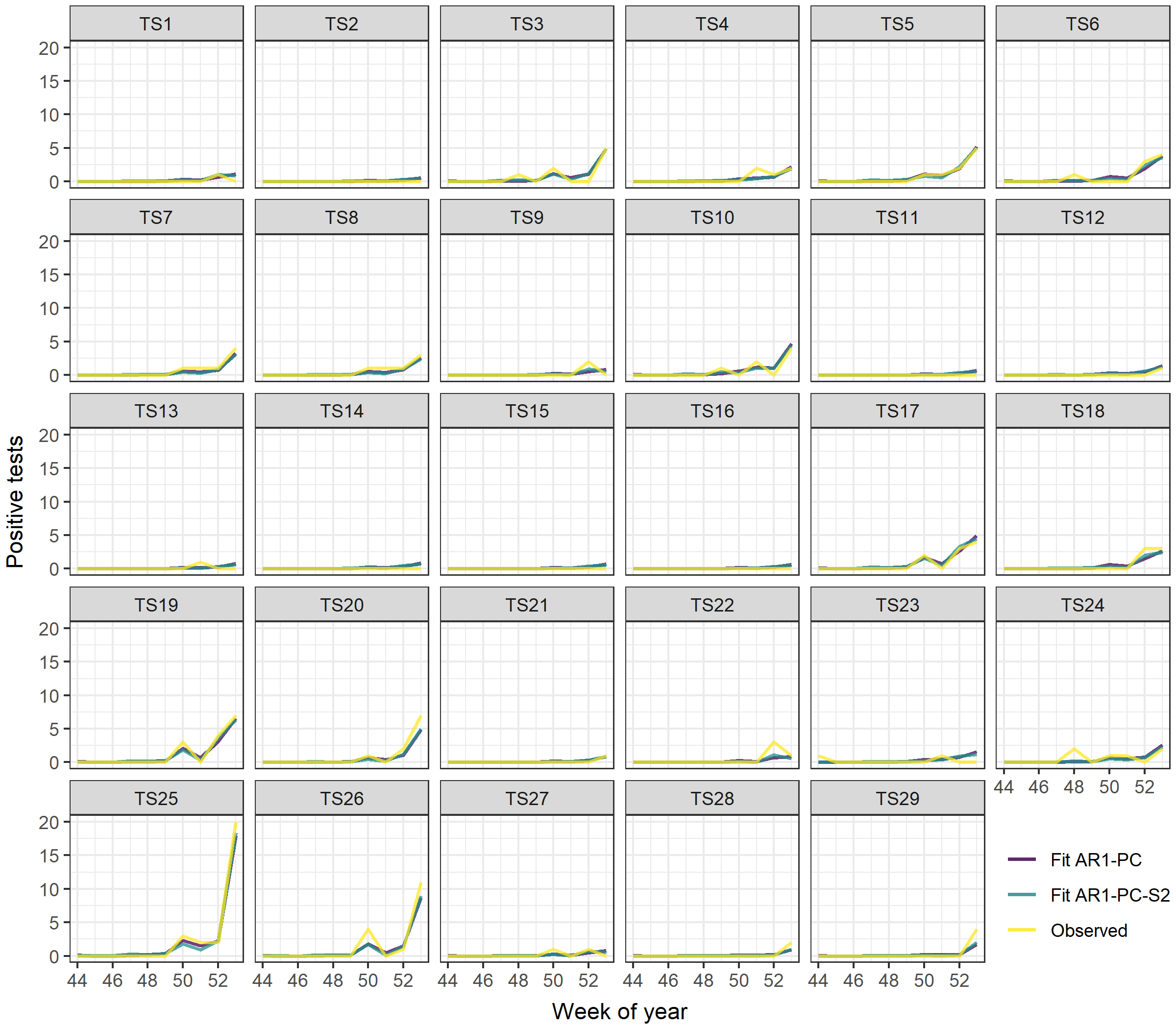

Supplement: Supplementary file: main dataset and code (compressed) [file EMS198536-supplement-Supplementary_file__main_dataset_and_code__compressed_.zip › Covid-19-Teesside-main/Figures/GLMM/Lin45/Lin45-B117_GLMM_Obs-vs-Fit_AR1PC-AR1PCS2.png]

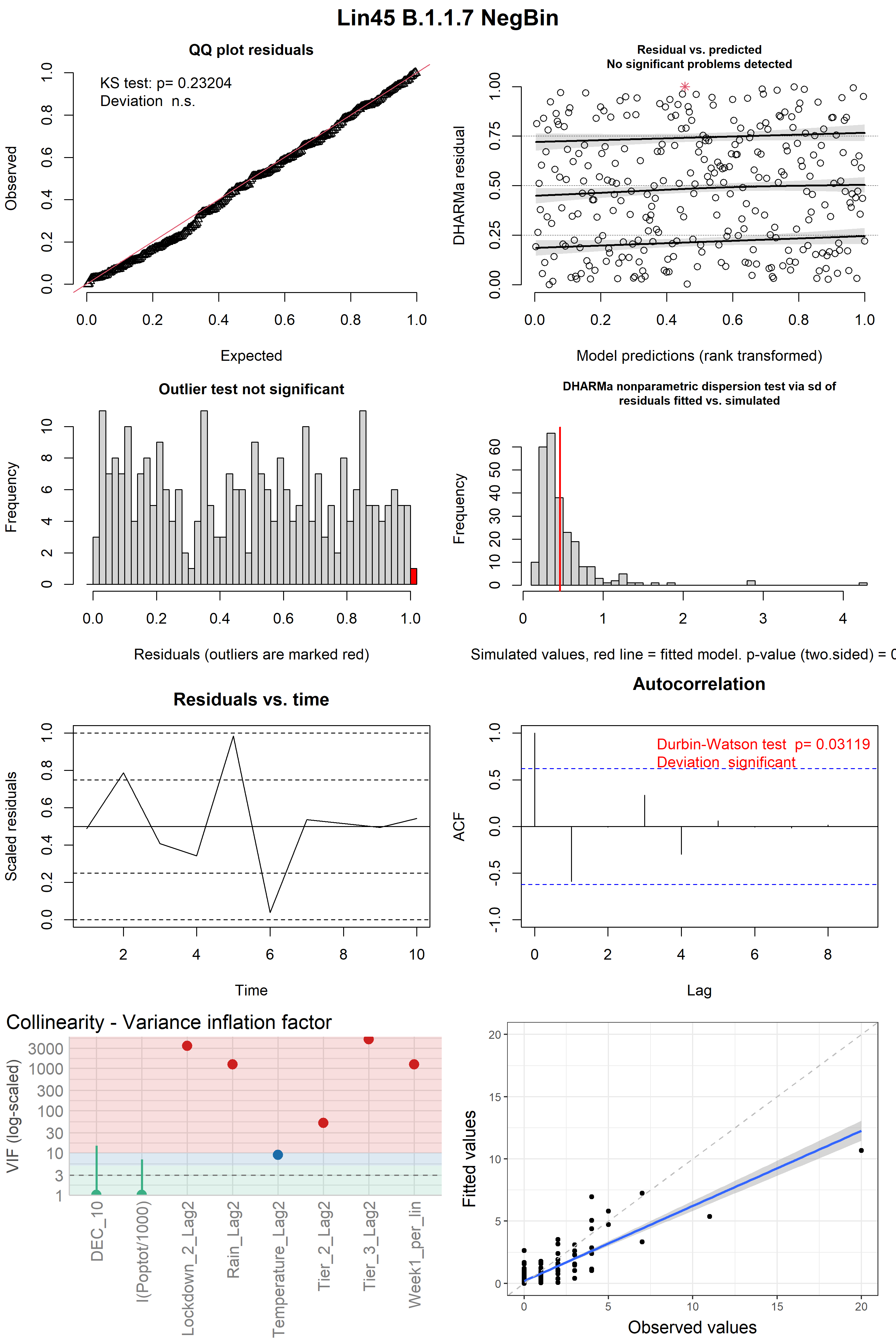

Supplement: Supplementary file: main dataset and code (compressed) [file EMS198536-supplement-Supplementary_file__main_dataset_and_code__compressed_.zip › Covid-19-Teesside-main/Figures/GLMM/Lin45/Lin45-B117_NB_Full_Fit.png]

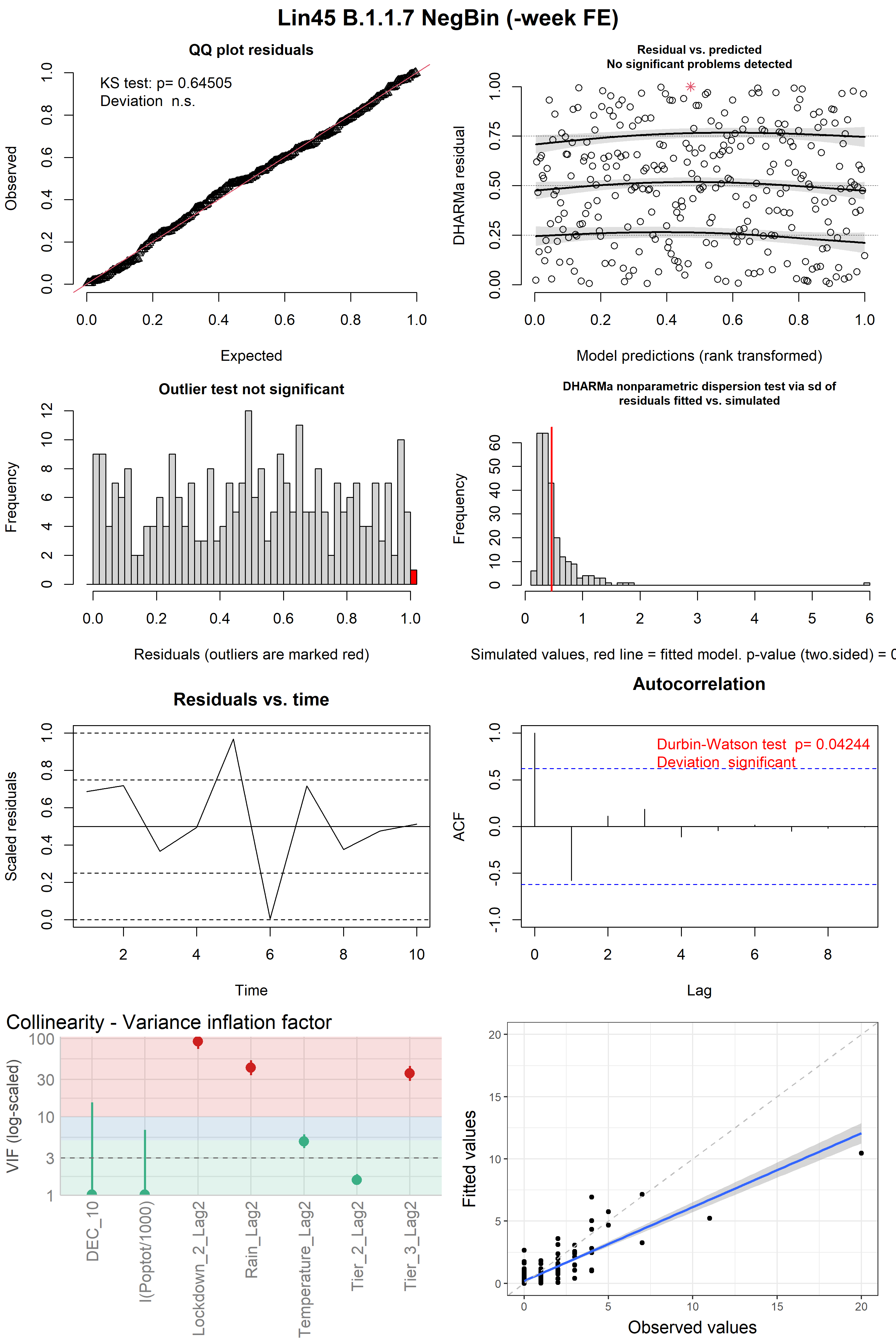

Supplement: Supplementary file: main dataset and code (compressed) [file EMS198536-supplement-Supplementary_file__main_dataset_and_code__compressed_.zip › Covid-19-Teesside-main/Figures/GLMM/Lin45/Lin45-B117_NB_No-week-FE_Fit.png]

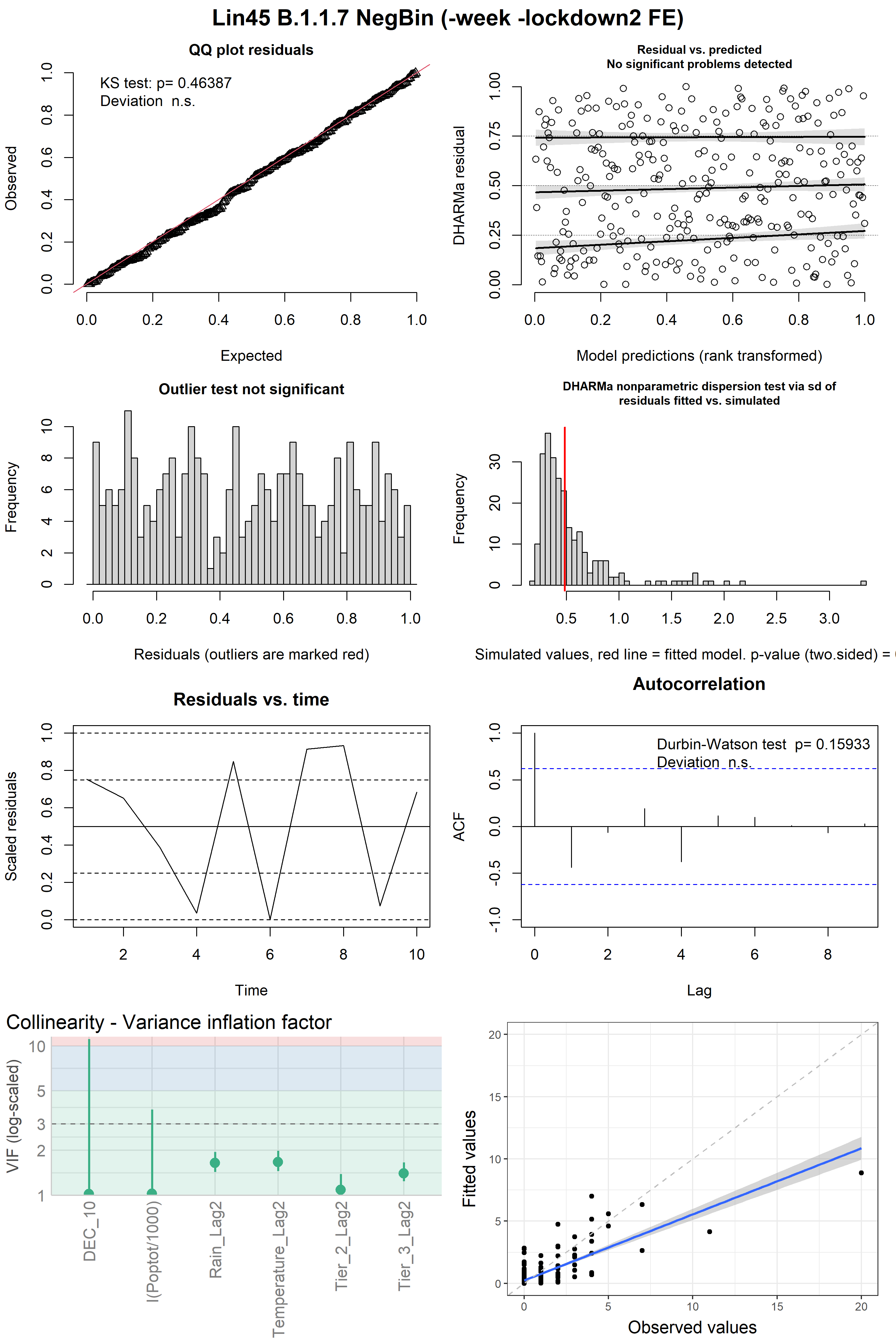

Supplement: Supplementary file: main dataset and code (compressed) [file EMS198536-supplement-Supplementary_file__main_dataset_and_code__compressed_.zip › Covid-19-Teesside-main/Figures/GLMM/Lin45/Lin45-B117_NB_No-week-no-lockdown2-FE_Fit.png]

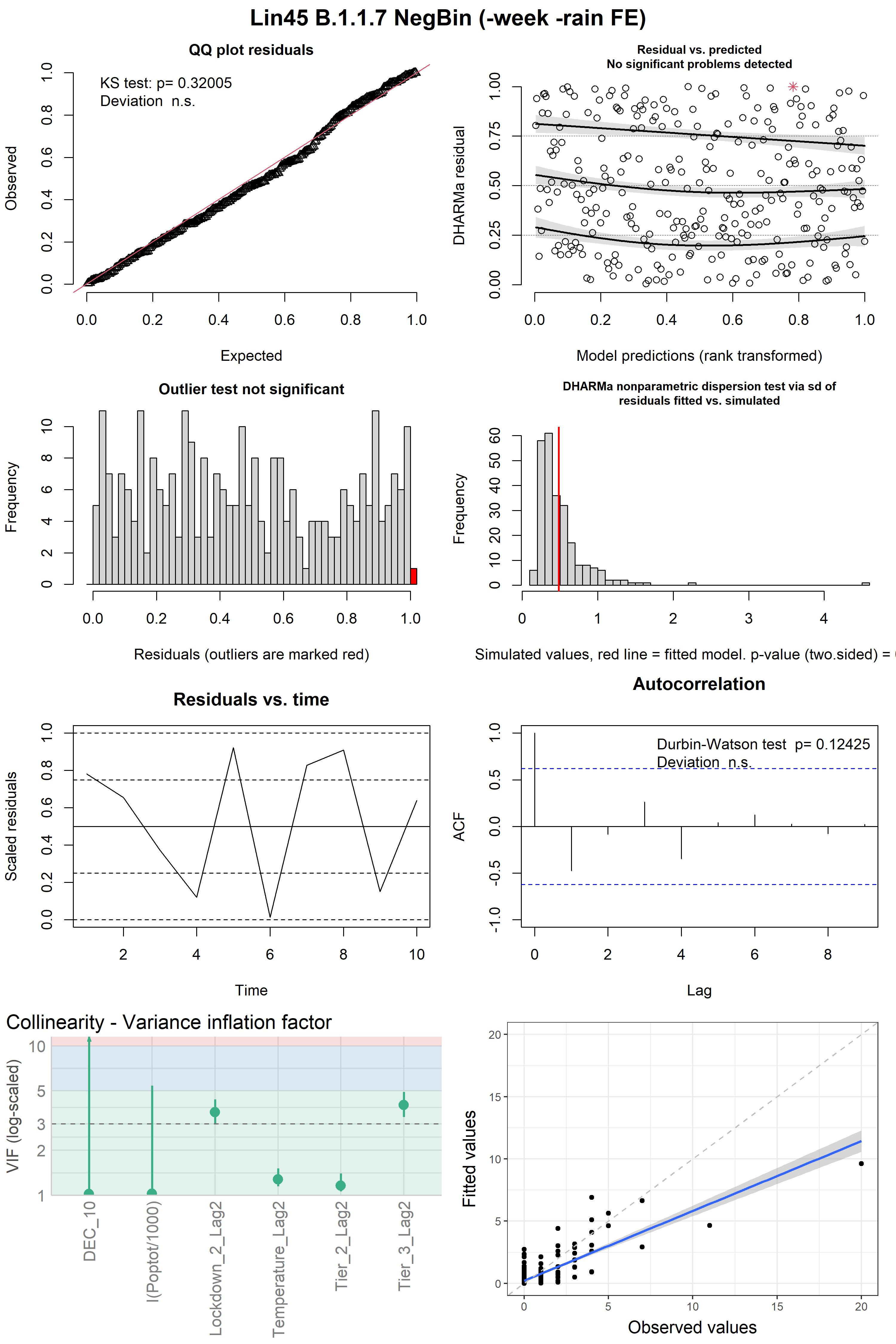

Supplement: Supplementary file: main dataset and code (compressed) [file EMS198536-supplement-Supplementary_file__main_dataset_and_code__compressed_.zip › Covid-19-Teesside-main/Figures/GLMM/Lin45/Lin45-B117_NB_No-week-no-rain-FE_Fit.png]

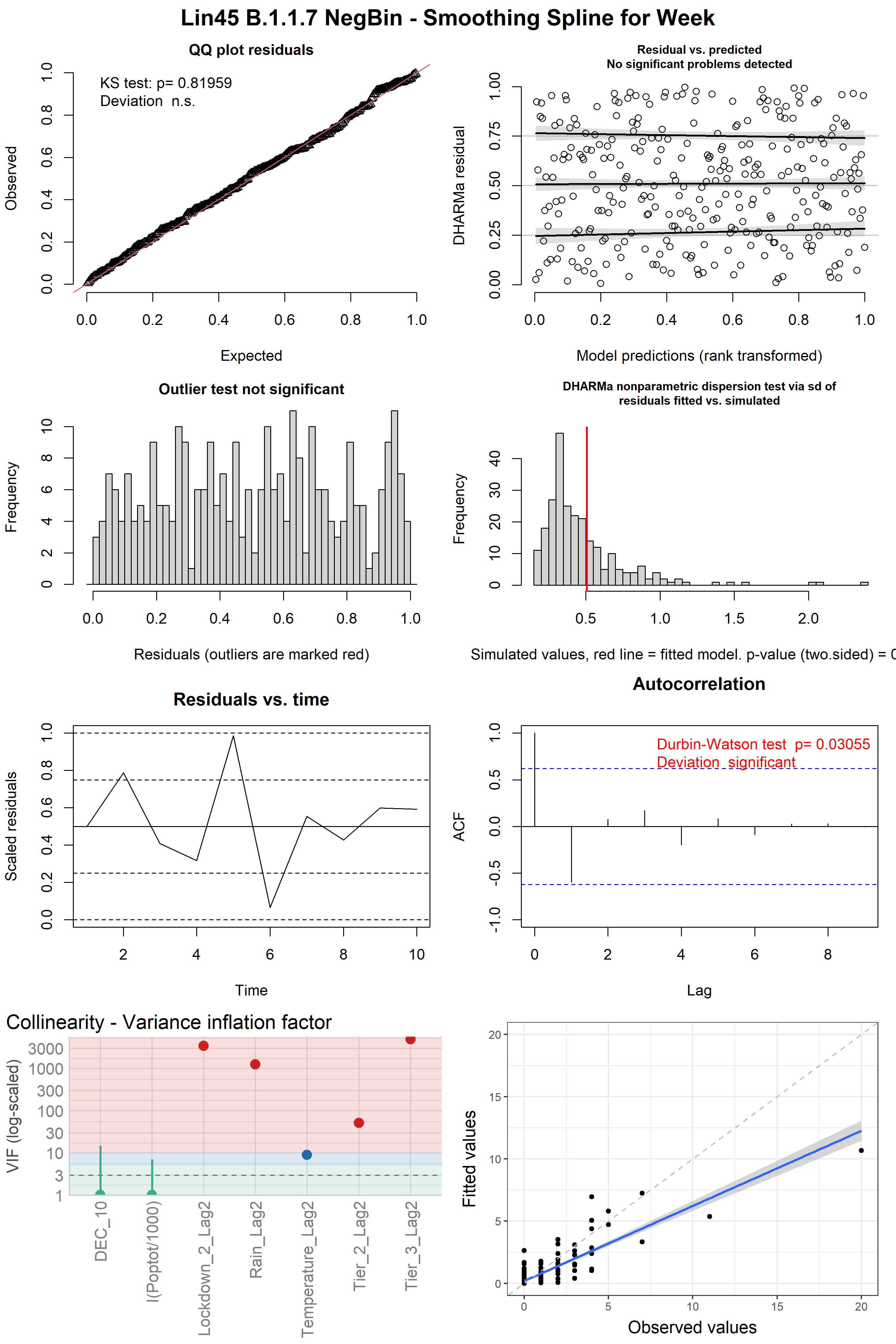

Supplement: Supplementary file: main dataset and code (compressed) [file EMS198536-supplement-Supplementary_file__main_dataset_and_code__compressed_.zip › Covid-19-Teesside-main/Figures/GLMM/Lin45/Lin45-B117_NB_SmoothSpline-Week-TPS_Fit.png]
